# Deprotonative C(*sp* ^3^)/C(*sp* ^2^)–H (Multi)Silylation of (Hetero)Arenes Mediated by NaTMP

**DOI:** 10.1002/anie.1774364

**Published:** 2026-04-09

**Authors:** David Sánchez‐Roa, Sophia Belrhomari, Ana McGinley, Clevin Anto Liju, Manting Mu, Max García‐Melchor, Eva Hevia

**Affiliations:** ^1^ Department Für Chemie, Biochemie und Pharmacie Universität Bern Bern Switzerland; ^2^ School of Chemistry Trinity College Dublin College Green Dublin 2 Ireland; ^3^ Parque Tecnológico de Álava Vitoria‐Gasteiz Spain; ^4^ Basque Foundation for Science Bilbao Spain

**Keywords:** coordination effects, mechanistic studies, metalation, silylation, sodium

## Abstract

The importance of organosilicon compounds in synthetic and materials chemistry has prompted the search for efficient and broadly applicable routes to these invaluable scaffolds, which often depend on scarce transition metals. In contrast, main group‐mediated strategies remain underdeveloped, with those reported generally limited to activated substrates and harsh reaction conditions. Here, a new sodium‐mediated protocol for deprotonative C(*sp* ^3^)/C(*sp* ^2^)–H silylation of (hetero)arenes is presented, which relies on the power of the strongly basic sodium amide NaTMP (TMP = 2,2,6,6‐tetramethylpiperidide) in combination with bulky chlorosilanes. This approach provides direct access to a myriad of silylated aromatic products, including toluene derivatives, non‐activated arenes such as benzene and naphthalene, pyridines and electron‐rich heterocycles. Mechanistic investigations, combining the isolation of key organometallic intermediates with theoretical calculations, underline the complementarity of NaTMP and the electrophilic chlorosilane, and reveal the steric and coordination effects that govern sodiation and silylation steps. Most notably, this protocol extends beyond conventional monosilylation, including orthogonal multisilylation of distinct C(*sp* ^3^) and C(*sp* ^2^)–H bonds, thereby broadening the scope of main group‐mediated arene functionalization.

## Graphical abstract

An operationally simple protocol using a strongly basic sodium amide in combination with sterically emcumbered silicon electrophiles enables the deprotonative C–H (multi)silylation of a myriad of (hetero)arenes. Mechanistic investigations highlight the pivotal role of steric and coordination effects in controlling the regioselectivity and efficiency of these reactions.

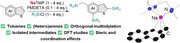

## 1 Introduction

Organosilicon compounds play a major role in synthetic chemistry [1, 2, 3, 4] and represent important motifs in drug discovery [5, 6], material sciences [7, 8] and biomedicine [9, 10]. The construction of new C─Si bonds is streamlined by the large natural availability of silicon, rendering the incorporation of silyl substituents into a diverse range of molecular architectures feasible. [11]. Direct C–H silylation of arenes constitutes one of the most attractive synthetic routes, bypassing the need of substrate prefunctionalization and improving atom economy [12]. To date, transition metal complexes mainly based on the precious metals Ir, Rh, or Ru have shown high activity [13], albeit the dependence on these scarce precious elements limits scalability and sustainability of these approaches.

**FIGURE 1:**
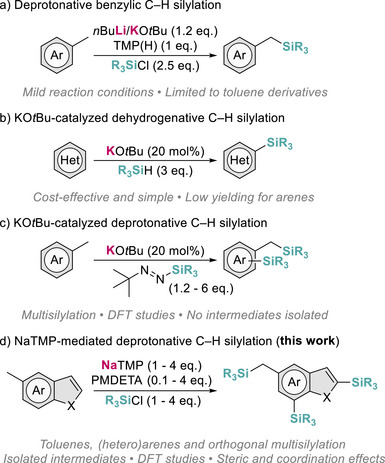
Main group strategies for the direct C(*sp* ^3^)/C(*sp* ^2^)–H silylation of (hetero)arenes and toluene derivatives.

Complementary main group strategies which combine the use of strong Brønsted bases such as organolithium reagents or Lochmann–Schlosser superbases with silyl electrophiles have also been reported [14, 15, 16, 17, 18]. However, these approaches typically require the use of cryogenic conditions and remain inefficient towards non‐activated arenes (in terms of p*K*_a_ values). Within benzylic functionalization, the *n*BuLi/KO*t*Bu/TMP(H) (TMP(H) = 2,2,6,6‐tetramethylpiperidine) system conceived by O'Shea stands as one of the most mild and effective stoichiometric procedures for C(*sp* ^3^)–H silylation (Figure 1a) [19]. Interestingly, KO*t*Bu has also emerged as a promising catalyst for C–H silylation, as demonstrated by Grubbs and Stoltz in cross‐dehydrogenative coupling of indoles and pyrazoles with hydrosilanes (Figure 1b) [20]. Despite its operational simplicity and cost‐effectiveness, this protocol encountered difficulties in the C(*sp* ^2^)–H and C(*sp* ^3^)–H silylation of arenes, forming the desired products in only moderate to low yields (< 50%). More recently, Chauvier has reported the catalytic deprotonative silylation of several heterocyclic motifs, fluoroarenes, and toluene derivatives, by combining KO*t*Bu with stoichiometric amounts of *tert*‐butyl‐substituted silyldiazenes (Figure 1c). This approach relies on the in situ generation of *t*BuK to achieve substrate deprotonation and silylation and has also enabled the multiple silylation of alkyl arenes at both benzylic and aromatic sites under mild conditions [21, 22]. Notably, while computational studies have provided valuable insights into these KO*t*Bu‐mediated protocols, the nature of the possible organometallic intermediates involved in these reactions remains elusive [22, 23, 24, 25].

Recently, we have reported that the tridentate donor *N*,*N*,*N*′,*N*″,*N*′’‐pentamethyldiethylenetriamine (PMDETA) enhances further the strong basicity of the sodium amide NaTMP enabling it to regioselectively borylate a broad range of aromatic substrates, including non‐activated arenes such as benzene and naphthalene, as well as catalyze hydrogen isotope exchange and alkene isomerization processes [26, 27, 28, 29]. Mechanistic studies on stoichiometric borylation reactions have revealed that PMDETA plays a key role by reducing aggregation and kinetically activating the sodium amide, while a sterically demanding boron electrophile is crucial to drive the metalation step and stabilize the resulting aryl sodium intermediates [27].

Building on these precedents and going beyond the current state of the art, here we present a sodium‐mediated protocol that enables regioselective deprotonative mono‐ and multisilylation of C(*sp* ^3^)/C(*sp* ^2^)─H bonds in a broad variety of aromatic and heteroaromatic substrates under mild, transition metal‐free conditions (Figure 1d). Through the isolation of key organometallic intermediates, complemented by spectroscopic monitoring and density functional theory (DFT) calculations, we establish the mechanistic basis of this transformation. These findings advance the understanding of sodium‐mediated metalation protocols and exemplify how steric and coordination effects can be harnessed to fine‐tune both regioselectivity and reactivity in main group‐driven silylation chemistry.

## 2 Results and Discussion

We initiated our investigations by examining the silylation of toluene as a model substrate. To this end, Et_3_SiCl was selected as the silicon electrophile and PMDETA as the Lewis donor to reduce aggregation and ramp up the reactivity of NaTMP in hexane. Since we have previously observed that metalation of weakly acidic substrates mediated by NaTMP is equilibrium‐controlled [27], we combined simultaneously the base, substrate and electrophile in a one‐pot process at room temperature, expecting the chlorosilane to drive the sodiation step forward. Under these reaction conditions, selective C–H silylation at the benzylic position afforded **1a** in a 75% yield (Table 1, entry 1). It should be noted that from our previous work on the perdeuteration of arenes we demonstrated the ability of NaTMP to also deprotonate toluene at the aromatic ring under catalytic regimes [28]; however, no other silylated products were detected, probably due to the greater thermodynamic stability of the benzyl anion [30]. The mild reaction conditions for the formation of **1a** contrast with those recently reported by Mao using LiHMDS, which require the use of toluene as a solvent, a large excess of base (3 eq. of LiHMDS in combination with 2 eq. of CsF) and extended high temperatures (130°C, 12 h) [18].

**TABLE 1:** Optimization studies for the C(*sp* ^3^)–H silylation of toluene.^a^

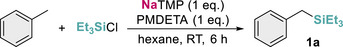		
Entry	Deviation from standard conditions	NMR yield [%]^b^
1	None	75
2	***n*BuLi** instead of NaTMP	0
3	**LiTMP** instead of NaTMP	18
4	**KTMP** instead of NaTMP	62
5	**NaHMDS** instead of NaTMP	0
6	**NaCH_2_SiMe_3_** instead of NaTMP	0
7	**LiTMP/NaO*t*Bu** instead of NaTMP	62
8	***n*BuLi/NaO*t*Bu/TMP(H)** instead of NaTMP	69
9	**TMEDA** instead of PMDETA	54
10	**10 mol%** of PMDETA	84
11	**Me_3_SiCl** instead of Et_3_SiCl	0^c^
12	***i*Pr_3_SiCl** instead of Et_3_SiCl	90^c^
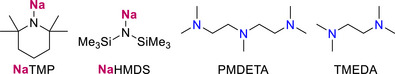		

^a^ Reaction conditions by addition order: NaTMP (0.5 mmol), hexane (3 mL), Et_3_SiCl (0.5 mmol), toluene (0.5 mmol), PMDETA (0.5 mmol).

^b^ NMR yields calculated using 1,1,2,2‐tetrachloroethane as internal standard.

^c^ These yields refer to the formation of **1a(Me)** or **1a(*i*Pr)** instead of **1a**.

The crucial role of NaTMP became evident when other alkali‐metal bases were examined (Table 1, entries 2–6). The common organolithium reagent *n*BuLi completely failed under these mild conditions, while LiTMP and KTMP were significantly less reactive in the presence of PMDETA (18% and 62% respectively). These results reveal a pronounced alkali‐metal effect, with sodium outperforming potassium and especially lithium. Moreover, two additional sodium bases were tested, highlighting the prominence of the TMP moiety. Thus, less basic NaHMDS cannot metalate the substrate at all [31, 32, 33], whereas alkylsodium NaCH_2_SiMe_3_ also failed to deliver any silylated product, presumably due to its rapid decomposition in the presence of the silicon reagent. With the importance of NaTMP firmly established, we investigated its in situ generation through two approaches (Table 1, entries 7 and 8). Although slightly less efficient, it should be noted that the combination of LiTMP with NaO*t*Bu proved competent in the benzylic silylation of toluene (62%), whereas the *n*BuLi/NaO*t*Bu/TMP(H) mixture gave comparable results to those when using isolated NaTMP (69% vs. 75%).

Coordination effects proved important as evidenced when PMDETA was replaced by bidentate TMEDA since the change resulted in a significant decrease in yield (54%, Table 1, entry 9) [28, 29]. Interestingly, when using substoichiometric amounts of PMDETA (10 mol%) a slightly higher yield was observed when compared to the standard conditions (84 vs. 75%). These results suggest that small amounts of this N‐donor are sufficient to partially disrupt NaTMP aggregation, activating it towards fulfilling the substrate metalation.

The chlorosilane partner also had a pronounced impact on the process (Table 1, entries 11–12). No traces of silylated product were found when employing the less encumbered chlorosilane Me_3_SiCl, whereas the bulkier *i*Pr_3_SiCl furnished **1a(*i*Pr)** in an excellent yield (90%) making it superior to when using Et_3_SiCl (75%). These results hint at the presence of a competing decomposition pathway between the chlorosilane and NaTMP, which appears to be highly dependent on the steric environment around the silicon atom. Consistent with this hypothesis, NMR monitoring of NaTMP with the different chlorosilanes in the presence of PMDETA revealed immediate decomposition for Me_3_SiCl with concomitant formation of TMP(H), while *i*Pr_3_SiCl remained unreactive toward NaTMP over 24 h at room temperature. In the case of Et_3_SiCl, there were only partial side reactions over the course of 2 h (*see* Supporting Informatiom *for more details)*. This competing decomposition pathway may explain the improved performance of the system when using catalytic loadings of PMDETA, reducing the amount of NaTMP present in solution, which can promote the background decomposition reaction with unreacted Et_3_SiCl.

**FIGURE 2:**
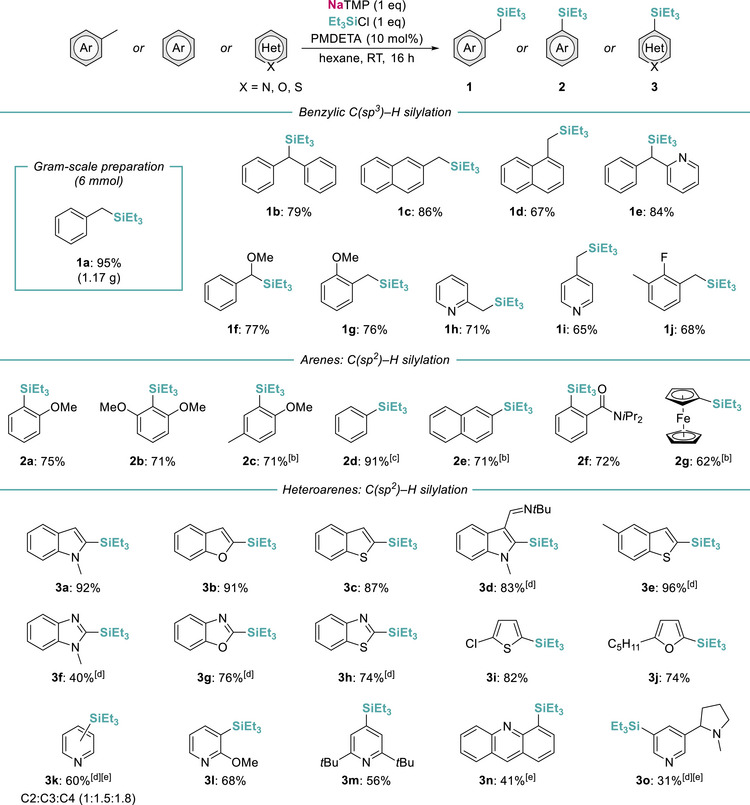
Substrate scope of NaTMP‐mediated C–H silylation of (hetero)arenes including isolated yields. [a] Reaction conditions by addition order: NaTMP (0.5 mmol), hexane (3 mL), Et_3_SiCl (0.5 mmol), substrate (0.5 mmol), PMDETA (0.05 mmol). [b] 0.75 mmol (1.5 eq.) of substrate [c] Benzene used as solvent. [d] NMR yield calculated using 1,1,2,2‐tetrachloroethane as internal standard. [e] Reaction performed at −30°C.

The scope of the protocol was evaluated toward a range of toluene derivatives, increasing the reaction times to 16 h to ensure full consumption of the substrates (Figure 2). Despite the partial decomposition of NaTMP in the presence of Et_3_SiCl, we decided to use this chlorosilane due to its higher cost‐effectiveness and the well‐established versatility of the SiEt_3_ group for further post‐functionalization steps [34, 35]. Consistent with the observations from the formation of **1a**, this competing degradation pathway had a minor impact, allowing for the regioselective C(*sp* ^3^)–H silylation of a wide range of substrates. The incorporation of a phenyl ring, either in the benzylic position or fused as a naphthyl, afforded silylated products **1b**‐**d** in good yields after isolation (79%, 86%, and 67% respectively). Interestingly, while 2‐benzylpyridine gave **1e** in a 84% yield, no traces of silylated products were observed for its 4‐benzyl isomer. In all cases, the SiEt_3_ group was installed selectively at the benzylic site, including substrates bearing *ortho*‐directing methoxy substituents and electron‐deficient 2‐ and 4‐picolines furnishing **1f‐1i** in yields ranging from 65% to 77% (Figure 2). Moreover, compound **1j** was obtained in a 68% yield from benzylic silylation of 2,6‐dimethylfluorobenzene without showing significant evidence of decomposition (*vide infra*). In addition, the practicality of the process was demonstrated further by scaling the reaction with toluene to a 6 mmol scale, affording **1a** in a 95% yield (1.17 g).

Having established efficient C(*sp* ^3^)–H silylation, we next examined whether similar conditions could enable functionalization of aromatic C(*sp* ^2^)─H bonds. Selective *ortho‐*silylation of anisole could be accomplished affording **2a** in a 75% isolated yield. Likewise, compounds **2b** and **2c** were obtained (both in a 71% yield) from the silylation of 1,3‐dimethoxybenzene and 4‐methylanisole respectively, which occurs selectively at the C─H bond adjacent to the OMe groups. Interestingly, it should be noted that for 4‐methylanisole, **2c** was obtained in combination with a small amount of the disilylated product resulting from *ortho* and benzylic metalation (ca. 70% mono‐ vs. 20% di‐silylated). However, when the reaction was conducted with a 0.5 eq. excess of the substrate, the second silylation step can be suppressed. Breaking new ground in the C─H silylation of arenes, we achieved the transformation of benzene and naphthalene, known inaccessible substrates due to the absence of directing groups. Formation of triethylsilylbenzene **2d** required using benzene as solvent, which boosted the initial sodiation step to afford the desired product in a 91% isolated yield. On the other hand, silylation of naphthalene proved totally regioselective towards the C2‐position to give compound **2e** (71% yield), although some 2,6‐disilylated material was also recovered. *N*,*N*‐diisopropylbenzamide proved compatible with this protocol yielding **2f** in a 72% yield. This is most likely due to steric protection incorporated by the isopropyl groups, since *N*,*N*‐dimethylbenzamide decomposed under reaction conditions. Reaction with ferrocene formed monosilylated product **2g** in a 62% yield, although a small amount of the 1,1’‐disilylated derivative was also observed.

The protocol also showed excellent results in the C(*sp* ^2^)–H silylation of electron‐rich heterocycles such as 1‐methylindole, benzofuran, and benzothiophene. Near‐quantitative yields were obtained for compounds **3a–c**, which we attribute to the higher acidity of the C2‐position [33], reducing the competing decomposition of NaTMP and the chlorosilane. The enhanced stability of the C2‐sodiated intermediate was further evidenced by successful silylation of an imino‐substituted indole to give **3d** (83% yield). Moreover, the superior acidity of this position conditions regioselectivity, as observed for 5‐methylthiophene, where the benzylic position remains unreacted upon metalation rendering C2‐silylated product **3e** (96% yield). Additional heterocyclic motifs were tested toward silylation, including 1,3‐benzoazoles, 2‐chlorothiophene, and 2‐pentylfuran, which afforded compounds **3f–j** with complete selectivity at their C2‐position (40%–82% yields). Electron‐deficient systems such as pyridine derivatives proved more challenging since their metalation rapidly promoted decomposition. In recent work, Thomas has shown that controlled metalation of pyridine is feasible under cryogenic conditions employing *n*BuNa, giving mainly C4‐metalation after equilibration time, whereas under these conditions NaTMP failed to give any conversion after electrophilic quenching [36]. We observed the same lack of reactivity on treating pyridine with our NaTMP, PMDETA, and Et_3_SiCl combination at −78°C. However, increasing the temperature to −30°C provided a significant improvement on the sodium‐mediated silylation process, rendering **3k** in a 60% yield which contains a 1:1.5:1.8 mixture of the C2, C3, and C4 silyl‐substituted pyridine (see Supporting Informatiom for more details). Introducing a directing group such as OMe provides a better control of the regioselectivity of the Na/H exchange process and allows for the use of room temperature conditions, as shown for 2‐methoxypyridine which furnished **3l** in a 68% yield resulting from the metalation of its C3 site. A similar effect is observed if sterically demanding groups are sited at the C2 and C6 positions, thus 2,6‐di‐*tert*‐butylpyridine is selectively functionalized at its C4 position to give **3m** in a 56% yield. In addition, acridine and nicotine could also undergo regioselective silylation to give **3n** and **3o** in a 41% and 31% yield respectively, although strict control of the temperature (−30°C) is required to partially avoid decomposition of the highly sensitive metalated intermediates.

While these results highlight the versatility and efficiency of NaTMP in deprotonative C–H silylation across diverse (hetero)aromatic frameworks, for completeness, it should be noted that some substrates investigated were not compatible with this approach. Thus, decomposition products were obtained with aromatic nitriles, ketones, and esters, even with strict control of the temperature. This is attributed to the decomposition of the sodiated intermediates prior to the electrophilic interception. Similarly, fluoroarenes could not be tolerated due to the lack of stability of the relevant metalated products, which rapidly eliminate NaF, forming a transient benzyne intermediate, which in the case of 4‐fluoroanisole, could be trapped with 1,3‐diphenylisobenzofuran (see Supporting Informatiom for more details).

**FIGURE 3:**
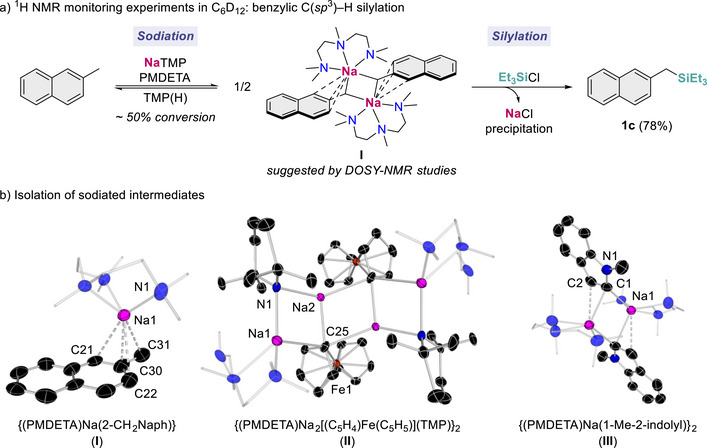
Mechanistic investigations of NaTMP‐mediated deprotonative silylation. (a) ^1^H NMR monitoring experiments for the C(*sp* ^3^)–H silylation of 2‐methylnaphthalene. (b) Molecular structures of {(PMDETA)Na(2‐CH_2_Naph)} (**I**), {(PMDETA)Na_2_[(C_5_H_4_)Fe(C_5_H_5_)](TMP)}_2_ (**II**) and {(PMDETA)Na(1‐Me‐2‐indolyl)}_2_ (**III**) with 30% probability displacement ellipsoids. Hydrogen atoms have been omitted for clarity.

To help shed light on the mechanism of the silylation process, we monitored the formation of the corresponding sodiated intermediates. First, we combined 2‐methylnaphthalene (2‐CH_3_Naph) with a stoichiometric amount of NaTMP and PMDETA in C_6_D_12_ (Figure 3a). While sodiated compound {(PMDETA)Na(2‐CH_2_Naph)} (**I**) was formed, only 50% conversion was observed, which contrasts with the high isolated yield of the intended silylation product **1c** (86%, Figure 2). Monitoring this reaction over 24 h showed no further conversion of the substrate to **I**, which is consistent with reaching an equilibrium for the Na/H exchange process (see Supporting Informatiom for more details). Analysis of the reaction mixture by diffusion‐ordered ^1^H NMR Spectroscopy (DOSY) suggested a dimeric arrangement for **I** in solution (*see* Supporting Information *for more details*). Remarkably, addition of one equivalent of Et_3_SiCl led to the formation of the silylated product **1c** in a 78% yield. These findings can be explained by the fact that the chlorosilane reacts selectively with **I** instead of {(PMDETA)Na(TMP)} which is largely favored by the steric incompatibility of the base and the silicon electrophile, shifting the equilibrium towards the sodiation of 2‐methylnaphthalene.

The structure of intermediate **I** was established by X‐ray crystallographic studies (Figure 3b) [37]. Notably, while in solution **I** adopts a dimeric motif (*vide supra*), in the solid state it forms a monomeric arrangement, where the {Na(PMDETA)}^+^ cation adopts a perpendicular disposition to the aromatic naphthalene ring, binding to the benzylic carbon that has undergone metalation [Na−C31, 2.779(11) Å] as well as *π*‐engaging with the *ipso*‐C [Na1−C30, 2.590(8) Å] and the two other aromatic carbons, which are *orth*o‐disposed to C30 [Na1−C21, 2.944(7) Å, Na1−C22, 2.908(7) Å]. These short‐medium Na−C contacts are consistent with Na gaining significant *π*‐interactions from the negative charge of the benzyl anion delocalized on the naphthyl ring [38, 39, 40].

Similar reactivity studies were performed with two other model substrates, namely ferrocene and *N*‐methylindole. Showcasing the structural and constitutional diversity of the organometallic intermediates involved in these reactions, in the case of ferrocene, formation of mixed aggregate {(PMDETA)Na_2_[(C_5_H_4_)Fe(C_5_H_5_)](TMP)}_2_ (**II**) was observed, isolable in crystalline form. In the solid state **II** exhibits a centrosymmetric dimeric arrangement, featuring a ladder motif made up of outer Na−N rungs and inner Na−C rungs (Figure 3b) [41]. This structure can also be envisaged as two dinuclear {NaNNaC} rings, which combine laterally via their Na−C edges to generate this tetranuclear ladder. Within this motif, the outer Na atom (Na1) is coordinated by a PMDETA ligand, which binds in a bidentate fashion employing only two of its N atoms. As far as we can ascertain **II** constitutes the first example of direct sodiation of a metallocene. Its formation can be rationalized considering the 2:2 co‐complexation of the metalation product {Na[(C_5_H_4_)Fe(C_5_H_5_)]} with the sodium base {(PMDETA)Na(TMP)}. It should be noted that previous studies from our group have also noted the formation of related mixed aggregates when reacting NaTMP with non‐activated arenes such as anisole [27] or benzene [28].

When the sodiation of *N*‐methylindole was investigated, isolation of {(PMDETA)Na(1‐Me‐2‐indolyl)}_2_ (**III**) in a 41% yield was observed. X‐ray crystallographic studies established the dimeric structure of **III** in which both Na atoms are connected by two bridging C2‐metallated indolyl anions, forming a planar four‐membered {NaCNaC} ring (sum of internal angles 360°) with a Na─C bond distance of 2.626(14) Å. Each sodium attains further coordinative stabilization by bonding to tridentate PMDETA and also by forming a long distance *π*‐electrostatic interaction with the carbon located next to the one that has undergone metalation [Na1−C2, 3.018(15) Å].

**FIGURE 4:**
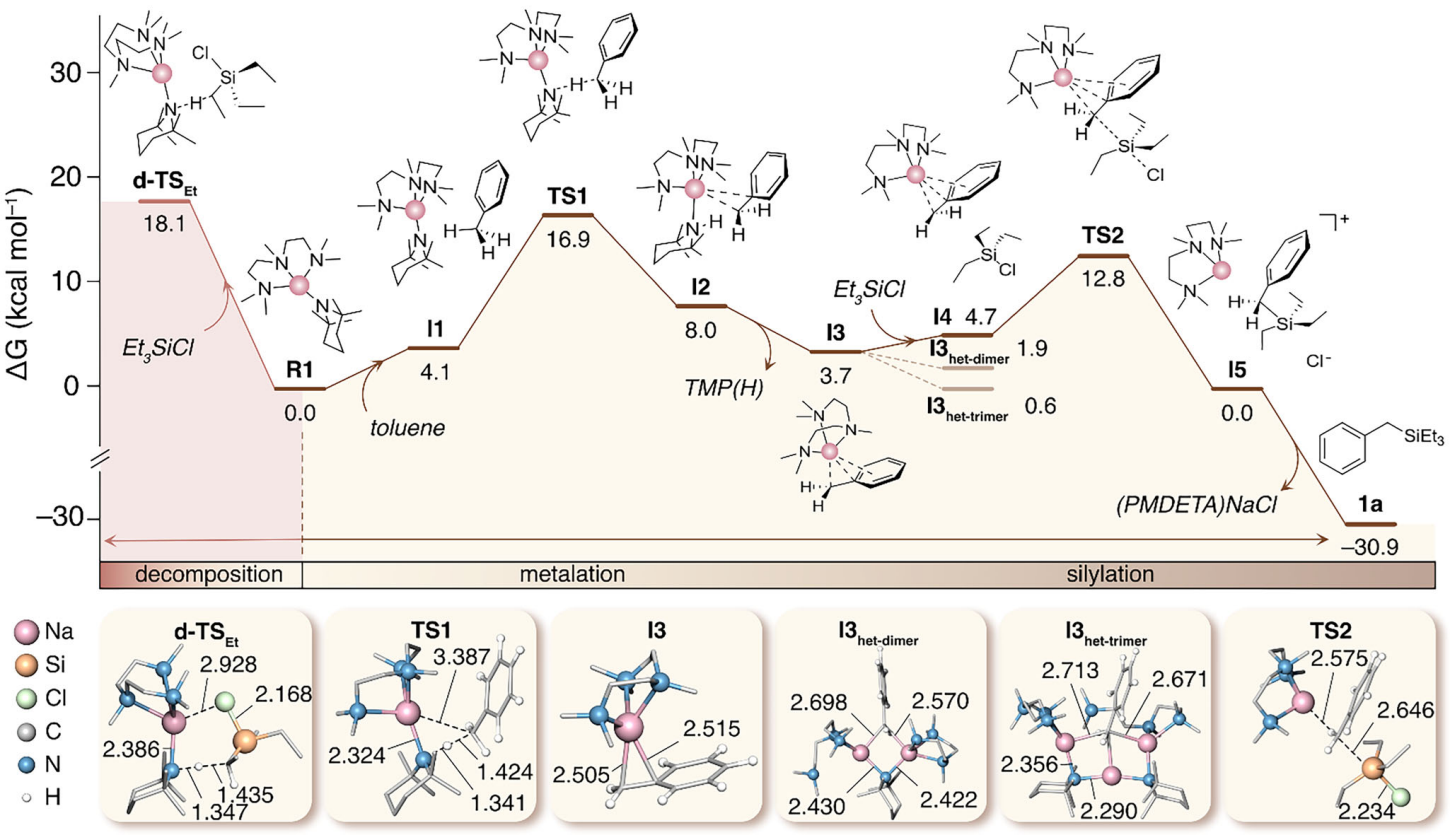
Gibbs energy profile for the silylation of toluene with Et_3_SiCl to form **1a** via the monomeric pathway (right) and for the decomposition of Et_3_SiCl with {(PMDETA)Na(TMP)} (**R1**) (left). Gibbs energies are reported in kcal·mol^−1^ relative to **R1** under experimental conditions (298.15 K, 1 atm, *n*‐hexane). Insets show key intermediates and transition states with selected bond lengths in Å. Hydrogen atoms are omitted for clarity where appropriate.

With the aid of DFT calculations, we next investigated the possible decomposition of NaTMP·PMDETA in the presence of chlorosilanes. Calculations were performed at the *ω*B97X‐D level (see Supporting Informatiom for more details). Building on our prior finding that {(PMDETA)NaTMP} (**R1**) coexists with its dimer {(PMDETA)Na(TMP)}_2_ in solution with a slight preference for the monomer (ΔG_solv_  = −0.2 kcal·mol^−1^) [29], we considered **R1** as the active species in the following analysis. We first examined the decomposition pathways as a function of chlorosilane sterics (Figure 4, left pathway, and S135). For Et_3_SiCl, two plausible reactions were evaluated: nucleophilic attack of NaTMP at the Si─Cl bond and deprotonation at the *α*‐carbon, both of which computed to be exergonic (Figures S134 and S135). The deprotonation transition state (**d‐TS_Et_**) lies much lower in energy than the nucleophilic attack transition state (**d‐TS_Et,A_**, Figure S134; +18.1 vs. +31.3 kcal·mol^−1^), indicating that chlorosilane deprotonation is the dominant competing pathway. Analogous deprotonation barriers were computed for Me_3_SiCl and *i*Pr_3_SiCl (**d‐TS_Me_** and **d‐TS_iPr_**) at +14.1 and +24.5 kcal·mol^−1^, respectively (Figure S134). Consistent with our experimental findings (*vide supra*), these results follow the logic that increasing steric hindrance around silicon minimizes possible competing decomposition of NaTMP by raising the barrier for deprotonating the chlorosilane, highlighting the importance of steric factors in this sodium‐mediated silylation processes. To support this decomposition pathway, we analyzed in more detail the NMR monitoring reaction between Me_3_SiCl with NaTMP·PMDETA (*vide supra*). Along with the concomitant formation of TMP(H), mass spectrometry suggests the formation of the silene Me_2_Si = CH_2_ after deprotonation of Me_3_SiCl and NaCl elimination, which potentially undergoes trimerization to give the major peak of the mass spectrum at 201.09 *m*/*z* (C_8_H_21_Si_3_ after the loss of a methyl radical, Figure S123).

Similarly for the studies described above for 2‐methylnaphthalene, monitoring by ^1^H NMR spectroscopy the reaction of toluene with equimolar amounts of NaTMP and PMDETA in C_6_D_12_ showed that the metalation reaction gave only a modest 30% conversion. While the metalated intermediate could not be isolated as a crystalline solid, DOSY NMR studies of the reaction mixture are consistent with the formation of the mixed aggregate intermediate {(PMDETA)_2_Na_3_(TMP)_2_(CH_2_Ph)} (see Supporting Informatiom for more details). Addition of Et_3_SiCl led to the almost immediate quantitative formation of **1a** along with the precipitation of NaCl.

This reaction was also studied by DFT calculations (Figure 4, right pathway). The lowest transition state (**TS1**) for the metalation of toluene by **R1** has an accessible barrier of +16.9 kcal·mol^−1^ (see Supporting Informatiom for more details). Notably, **TS1** lies between the energies calculated for the TS of the decomposition pathways **d‐TS_Me_** and **d‐TS_Et_** (+14.1 and +18.1 kcal·mol^−1^, *vide supra*). These findings rationalize the experimentally observed competition between substrate metalation and electrophile decomposition, while the higher **d‐TS*_i_*_Pr_** (+24.5 kcal·mol^−1^) explains the reduced background reactivity with *i*Pr_3_SiCl. Relaxation from **TS1** and subsequent dissociation of TMP(H) yield the benzyl sodium intermediate **I3**, which is endergonic by +3.7 kcal·mol^−1^. Aggregation of **I3** to form dinuclear {(PMDETA)_2_Na_2_(TMP)(CH_2_Ph)} (**I3_het‐dimer_**) and {(PMDETA)Na(CH_2_Ph)}_2_ (**I3_homo‐dimer_**) and trinuclear {(PMDETA)_2_Na_3_(TMP)_2_(CH_2_Ph)} (**I3_het‐trimer_**) species was also examined (see Supporting Information for more details). The **I3_het‐trimer_** is the most stable at +0.6 kcal·mol^−1^ relative to **R1**. These species lie close in energy, suggesting that they may coexist and interconvert dynamically with {(PMDETA)Na(TMP)} (**R1**) in solution.

Combination of the monomeric **I3** with Et_3_SiCl proceeds via **I4** to **TS2** with a barrier of +12.8 kcal·mol^−1^. The C_benzylic_–Si–Cl angle of 176.6° in **TS2** is consistent with a concerted S_N_^2^ reaction at silicon with backside approach, minimizing steric repulsion between the electrophile and the {(PMDETA)Na}^+^ moiety. Corresponding pathways for the aggregates were also computed (Figure S134). The **I3_het‐dimer_** (**TS2_D_**) and **I3_het‐trimer_** (**TS2_E_**) routes are higher at +16.2 and +23.8 kcal·mol^−1^, respectively, whereas the **I3_homo‐dimer_** route (**TS2_dimer_**, Figure S138) is slightly lower at +10.7 kcal·mol^−1^, comparable to the monomeric **TS2**. Because several silylation transition states are relatively close in energy, we performed microkinetic modelling to assess their contributions (see Supporting Informatiom for more details). The simulated concentration‐time profiles indicate that product formation occurs almost exclusively through the monomeric pathway (>99%, Figures S139 and S140), with negligible dimeric contribution. Since **TS2** is lower than **TS1**, benzylic deprotonation/sodiation is rate determining, whereas silylation shifts the equilibrium toward the products. From **TS2**, chloride substitution by the benzyl anion gives **I5**, which rapidly evolves into the final silylated product **1a** and {(PMDETA)NaCl}. Overall, their formation renders the sodiation/silylation process exergonic by −30.9 kcal·mol^−1^, with additional stabilization expected from NaCl precipitation as the driving force of the overall process.

**FIGURE 5:**
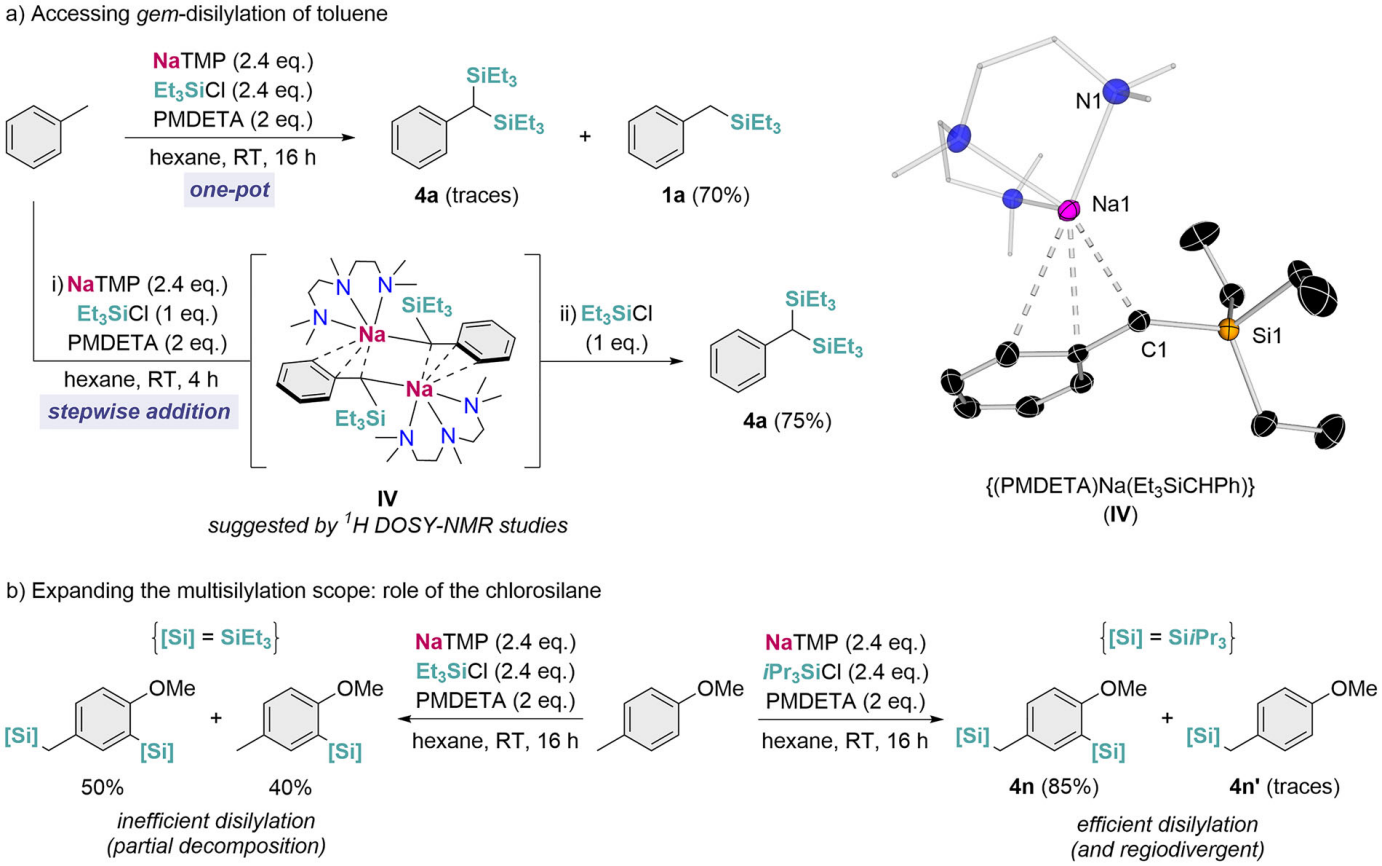
Initial studies of NaTMP‐mediated deprotonative multisilylation of arenes. (a) One‐pot vs. stepwise *gem*‐disilylation of toluene and molecular structure of {(PMDETA)Na(Et_3_SiCHPh)} (**IV**) with 30% probability displacement ellipsoids. Hydrogen atoms have been omitted for clarity. (b) Influence of the chlorosilane in the orthogonal disilylation of 4‐methylanisole.

Based on the mechanistic understanding deduced from both experimental and computational studies, we next pondered if this sodium mediated approach could be extended to promote regioselective multisilylation of C─H bonds. We first attempted the *gem*‐disilylation of toluene at the benzylic position. Increasing the amount of NaTMP and Et_3_SiCl to 2.4 eq. to facilitate the double metalation and silylation steps gave mainly monosilylated compound **1a**, with only traces of the desired disilylated material (Figure 5a). Prior work by Chauvier showed that *gem*‐disilanes formation is highly sensitive to the steric hindrance of the silyl substituent, furnishing excellent results for the less encumbered SiMe_3_ but negligible yields for SiEt_3_ [22]. Intrigued by this observation, we investigated whether the limitation in our system arose from the second metalation or silylation steps once the first silylated product **1a** had been formed. For that purpose, we monitored the sodiation of **1a** by adding 1 eq. of NaTMP and PMDETA in C_6_D_12_ (Figure 5a). ^1^H NMR spectra of the reaction revealed significant consumption of both the base and the substrate, furnishing the sodiated intermediate {(PMDETA)Na(Et_3_SiCHPh)} (**IV**) and TMP(H). As previously discussed for toluene and 2‐methylnaphthalene, an equilibrium for the metalation process was observed, although in this case **IV** is the major species present in solution (75% conversion). This is attributed to the higher stabilization of the carbanion geminal to the silicon atom. The molecular structure of **IV**, which was established by x‐ray crystallography, displays a monomeric motif where the Na atom binds the substrate in an allylic‐type fashion, similar to that previously described for **I**.

After confirming that the second metalation step is feasible, stepwise addition of 1 eq. of Et_3_SiCl to the mixture, delivered *gem*‐disilylated product **4a** in a 75% yield. This result contrasts sharply with our previous observations using a one‐pot approach. A possible explanation for this disparity in the results could be that performing the reaction under one‐pot conditions switches on the competing decomposition pathway of the sodium base with Et_3_SiCl. To gain a deeper understanding of this process, we also conducted additional DFT studies on the second sodiation and silylation steps (Figure S142).

Starting from **R1** and **1a**, the second proton transfer via **TS3** closely resembles **TS1** but is slightly higher at +17.1 kcal·mol^−1^, so premature Et_3_SiCl decomposition can compete when both base and electrophile are present at doubled concentrations. Consequently, the one‐pot, single‐step protocol favors early decomposition of NaTMP and chlorosilane over productive sodiation/silylation, consistent with the poor experimental yield of **4a**. In contrast, the one‐pot, stepwise protocol minimizes side reactivity. Formation of **IV** (**I8**) is exergonic by −1.3 kcal·mol^−1^, in accordance with the enhanced stability of the *α*‐silyl carbanion. Dimerization to {(PMDETA)Na(Et_3_SiCHPh)}_2_ (**I8_dimer_**) provides additional stabilization, rendering the second sodiation exergonic by –9.8 kcal·mol^−1^. This dimeric species was also found to be present experimentally by DOSY studies (see Supporting Informatiom for more details), thus supporting the DFT predictions. For the second silylation, the dimeric pathway is now favored: **TS4_dimer_** has a barrier of +14.8 kcal·mol^−1^ (Figure S134), whereas the analogous monomeric **TS4_B_** is higher at +25.0 kcal·mol^−1^. Release of {(PMDETA)NaCl} furnishes the disilylated product **4a**, and the overall second silylation is exergonic by −30.3 kcal·mol^−1^, again driven by NaCl precipitation.

To assess broader versatility, we next targeted multisilylation at distinct positions within a single substrate. Treating 4‐methylanisole with 2.4 eq. each of NaTMP and Et_3_SiCl resulted in partial conversion to the disilylated product functionalized in the benzylic and the *ortho*‐position to the methoxy group (Figure 5b). However, this transformation remained inefficient (50% disilylation and 40% monosilylation), mainly due to partial decomposition of the NaTMP by the silicon electrophile. To circumvent this side process, we replaced the silyl reagent with the more sterically encumbered *i*Pr_3_SiCl, which afforded disilylated product **4n** in a 85% yield with only traces of monosilylation. Notably, this residual material **4n’** was functionalized in the benzylic position, which diverges from the result obtained on using Et_3_SiCl (**2c**). This outcome indicates a strong steric influence of the chlorosilane in the process. Compound **4n’** was independently prepared by using 1 eq. of NaTMP and *i*Pr_3_SiCl in a 87% isolated yield, highlighting the role of the electrophile to modulate the regioselectivity in the final product.

**FIGURE 6:**
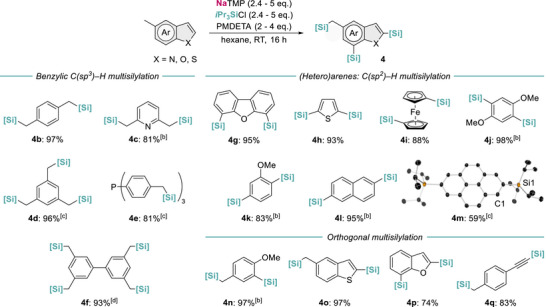
Substrate scope of NaTMP‐mediated C─H multisilylation of (hetero)arenes including isolated yields. (a) Reaction conditions by addition order: NaTMP (1.2 mmol, 2.4 eq.), hexane (3 mL), *i*Pr_3_SiCl (1.2 mmol, 2.4 eq.), substrate (0.5 mmol), PMDETA (1 mmol). (b) 1.5 mmol (3 eq.) of NaTMP and *i*Pr_3_SiCl. (c) 2 mmol (4 eq.) of NaTMP and *i*Pr_3_SiCl, 1.5 mmol of PMDETA. (d) 2.5 mmol (5 eq.) of NaTMP and *i*Pr_3_SiCl, 2 mmol of PMDETA.

Next we then mapped the scope of the multisilylation process (Figure 6), initially testing substrates with different benzylic positions. Compounds **4b** and **4c** were obtained from disilylation of *p*‐xylene and 2,6‐lutidine (97% and 81% respectively), while trisilylation of 1,3,5‐mesitylene and tri(*p*‐tolyl)phosphine afforded **4d** and **4e** (96% and 81% respectively). In all cases, excellent yields were obtained with no traces of *gem*‐silylated products. Moreover, 3,3’,5,5’‐tetramethylbiphenyl was selectively silylated in the four methyl groups with near‐quantitative conversion (93% yield), highlighting the efficiency of the sequential sodiation–silylation steps and the complete suppression of the side decomposition process when using the bulkier silane coupling partner.

Moving towards the multisilylation of C(*sp* ^2^)–H bonds, derivatives **4g–j** were isolated in excellent yields (88%–98% yields), including 2,5‐disilylation of thiophene and selective disilylation of ferrocene on opposite cyclopentadienyl rings. Anisole could also be selectively disilylated at its *ortho*‐ and *meta*‐positions. This regioselectivity suggests that after *ortho*‐silylation, the presence of the Si*i*Pr_3_ group drives the second sodiation toward the *meta*‐position. The remote directing effect of bulky silyl groups has been previously noticed by Schlosser [42] and Knochel [43] for lithiation of activated arenes having F or Cl groups in combination with SiR_3_ substituents. Mulvey has reported the remarkable di‐magnesiation of anisole at its *ortho*‐ and *meta‐* positions, although in this case the selectivity is templated by the supramolecular constitution of the mixed Na/Mg base employed and high temperatures are required (100°C, 12 h) [44]. Demonstrating the strong metalating power of this Na/Si combination, non‐substituted naphthalene underwent selective disilylation at the 2,6‐positions (**4l**), whereas tetra‐fused pyrene ring system rendered exclusively its 2,7‐disilylated isomer (**4m**). The structure of **4m** was determined by ^1^H NMR and X‐ray crystallographic analysis (Figure 6). Overall, multifunctionalization seemed to occur minimizing steric repulsions between the silyl substituents since no other regioisomers were detected when analyzing and purifying the crude reaction mixtures. This is particularly noteworthy as selective metalation of non‐activated arenes is challenging and using super‐basic combinations such as the Lochman–Schlosser superbase led to the formation of a complex mixture of regioisomers from both mono‐ and dimetalation [16]. To the best of our knowledge, the case of pyrene represents the first example of selective main group dimetalation of this substrate. Orthogonal multisilylation of several (hetero)arenes was also investigated. In addition to 4‐methylanisole, products **4o**, **4p,** and **4q** were synthesized from 5‐methylbenzothiophene, benzofuran and 4‐ethynyltoluene (97%, 74%, and 83% yields respectively), showcasing the ability to regioselectively functionalize chemically distinct C─H bonds within a single substrate.

**FIGURE 7:**
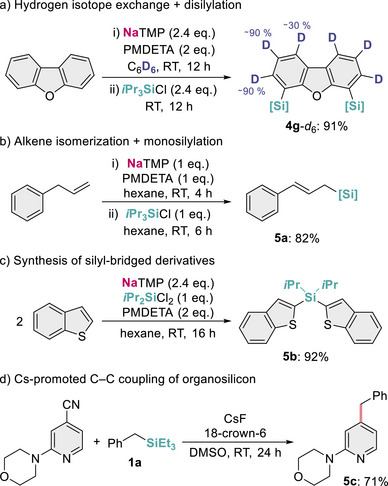
Additional NaTMP‐mediated silylation and late‐functionalization experiments. (a) Arene perdeuteration + C(*sp* ^2^)–H disilylation. (b) Alkene isomerization + C(*sp* ^3^)–H allylic silylation. (c) Synthesis of Si‐bridged derivatives. (d) C–C coupling of an organosilicon with a cyanopyridine.

This new sodium‐mediated silylation protocol could also be integrated with other applications that we have previously reported for NaTMP [28, 29]. The reaction of dibenzofuran with 2.4 eq. of NaTMP in the presence of PMDETA for 12 h in C_6_D_6_ (0.5 mL) followed by addition of 2.4 eq. of *i*Pr_3_SiCl furnished disilylation product **4g**‐*d*_6_ in a 91% isolated yield (Figure 7a), which has also undergone perdeuteration. The efficiency of the deuterium incorporation was confirmed by NMR and high‐resolution mass spectrometry, showing excellent isotopic labeling at the C3 and C4 atoms (∼90%) and partial deuteration in the most hindered C5‐positions (∼30%). Similarly, reacting allylbenzene with equimolar amounts of NaTMP and PMDETA for 4 h, followed by the addition of 1 eq. of *i*Pr_3_SiCl, allows for the isomerization of the double bond to form the internal alkene, which can subsequently be trapped by the silicon electrophile rendering **5a** in an 82% yield (Figure 7b). The reaction proved highly selective for the *E* isomer, and no silylation at the original benzylic position was observed, indicating complete isomerization before silylation.

Broadening even more the synthetic utility of this NaTMP‐mediated protocol, we performed two additional experiments. Replacing the chlorosilane *i*Pr_3_SiCl with *i*Pr_2_SiCl_2_ unlocked the synthesis of silyl‐bridged derivatives. This electrophile resisted lateral decomposition by NaTMP and efficiently furnished **5b** in nearly quantitative yield (Figure 7c). Furthermore, inspired by recent work by Bandar [45], we showed that **1a** can undergo C–C coupling with a cyanopyridine using CsF and 18‐crown‐6 in DMSO to furnish modified benzylpyridine **5c** in a 71% yield (Figure 7d), illustrating the role of organosilicon compounds in late‐stage functionalization of relevant organic scaffolds.

## 3 Conclusions

In summary, we have developed an efficient sodium‐mediated protocol for the regioselective deprotonative C(*sp* ^3^)/C(*sp* ^2^)–H mono‐ and multisilylation of a broad range of (hetero)arenes using commercially available chlorosilanes under mild reaction conditions. Control experiments have demonstrated the pivotal role of NaTMP, revealing a pronounced alkali‐metal effect in which sodium outperforms its nearest neighbors, potassium and lithium. Combined experimental and computational studies, including the isolation of key organometallic intermediates and extensive DFT investigations of both lateral decomposition and sodiation–silylation pathways, unveiled the cooperative interplay between NaTMP and the chlorosilane and underscored the decisive influence of steric factors on reactivity and selectivity. Furthermore, the protocol can be seamlessly integrated with NaTMP‐catalyzed hydrogen isotope exchange and alkene isomerization reactions, thereby expanding its synthetic utility. Collectively, these findings represent a significant advance in main group‐mediated C–H functionalization and deepen the understanding of sodium‐based reactivity in organic synthesis. This study is particularly promising from a sustainability perspective, given the prospect of limited lithium reagent supplies in the future due to the rapid increase in lithium usage in energy storage technologies.

## Conflicts of Interest

The authors declare no conflicts of interest.

## Supporting information




**Supporting File 1**: anie72077‐sup‐0001‐SuppMat.pdf.

**Supporting File 2**: anie72077‐sup‐0002‐Data.zip.

## Data Availability

The data that support the findings of this study are openly available in Boris at https://boris‐portal.unibe.ch/entities/product/fb8e87db‐336b‐4ad1‐a226‐13988c68f46f.
