# Supplementary material for: Deprotonative C(sp 3)/C(sp 2)–H (Multi)Silylation of (Hetero)Arenes Mediated by NaTMP
Source: Angew Chem Int Ed Engl. 2026 Apr 9;65(21):e1774364. doi: 10.1002/anie.1774364 (PMC13182195; doi:10.1002/anie.1774364)
Supplement: Supplementary file 1 — Supporting File 1: anie72077‐sup‐0001‐SuppMat.pdf. [file ANIE-65-e1774364-s001.pdf]

Supporting Information

for

Deprotonative C(sp^3)/C(sp^2)–H (Multi)Silylation of (Hetero)Arenes Mediated by NaTMP

David Sánchez-Roa, Sophia Belrhomari, Ana McGinley, Clevin Anto Liju, Manting Mu, Max García-Melchor and Eva Hevia

Table of Contents

General Methods 3
Starting Materials Preparation 4
Silylation of Toluene: Reaction Optimization 6
Synthesis of Organometallic Complexes 8
Synthesis of Silylated Compounds 19
General Procedure A: Monosilylation 19
Spectroscopic Data for Compounds 1a-j, 2a-g and 3a-n 20
Synthesis of *gem*-disilane 78
General Procedure B: Multisilylation 80
Spectroscopic Data for Compounds 4b-q 81
Regiodivergent Monosilylation of 4-Methylanisole 114
Combined Hydrogen Isotope Exchange and Silylation Procedure 116
Combined Alkene Isomerization and Silylation Procedure 118
Synthesis of Silyl-Bridged Derivative 120
C–C Coupling of Organosilicon Reagent 122
Decomposition of Fluoroarenes 124
NMR Monitoring Experiments 126
C(sp^3)–H silylation of 2-methylnaphthalene 126
C(sp^3)–H silylation of benzyltriethylsilane (1a) 128
Decomposition studies of trialkylchlorosilanes R_3SiCl with NaTMP 130
^{1}H DOSY NMR Experiments 134
X-Ray Crystallographic Details 137
Computational Methods 144
References 162

General Methods

All procedures were conducted using standard Schlenk line and glove box techniques under an inert atmosphere of argon. Hexane was degassed, purified and collected via an MBraun SPS 5 and stored over 4 Å molecular sieves for at least 24 hours prior to use. Deuterated solvents (C_6D_6 and C_6D_{12}) were purchased from VWR/Euroisotope/Merck, dried over NaK alloy for 16 hours and then cycled through three rounds of degassing by employing a freeze-pump-thaw method. The deuterated solvents were then collected via vacuum transfer and stored under argon atmosphere over 4 Å molecular sieves. Unless otherwise specified, all substrates employed are commercially available and were used as received (solids) or degassed by freeze-pump-thaw and stored over molecular sieves (liquids). The polydentate amines used in this study were dried over calcium hydride, distilled under reduced pressure and stored over molecular sieves prior to their use.

NMR spectra were recorded on Bruker spectrometers operating at 300 MHz. ^{1}H NMR spectra: 300.1 MHz, ^{13}C NMR spectra: 75.5 MHz, ^{19}F NMR spectra: 282 MHz, ^{31}P NMR spectra: 121 MHz. Spectra were analyzed using MestReNova software and referenced internally to the corresponding residual protium solvent peaks. Diffusion Ordered Spectroscopy (DOSY) experiments were conducted by NMR using the External Calibration Curve (ECC) method at 50 mM in C_6D_{12} as described by Stalke.[1] Data was accumulated by linearly varying the diffusion encoding gradients over a range of 2% to 95% for 32 gradient values. The signal decay dimension on the pseudo-2D data was generated by Fourier transformation of the time-domain data. The diffusion profile and coefficients were ascertained by use of the DOSY processing features of TopSpin software. For the determination of the deuterium incorporation by ^{1}H NMR spectroscopy, experiments were done with a single scan and T1=120s to ensure a reliable integration.

Elemental analyses (C, H and N) were conducted with a Flash 2000 Organic Elemental Analyser (Thermo Scientific). Samples were prepared in the glovebox under argon atmosphere and sealed in an air-tight container prior to analyses. All results were obtained by the Analytical Research and Services (Schürch Group) of the University of Bern. Samples were weighed on a Mettler Toledo balance with +2 µg resolution and sample weights from 1 - 3 mg were used. For calibration, cysteine was used as a reference material. The presented values are the average of determinations in triplicate to ensure consistency.

HRMS measurements were performed by the Analytical Research and Services (Schürch Group) of the University of Bern, using a ThermoScientific LTQ Orbitrap XL, equipped with a Nanoelectrospray Ion Source (NSI) or a ThermoScientific Q Exactive GC-MS. Deuterium incorporation was determined by comparison of the mass spectral patterns of the deuterated products versus the original starting material with isotopic natural abundance using the IsoPat2 spreadsheet.[2]

Starting Materials Preparation

*n*BuNa

Prepared according to a literature procedure.[3] In an oven-dried and argon-flushed Schlenk flask containing a Teflon-coated magnetic stirrer, sodium *tert*-butoxide (3.84 g, 40 mmol) was suspended in dry hexane (75 mL). The resulting white suspension was cooled to 0 °C in an ice bath and *n*BuLi (27 mL, 1.6 M, 44 mmol) was added dropwise. The ice bath was removed, and the thick white suspension was allowed to reach room temperature and stirred overnight. The next day, the suspension was filtered under argon through an oven-dried Schlenk-frit and the solid was washed with dry hexane (2 x 10 mL). The solid was then dried under vacuum and transferred to a vial for storage inside the glovebox freezer at – 30 ºC. *n*BuNa was obtained as a white solid (2.60 g, 80 % yield).

NaTMP

Prepared according to a literature procedure.[3] In an oven-dried and argon-flushed Schlenk flask containing a Teflon-coated magnetic stirrer, *n*BuNa (2.60 g, 32.5 mmol) was suspended in dry hexane (60 mL). The resulting white suspension was cooled to 0 °C in an ice bath and dry TMP(H) (5.5 mL, 32.5 mmol) was added dropwise. The ice bath was removed, and the thick white suspension was allowed to reach room temperature and stirred overnight. The next day, the suspension was filtered under argon through an oven-dried Schlenk-frit and the solid was washed with dry hexane (3 x 5 mL). The solid was then dried under vacuum and transferred to a vial for storage inside the glovebox freezer at – 30 ºC. NaTMP was obtained as a white solid (4.85 g, 92 % yield).

LiTMP

Prepared according to a literature procedure.[4] In an oven-dried and argon-flushed Schlenk flask containing a Teflon-coated magnetic stirrer, TMP(H) (1.6 mL, 10 mmol) was dissolved in dry hexane (50 mL). The resulting solution was cooled to 0 °C in an ice bath and *n*BuLi (6.3 mL, 1.6 M, 10 mmol) was added dropwise. The ice bath was removed, and the pale-yellow suspension was allowed to reach room temperature and stirred for an additional hour. The reaction mixture was then concentrated under reduced pressure to encourage further precipitation of LiTMP and then stored in a – 30 °C freezer overnight. The solid was separated from the solution by cannula filtration and dried under vacuum. Due to the considerable solubility of LiTMP even in cold hexane, the precipitate was not subjected to any additional washes and was used without further purification.

KTMP

Prepared according to a literature procedure.[5] In an oven-dried and argon-flushed Schlenk flask containing a Teflon-coated magnetic stirrer, KCH_2SiMe_3 (1.26 g, 10 mmol) was suspended in dry hexane (40 mL). The resulting white suspension was cooled to 0 °C in an ice bath and dry TMP(H) (1.69 mL, 10 mmol) was added dropwise. The ice bath was removed,

and the yellowish suspension was allowed to reach room temperature and stirred overnight. The next day, the suspension was filtered under argon through an oven-dried Schlenk-frit and the solid was washed with dry hexane (3 x 10 mL). The solid was then dried under vacuum and transferred to a vial for storage inside the glovebox freezer at – 30 ºC. KTMP was obtained as a light-brown solid (1.51 g, 50 % yield).

$NaCH_2SiMe_3$

Prepared according to a literature procedure.[5] In an oven-dried and argon-flushed Schlenk flask containing a Teflon-coated magnetic stirrer, NaO*t*Bu (2.88 g, 30 mmol) was suspended in 50 mL of dry hexane. The resulting white suspension was cooled to 0 °C in an ice bath and $LiCH_2SiMe_3$ (2.82 mL, 30 mmol) was added dropwise. The ice bath was removed, and the fine white suspension was allowed to reach room temperature and stirred overnight. The next day, the suspension was filtered under argon through an oven-dried Schlenk-frit and the solid was washed with dry hexane (3 x 15 mL). The solid was then dried under vacuum and transferred to a vial for storage inside the glovebox. $NaCH_2SiMe_3$ was obtained as a white solid (2.94 g, 89 % yield).

***N*-*Tert*-butyl-1-(1-methyl-1*H*-indol-3-yl)methanimine**

In an oven-dried and argon-flushed Schlenk flask containing a Teflon-coated magnetic stirrer and molecular sieves, 1-methylindole-3-carboxaldehyde (0.5 g, 3.14 mmol) and *tert*-butylamine (1.65 mL, 15.7 mmol) were dissolved in 5 mL of dry dichloromethane, and the resulting solution was stirred overnight at room temperature. The next day, the solution was filtered under air to remove the molecular sieves and dried under reduced pressure. *N*-*Tert*-butyl-1-(1-methyl-1H-indol-3-yl)methanimine was obtained as an off-white solid (473 mg, 70 % yield).

Silylation of Toluene: Reaction Optimization

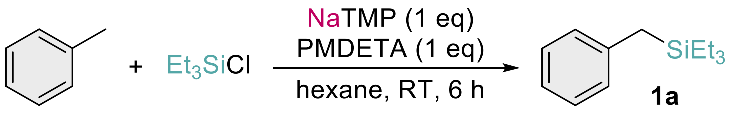

Table S1. Optimization studies for the C(sp^3)–H silylation of toluene.

Entry	Deviation from standard conditions	NMR yield[b] [%]
1	None	75
2	***n*BuLi** instead of NaTMP	0
3	**LiTMP** instead of NaTMP	18
4	**KTMP** instead of NaTMP	62
5	**NaHMDS** instead of NaTMP	0
6	**$NaCH_2SiMe_3$** instead of NaTMP	0
7	**TMEDA** instead of PMDETA	54
8	**10 mol%** of PMDETA	84
9	**Me_3SiCl** instead of Et_3SiCl	0
10	***i*Pr_3SiCl** instead of Et_3SiCl	90
11	***n*BuLi·TMEDA** instead of NaTMP·PMDETA	0
12	**LiTMP + NaO*t*Bu** instead of NaTMP	62
13	***n*BuLi/NaO*t*Bu/TMP(H)** instead of NaTMP	69
14	***n*BuLi/KO*t*Bu/TMP(H)** instead of NaTMP	71
15	***n*BuLi/NaO*t*Bu/TMP(H)** instead of NaTMP (**10 mol%** of PMDETA)	72
16	***n*BuLi/KO*t*Bu/TMP(H)** instead of NaTMP (**10 mol%** of PMDETA)	74

In an oven-dried and argon-flushed Schlenk flask containing a Teflon-coated magnetic stirrer, the base (0.5 mmol) was suspended in 3 mL of dry hexane. The resulting white suspension was stirred and the chlorosilane (0.5 mmol) was added, followed by toluene (0.5 mmol) and the donor (0.5 mmol; 0.05 mmol for entries 8, 15 and 16). The mixture was then stirred at room temperature for 6 h. Afterwards, the reaction was quenched with a saturated solution of NH_4Cl (10 mL) and extracted with Et_2O (3 x 10 mL). The organic phase was dried over Na_2SO_4, filtered and dried under reduced pressure. NMR yield was measured by adding 1,1,2,2-tetrachloroethane (26.4 µL, 0.25 mmol) and comparing the corresponding signal in the ^{1}H NMR spectrum. An example corresponding to entry 7 is shown below.

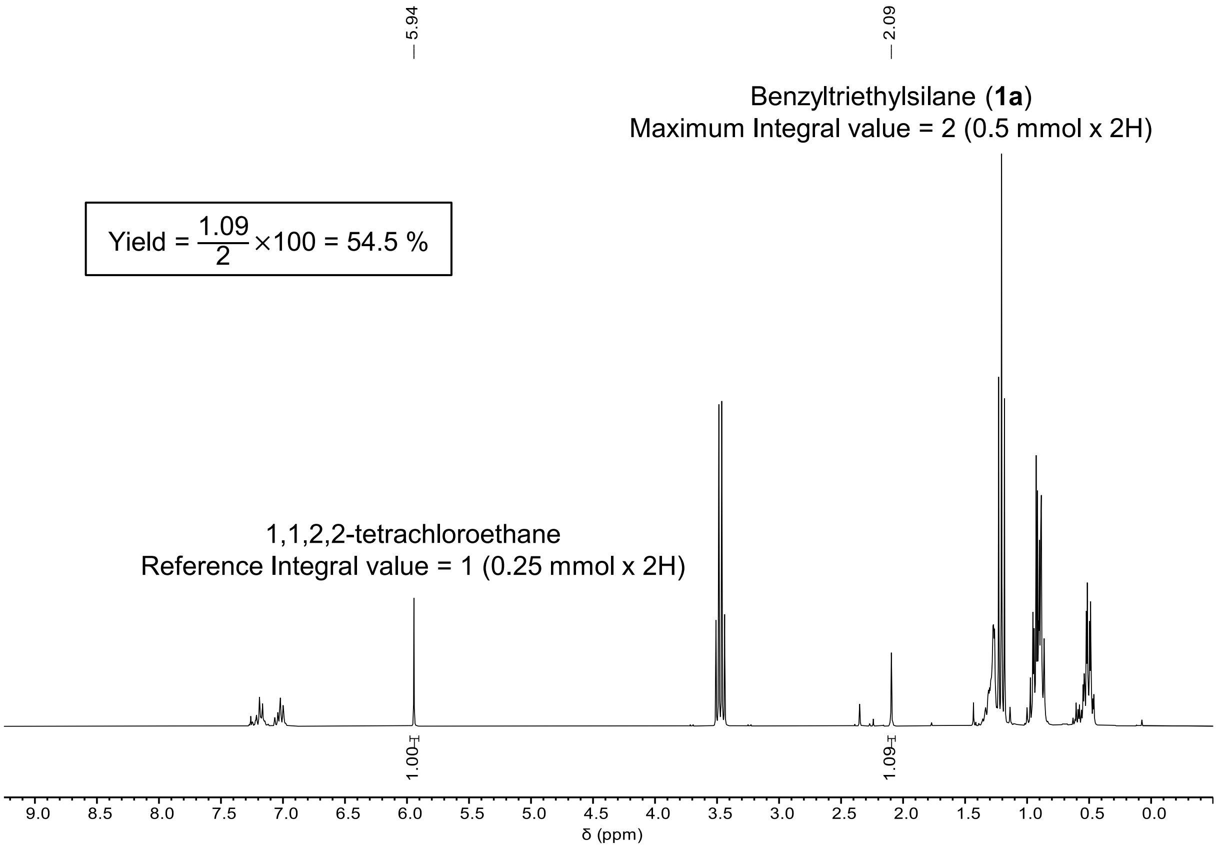

Figure S1. ^{1}H NMR spectrum of the optimization studies for the C(sp^3)–H silylation of toluene (Table S1, entry 7).

Synthesis of Organometallic Complexes

{(PMDETA)Na(2-CH_2Naph)} (I)

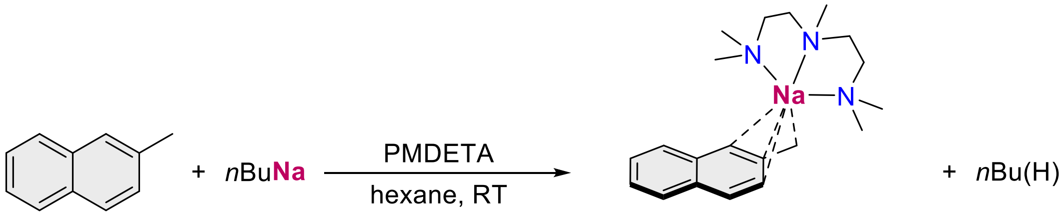

{(PMDETA)Na(2-CH_2Naph)}
(**I**)

In an argon-filled glovebox, *n*BuNa (16.0 mg, 0.2 mmol) was added to an oven-dried 7 mL glass vial and suspended in 2 mL of dry hexane. Then, 2-methylnaphthalene (28.4 mg, 0.2 mmol) was added followed by PMDETA (41.6 µL, 0.2 mmol), leading to the immediate formation of a deep purple solution that was stirred and allowed to react for 20 min. The mixture was passed through a syringe filter to remove the dark purple solid material precipitated (non-crystalline desired product mostly) and then stored in the glovebox freezer at -30 ºC. After 24 h, purple crystals were grown, which were washed with cold pentane (3 x 2 mL) to afford {(PMDETA)Na(2-CH_2Naph)} (**I**) as a dark purple crystalline solid in 31% yield (20.8 mg).

Alternatively, NaTMP can be used as a base to deprotonate the substrate, but the reaction is equilibrium-controlled and releases TMP(H), which reduces the quantity of the crystalline material and complicates its isolation.

^{1}H NMR (300 MHz, C_6D_6): δ 7.22 (d, *J* = 7.8 Hz, 1H), 7.09 (ddd, *J* = 8.3, 6.7, 1.3 Hz, 1H), 6.95 (d, *J* = 8.9 Hz, 1H), 6.92 (d, *J* = 8.3 Hz, 1H), 6.85 (dd, *J* = 8.9, 2.1 Hz, 1H), 6.54 (ddd, *J* = 7.8, 6.5, 1.2 Hz, 1H), 6.10 (d, *J* = 2.1 Hz, 1H), 3.08 (br s, 1H), 3.04 (br s, 1H), 1.78 (s, 12H), 1.65 (s, 3H), 1.60 (br s, 8H).

^{13}C{^{1}H} NMR (75 MHz, C_6D_6): δ 153.1, 141.5, 128.8, 128.1 (overlaps with C_6D_6, see HSQC), 127.0, 125.4, 123.4, 120.2, 112.3, 94.7, 57.1, 54.2, 50.7, 45.2, 42.8.

Elemental Analysis: Calculated for $C_{20}H_{32}N_3Na$: C: 71.18, H: 9.56, N: 12.45. Found: C: 71.06, H: 9.32, N: 11.93.

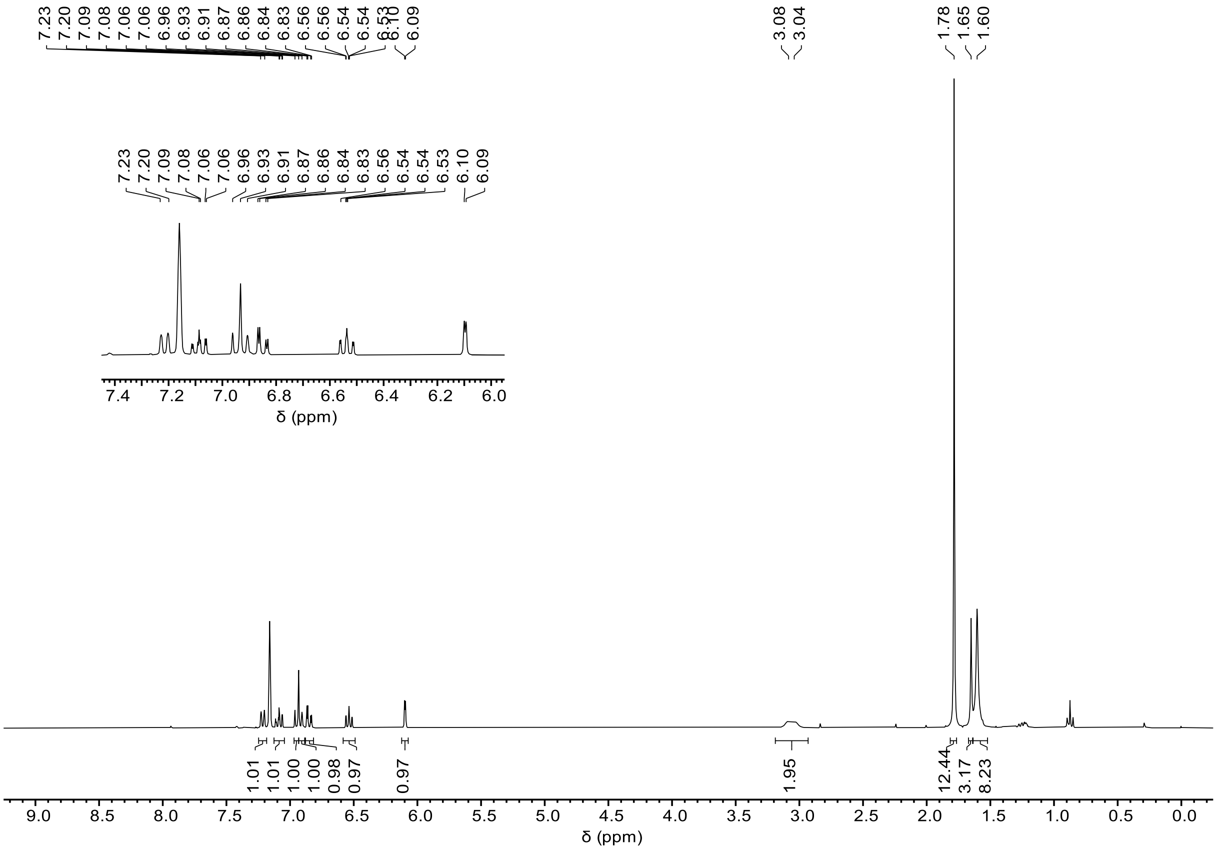

Figure S2. ^1H NMR of {(PMDETA)Na(2-CH_2Naph)} (**I**) in C_6D_6.

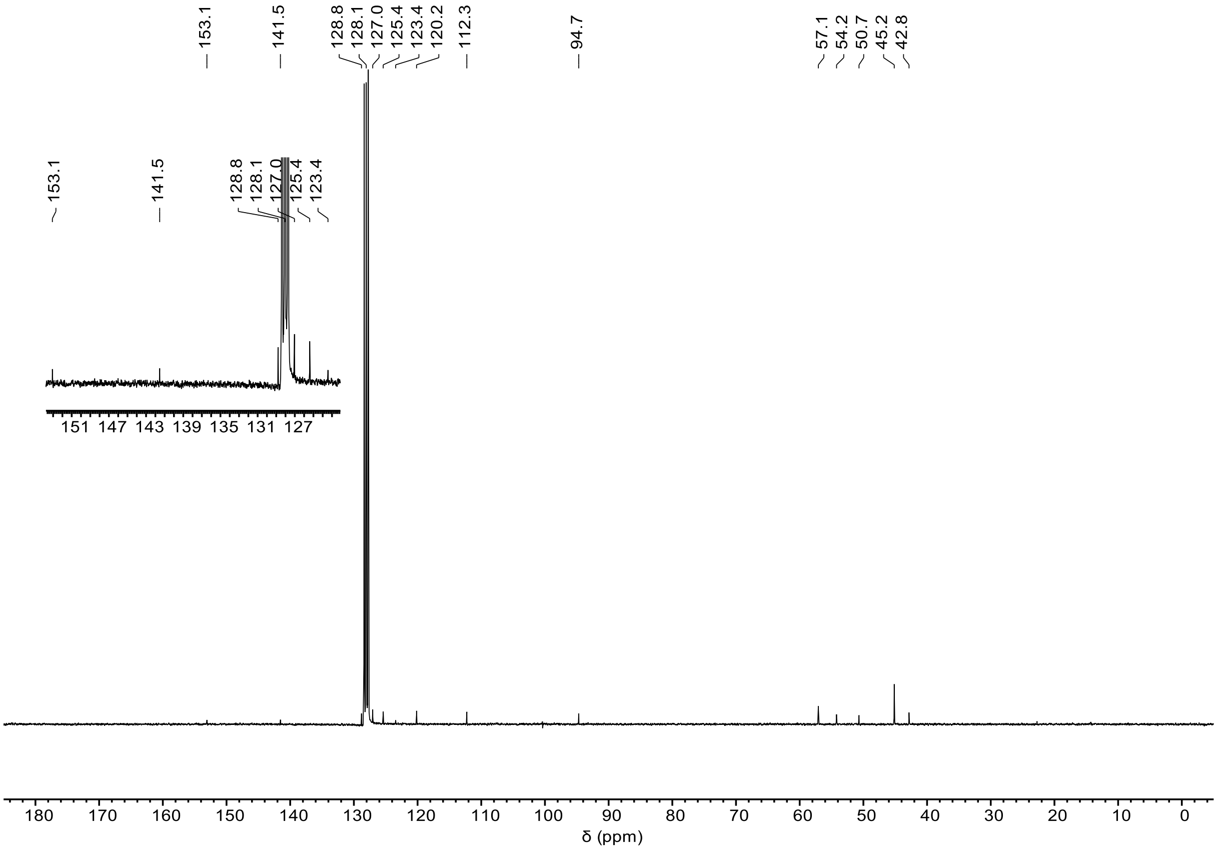

Figure S3. $^{13}C\{^1H\}$ NMR of {(PMDETA)Na(2-CH_2Naph)} (**I**) in C_6D_6.

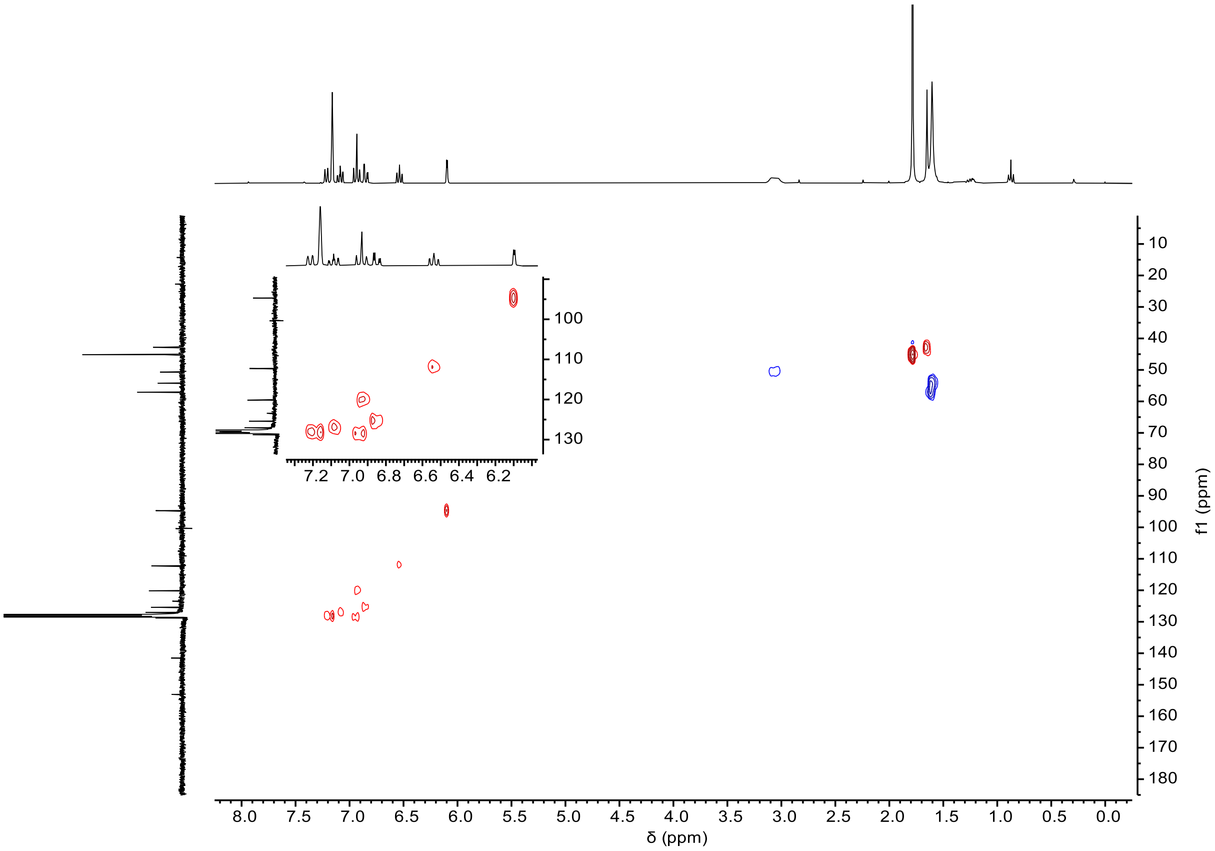

Figure S4. ^1H–^{13}C HSQC NMR of {(PMDETA)Na(2-CH_2Naph)} (**I**) in C_6D_6.

{(PMDETA)Na_2[(C_5H_4)Fe(C_5H_5)](TMP)}$_2$ (II)

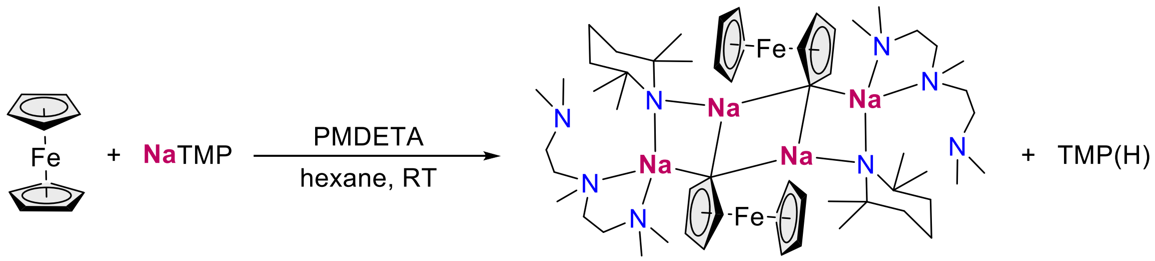

{(PMDETA)Na_2[(C_5H_4)Fe(C_5H_5)](TMP)}$_2$
(**II**)

In an argon-filled glovebox, NaTMP (65.2 mg, 0.4 mmol) was added to an oven-dried 7 mL glass vial and suspended in 2 mL of dry hexane. Then, ferrocene (37.2 mg, 0.2 mmol) was added followed by PMDETA (41.6 µL, 0.2 mmol), leading to the immediate formation of a red solution that was stirred and allowed to react for 20 min. The mixture was passed through a syringe filter to remove the red solid material precipitated and then stored in the glovebox freezer at -30 ºC. After 24 h, red crystals were grown, which were washed with cold pentane (3 x 2 mL) to afford {(PMDETA)Na_2[(C_5H_4)Fe(C_5H_5)](TMP)}$_2$ (**II**) as a red crystalline solid in 67% yield (73 mg). Complete characterization of the compound was not possible due to its high insolubility and reactivity.

Elemental Analysis: Calculated for $C_{56}H_{100}Fe_2N_8Na_4$: C: 61.75, H: 10.26, N: 9.26. Found: C: 62.10, H: 10.56, N: 9.62.

{(PMDETA)Na(1-Me-2-indolyl)}$_2$ (III)

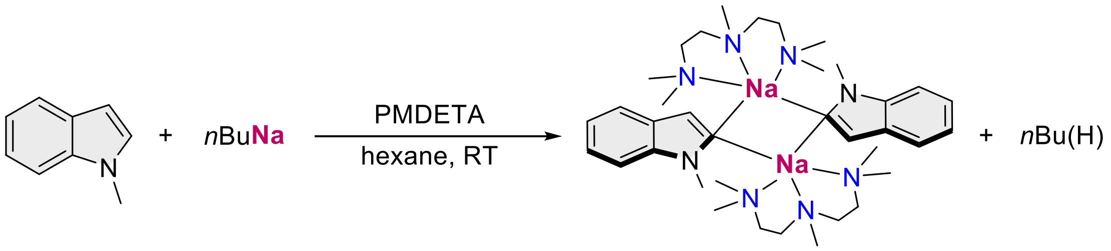

{(PMDETA)Na(1-Me-2-indolyl)}$_2$
(**III**)

In an argon-filled glovebox, *n*BuNa (16.0 mg, 0.2 mmol) was added to an oven-dried 7 mL glass vial and suspended in 2 mL of dry hexane. Then, 1-methylindole (25.0 μL, 0.2 mmol) was added followed by PMDETA (41.6 μL, 0.2 mmol), leading to the immediate formation of a yellow solution that was stirred and allowed to react for 20 min. The mixture was passed through a syringe filter to remove any remaining solid material and then stored in the glovebox freezer at -30 ºC. After 24 h, yellow crystals were grown, which were washed with cold pentane (3 x 2 mL) to afford {(PMDETA)Na(1-Me-2-indolyl)}$_2$ (**III**) as a yellow crystalline solid in 41% yield (33.4 mg).

Alternatively, NaTMP can be used as a base to deprotonate the substrate, but the reaction is equilibrium-controlled and releases TMP(H), which reduces the quantity of the crystalline material and complicates its isolation.

^{1}H NMR (300 MHz, C_6D_6): δ 7.87 – 7.80 (m, 1H), 7.44 (dt, J = 5.9, 1.5 Hz, 1H), 7.30 (td, J = 7.0, 1.5 Hz, 1H), 7.26 (td, J = 7.0, 1.5 Hz, 1H), 6.73 (d, J = 0.6 Hz, 1H), 4.00 (s, 3H), 1.93 (s, 12H), 1.86 – 1.79 (m, 4H), 1.79 – 1.72 (m, 4H), 1.30 (s, 3H).

^{13}C{^{1}H} NMR (75 MHz, C_6D_6): δ 197.0, 141.5, 133.7, 117.0, 116.9, 116.9, 111.5, 107.7, 57.4, 55.1, 45.4, 42.5, 38.1.

Elemental Analysis: Calculated for $C_{18}H_{31}N_4Na$: C: 66.22, H: 9.67, N: 17.16. Found: C: 66.60, H: 9.67, N: 17.07.

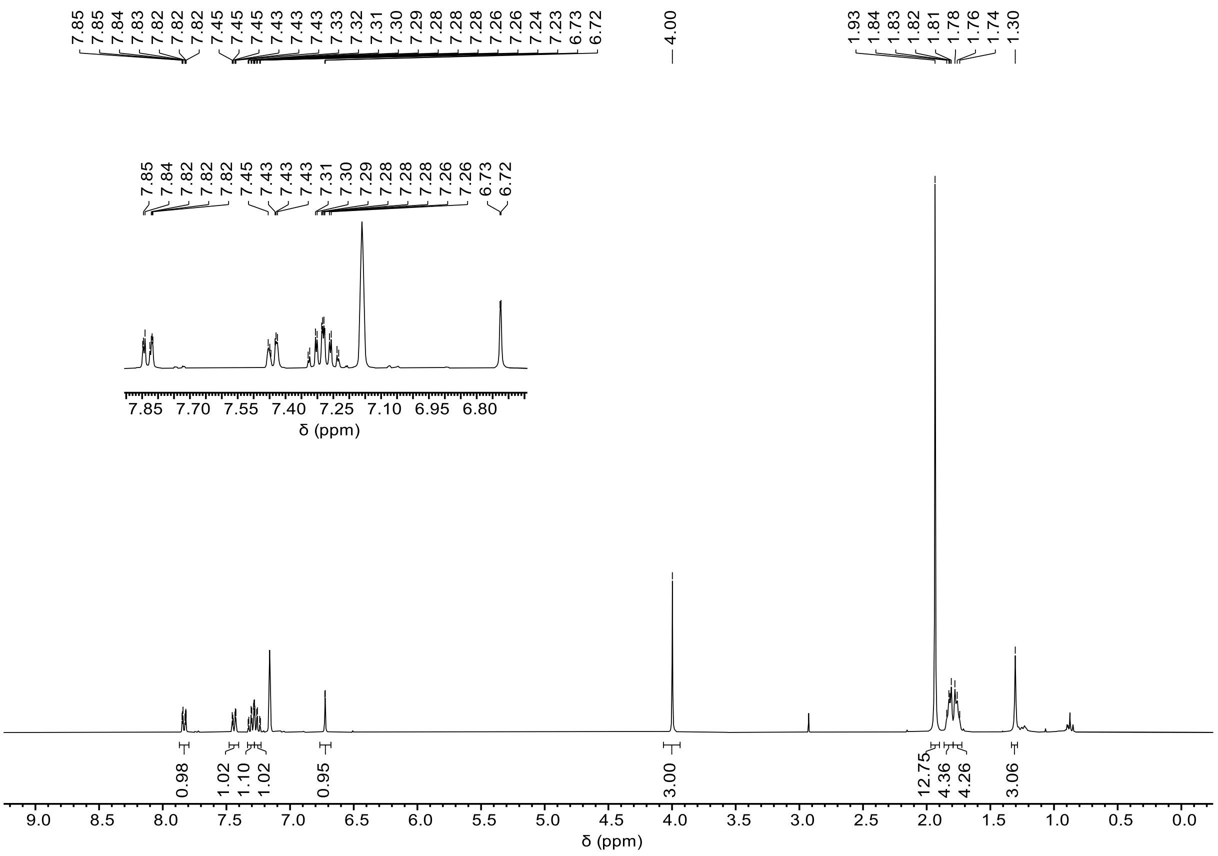

Figure S5. ^{1}H NMR of {(PMDETA)Na(1-Me-2-indolyl)}$_2$ (**III**) in C_6D_6.

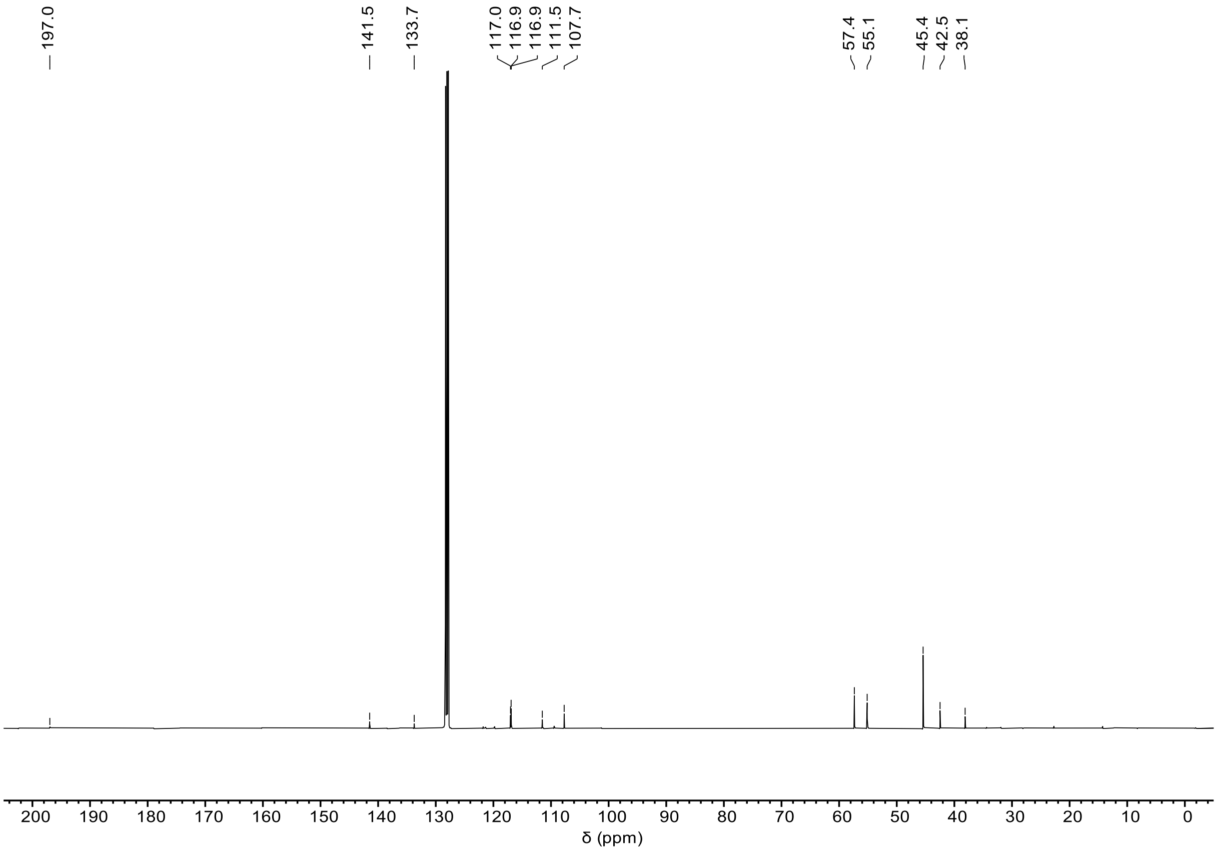

Figure S6. $^{13}C\{^{1}H\}$ NMR of {(PMDETA)Na(1-Me-2-indolyl)}$_2$ (**III**) in C_6D_6.

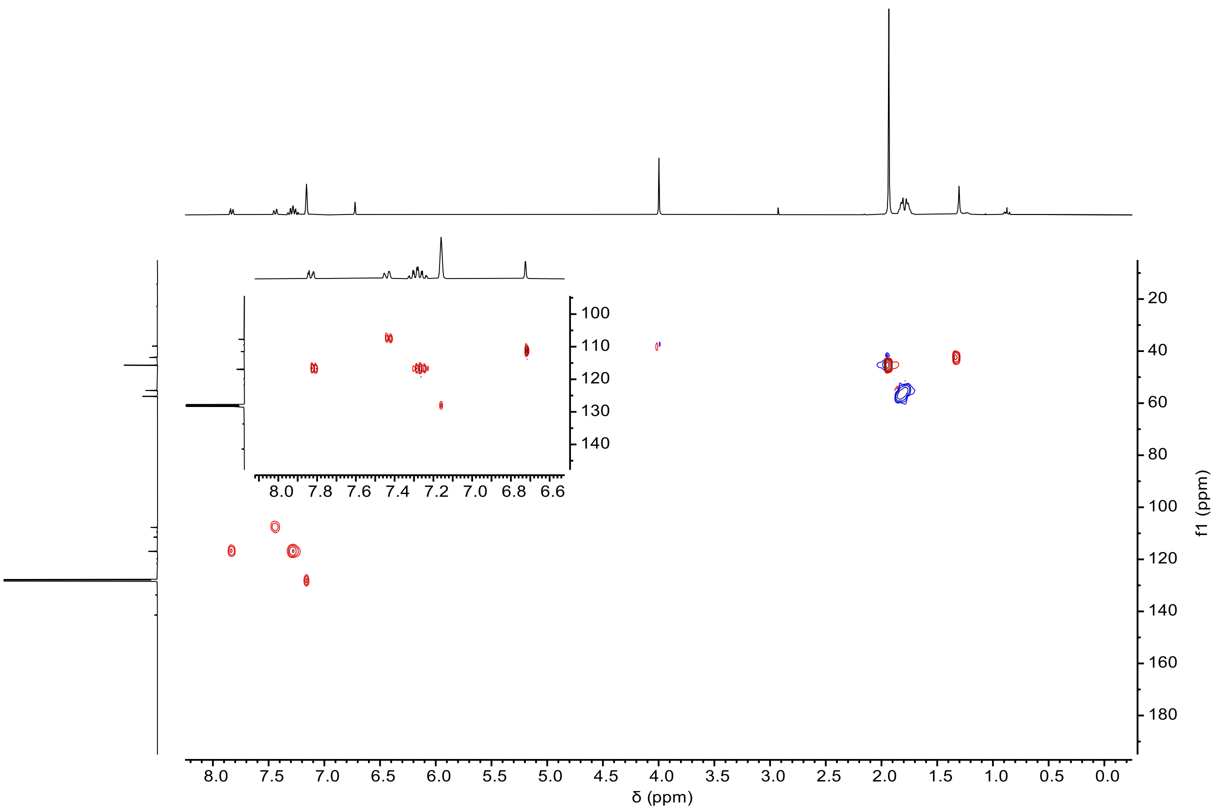

Figure S7. ^{1}H–^{13}C HSQC NMR of {(PMDETA)Na(1-Me-2-indolyl)}$_2$ (**III**) in C_6D_6.

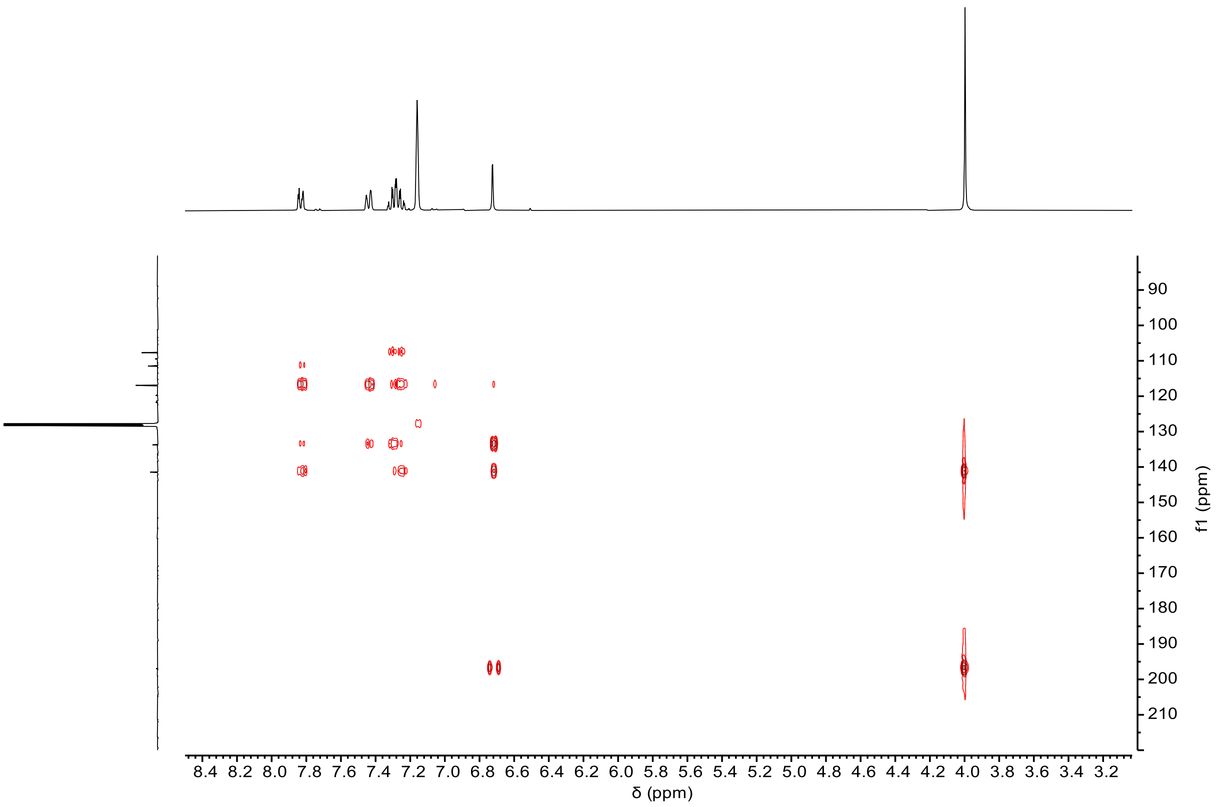

Figure S8. ^{1}H–^{13}C HMBC NMR of {(PMDETA)Na(1-Me-2-indolyl)}$_2$ (**III**) in C_6D_6.

{(PMDETA)Na($Et_3SiCHPh$)} (IV)

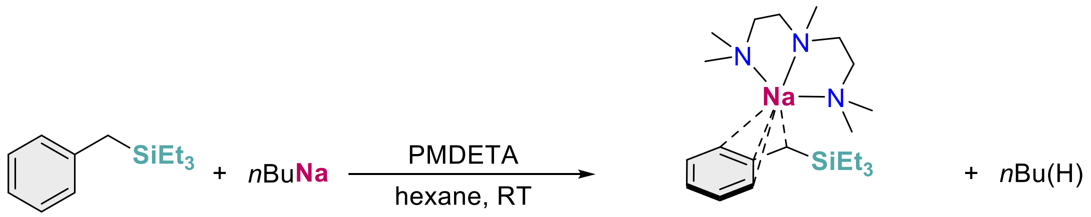

{(PMDETA)Na($Et_3SiCHPh$)}
(**IV**)

In an argon-filled glovebox, *n*BuNa (16.0 mg, 0.2 mmol) was added to an oven-dried 7 mL glass vial and suspended in 2 mL of dry hexane. Then, benzyltriethylsilane (**1a**) (41.3 mg, 0.2 mmol) was added followed by PMDETA (41.6 µL, 0.2 mmol), leading to the immediate formation of a yellow solution that was stirred and allowed to react for 20 min. The mixture was passed through a syringe filter to remove any remaining solid material and then stored in the glovebox freezer at -30 °C. After 24 h, yellow crystals were grown, which were washed with cold pentane (3 x 2 mL) to afford {(PMDETA)Na($Et_3SiCHPh$)} (**IV**) as a yellow crystalline solid in 74% yield (59.3 mg).

Alternatively, NaTMP can be used as a base to deprotonate the substrate, but the reaction is equilibrium-controlled and releases TMP(H), which reduces the quantity of the crystalline material and complicates its isolation.

^{1}H NMR (300 MHz, C_6D_{12}): δ 6.45 – 6.37 (m, 2H), 6.37 – 6.09 (br m, 2H), 5.54 (tt, *J* = 6.9, 1.2 Hz, 1H), 2.33 – 2.18 (m, 8H), 2.15 (s, 3H), 2.14 (s, 12H), 1.76 (s, 1H), 0.94 (t, *J* = 7.9 Hz, 9H), 0.51 (q, *J* = 7.9 Hz, 6H).

^{13}C{^{1}H} NMR (75 MHz, C_6D_{12}): δ 159.2, 129.9, 117.9, 106.0, 58.0, 55.0, 45.8, 44.2, 39.8, 9.2, 7.2.

Elemental Analysis: Calculated for $C_{22}H_{44}N_3NaSi$: C: 65.78, H: 11.04, N: 10.46. Found: C: 65.61, H: 11.43, N: 10.78.

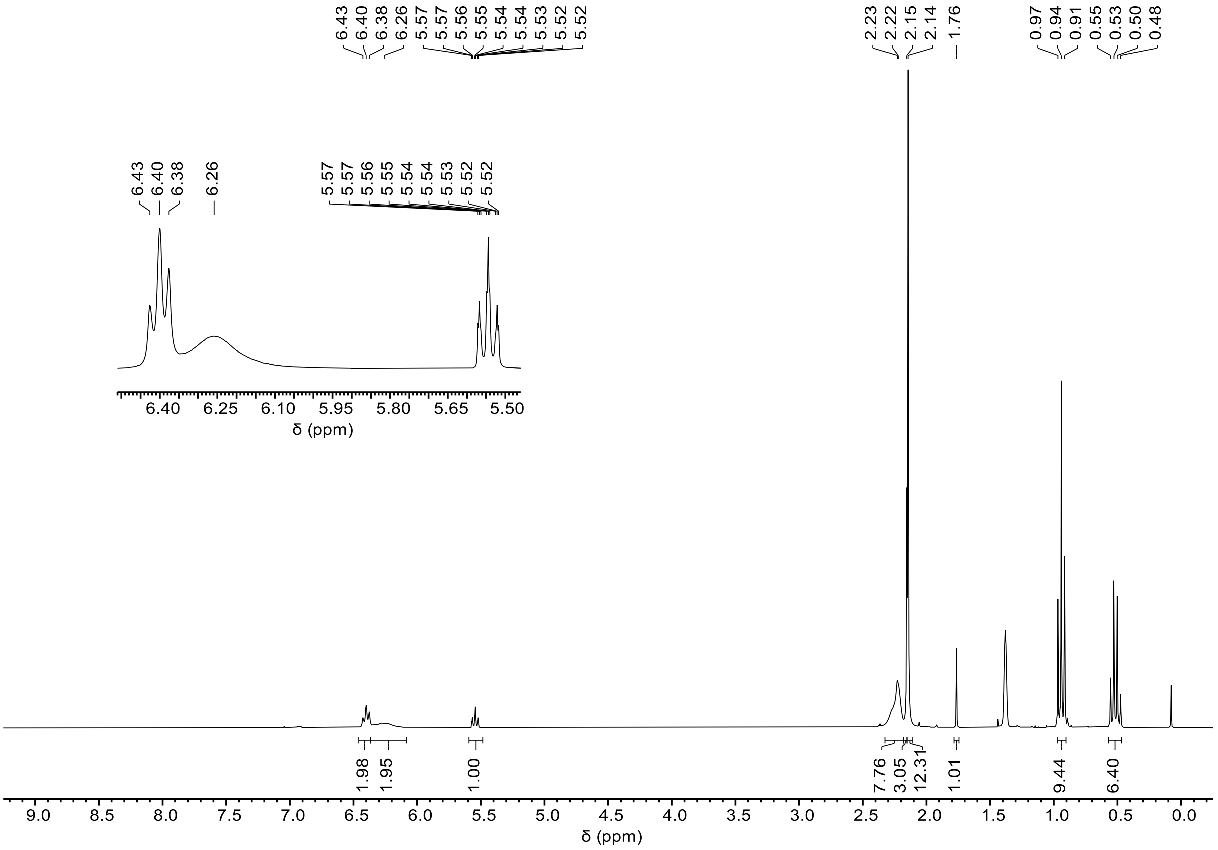

Figure S9. ^{1}H NMR of {(PMDETA)Na(Et_3SiCHPh)} (**IV**) in C_6D_{12}.

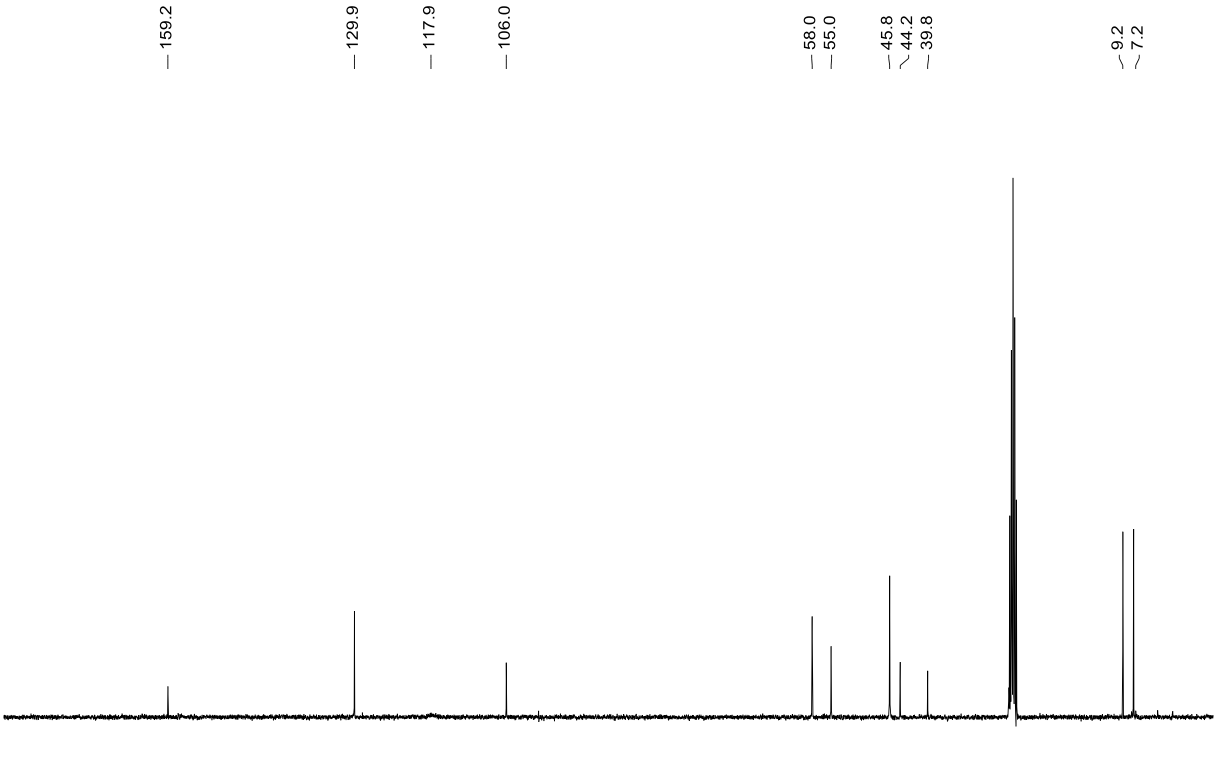

Figure S10. $^{13}C\{^{1}H\}$ NMR of {(PMDETA)Na(Et_3SiCHPh)} (**IV**) in C_6D_{12}.

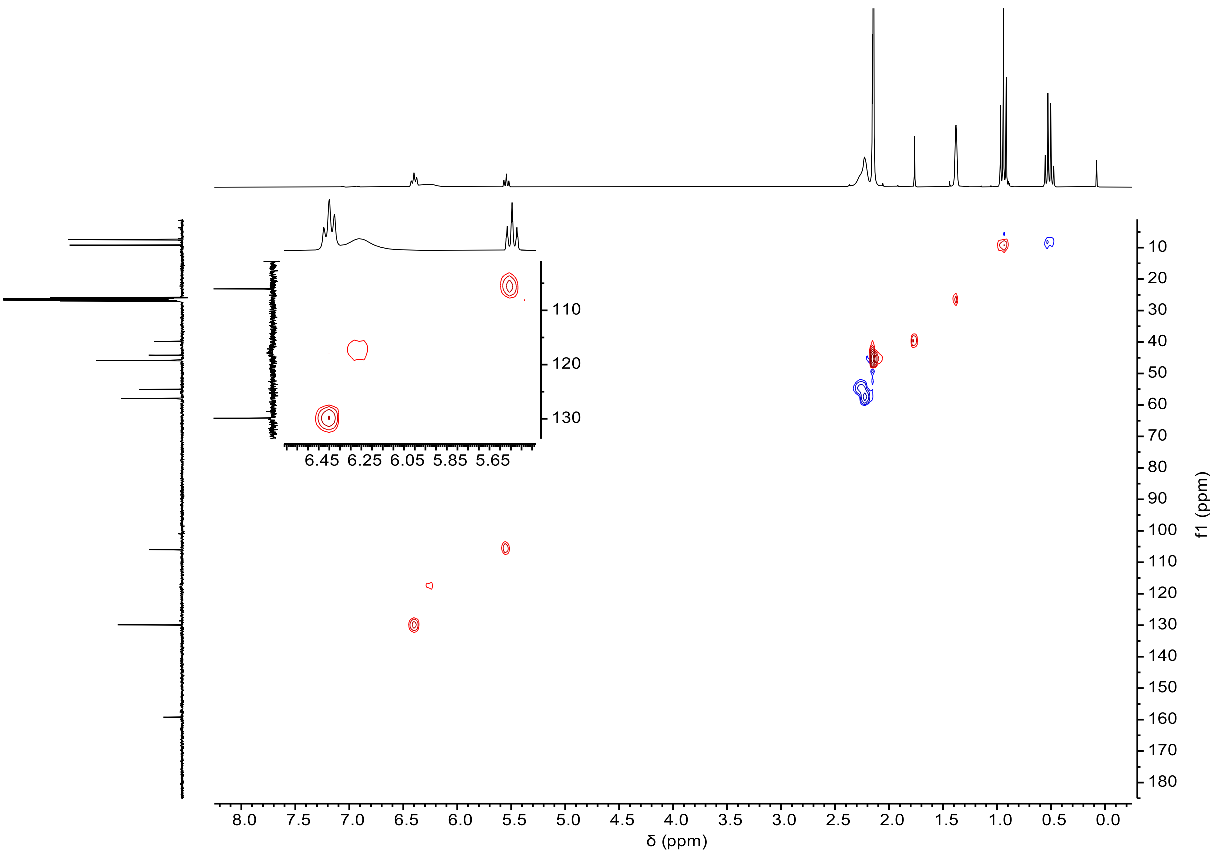

Figure S11. $^{1}H–^{13}C$ HSQC NMR of {(PMDETA)Na(Et$_3$SiCHPh)} (**IV**) in C_6D_{12}.

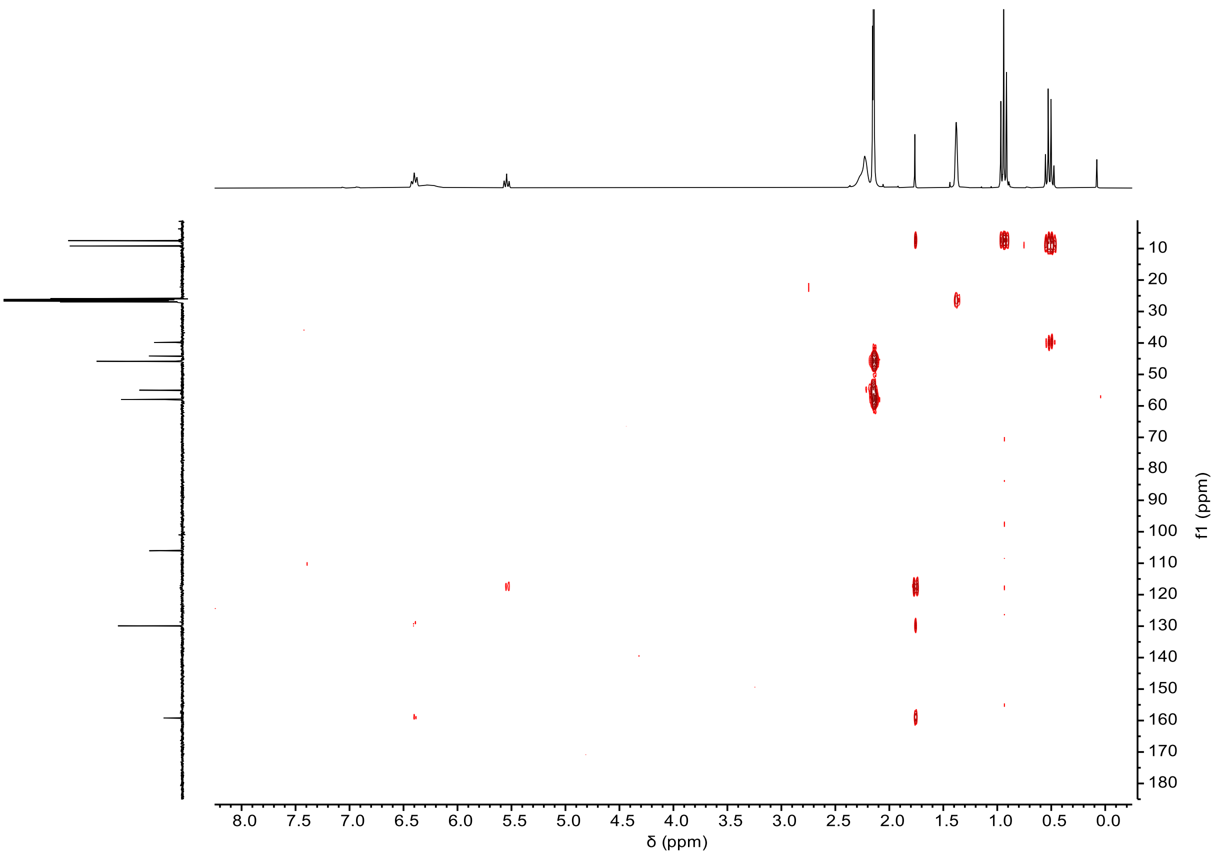

Figure S12. $^{1}H–^{13}C$ HMBC NMR of {(PMDETA)Na(Et$_3$SiCHPh)} (**IV**) in C_6D_{12}.

Synthesis of Silylated Compounds

General Procedure A: Monosilylation

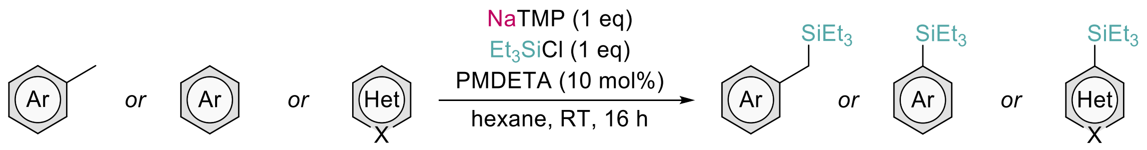

X = N, O, S **1** **2** **3**

In an oven-dried and argon-flushed Schlenk flask containing a Teflon-coated magnetic stirrer, NaTMP (81.6 mg, 0.5 mmol, 1 eq.) was suspended in 3 mL of dry hexane. The resulting white suspension was stirred and Et_3SiCl (83.9 µL, 0.5 mmol, 1 eq.) was added, followed by the desired substrate (0.5 mmol, 1 eq.) and PMDETA (10.4 µL, 0.05 mmol, 10 mol%). The mixture was then stirred at room temperature for 16 h. Afterwards, the reaction was quenched with a saturated solution of NH_4Cl (10 mL) and extracted with Et_2O (3 x 10 mL). The organic phase was dried over Na_2SO_4, filtered and dried under reduced pressure. NMR yield was measured by adding 1,1,2,2-tetrachloroethane (26.4 µL, 0.25 mmol) and comparing the corresponding signal in the ^1H NMR spectrum. Purification of the silylated compounds was performed by flash column chromatography. The details of the purification process and other possible deviations from the General Method are specified with each substrate.

Spectroscopic Data for Compounds 1a-j, 2a-g and 3a-n

Benzyltriethylsilane (1a)

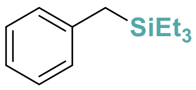

$C_{13}H_{22}Si$
Molecular Weight: 206.40

Prepared according to General Procedure A on a 6 mmol scale and purified by flash column chromatography in silica gel and hexane, compound **1a** was isolated as a colorless oil (1.17 g, 95 % yield). Spectroscopic data are in accordance with literature reports.[6]

^{1}H NMR (300 MHz, $CDCl_3$): *δ* 7.29 – 7.18 (m, 2H), 7.15 – 7.00 (m, 3H), 2.14 (s, 2H), 0.96 (t, *J* = 7.9 Hz, 9H), 0.55 (q, *J* = 7.9 Hz, 6H).

^{13}C{^{1}H} NMR (75 MHz, $CDCl_3$): *δ* 140.8, 128.3, 128.3, 123.9, 21.8, 7.4, 3.1.

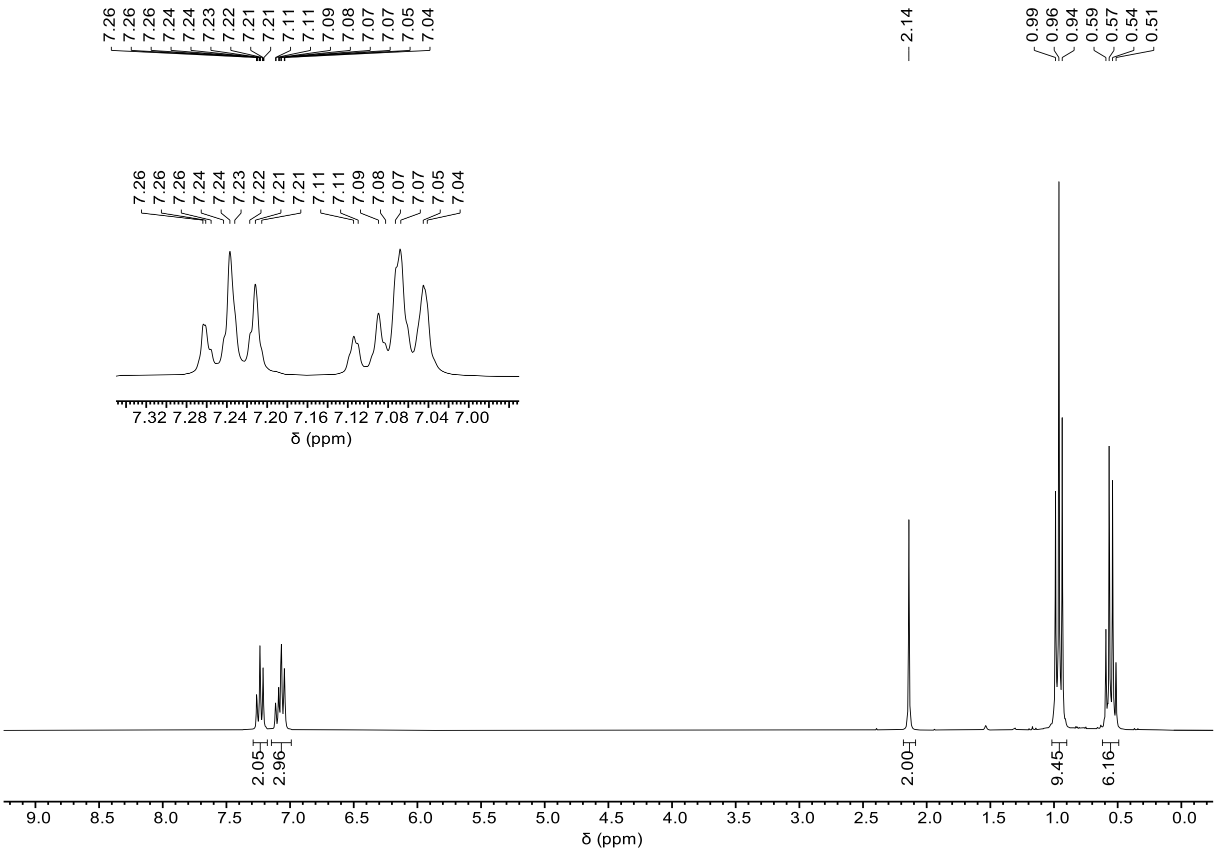

Figure S13. ^{1}H NMR of **1a** in $CDCl_3$.

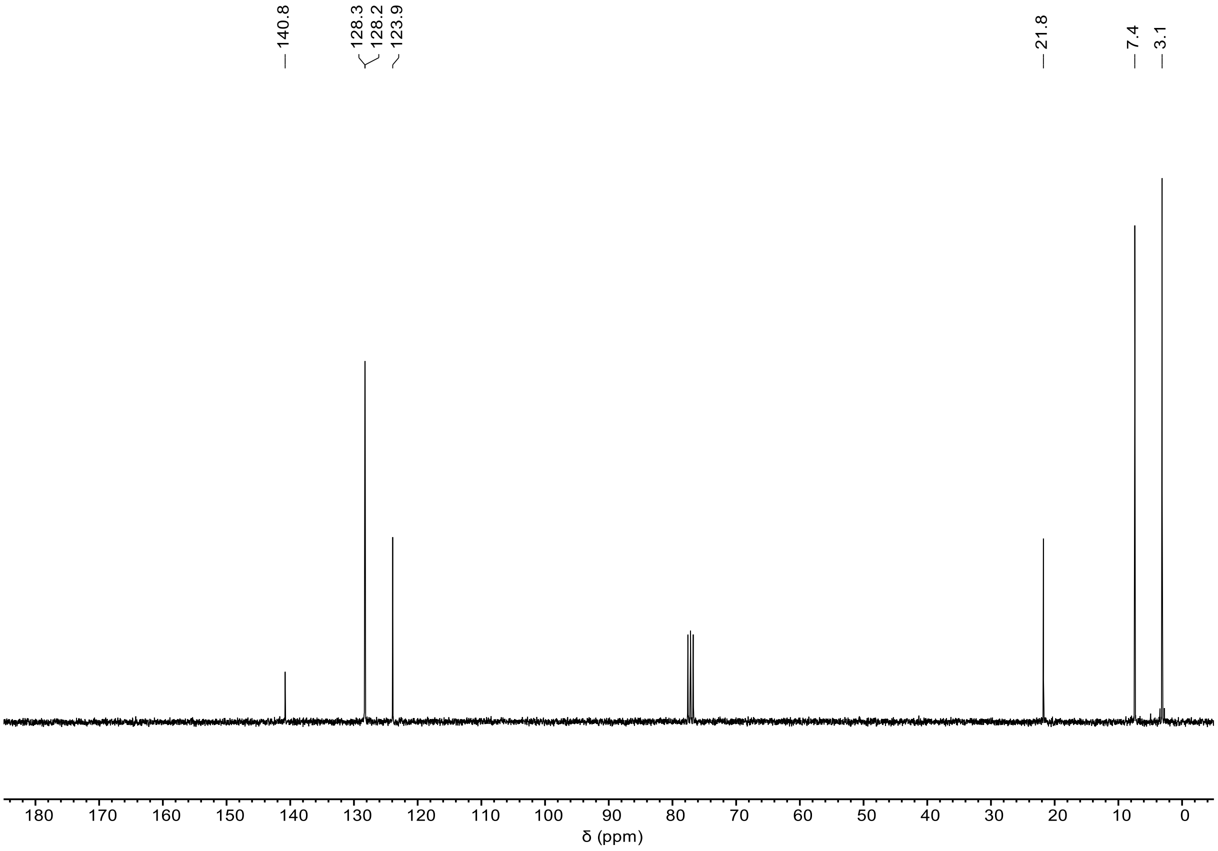

Figure S14. $^{13}C\{^1H\}$ NMR of **1a** in $CDCl_3$.

Benzhydryltriethylsilane (1b)

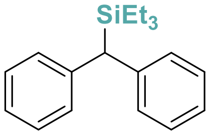

$C_{19}H_{26}Si$
Molecular Weight: 282.50

Prepared according to General Procedure A and purified by flash column chromatography in silica gel and hexane, compound **1b** was isolated as a colorless oil (112 mg, 79 % yield). Spectroscopic data are in accordance with literature reports.[7]

^{1}H NMR (300 MHz, $CDCl_3$): δ 7.29 – 7.19 (m, 8H), 7.15 – 7.07 (m, 2H), 3.65 (s, 1H), 0.85 (t, *J* = 7.9 Hz, 9H), 0.61 (q, *J* = 7.9 Hz, 6H).

^{13}C{^{1}H} NMR (75 MHz, $CDCl_3$): δ 143.0, 128.9, 128.4, 125.2, 43.2, 7.6, 3.6.

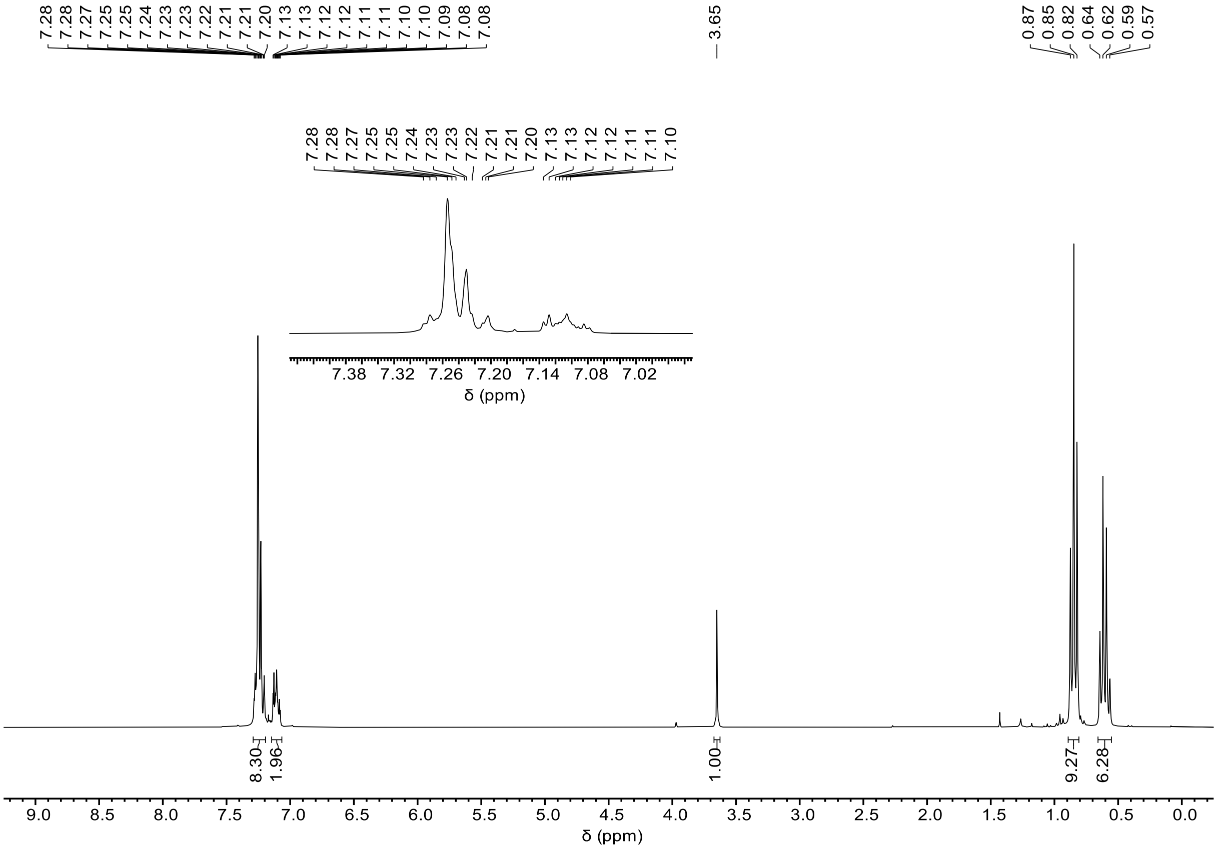

Figure S15. ^{1}H NMR of **1b** in $CDCl_3$.

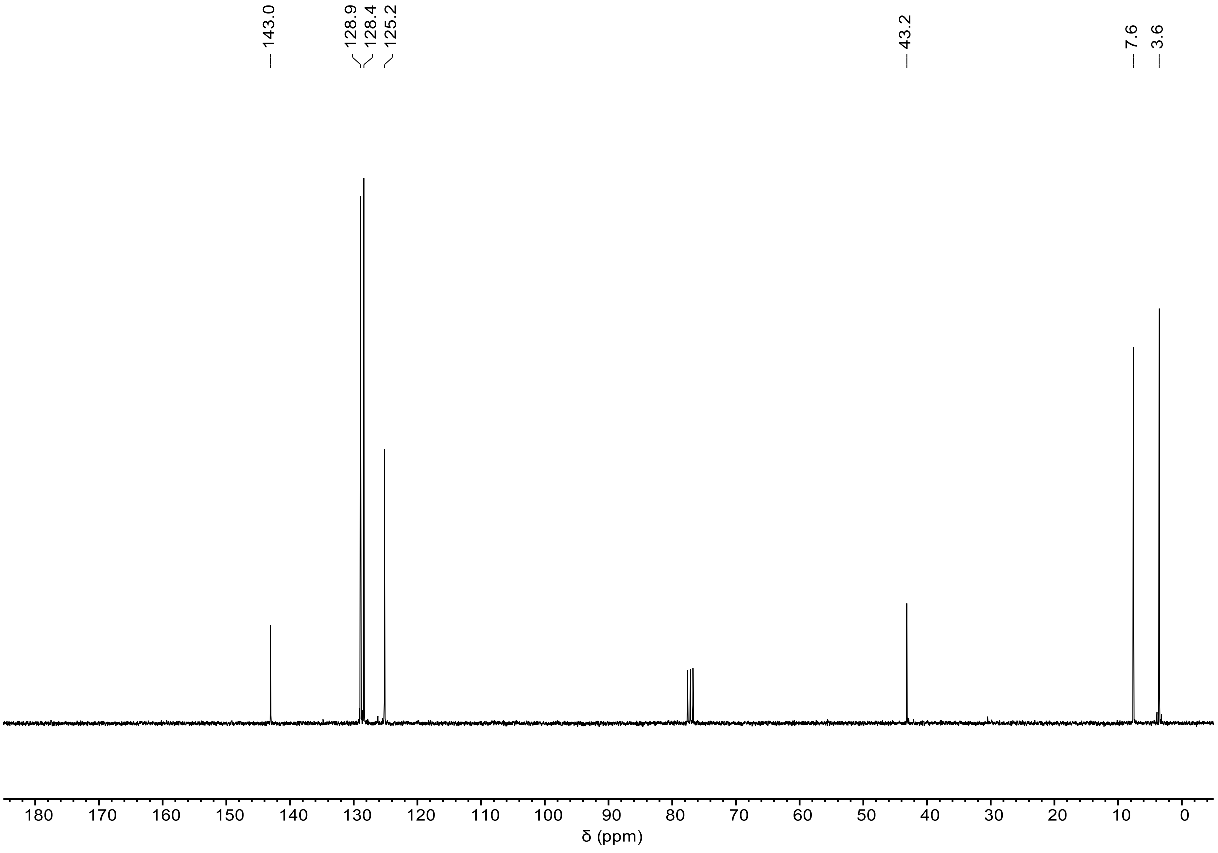

Figure S16. $^{13}C\{^1H\}$ NMR of **1b** in $CDCl_3$.

Triethyl(naphthalen-2-ylmethyl)silane (1c)

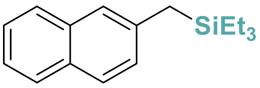

$C_{17}H_{24}Si$
Molecular Weight: 256.46

Prepared according to General Procedure A and purified by flash column chromatography in silica gel and hexane, compound **1c** was isolated as a colorless oil (110 mg, 86 % yield). Spectroscopic data are in accordance with literature reports.[8]

^{1}H NMR (300 MHz, $CDCl_3$): δ 7.78 (dd, J = 8.0, 1.2 Hz, 1H), 7.73 (dd, J = 7.9, 1.3 Hz, 1H), 7.70 (d, J = 8.0 Hz, 1H), 7.49 – 7.45 (m, 1H), 7.43 (ddd, J = 8.2, 6.9, 1.5 Hz, 1H), 7.37 (ddd, J = 8.1, 7.0, 1.5 Hz, 1H), 7.20 (dd, J = 8.4, 1.8 Hz, 1H), 2.29 (s, 2H), 0.97 (t, J = 7.9 Hz, 9H), 0.57 (q, J = 7.9 Hz, 6H).

^{13}C{^{1}H} NMR (75 MHz, $CDCl_3$): δ 138.6, 134.0, 131.1, 128.1, 127.7, 127.7, 127.1, 125.9, 125.4, 124.4, 22.1, 7.5, 3.2.

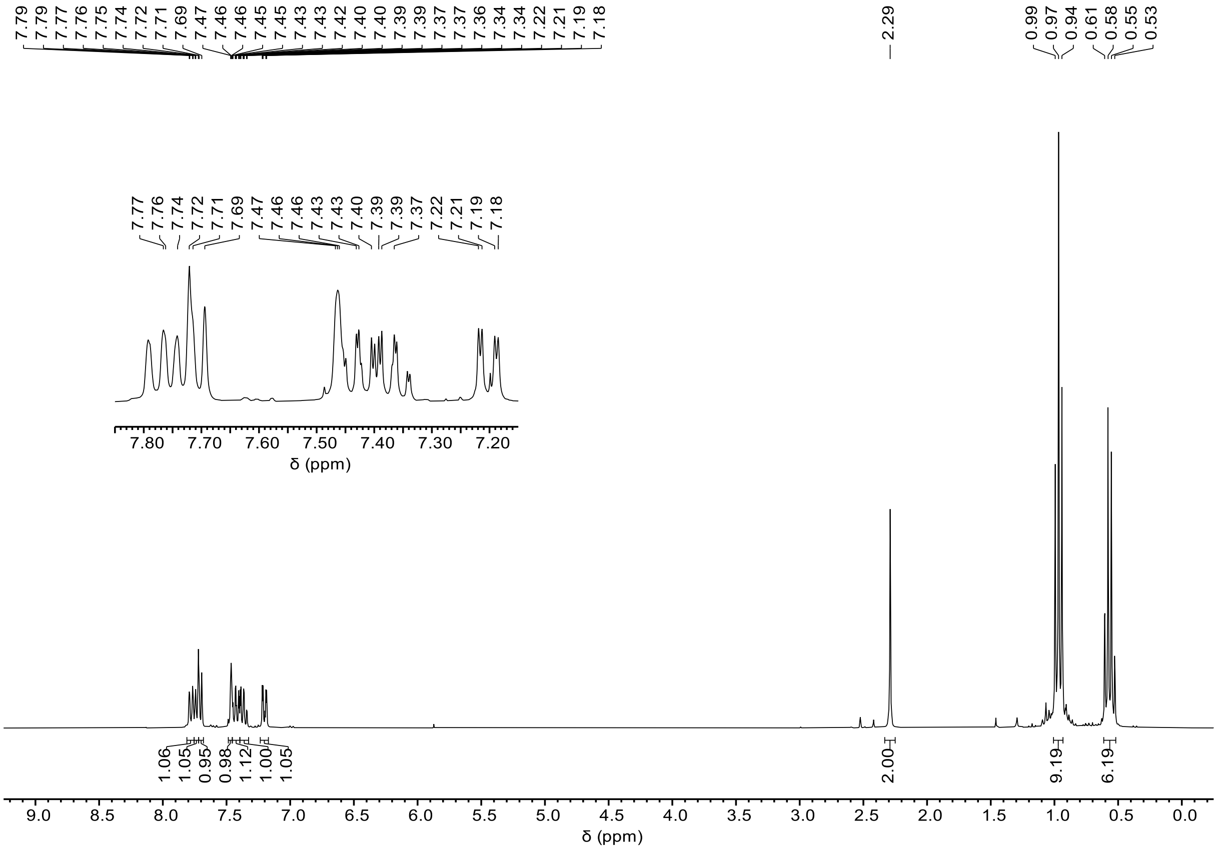

Figure S17. ^{1}H NMR of **1c** in $CDCl_3$.

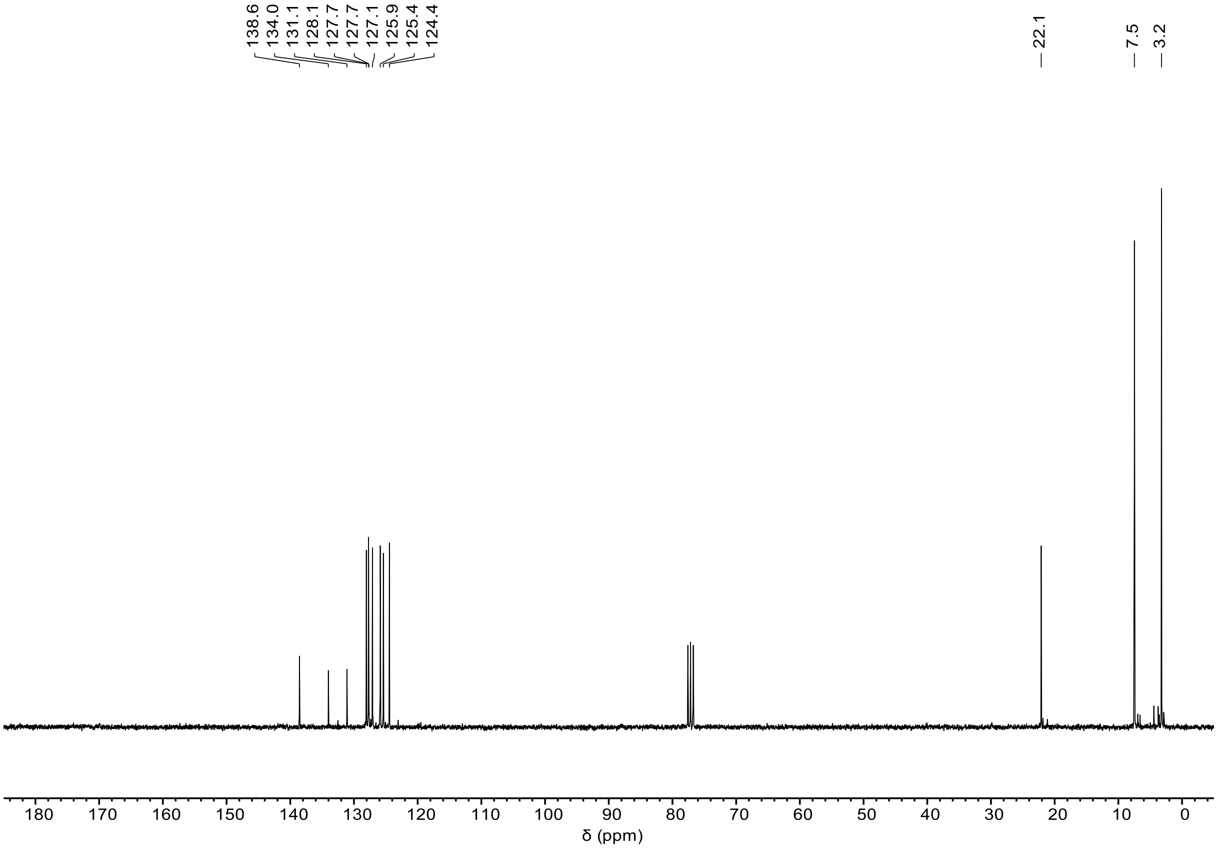

Figure S18. $^{13}C\{^1H\}$ NMR of **1c** in $CDCl_3$.

Triethyl(naphthalen-1-ylmethyl)silane (1d)

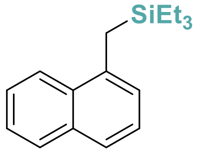

$C_{17}H_{24}Si$
Molecular Weight: 256.46

Prepared according to General Procedure A and purified by flash column chromatography in silica gel and hexane, compound **1d** was isolated as a colorless oil (86 mg, 67 % yield). Spectroscopic data are in accordance with literature reports.[9]

^{1}H NMR (300 MHz, $CDCl_3$): δ 8.05 – 7.95 (m, 1H), 7.87 – 7.80 (m, 1H), 7.62 (d, J = 8.1 Hz, 1H), 7.53 – 7.42 (m, 2H), 7.36 (dd, J = 8.1, 7.1 Hz, 1H), 7.21 (d, J = 7.1 Hz, 1H), 2.60 (s, 2H), 0.91 (t, J = 7.9 Hz, 9H), 0.54 (q, J = 7.9 Hz, 6H).

^{13}C{^{1}H} NMR (75 MHz, $CDCl_3$): δ 137.6, 134.1, 132.0, 128.8, 125.7, 125.5, 125.4, 125.1, 124.7, 124.7, 18.5, 7.5, 3.7.

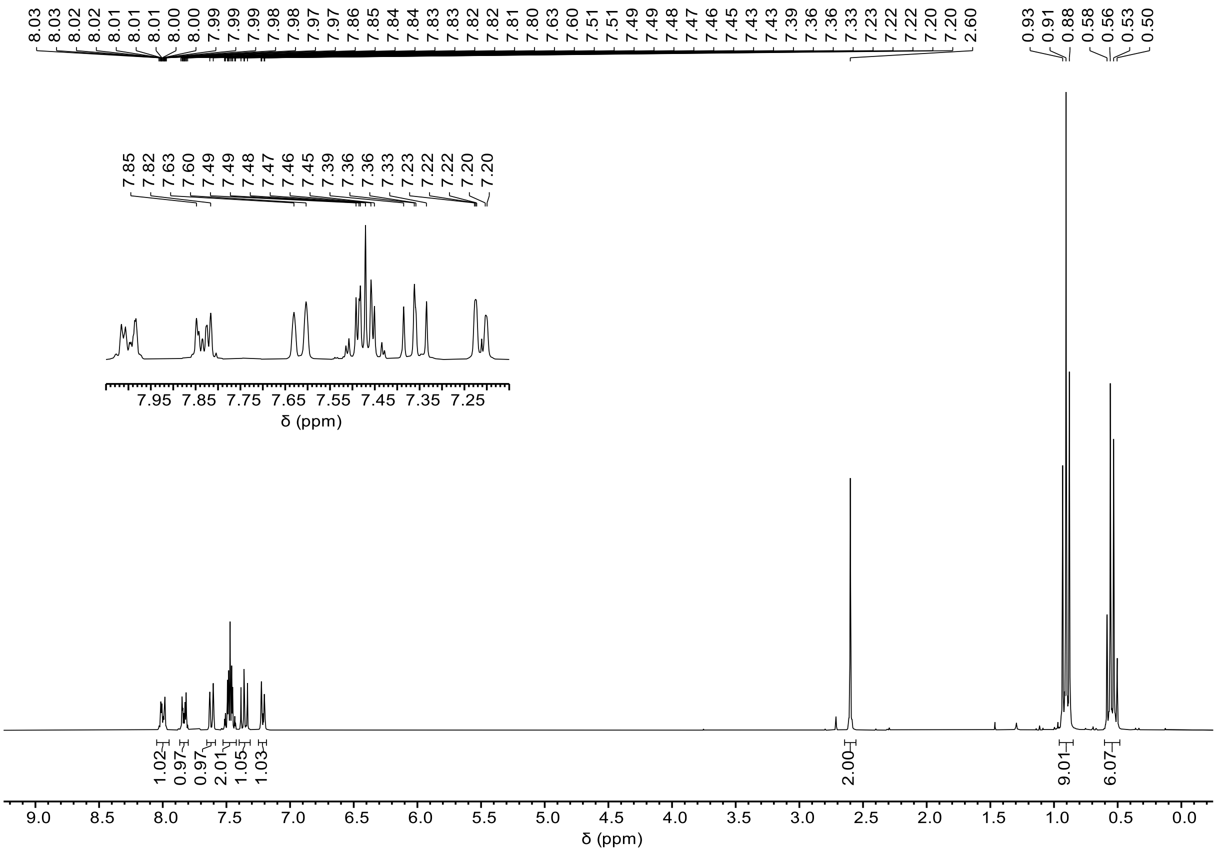

Figure S19. ^{1}H NMR of **1d** in $CDCl_3$.

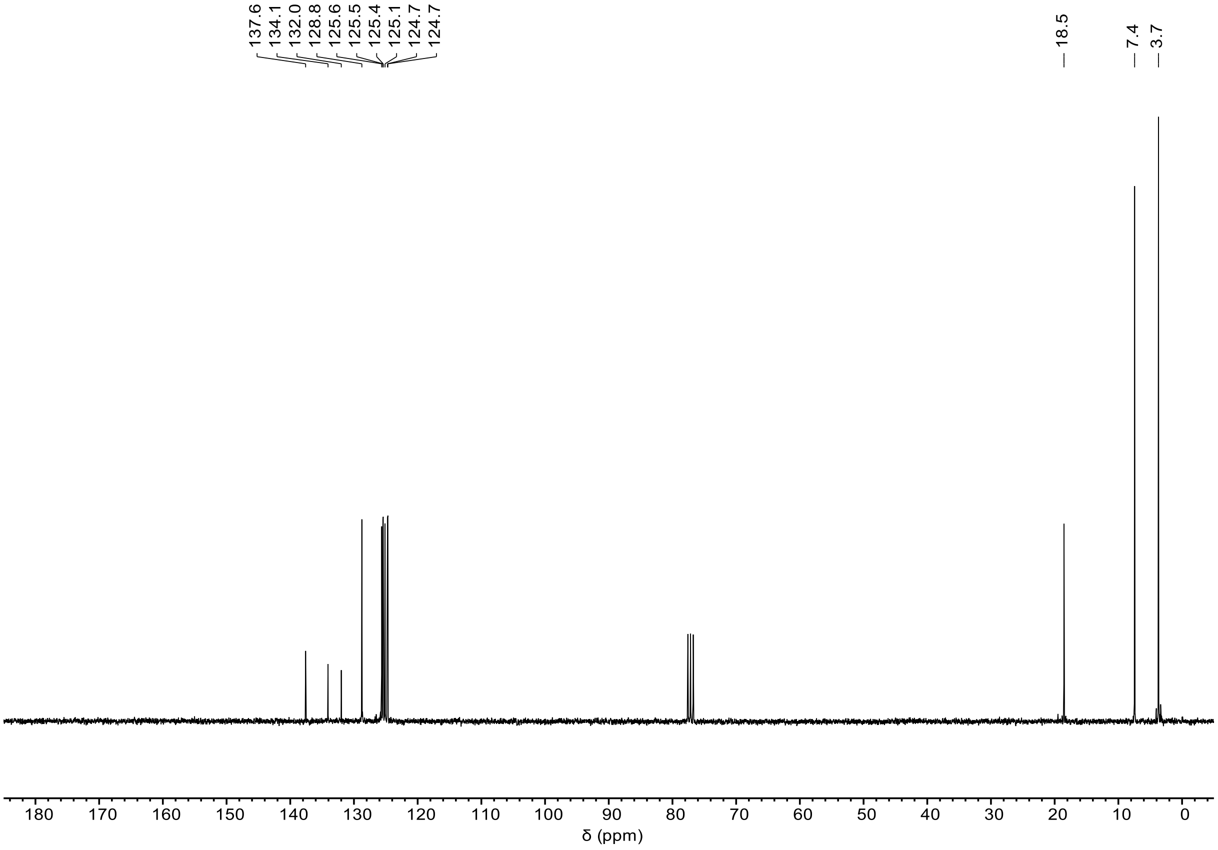

Figure S20. $^{13}C\{^1H\}$ NMR of **1d** in $CDCl_3$.

2-(Phenyl(triethylsilyl)methyl)pyridine (1e)

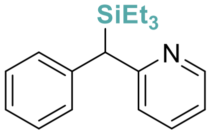

$C_{18}H_{25}NSi$
Molecular Weight: 283.49

Prepared according to General Procedure A and purified by flash column chromatography in silica gel and hexane/EtOAc (90:10), compound **1e** was isolated as a colorless oil (119 mg, 84 % yield). Spectroscopic data are in accordance with literature reports.[10]

^{1}H NMR (300 MHz, CDCl$_3$): δ 8.53 (ddd, J = 4.9, 2.1, 0.8 Hz, 1H), 7.50 – 7.38 (m, 3H), 7.29 – 7.19 (m, 2H), 7.16 – 7.05 (m, 2H), 6.98 (ddd, J = 7.6, 4.9, 1.0 Hz, 1H), 3.81 (s, 1H), 0.83 (t, J = 7.9 Hz, 9H), 0.65 – 0.54 (m, 6H).

^{13}C{^{1}H} NMR (75 MHz, CDCl$_3$): δ 163.2, 148.9, 141.8, 136.0, 128.7, 128.2, 125.1, 123.3, 120.1, 45.9, 7.5, 3.2.

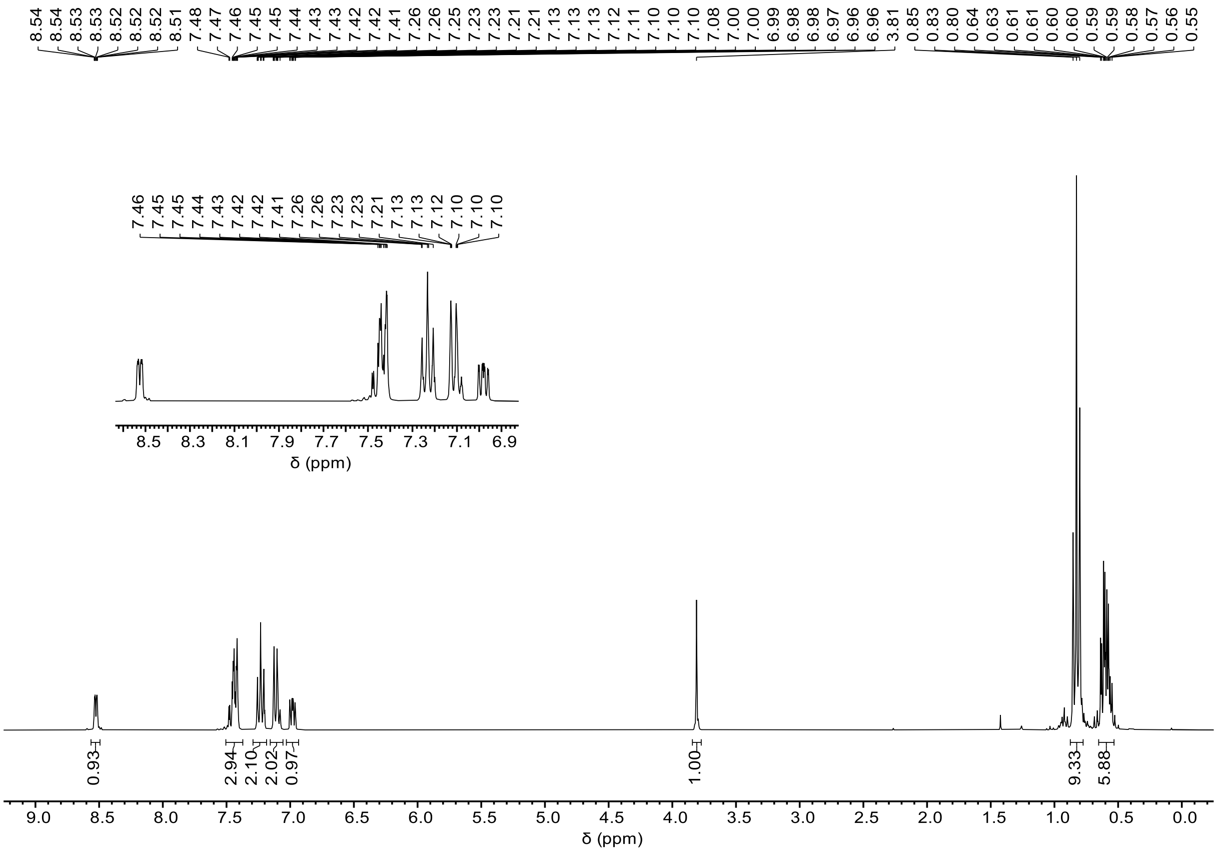

Figure S21. ^{1}H NMR of **1e** in CDCl$_3$.

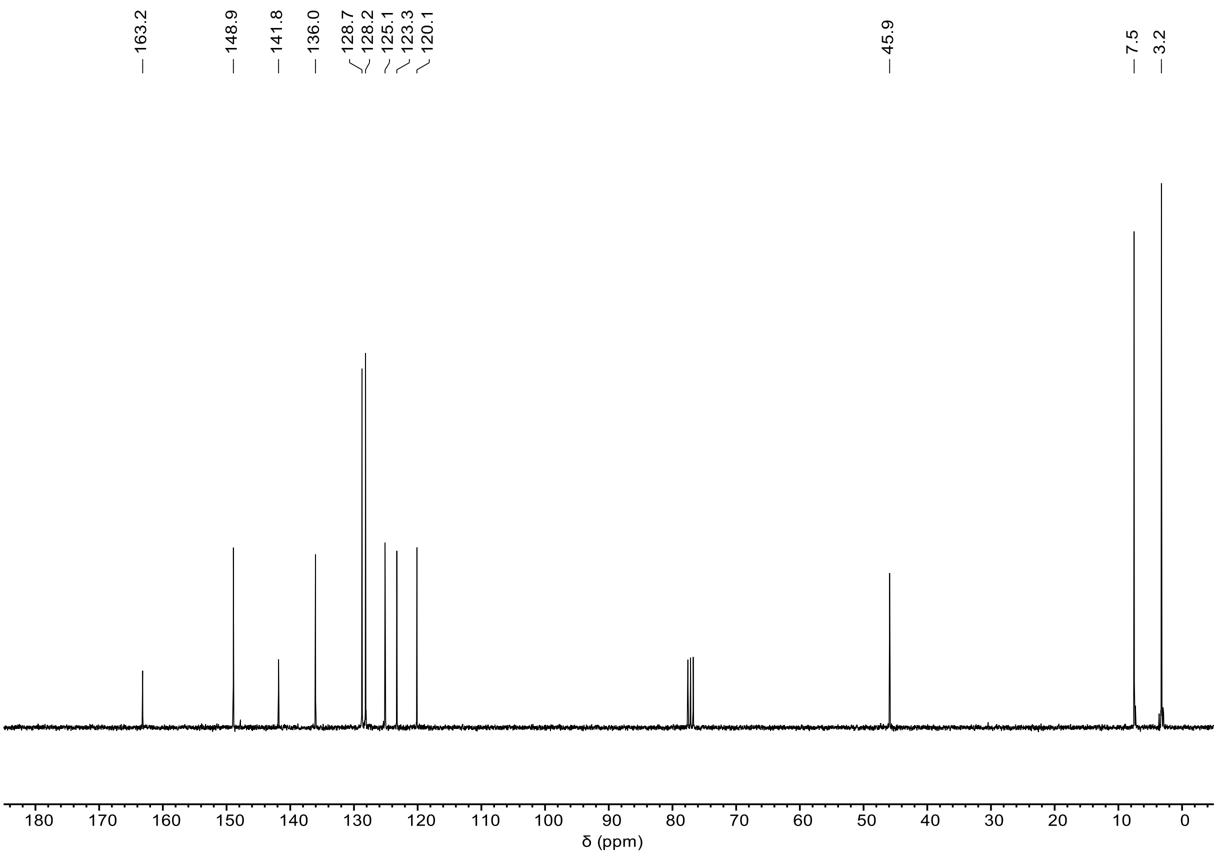

Figure S22. $^{13}C\{^{1}H\}$ NMR of **1e** in $CDCl_3$.

Triethyl(methoxy(phenyl)methyl)silane (1f)

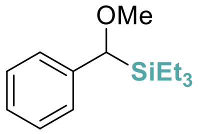

$C_{14}H_{24}OSi$
Molecular Weight: 236.43

Prepared according to General Procedure A and purified by flash column chromatography in silica gel and hexane, compound **1f** was isolated as a colorless oil (91 mg, 77 % yield). Spectroscopic data are in accordance with literature reports.[11]

^{1}H NMR (300 MHz, $CDCl_3$): *δ* 7.36 – 7.27 (m, 2H), 7.24 – 7.13 (m, 3H), 4.09 (s, 1H), 3.29 (s, 3H), 0.94 (t, *J* = 7.9 Hz, 9H), 0.63 – 0.51 (m, 6H).

^{13}C{^{1}H} NMR (75 MHz, $CDCl_3$): *δ* 141.9, 128.3, 126.0, 125.8, 79.1, 59.2, 7.5, 1.7.

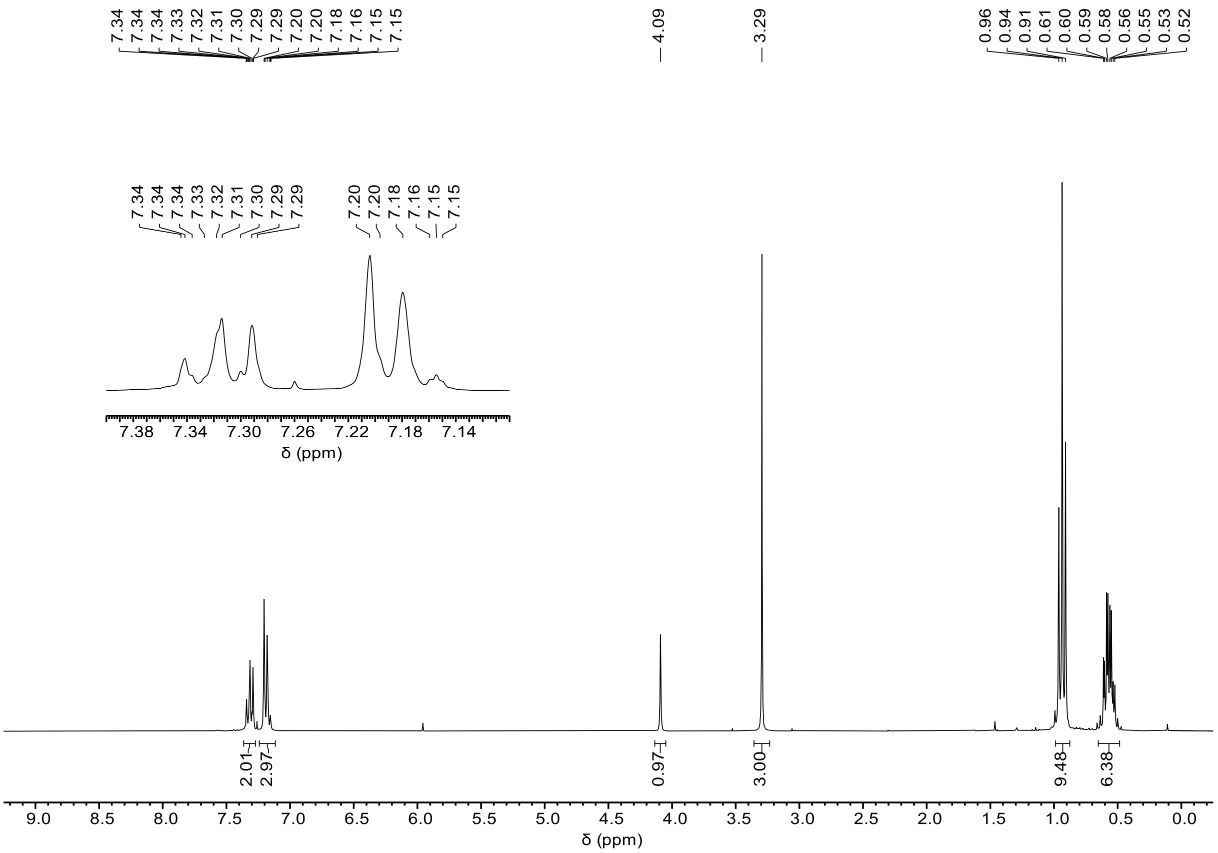

Figure S23. ^{1}H NMR of **1f** in $CDCl_3$.

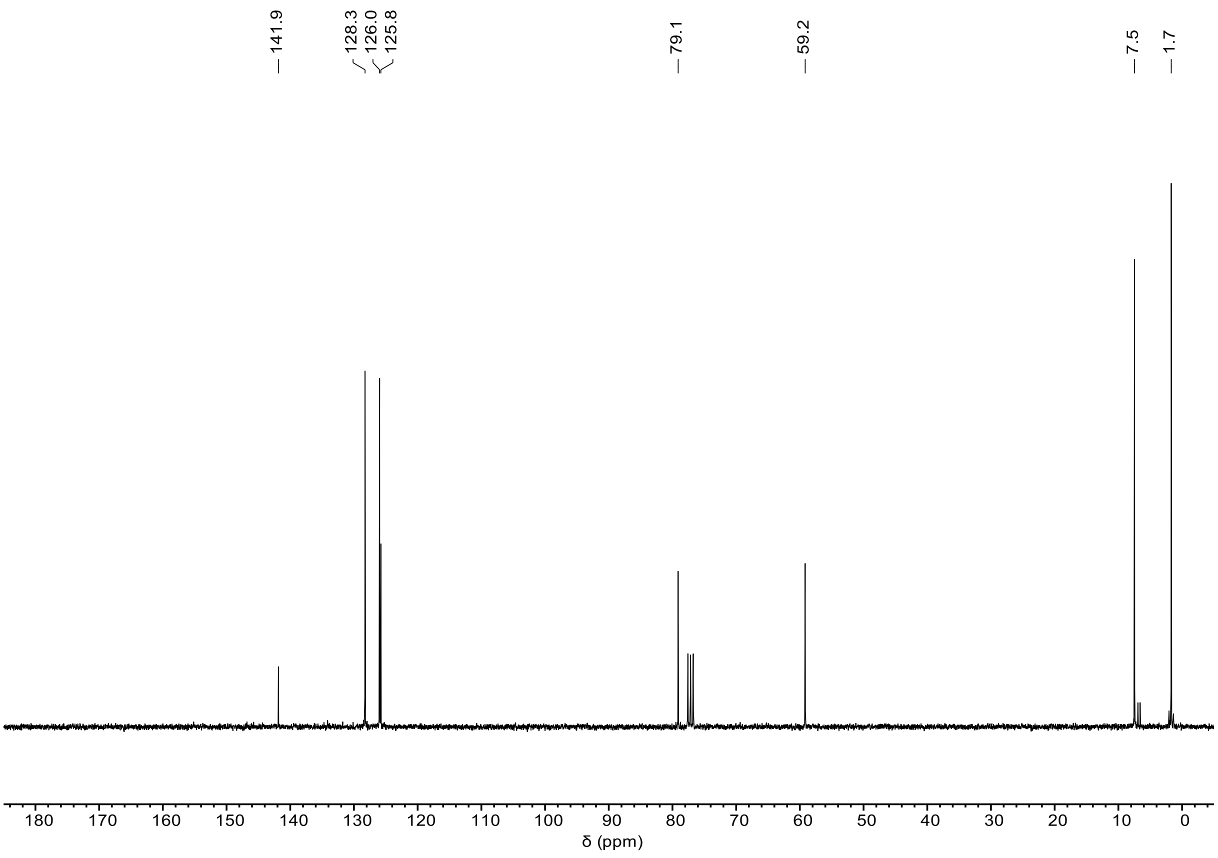

Figure S24. $^{13}C\{^1H\}$ NMR of **1f** in $CDCl_3$.

Triethyl(2-methoxybenzyl)silane (1g)

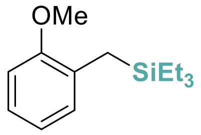

$C_{14}H_{24}OSi$
Molecular Weight: 236.43

Prepared according to General Procedure A and purified by flash column chromatography in silica gel and hexane, compound **1h** was isolated as a colorless oil (90 mg, 76 % yield). Spectroscopic data are in accordance with literature reports.[10]

^{1}H NMR (300 MHz, $CDCl_3$): *δ* 7.10 (td, *J* = 7.7, 1.7 Hz, 1H), 7.04 (dd, *J* = 7.5, 1.6 Hz, 1H), 6.86 (td, *J* = 7.3, 1.0 Hz, 1H), 6.82 (dd, *J* = 7.5, 1.0 Hz, 1H), 3.82 (s, 3H), 2.16 (s, 2H), 0.96 (t, *J* = 7.9 Hz, 9H), 0.54 (q, *J* = 7.9 Hz, 6H).

^{13}C{^{1}H} NMR (75 MHz, $CDCl_3$): *δ* 156.6, 129.6, 125.0, 120.3, 109.8, 54.9, 15.4, 7.4, 3.5.

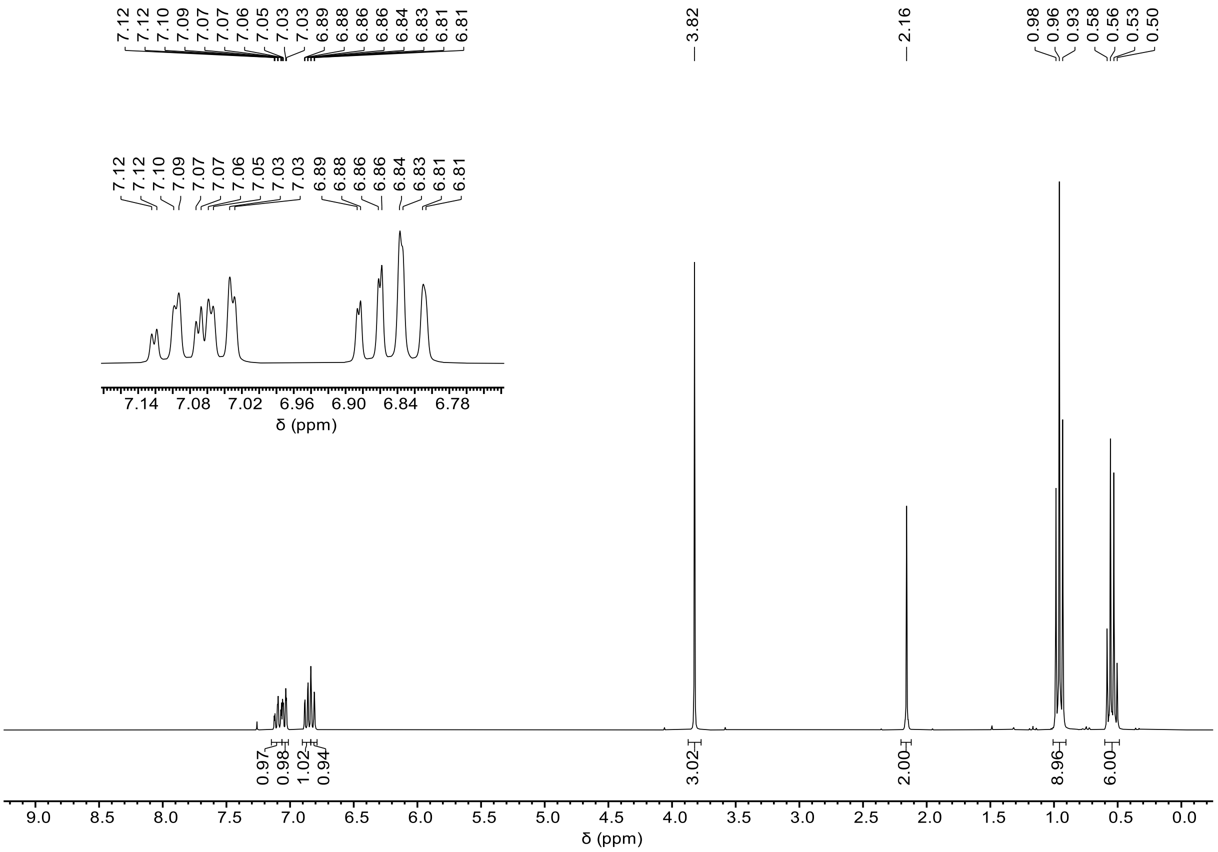

Figure S25. ^{1}H NMR of **1g** in $CDCl_3$.

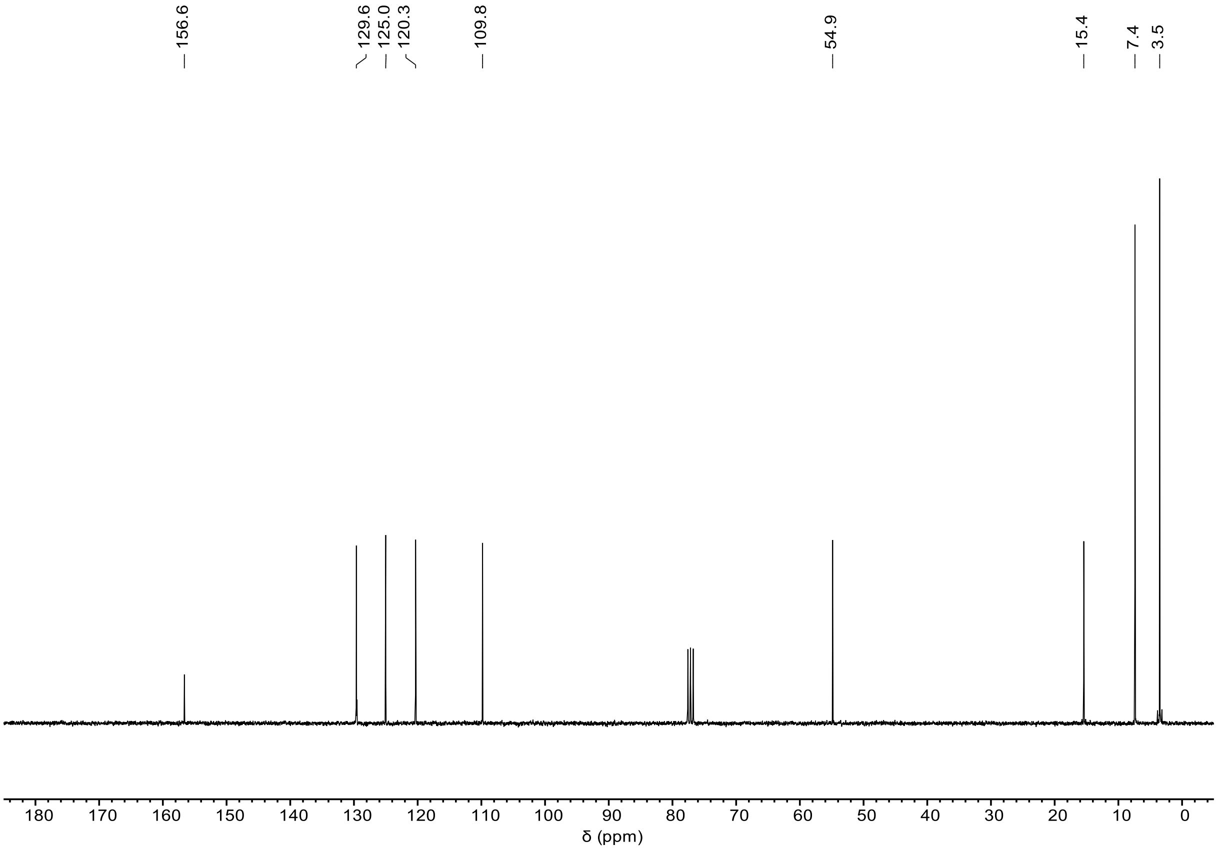

Figure S26. $^{13}C\{^{1}H\}$ NMR of **1g** in $CDCl_3$.

2-((Triethylsilyl)methyl)pyridine (1h)

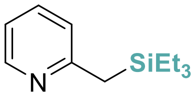

$C_{12}H_{21}NSi$
Molecular Weight: 207.39

Prepared according to General Procedure A and purified by flash column chromatography in silica gel and hexane/EtOAc (90:10), compound **1g** was isolated as a colorless oil (74 mg, 71 % yield). Spectroscopic data are in accordance with literature reports.[10]

^{1}H NMR (300 MHz, $CDCl_3$): δ 8.40 (ddd, J = 4.9, 1.9, 1.0 Hz, 1H), 7.45 (td, J = 7.7, 1.9 Hz, 1H), 6.98 – 6.89 (m, 2H), 2.34 (s, 2H), 0.89 (t, J = 7.9 Hz, 9H), 0.53 (q, J = 7.9 Hz, 6H).

^{13}C{^{1}H} NMR (75 MHz, $CDCl_3$): δ 161.8, 149.1, 135.8, 122.3, 119.1, 25.5, 7.3, 3.4.

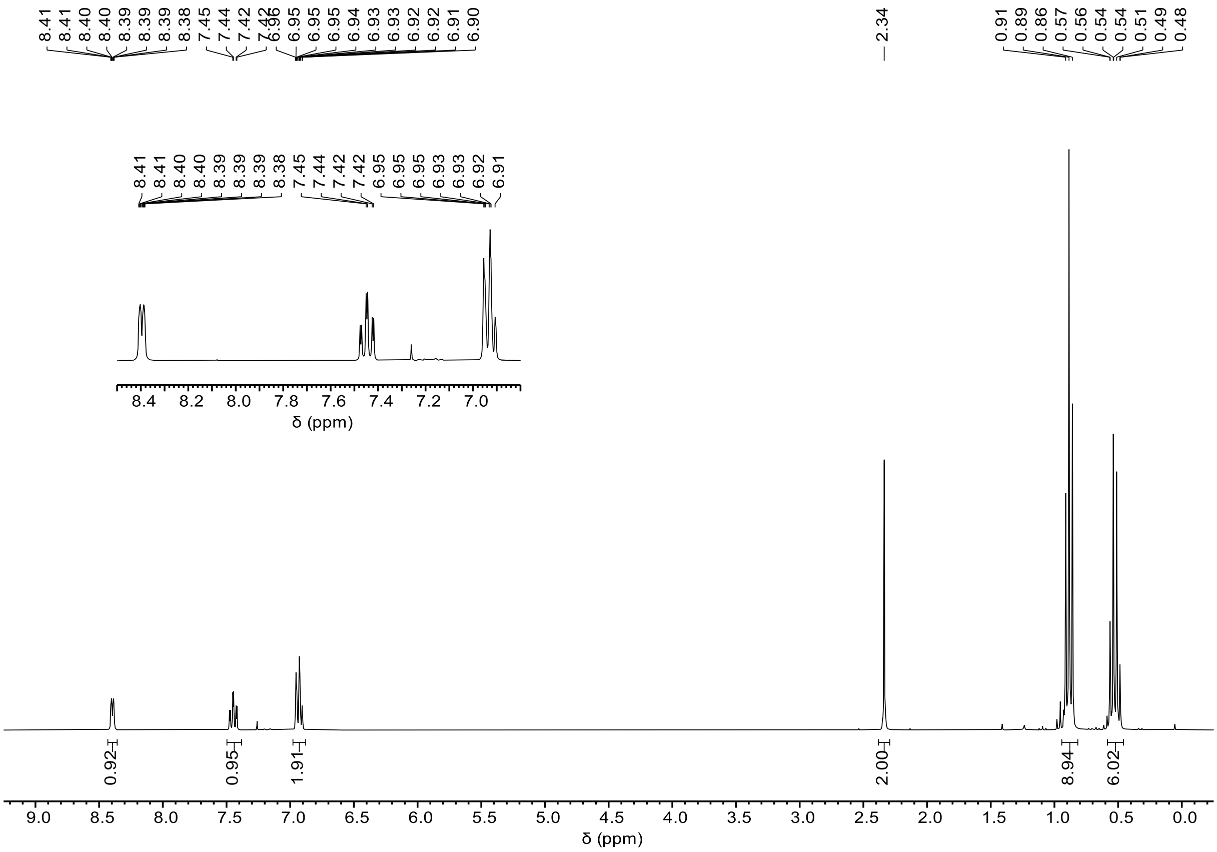

Figure S27. ^{1}H NMR of **1h** in $CDCl_3$.

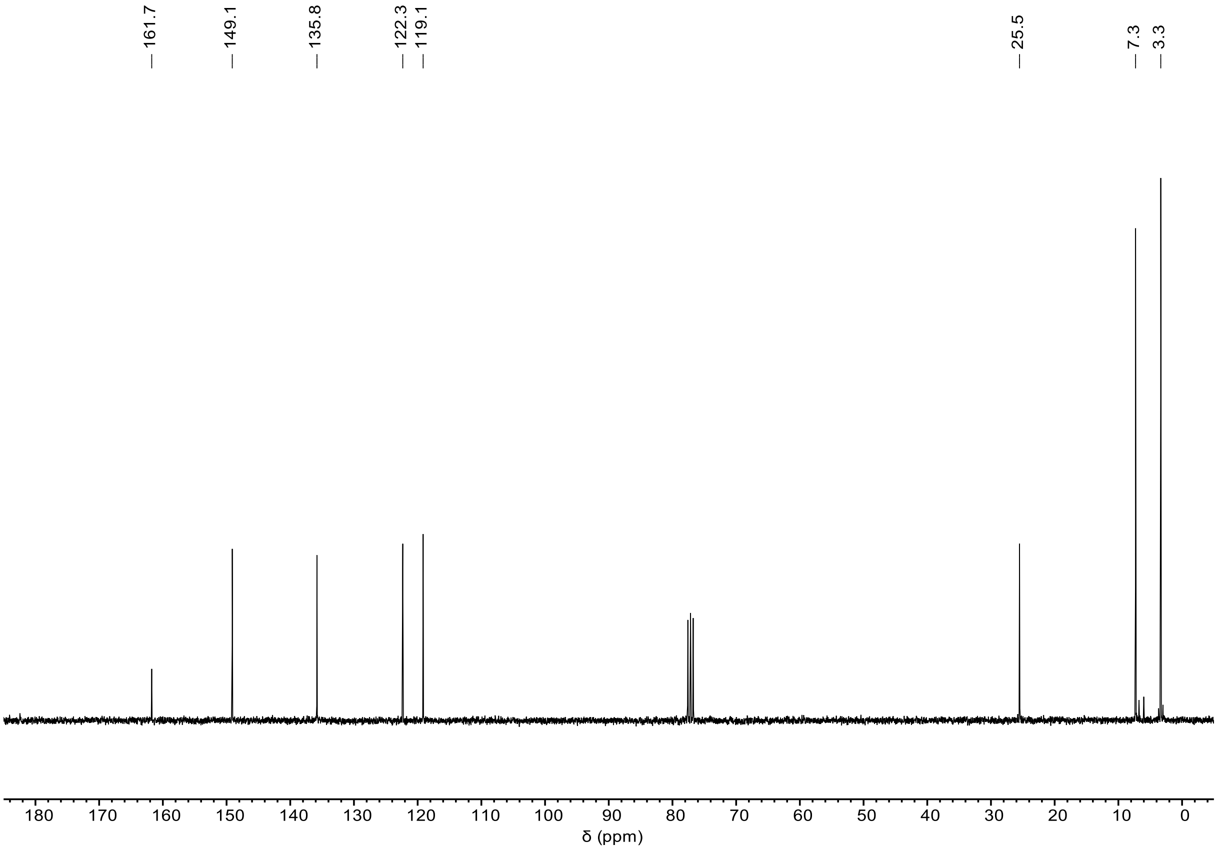

Figure S28. $^{13}C\{^{1}H\}$ NMR of **1h** in $CDCl_3$.

4-((Triethylsilyl)methyl)pyridine (1i)

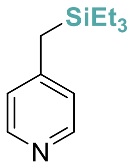

$C_{12}H_{21}NSi$
Molecular Weight: 207.39

Prepared according to General Procedure A and purified by flash column chromatography in silica gel and hexane/EtOAc (50:50), compound **1i** was isolated as a colorless oil (67 mg, 65 % yield). Spectroscopic data are in accordance with literature reports.[12]

^{1}H NMR (300 MHz, $CDCl_3$): δ 8.38 – 8.31 (m, 2H), 6.93 – 6.86 (m, 2H), 2.08 (s, 2H), 0.90 (t, *J* = 7.9 Hz, 9H), 049 (q, *J* = 7.9 Hz, 6H).

^{13}C{^{1}H} NMR (75 MHz, $CDCl_3$): δ 150.6, 149.4, 123.7, 22.1, 7.3, 2.9.

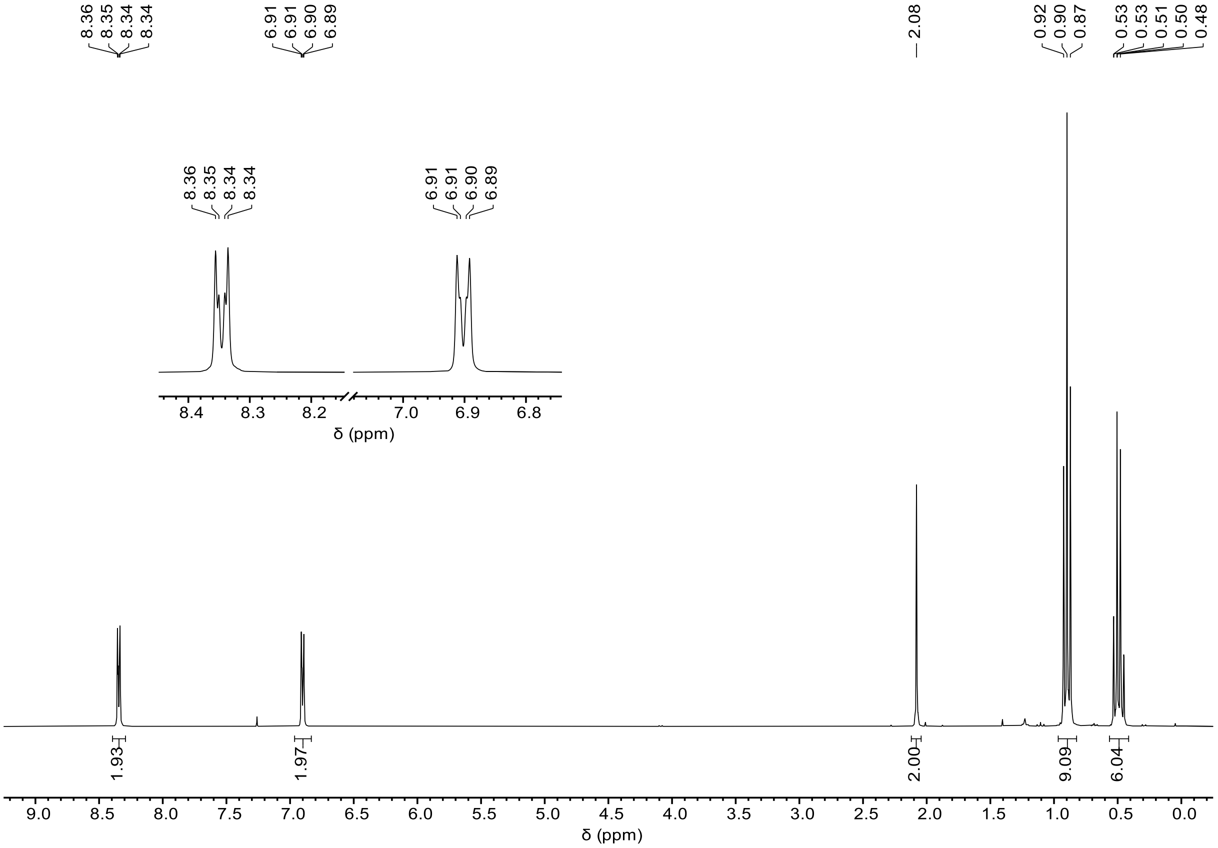

Figure S29. ^{1}H NMR of **1i** in $CDCl_3$.

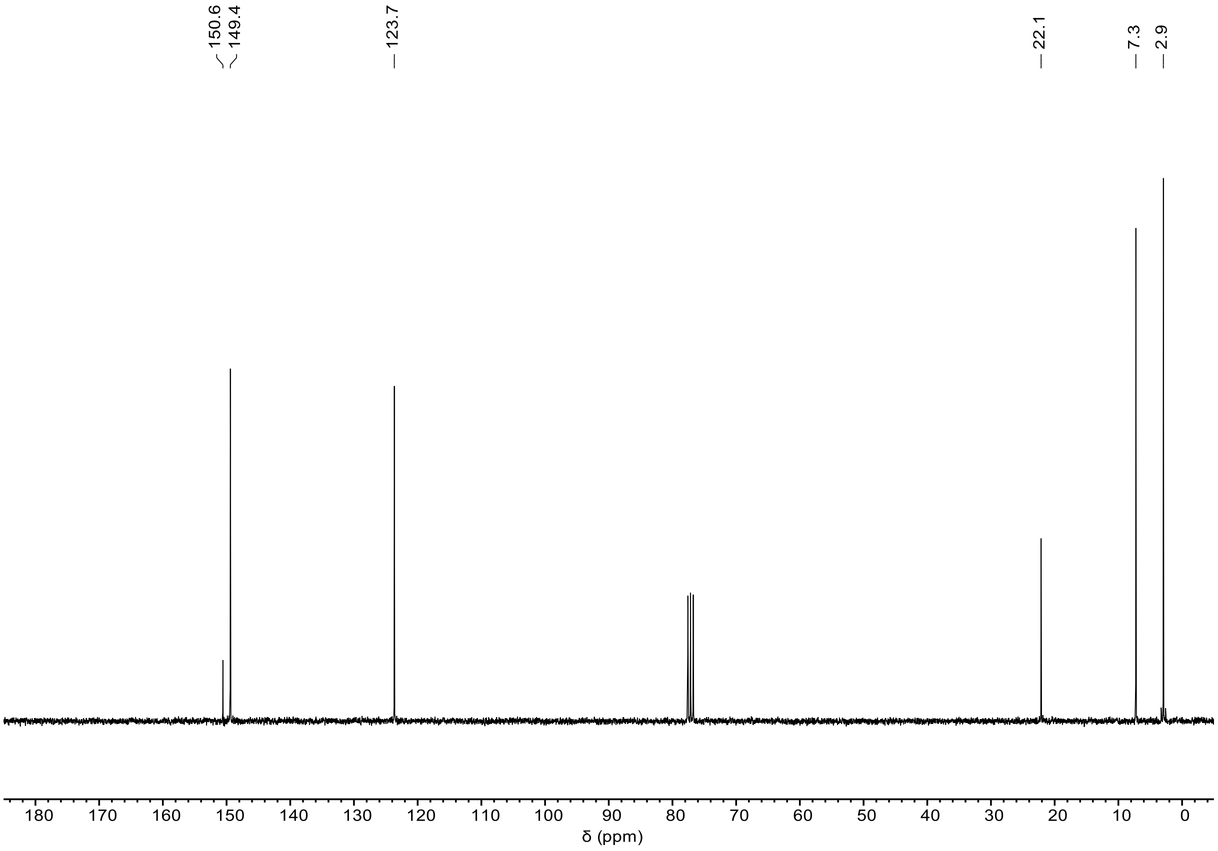

Figure S30. $^{13}C\{^1H\}$ NMR of **1i** in $CDCl_3$.

Triethyl(2-fluoro-3-methylbenzyl)silane (1j)

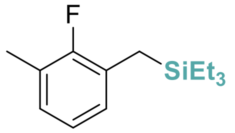

$C_{14}H_{23}FSi$
Molecular Weight: 238.42

Prepared according to General Procedure A and purified by flash column chromatography in silica gel and hexane, compound **1j** was isolated as a colorless oil (81 mg, 68 % yield).

^{1}H NMR (300 MHz, $CDCl_3$): δ 6.81 – 6.72 (m, 3H), 2.15 (d, J = 2.1 Hz), 1.98 (d, J = 2.1 Hz), 0.82 (t, J = 7.9 Hz, 9H), 0.43 (q, J = 7.9 Hz, 6H).

^{13}C{^{1}H} NMR (75 MHz, $CDCl_3$): δ 159.0 (d, J = 241.5 Hz), 128.0 (d, J = 5.1 Hz), 127.5 (d, J = 17.7 Hz), 127.1 (d, J = 4.7 Hz), 124.5 (d, J = 18.3 Hz), 123.2 (d, J = 4.2 Hz), 14.9 (d, J = 4.6 Hz), 14.4 (d, J = 3.2 Hz), 7.3, 3.3.

^{19}F{^{1}H} NMR (282 MHz, $CDCl_3$): δ -121.7.

HRMS (EI) m/z: $[M]^+$ Calculated for $C_{14}H_{23}FSi$ 238.1548. Found 238.1551.

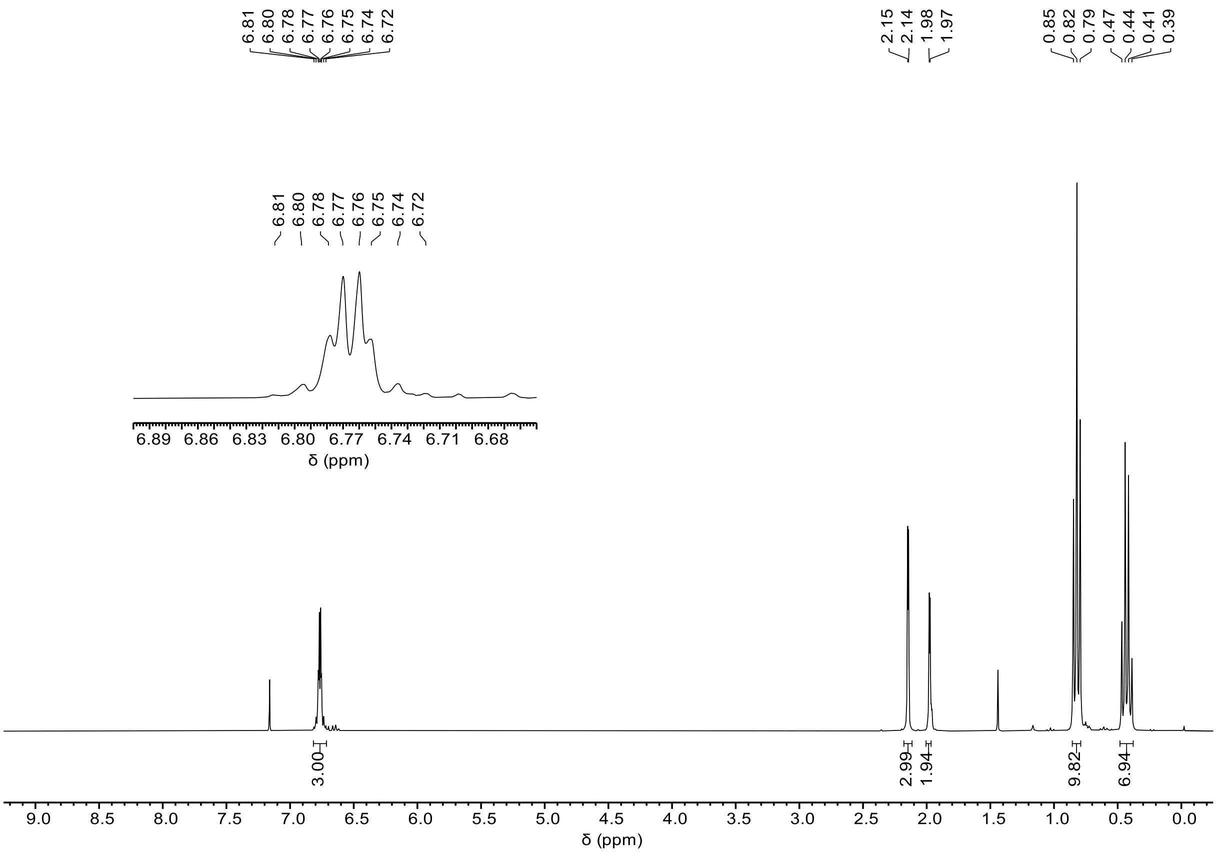

Figure S31. ^{1}H NMR of **1j** in $CDCl_3$.

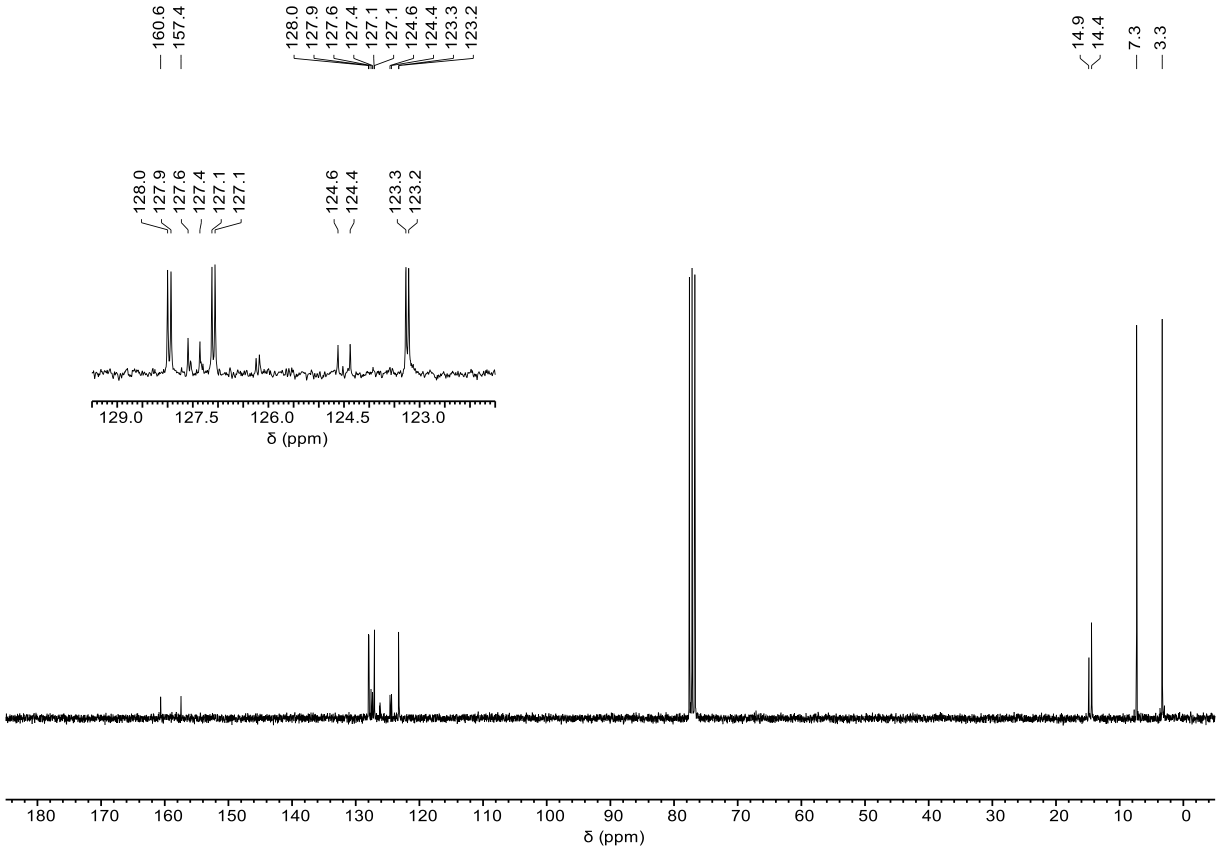

Figure S32. $^{13}C\{^{1}H\}$ NMR of **1j** in $CDCl_3$.

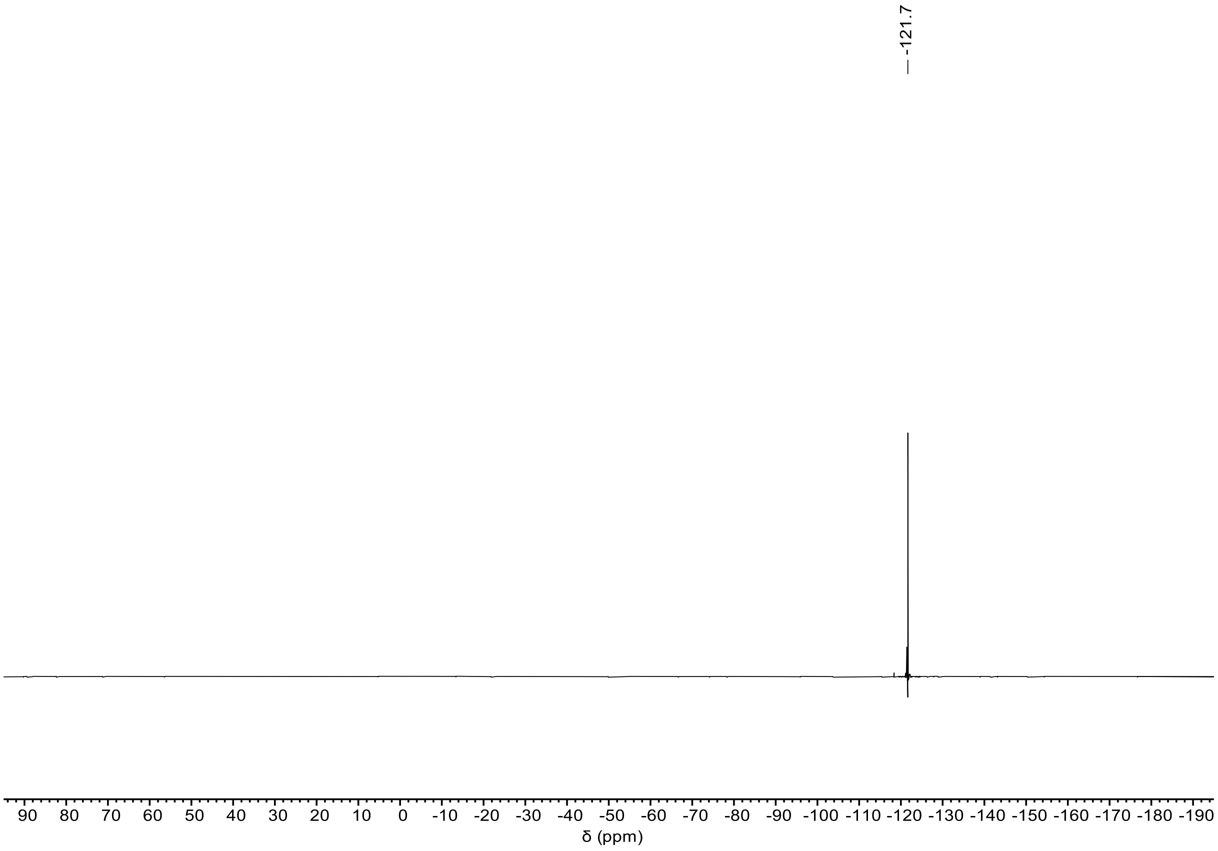

Figure S33. $^{19}F\{^1H\}$ NMR of **1j** in $CDCl_3$.

Triethyl(2-methoxyphenyl)silane (2a)

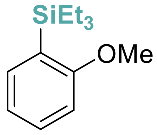

$C_{13}H_{22}OSi$
Molecular Weight: 222.40

Prepared according to General Procedure A and purified by flash column chromatography in silica gel and hexane, compound **2a** was isolated as a colorless oil (83 mg, 75 % yield). Spectroscopic data are in accordance with literature reports.[13]

^{1}H NMR (300 MHz, $CDCl_3$): *δ* 7.44 – 7.34 (m, 2H), 6.99 (td, *J* = 7.3, 0.9 Hz, 1H), 6.87 (d, *J* = 8.1 Hz, 1H), 3.82 (s, 3H), 1.04 – 0.95 (m, 9H), 0.92 – 0.82 (m, 6H).

^{13}C{^{1}H} NMR (75 MHz, $CDCl_3$): *δ* 164.7, 136.1, 130.6, 125.2, 120.5, 109.5, 55.0, 7.7, 3.7.

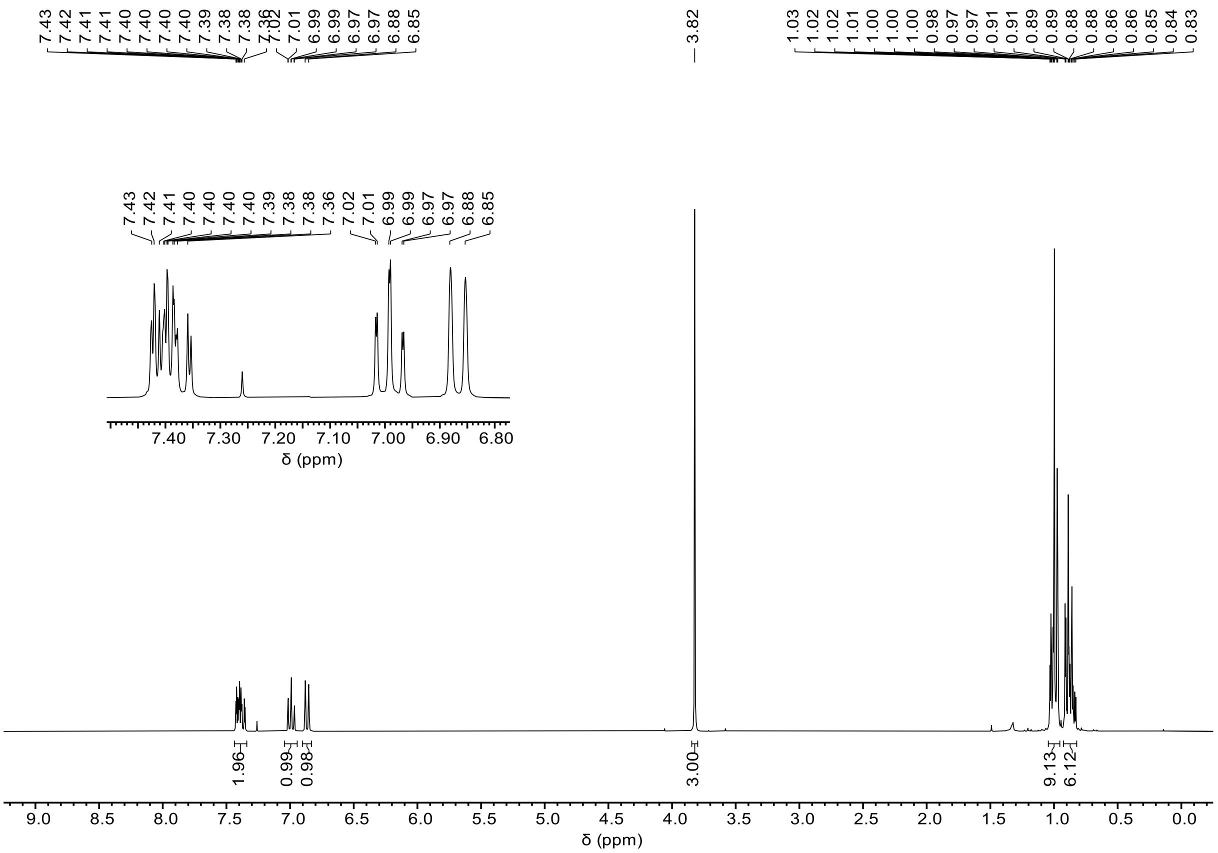

Figure S34. ^{1}H NMR of **2a** in $CDCl_3$.

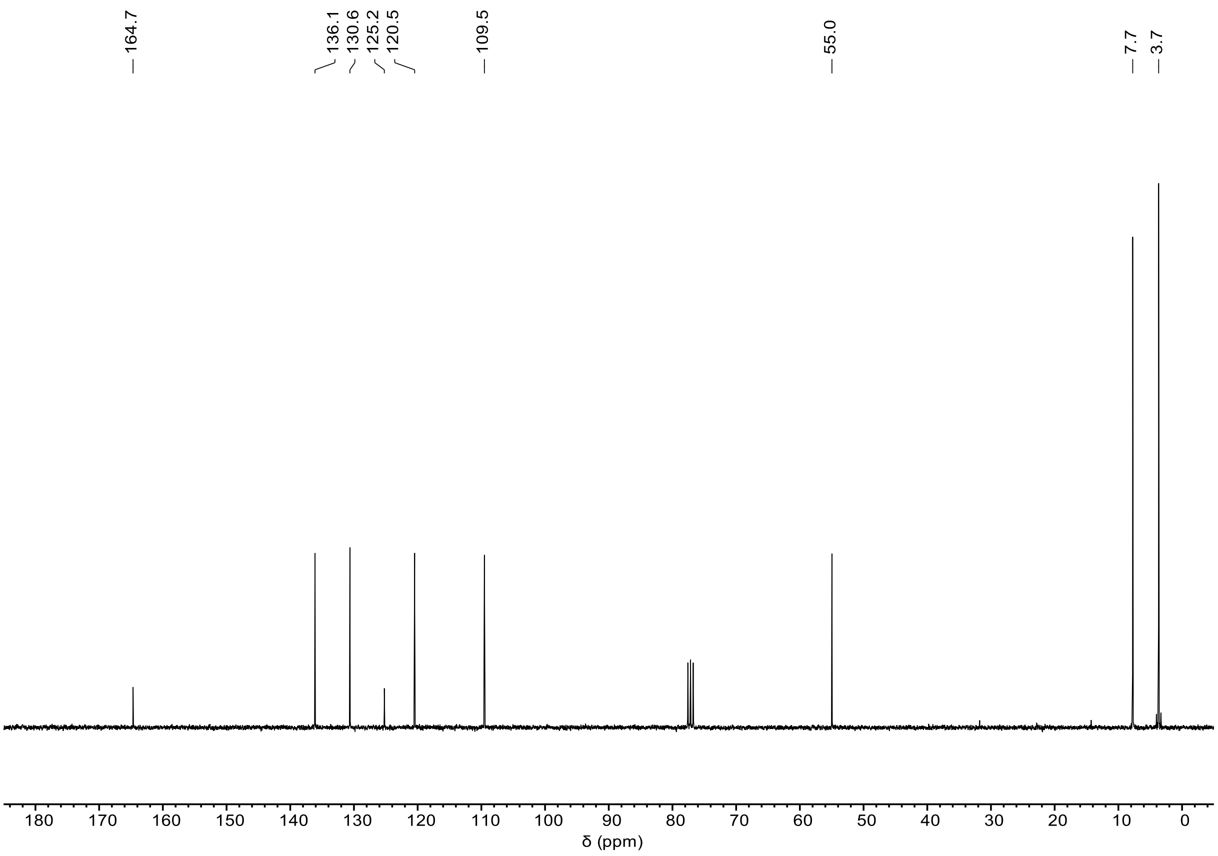

Figure S35. $^{13}C\{^1H\}$ NMR of **2a** in $CDCl_3$.

(2,6-Dimethoxyphenyl)triethylsilane (2b)

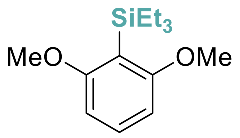

$C_{14}H_{24}O_2Si$
Molecular Weight: 252.43

Prepared according to General Procedure A and purified by flash column chromatography in silica gel and hexane, compound **2b** was isolated as a colorless oil (90 mg, 71 % yield). Spectroscopic data are in accordance with literature reports.[14]

^{1}H NMR (300 MHz, $CDCl_3$): *δ* 7.28 (t, *J* = 8.2 Hz, 1H), 6.50 (d, *J* = 8.2 Hz, 2H), 3.75 (s, 6H), 1.00 – 0.91 (m, 9H), 0.90 – 0.80 (m, 6H).

^{13}C{^{1}H} NMR (75 MHz, $CDCl_3$): *δ* 166.1, 131.3, 112.1, 103.5, 55.2, 8.0, 5.5.

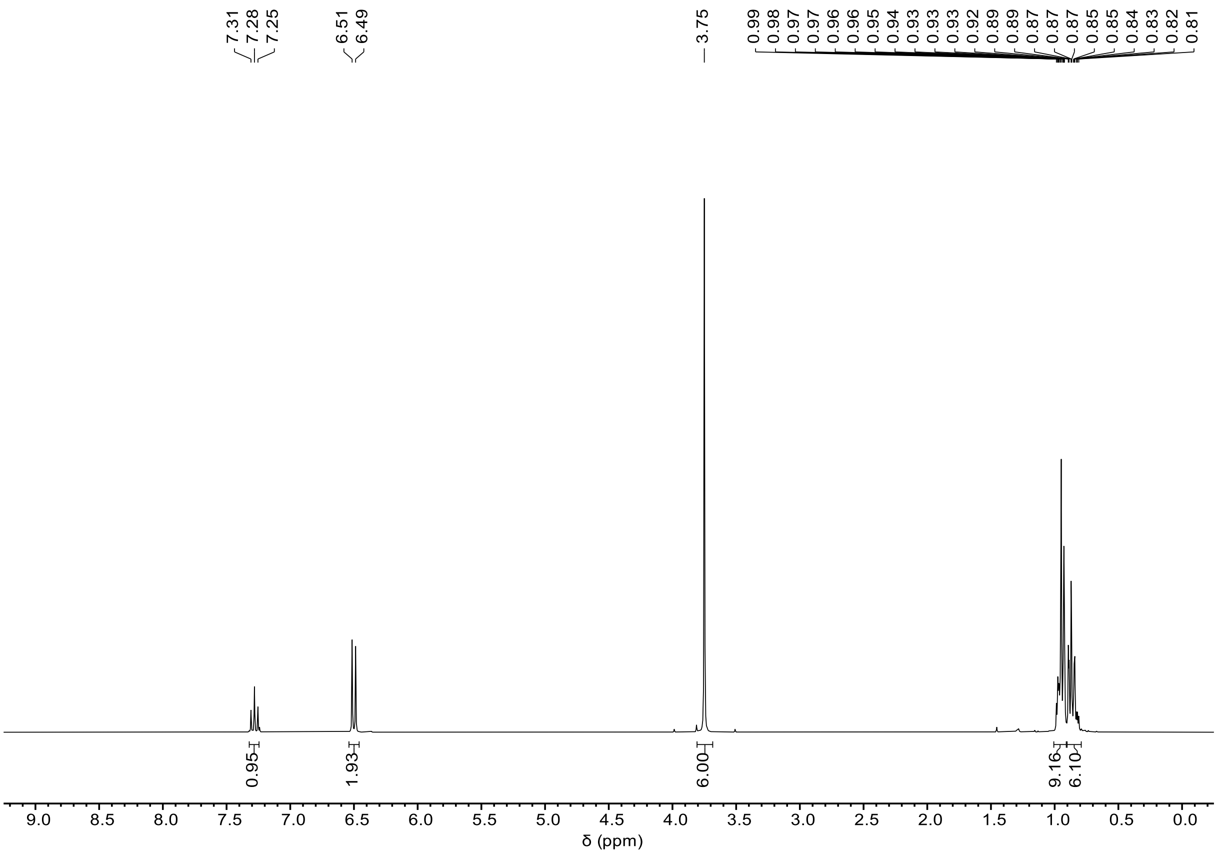

Figure S36. ^{1}H NMR of **2b** in $CDCl_3$.

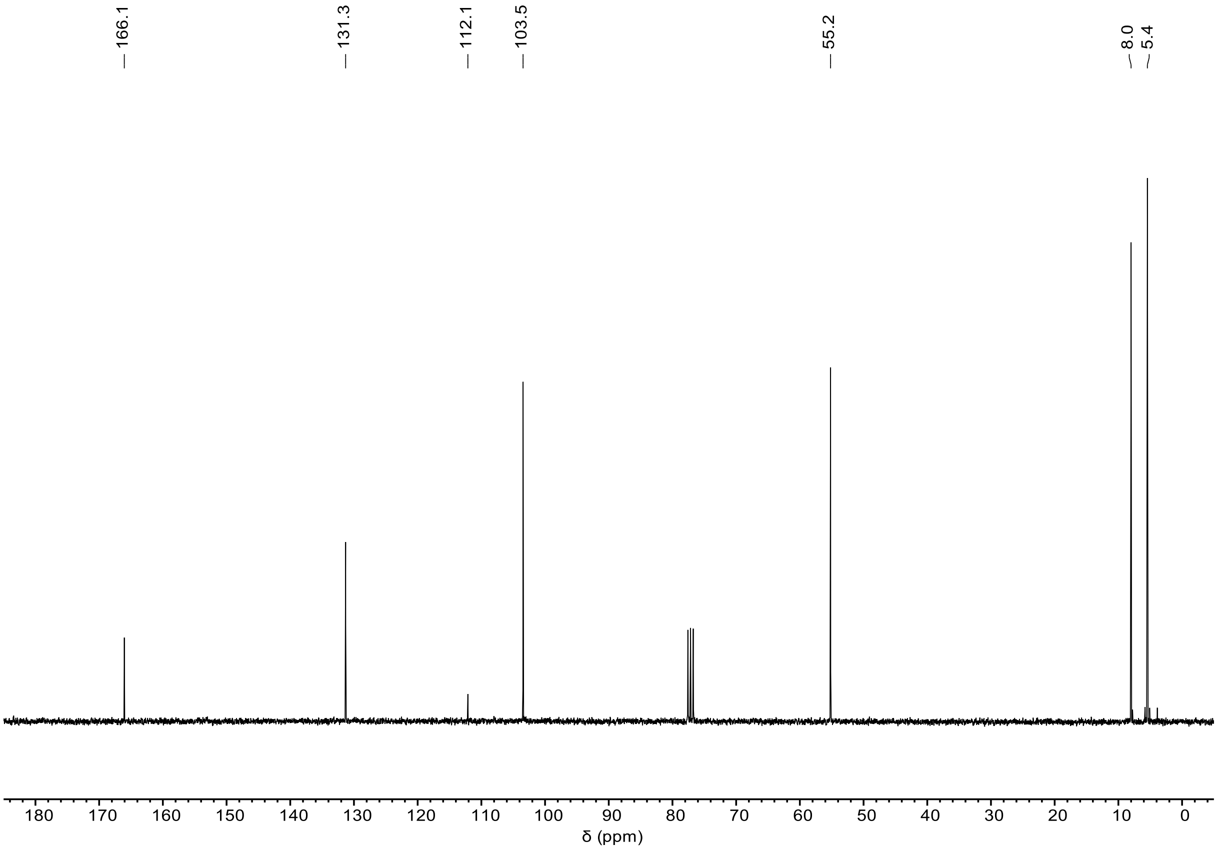

Figure S37. $^{13}C\{^1H\}$ NMR of **2b** in $CDCl_3$.

Triethyl(2-methoxy-5-methylphenyl)silane (2c)

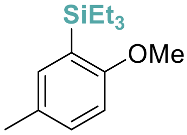

$C_{14}H_{24}OSi$
Molecular Weight: 236.43

Prepared according to General Procedure A using 0.75 mmol (1.5 eq.) of substrate and purified by flash column chromatography in silica gel and hexane, compound **2c** was isolated as a colorless oil (84 mg, 71 % yield). Spectroscopic data are in accordance with literature reports.[15]

^{1}H NMR (300 MHz, $CDCl_3$): *δ* 7.22 – 7.13 (m, 2H), 6.77 (dd, *J* = 8.0, 0.9 Hz, 1H), 3.79 (s, 3H), 2.34 (s, 3H), 1.04 – 0.96 (m, 9H), 0.91 – 0.81 (m, 6H).

^{13}C{^{1}H} NMR (75 MHz, $CDCl_3$): *δ* 162.8, 136.7, 131.0, 129.3, 125.0, 109.5, 55.1, 20.7, 7.8, 3.7.

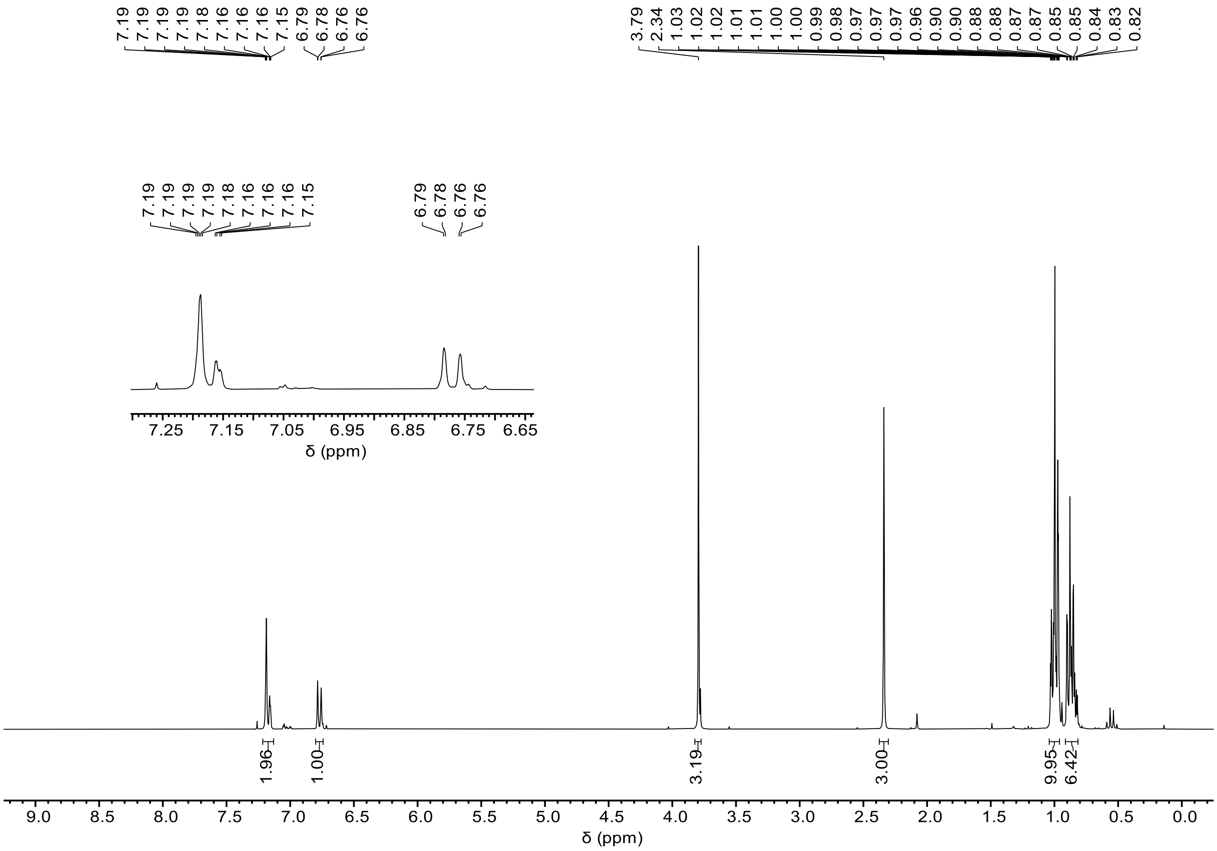

Figure S38. ^{1}H NMR of **2c** in $CDCl_3$.

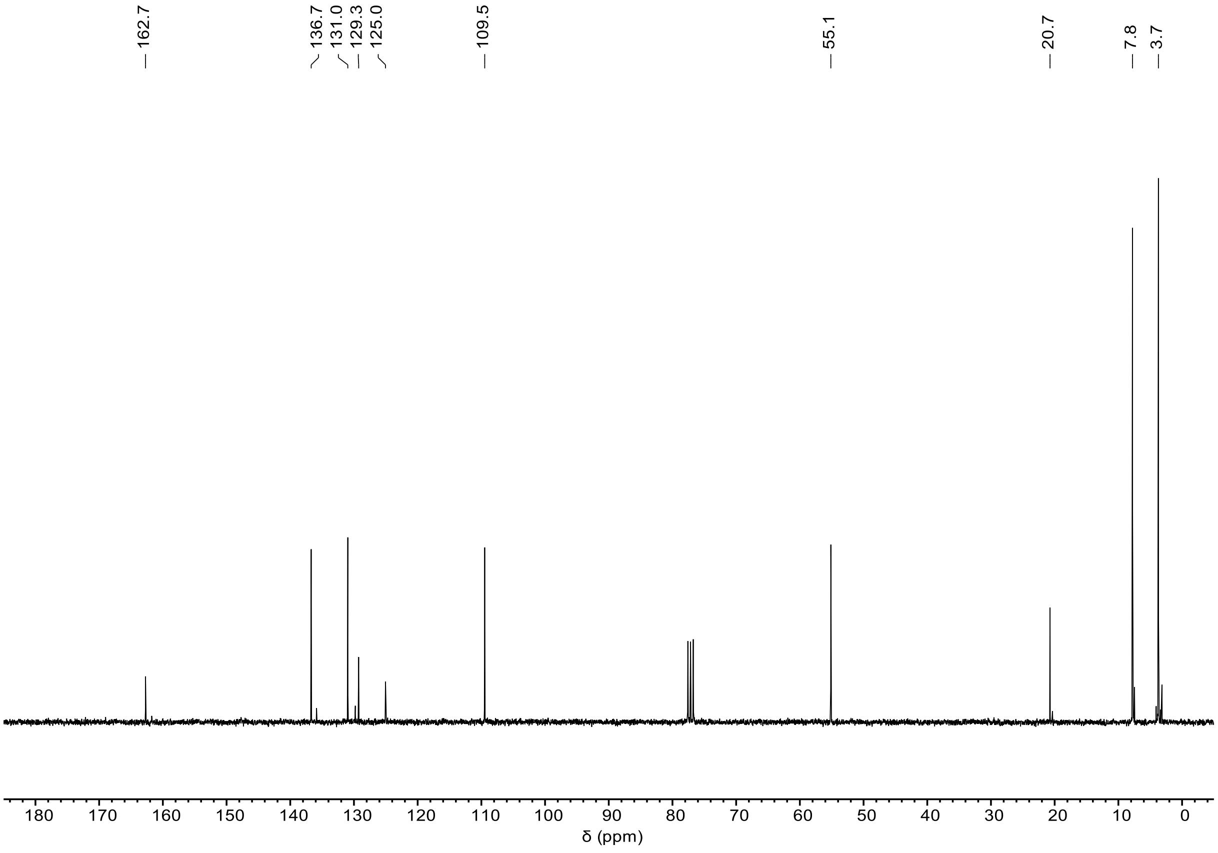

Figure S39. $^{13}C\{^{1}H\}$ NMR of **2c** in $CDCl_3$.

Triethyl(phenyl)silane (2d)

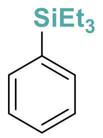

$C_{12}H_{20}Si$
Molecular Weight: 192.38

Prepared according to General Procedure A using benzene as solvent and purified by flash column chromatography in silica gel and hexane, compound **2d** was isolated as a colorless oil (87 mg, 91 % yield). Spectroscopic data are in accordance with literature reports.[9]

^{1}H NMR (300 MHz, $CDCl_3$): *δ* 7.60 – 7.52 (m, 2H), 7.45 – 7.35 (m, 3H), 1.10 – 0.99 (m, 9H), 0.93 – 0.81 (m, 6H).

^{13}C{^{1}H} NMR (75 MHz, $CDCl_3$): *δ* 137.6, 134.3, 128.8, 127.8, 7.5, 3.5.

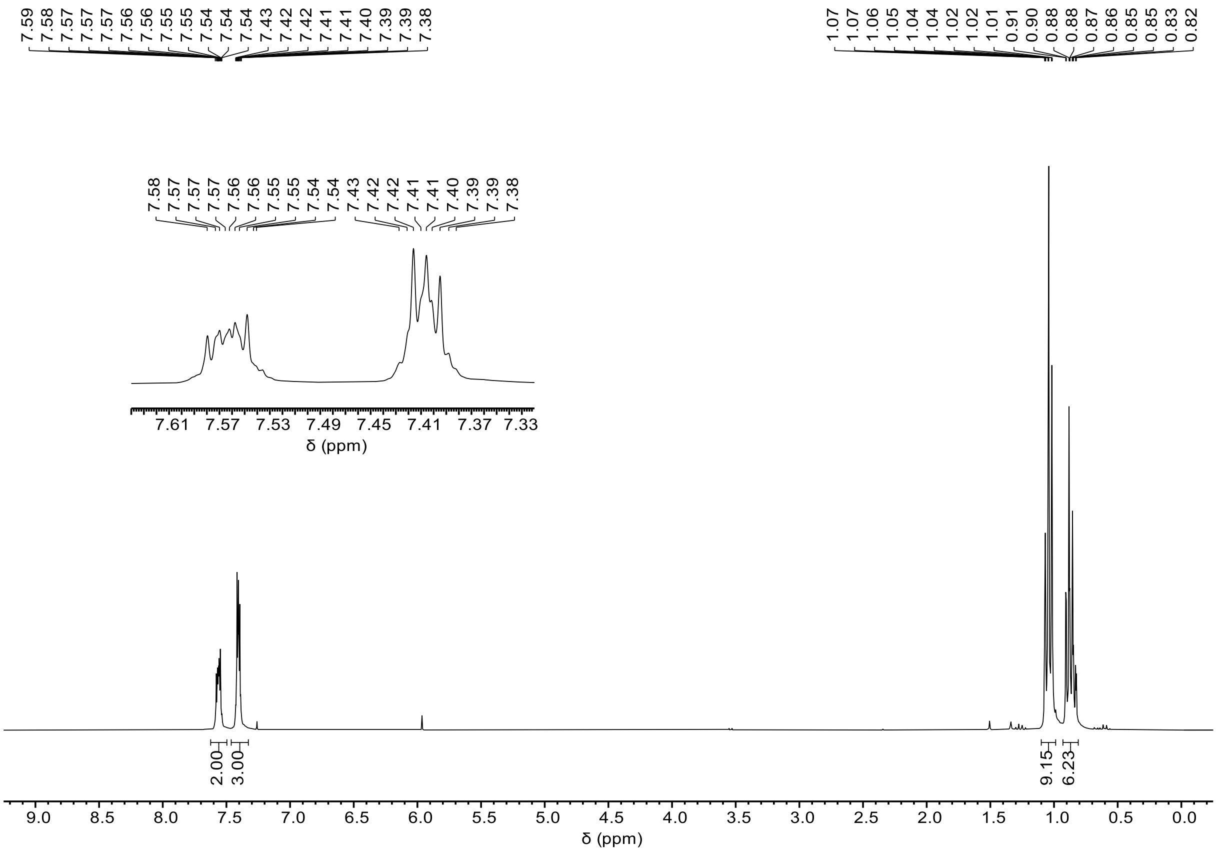

Figure S40. ^{1}H NMR of **2d** in $CDCl_3$.

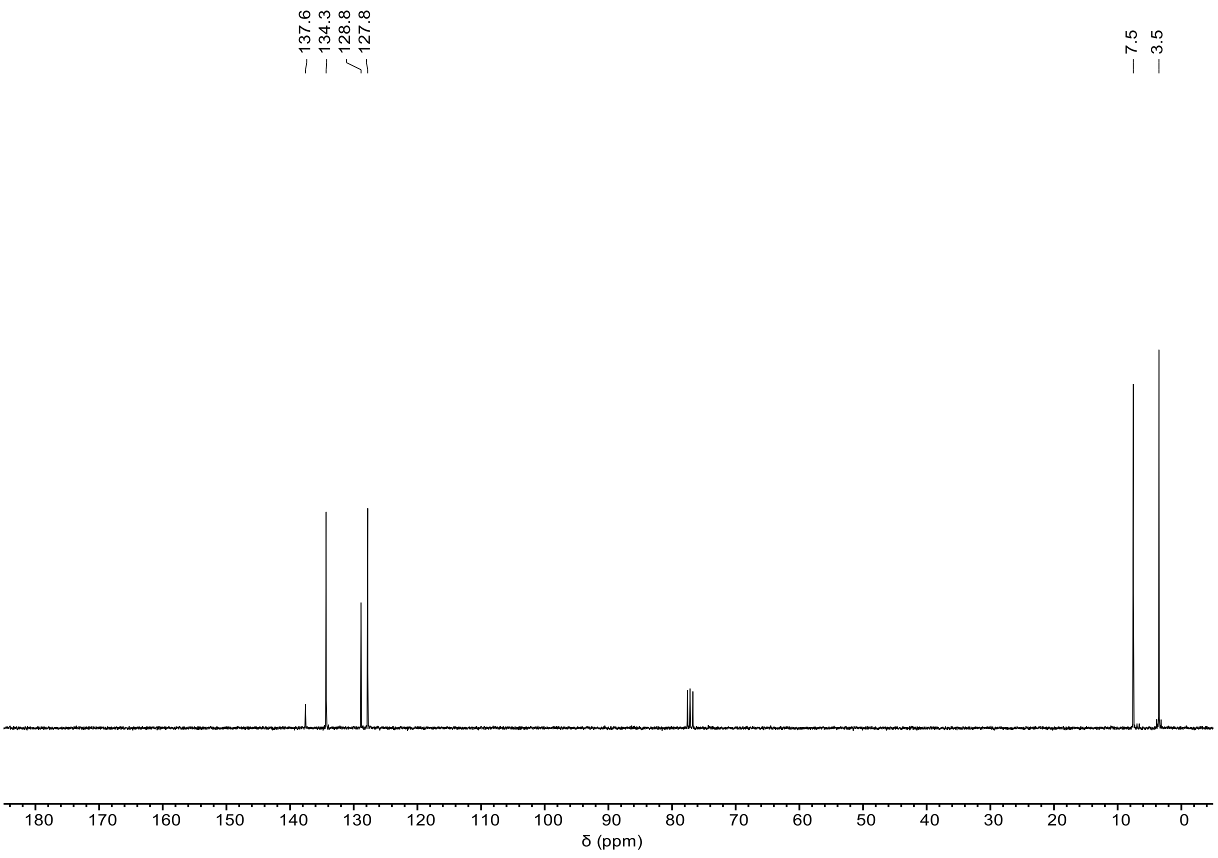

Figure S41. $^{13}C\{^1H\}$ NMR of **2d** in $CDCl_3$.

Triethyl(naphthalen-2-yl)silane (2e)

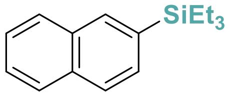

$C_{16}H_{22}Si$
Molecular Weight: 242.44

Prepared according to General Procedure A using 1 mmol (2 eq.) of substrate and purified by flash column chromatography in silica gel and hexane, compound **2e** was isolated as a colorless oil (86 mg, 71 % yield). Spectroscopic data are in accordance with literature reports.[16]

^{1}H NMR (300 MHz, $CDCl_3$): δ 7.99 (s, 1H), 7.86 – 7.78 (m, 3H), 7.57 (d, J = 8.1, 1.1 Hz, 1H), 7.49 – 7.43 (m, 2H), 1.04 – 0.97 (m, 9 H), 0.94 – 0.83 (m, 6H).

^{13}C{^{1}H} NMR (75 MHz, $CDCl_3$): δ 135.1, 135.0, 133.8, 133.1, 130.7, 128.2, 127.8, 126.9, 126.3, 125.9, 7.6, 3.6.

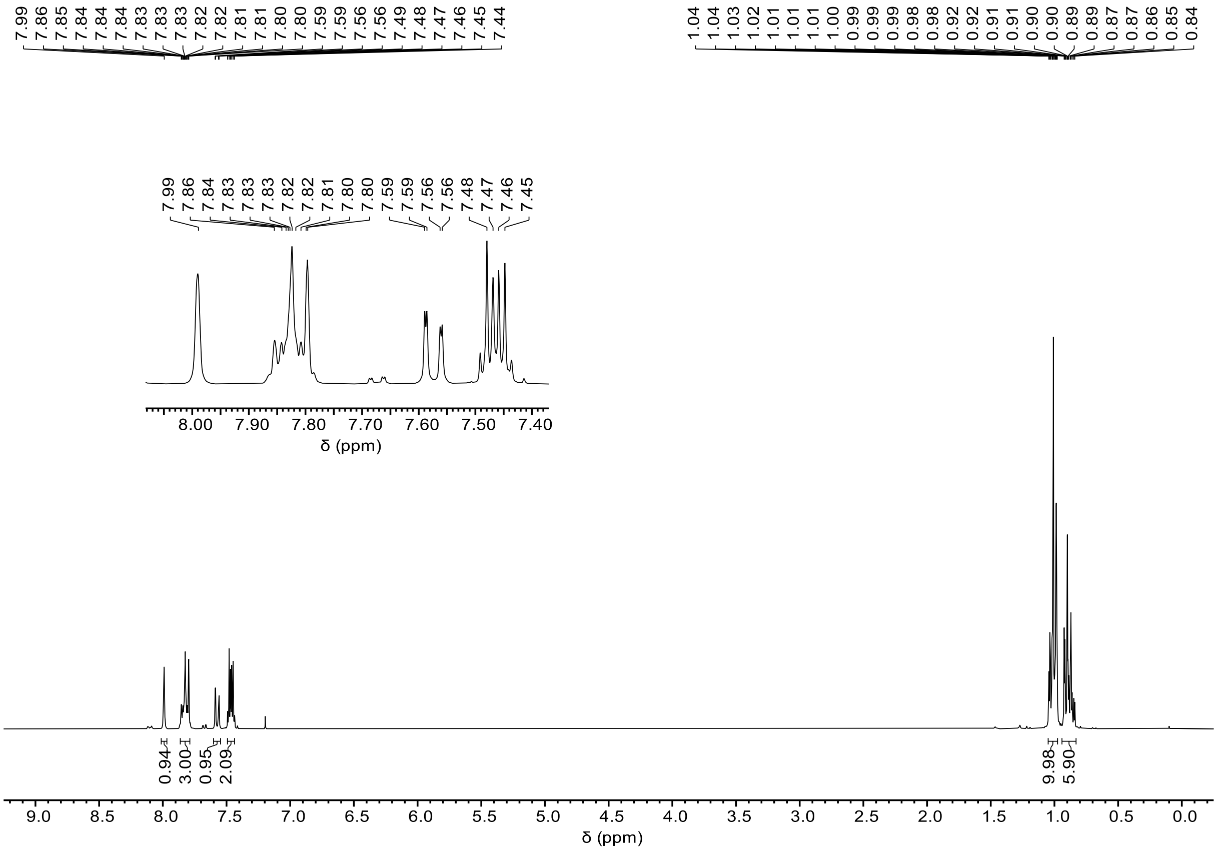

Figure S42. ^{1}H NMR of **2e** in $CDCl_3$.

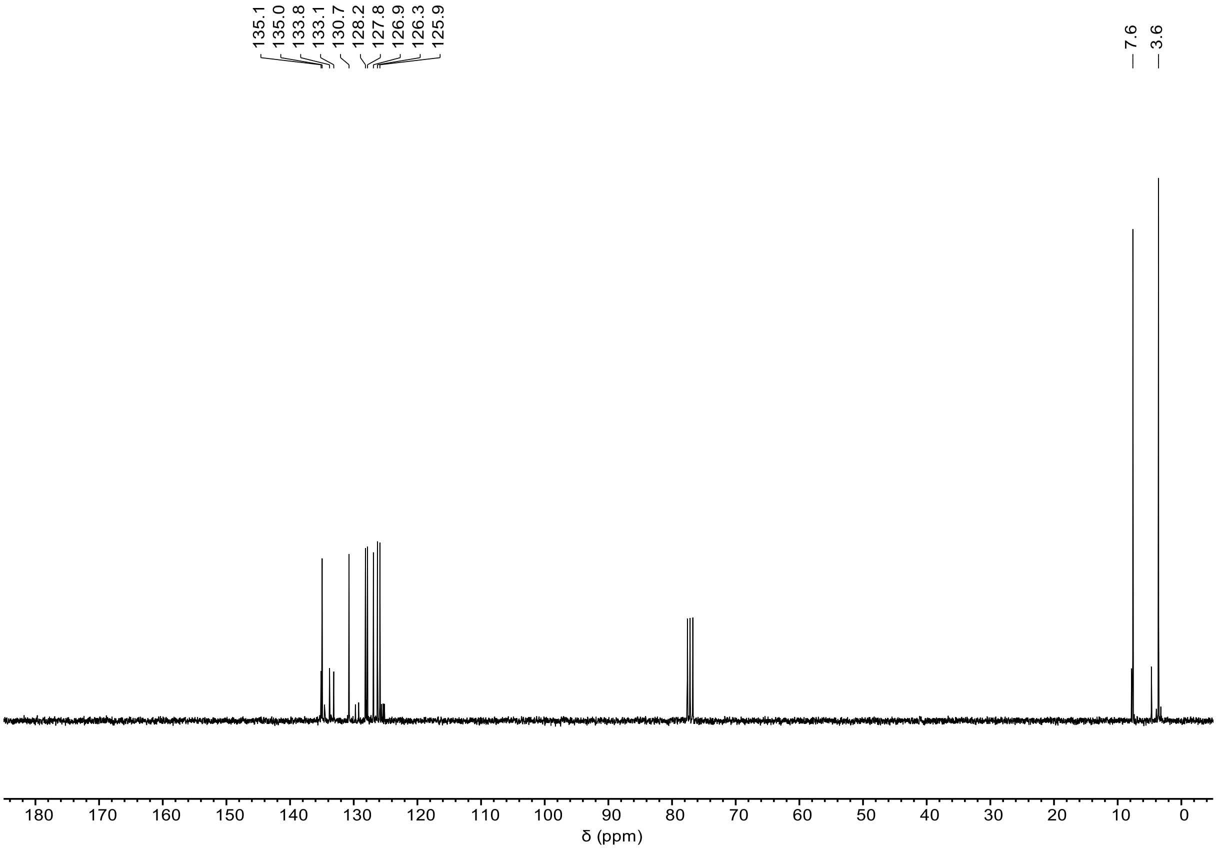

Figure S43. $^{13}C\{^{1}H\}$ NMR of **2e** in $CDCl_3$.

***N*,*N*-Diisopropyl-2-(triethylsilyl)benzamide (2f)**

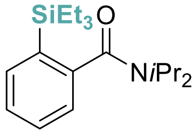

$C_{19}H_{33}NOSi$
Molecular Weight: 319.56

Prepared according to General Procedure A and purified by flash column chromatography in silica gel and hexane/EtOAc (95:5), compound **2f** was isolated as a white solid (115 mg, 72 % yield). Spectroscopic data are in accordance with literature reports.[9]

^{1}H NMR (300 MHz, $CDCl_3$): *δ* 7.60 – 7.53 (m, 1H), 7.35 – 7.27 (m, 2H), 7.19 – 7.12 (m, 1H), 3.78 (hept, *J* = 6.8 Hz, 1H), 3.49 (hept, *J* = 6.8 Hz, 1H), 1.56 (d, *J* = 6.7 Hz, 6H), 1.15 (d, *J* = 6.7 Hz, 6H), 1.00 – 0.77 (m, 15H).

^{13}C{^{1}H} NMR (75 MHz, $CDCl_3$): *δ* 172.3, 144.9, 136.2, 135.2, 128.1, 127.4, 125.5, 50.9, 45.9, 20.7, 7.6, 3.8.

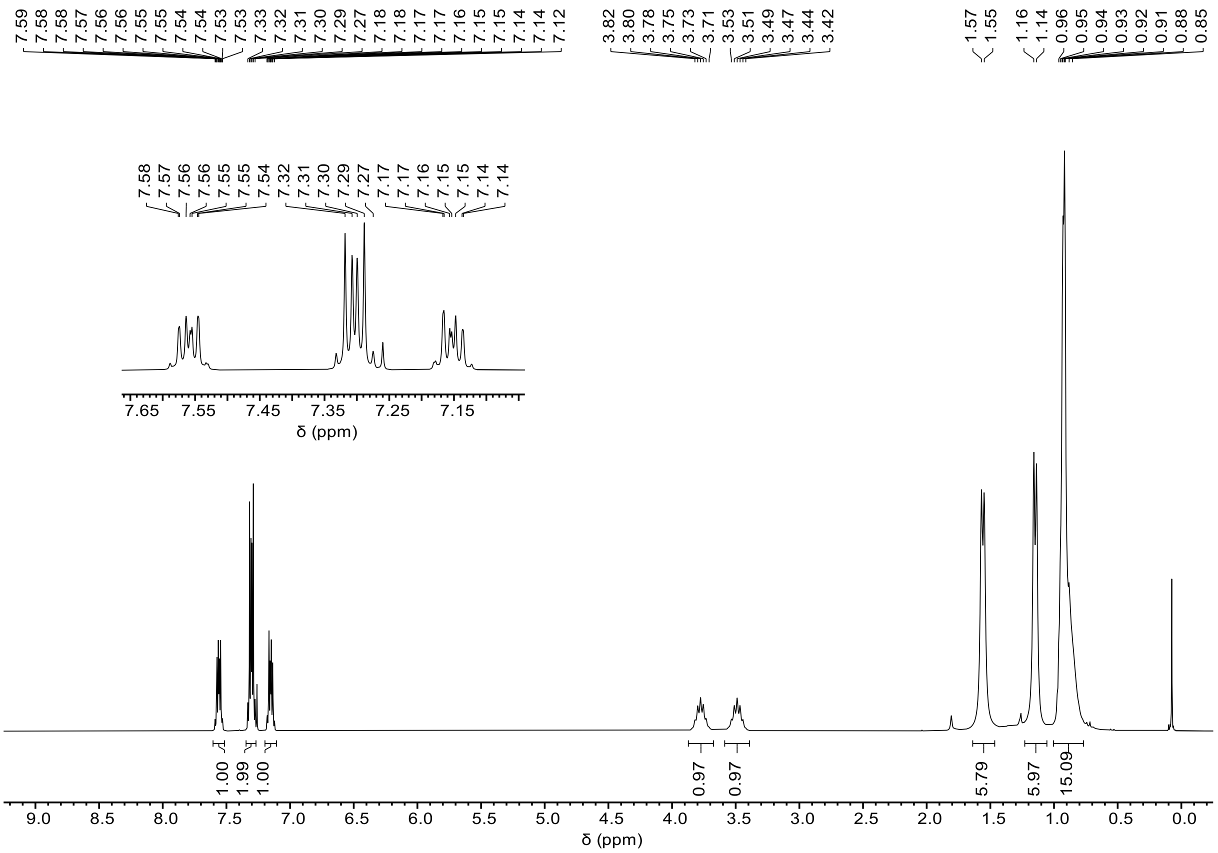

Figure S44. ^{1}H NMR of **2f** in $CDCl_3$.

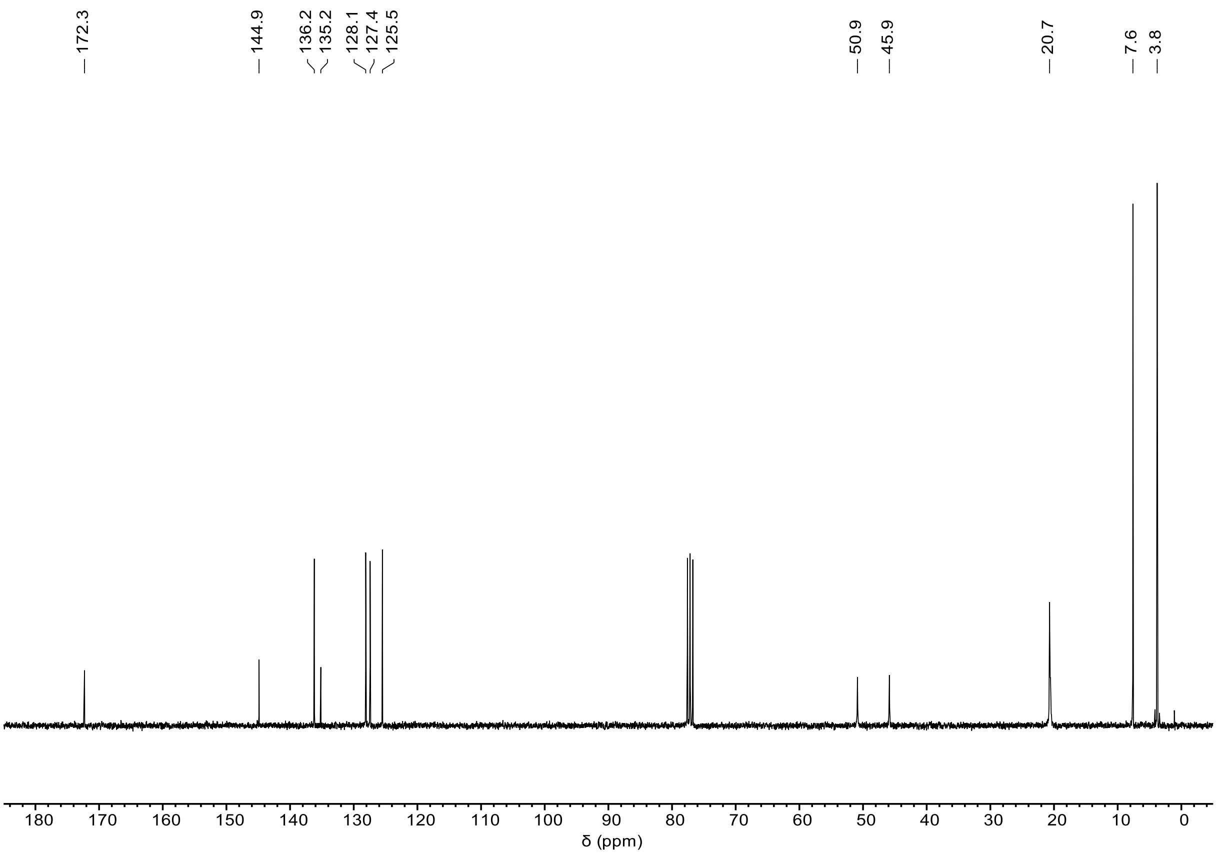

Figure S45. $^{13}C\{^{1}H\}$ NMR of **2f** in $CDCl_3$.

Triethylsilylferrocene (2g)

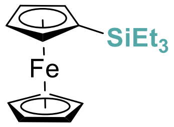

$C_{16}H_{24}FeSi$
Molecular Weight: 300.30

Prepared according to General Procedure A using 0.75 mmol (1.5 eq.) of substrate and purified by flash column chromatography in silica gel and hexane, compound **2g** was isolated as an orange oil (93 mg, 62 % yield).

^{1}H NMR (300 MHz, $CDCl_3$): *δ* 4.36 – 4.33 (m, 2H), 4.15 (s, 5H), 4.12 – 4.09 (m, 2H), 1.10 – 1.00 (m, 9H), 0.83 – 0.71 (m, 6H).

^{13}C{^{1}H} NMR (75 MHz, $CDCl_3$): *δ* 73.4, 70.5, 69.7, 68.3, 7.9, 4.8.

HRMS (EI) m/z: $[M]^+$ Calculated for $C_{16}H_{24}FeSi$ 300.0991. Found 300.0995.

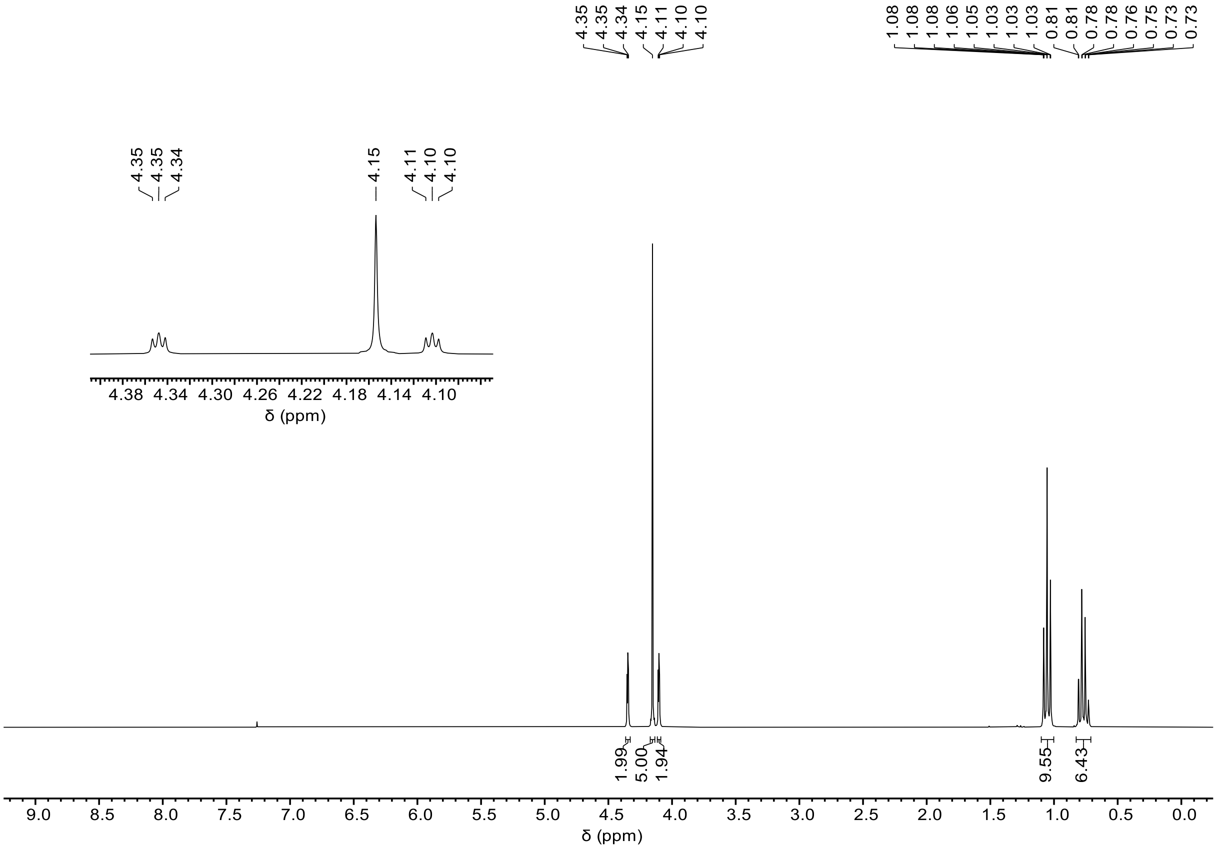

Figure S46. ^{1}H NMR of **2g** in $CDCl_3$.

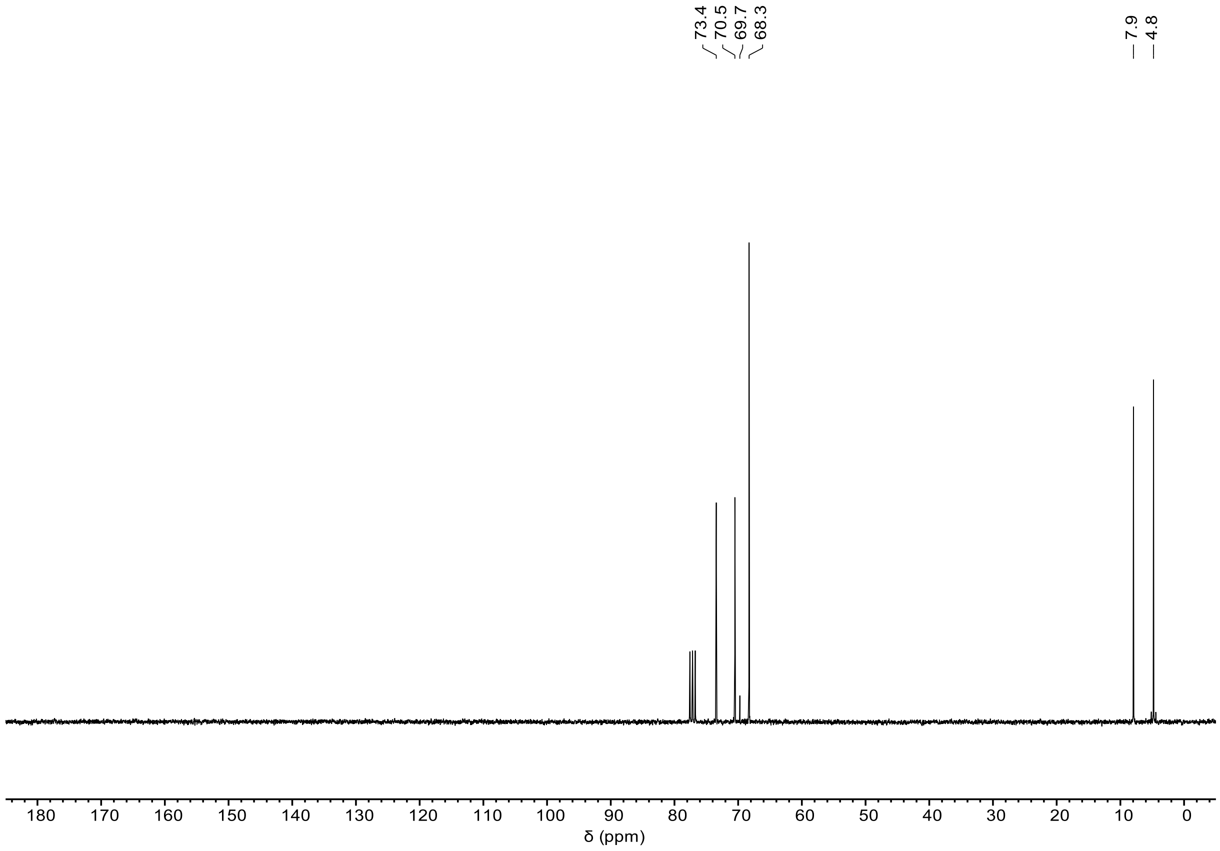

Figure S47. $^{13}C\{^{1}H\}$ NMR of **2f** in $CDCl_3$.

1-Methyl-2-(triethylsilyl)-1*H*-indole (3a)

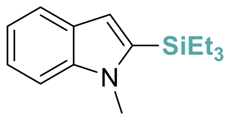

$C_{15}H_{23}NSi$
Molecular Weight: 245.44

Prepared according to General Procedure A and purified by flash column chromatography in silica gel and hexane/EtOAc (95:5), compound **3a** was isolated as a colorless oil (113 mg, 92 % yield). Spectroscopic data are in accordance with literature reports.[17]

^{1}H NMR (300 MHz, $CDCl_3$): δ 7.67 (dt, J = 7.9, 0.9 Hz, 1H), 7.36 (dq, J = 8.2, 0.7 Hz, 1H), 7.26 (ddd, J = 8.1, 6.9, 1.2 Hz, 1H), 7.13 (ddd, J = 7.9, 7.0, 1.0 Hz, 1H), 6.78 (d, J = 0.7 Hz, 1H), 3.86 (s, 3H), 1.10 – 1.02 (m, 9H), 1.00 – 0.91 (m, 6H).

^{13}C{^{1}H} NMR (75 MHz, $CDCl_3$): δ 140.3, 138.3, 128.7, 121.9, 120.7, 119.1, 113.1, 109.1, 33.0, 7.7, 4.1.

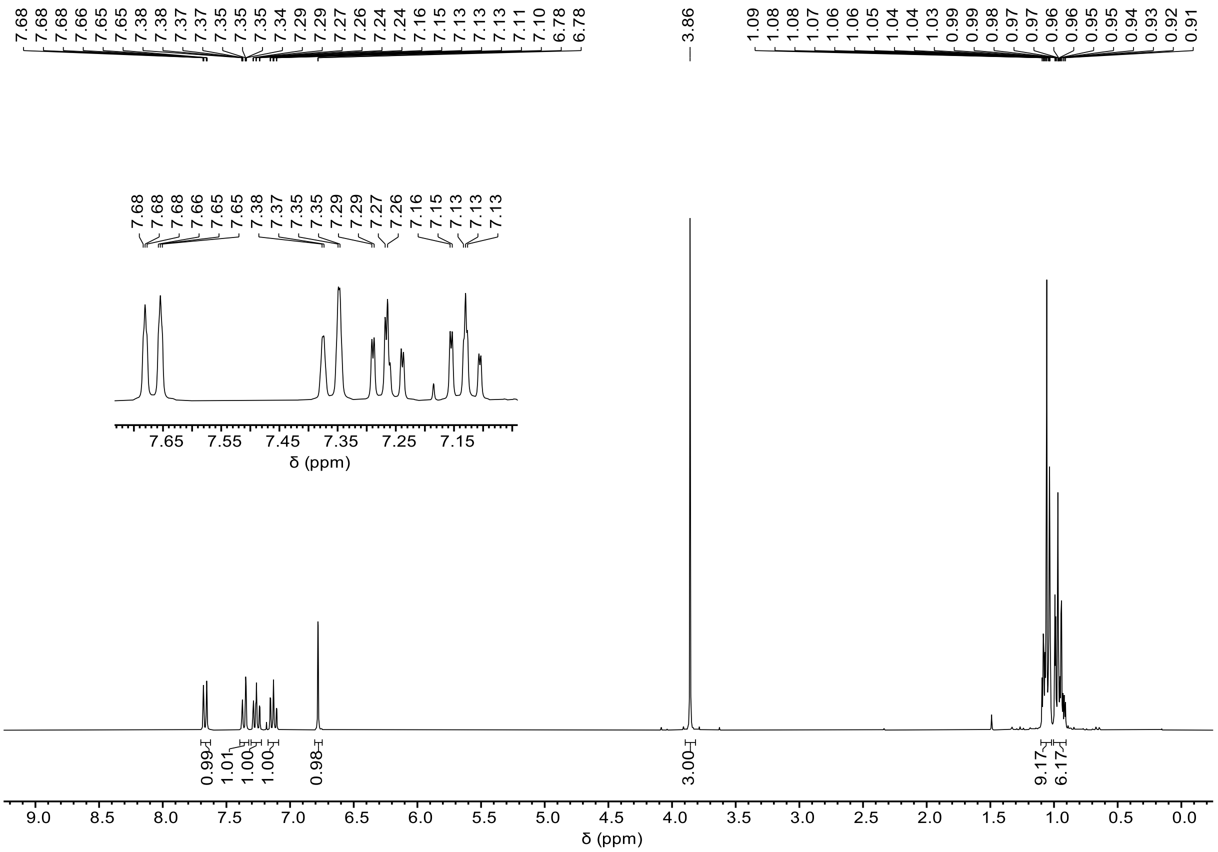

Figure S48. ^{1}H NMR of **3a** in $CDCl_3$.

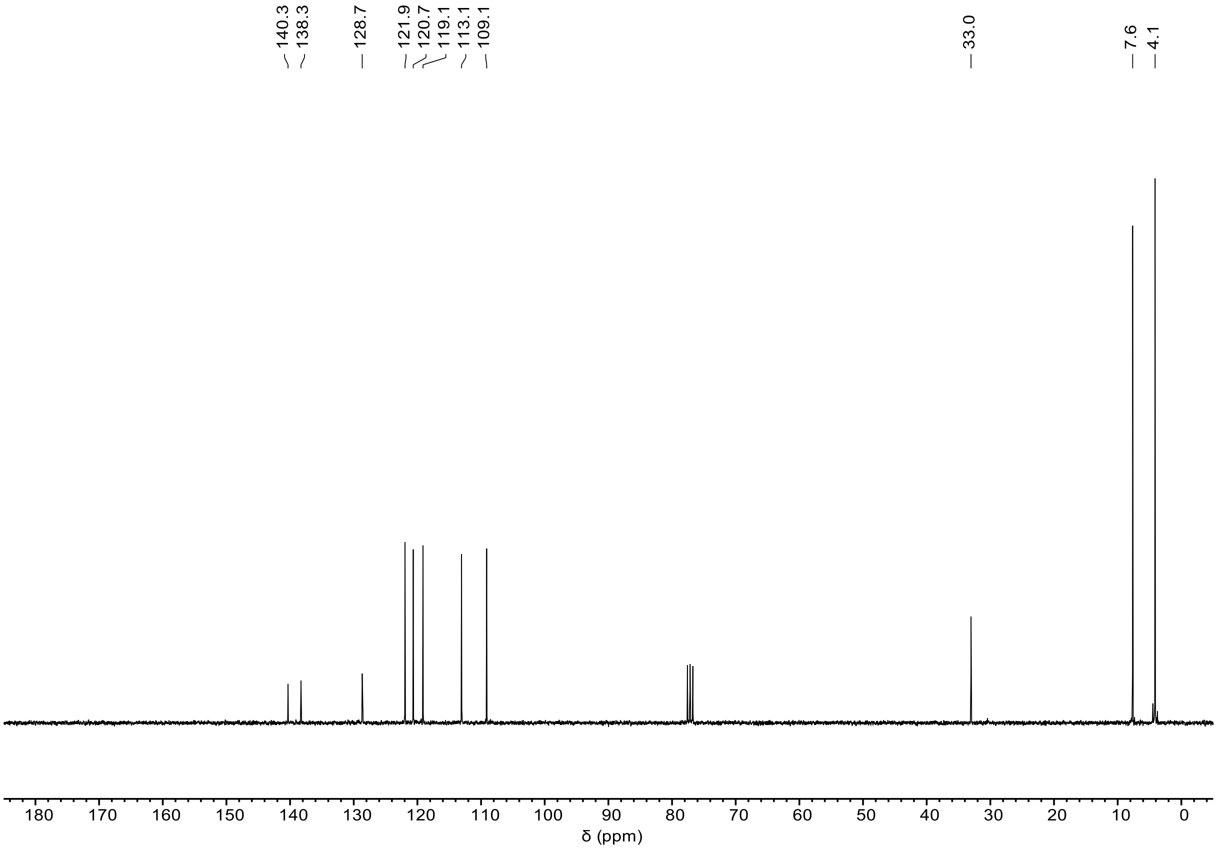

Figure S49. $^{13}C\{^1H\}$ NMR of **3a** in $CDCl_3$.

Benzo[*b*]furan-2-yltriethylsilane (3b)

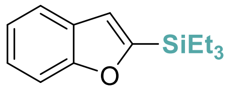

$C_{14}H_{20}OSi$
Molecular Weight: 232.40

Prepared according to General Procedure A and purified by flash column chromatography in silica gel and hexane, compound **3b** was isolated as a colorless oil (106 mg, 91 % yield). Spectroscopic data are in accordance with literature reports.[17]

^{1}H NMR (300 MHz, $CDCl_3$): δ 7.58 (ddd, *J* = 7.3, 1.3, 0.6 Hz, 1H), 7.51 (dq, *J* = 7.9, 0.6 Hz, 1H), 7.27 (dt, *J* = 7.2, 1.4 Hz, 1H), 7.19 (dt, *J* = 7.6, 1.2 Hz, 1H), 6.99 (d, *J* = 0.9, 1H), 1.09 – 1.00 (m, 9H), 0.91 – 0.81 (m, 6H).

^{13}C{^{1}H} NMR (75 MHz, $CDCl_3$): δ 161.8, 158.3, 128.1, 124.3, 122.4, 121.0, 117.3, 111.4, 7.5, 3.3.

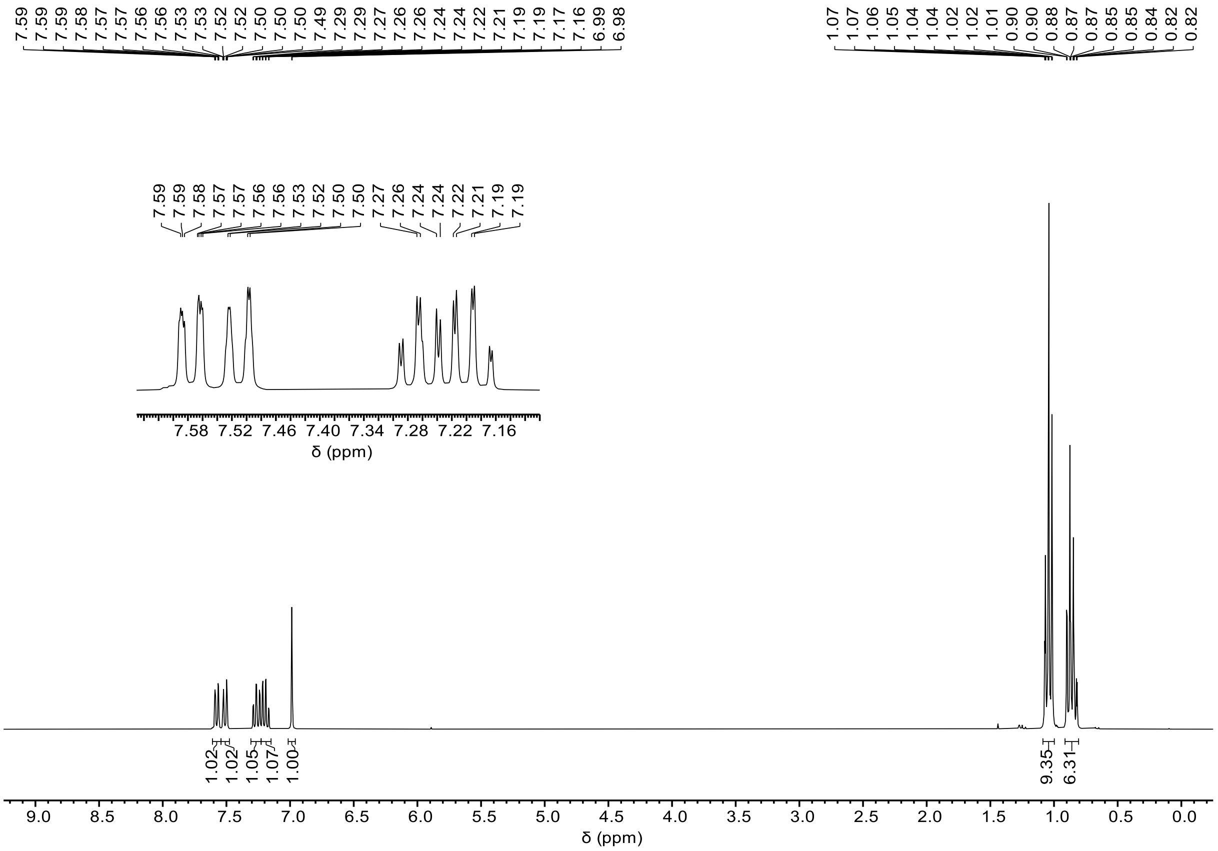

Figure S50. ^{1}H NMR of **3b** in $CDCl_3$.

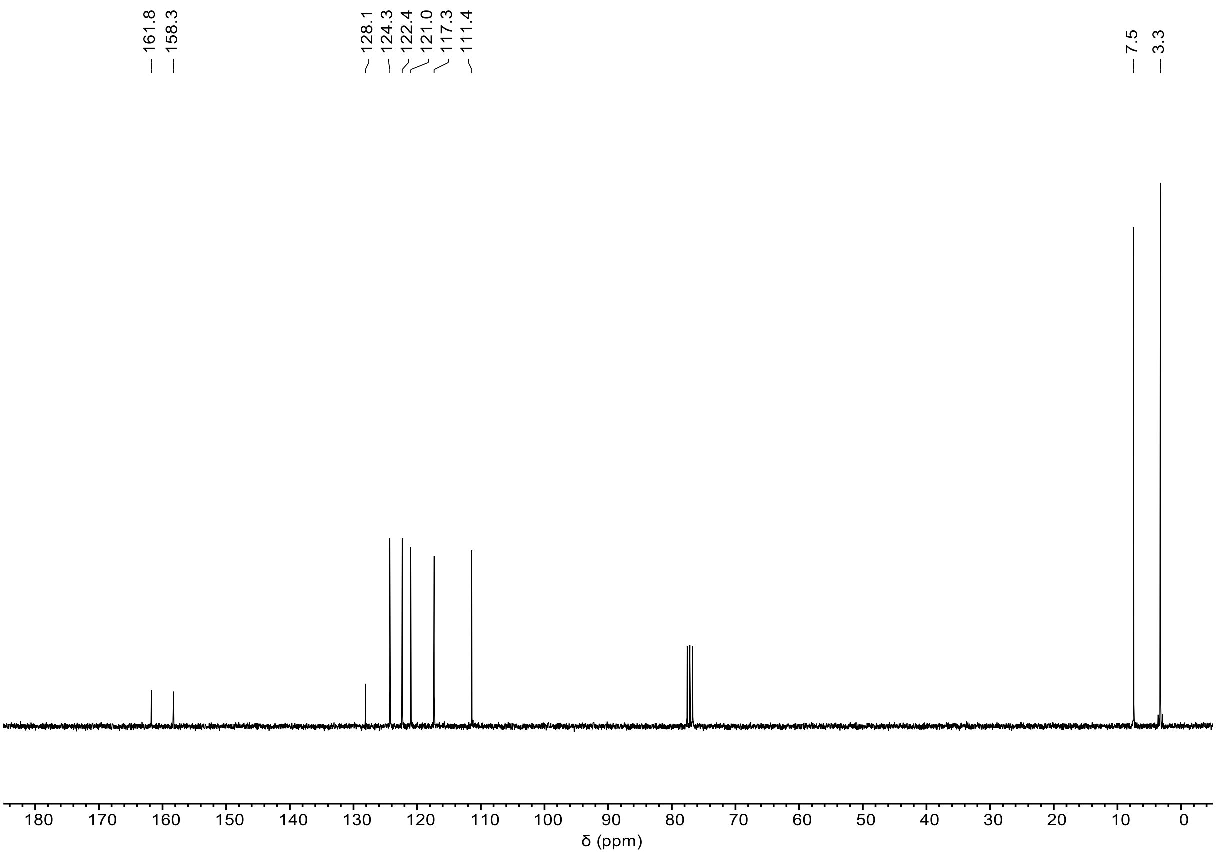

Figure S51. $^{13}C\{^1H\}$ NMR of **3b** in $CDCl_3$.

Benzo[*b*]thiophen-2-yltriethylsilane (3c)

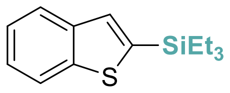

$C_{14}H_{20}SSi$
Molecular Weight: 248.46

Prepared according to General Procedure A and purified by flash column chromatography in silica gel and hexane, compound **3c** was isolated as a colorless oil (108 mg, 87 % yield). Spectroscopic data are in accordance with literature reports.[17]

^{1}H NMR (300 MHz, $CDCl_3$): *δ* 7.93 – 7.86 (m, 1H), 7.85 – 7.78 (m, 1H), 7.48 (d, *J* = 0.7 Hz, 1H), 7.34 (td, *J* = 7.2, 1.5 Hz, 1H), 7.29 (td, *J* = 7.1, 1.6 Hz, 1H), 1.10 – 1.00 (m, 9H), 0.94 – 0.83 (m, 6H).

^{13}C{^{1}H} NMR (75 MHz, $CDCl_3$): *δ* 143.8, 141.2, 139.1, 131.7, 124.2, 124.0, 123.5, 122.2, 7.5, 4.4.

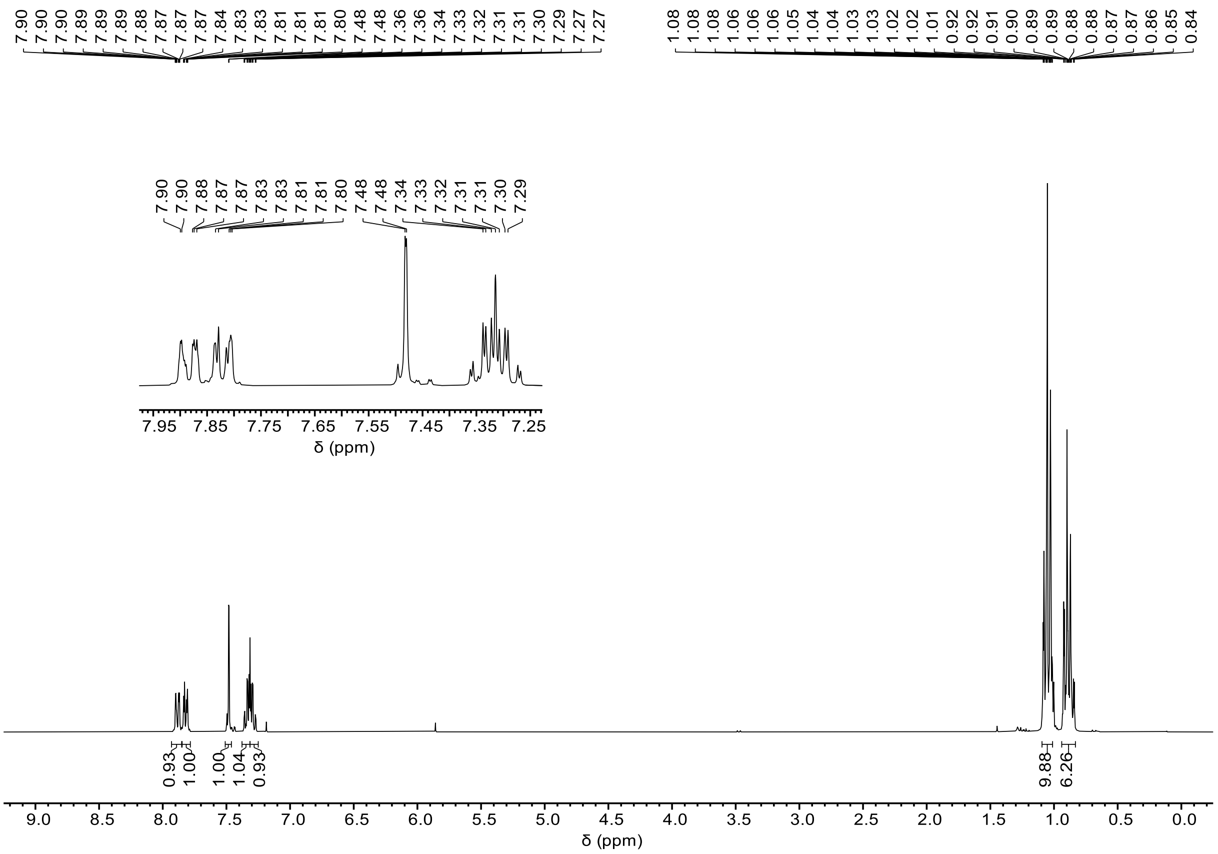

Figure S52. ^{1}H NMR of **3c** in $CDCl_3$.

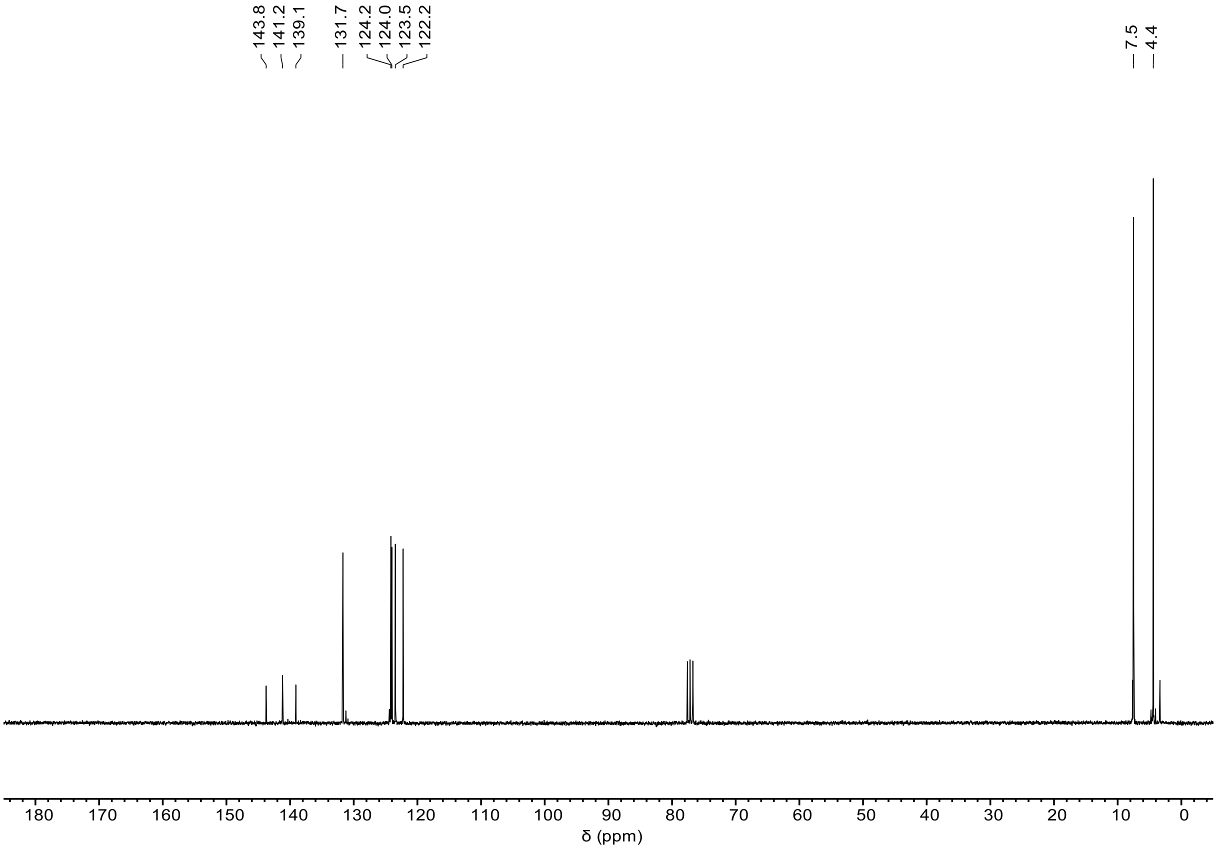

Figure S53. $^{13}C\{^1H\}$ NMR of **3c** in $CDCl_3$.

***N-tert*-Butyl-1-(1-methyl-2-(triethylsilyl)-1*H*-indol-3-yl)methanimine (3d)**

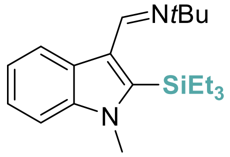

$C_{20}H_{32}N_2Si$
Molecular Weight: 328.58

Prepared according to General Procedure A. NMR yield for **3d** (83%) was measured by adding 1,1,2,2-tetrachloroethane (26.4 µL, 0.25 mmol) and comparing the corresponding signal in the ^{1}H NMR spectrum.

^{1}H NMR (300 MHz, $CDCl_3$): δ 8.83 (s, 1H), 8.78 (dt, J = 7.8, 1.1 Hz, 1H), 7.41 – 7.36 (m, 2H), 7.34 – 7.27 (m, 1H), 3.94 (s, 3H), 1.47 (s, 9H), 1.17 – 1.07 (m, 15H).

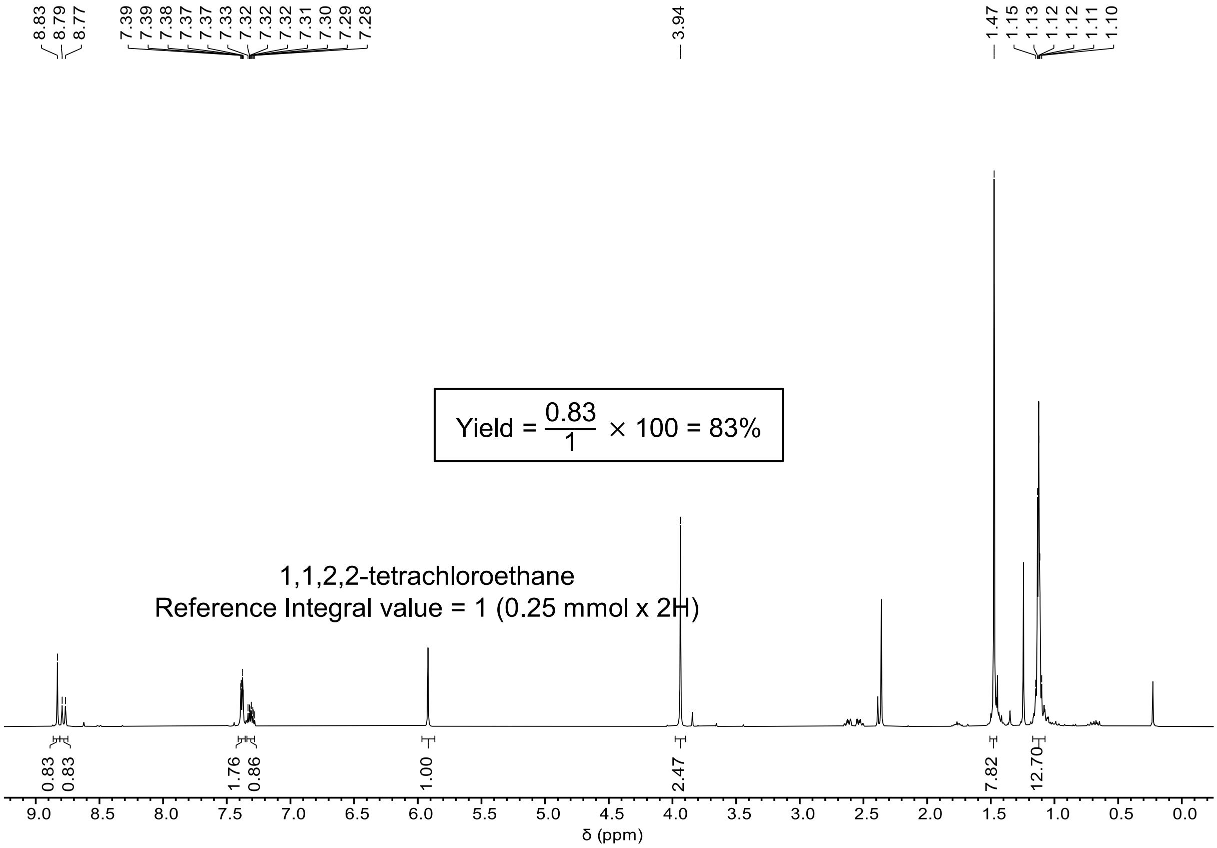

Figure S54. ^{1}H NMR of **3d** (not isolated) in $CDCl_3$.

Triethyl(5-methylbenzo[*b*]thiophen-2-yl)silane (3e)

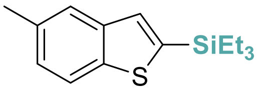

$C_{15}H_{22}SSi$
Molecular Weight: 262.49

Prepared according to General Procedure A. NMR yield for **3e** (96%) was measured by adding 1,1,2,2-tetrachloroethane (26.4 μL, 0.25 mmol) and comparing the corresponding signal in the ^{1}H NMR spectrum.

^{1}H NMR (300 MHz, $CDCl_3$): δ 7.81 (d, J = 8.2 Hz, 1H), 7.67 (s, 1H), 7.46 (s, 1H), 7.19 (d, J = 8.2 Hz, 1H), 2.53 (s, 3H), 1.11 (t, J = 7.4 Hz, 9H), 0.93 (q, J = 7.4 Hz, 6H).

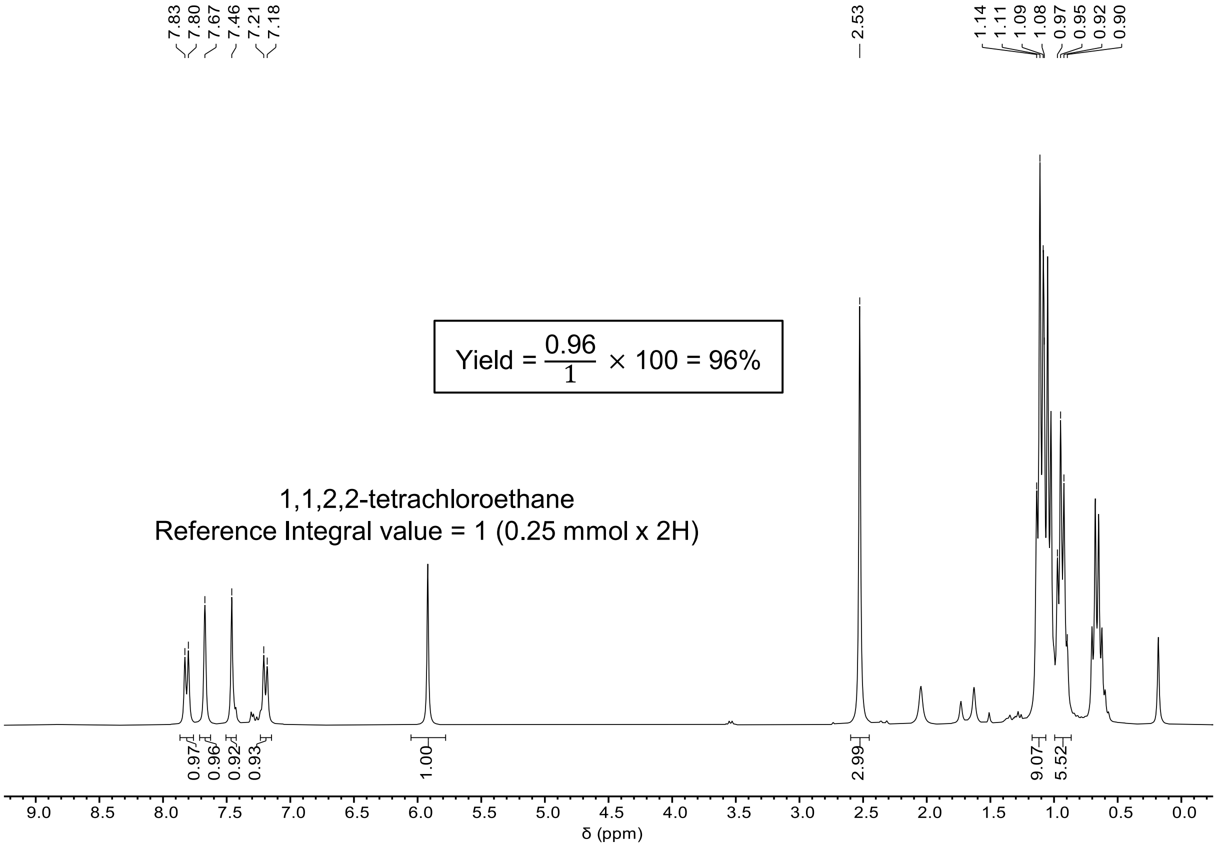

Figure S55. ^{1}H NMR of **3e** (not isolated) in $CDCl_3$.

1-Methyl-2-(triethylsilyl)-1*H*-benzo[*d*]imidazole (3f)

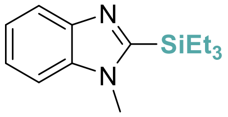

$C_{14}H_{22}N_2Si$
Molecular Weight: 246.43

Prepared according to General Procedure A. NMR yield for **3f** (33%) was measured by adding 1,1,2,2-tetrachloroethane (26.4 μL, 0.25 mmol) and comparing the corresponding signal in the ^{1}H NMR spectrum.

^{1}H NMR (300 MHz, $CDCl_3$): δ 7.91 – 7.76 (m, 1H), 7.38 – 7.29 (m, 2H), 7.28 – 7.18 (m, 1H), 3.76 (s, 3H), 0.97 – 0.91 (m, 9H), 0.58 – 0.49 (m, 6H).

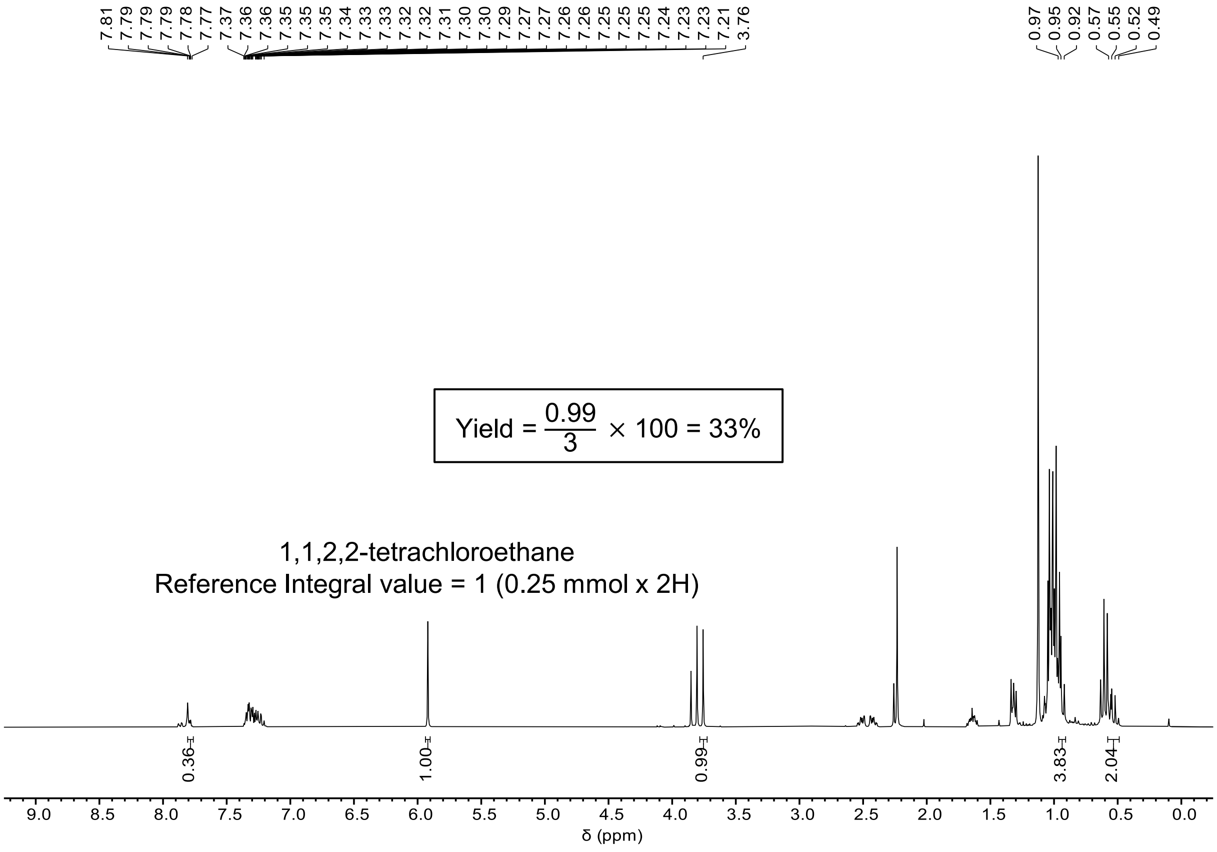

Figure S56. ^{1}H NMR of **3f** (not isolated) in $CDCl_3$.

2-(Triethylsilyl)benzo[*d*]oxazole (3g)

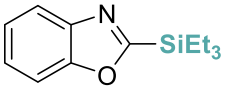

$C_{13}H_{19}NOSi$
Molecular Weight: 233.39

Prepared according to General Procedure A. NMR yield for **3g** (76%) was measured by adding 1,1,2,2-tetrachloroethane (26.4 µL, 0.25 mmol) and comparing the corresponding signal in the ^{1}H NMR spectrum.

^{1}H NMR (300 MHz, $CDCl_3$): *δ* 7.29 – 7.19 (m, 2H), 6.93 – 6.82 (m, 2H), 1.04 – 0.95 (m, 9H), 0.83 – 0.72 (m, 6H).

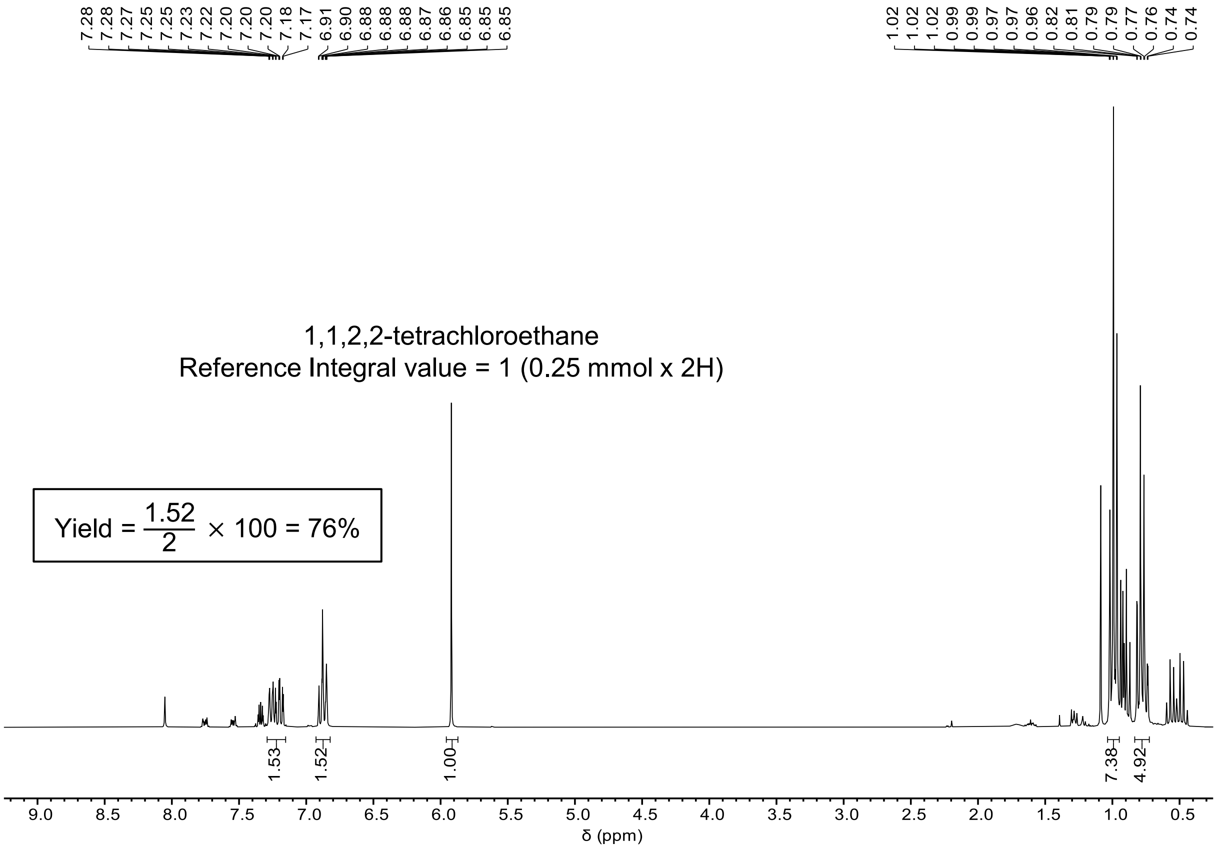

Figure S57. ^{1}H NMR of **3g** (not isolated) in $CDCl_3$.

2-(Triethylsilyl)benzo[*d*]thiazole (3h)

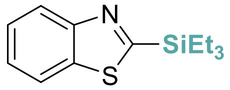

$C_{13}H_{19}NSSi$
Molecular Weight: 249.45

Prepared according to General Procedure A. NMR yield for **3h** (74%) was measured by adding 1,1,2,2-tetrachloroethane (26.4 μL, 0.25 mmol) and comparing the corresponding signal in the ^{1}H NMR spectrum. Spectroscopic data are in accordance with literature reports.[18]

^{1}H NMR (300 MHz, $CDCl_3$): *δ* 8.20 (ddd, *J* = 8.1, 1.3, 0.7 Hz, 1H), 7.96 (ddd, *J* = 7.9, 1.4, 0.6 Hz, 1H), 7.47 (ddd, *J* = 8.3, 7.1, 1.0 Hz, 1H), 7.38 (ddd, *J* = 8.0, 7.2, 1.2 Hz, 1H), 1.12 − 1.04 (m, 9H), 1.03 − 0.96 (m, 6H).

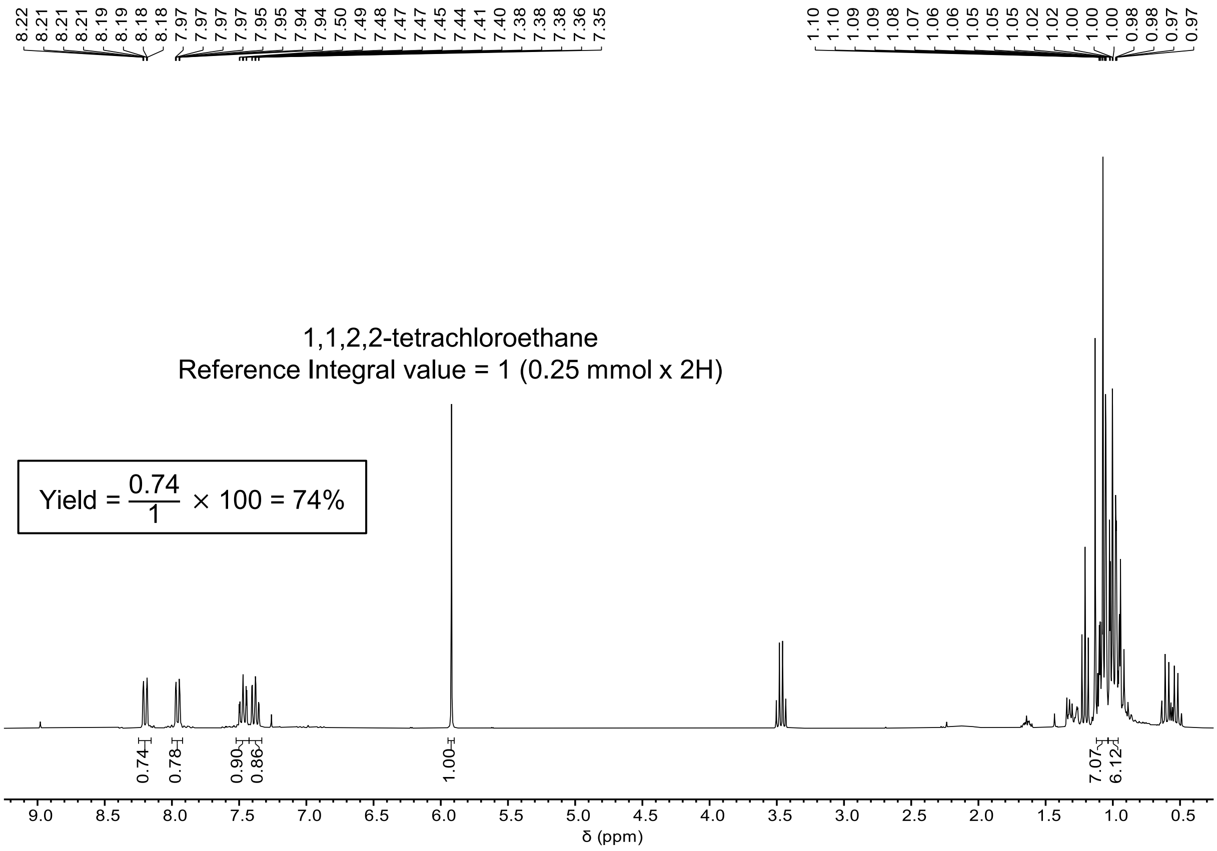

Figure S58. ^{1}H NMR of **3h** (not isolated) in $CDCl_3$.

(5-Chlorothiophen-2-yl)triethylsilane (3i)

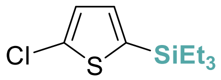

$C_{10}H_{17}ClSSi$
Molecular Weight: 232.84

Prepared according to General Procedure A and purified by flash column chromatography in silica gel and hexane, compound **3i** was isolated as a colorless oil (95 mg, 82 % yield). Spectroscopic data are in accordance with literature reports.[19]

^{1}H NMR (300 MHz, $CDCl_3$): *δ* 7.02 (d, *J* = 3.6 Hz, 1H), 6.98 (d, *J* = 3.6 Hz, 1H), 1.01, (t, *J* = 7.8 Hz, 9H), 0.80 (q, *J* = 7.8 Hz, 6H).

^{13}C{^{1}H} NMR (75 MHz, $CDCl_3$): *δ* 136.9, 134.6, 134.2, 127.5, 7.4, 4.4.

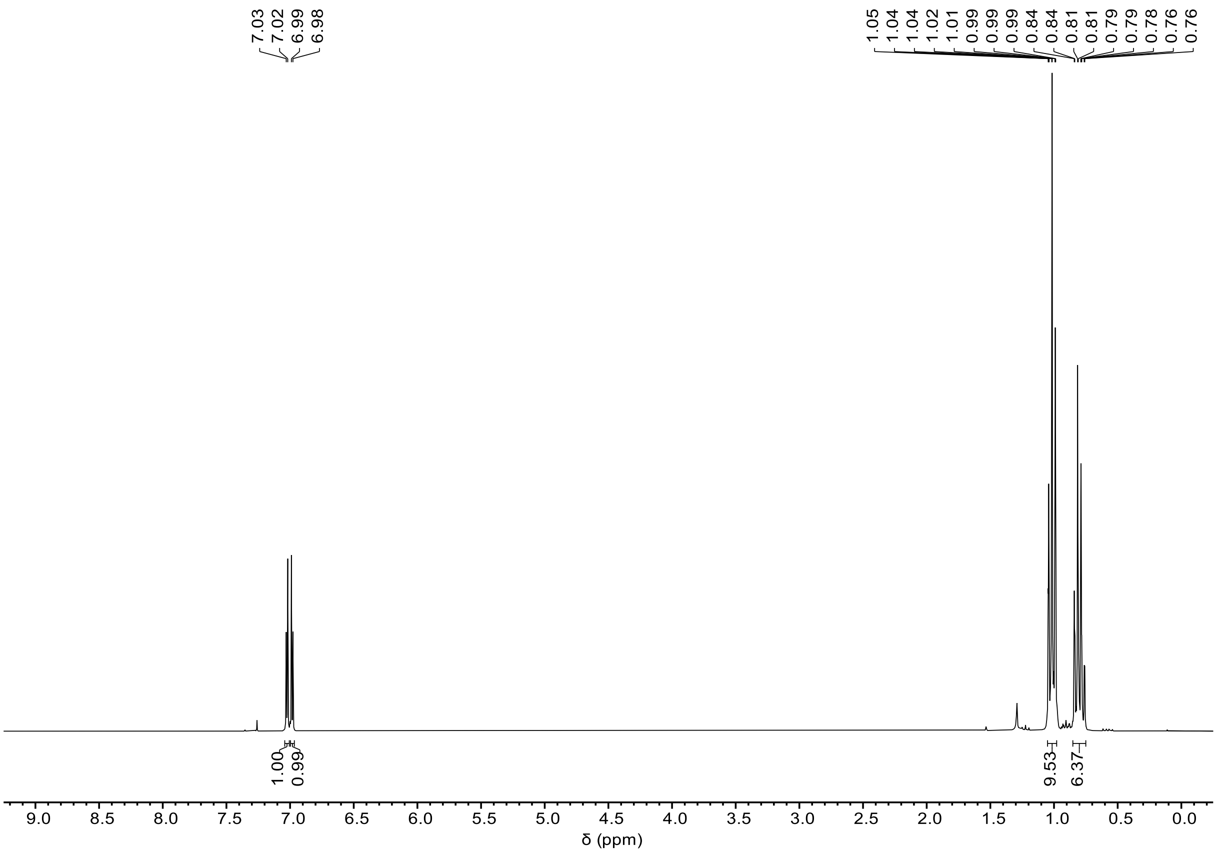

Figure S59. ^{1}H NMR of **3i** in $CDCl_3$.

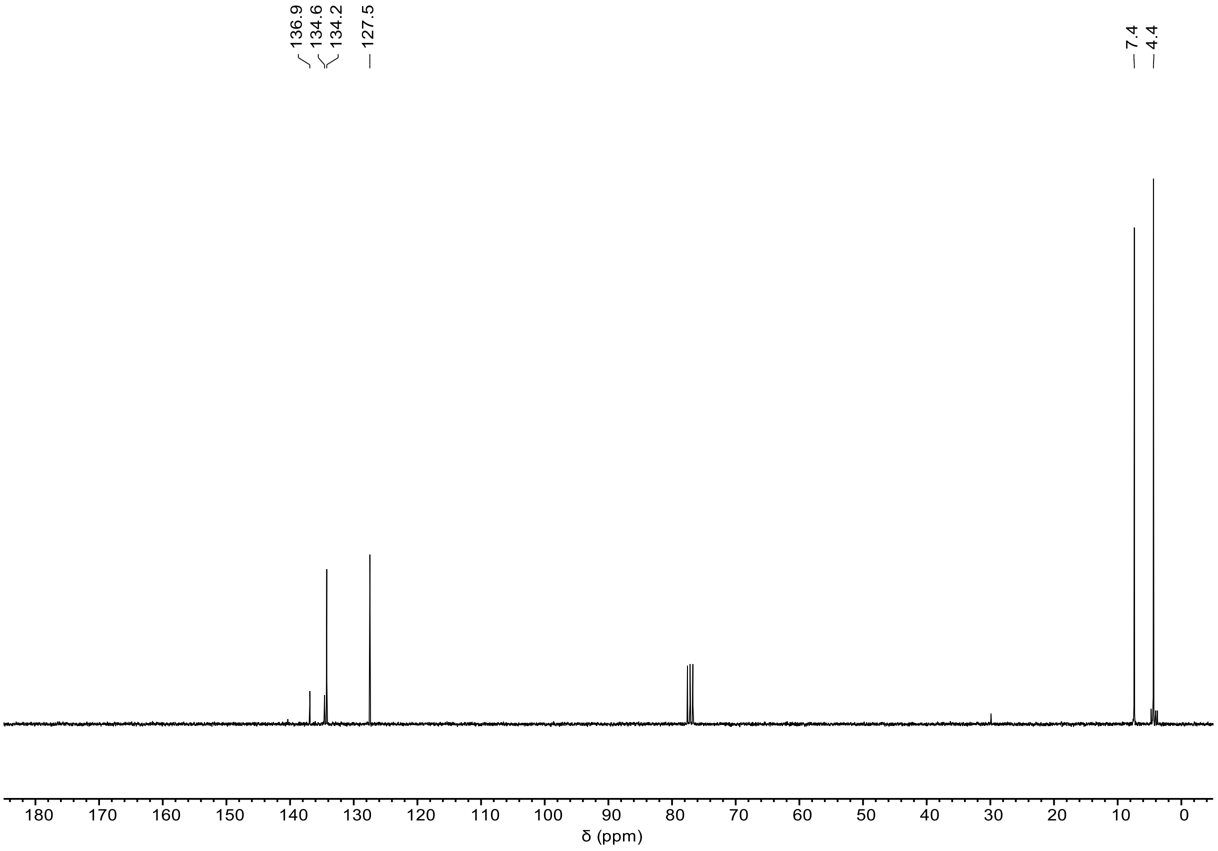

Figure S60. $^{13}C\{^{1}H\}$ NMR of **3i** in $CDCl_3$.

Triethyl(5-pentylfuran-2-yl)silane (3j)

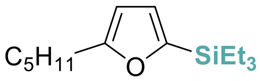

$C_{15}H_{28}OSi$
Molecular Weight: 252.47

Prepared according to General Procedure A and purified by flash column chromatography in silica gel and hexane, compound **3j** was isolated as a colorless oil (93 mg, 74 % yield). Spectroscopic data are in accordance with literature reports.[15]

^{1}H NMR (300 MHz, $CDCl_3$): δ 6.56 (d, J = 3.1 Hz, 1H), 5.98 (d, J = 3.1 Hz, 1H), 2.67 (t, J = 7.5 Hz, 2H), 1.75 – 1.60 (m, 2H), 1.42 – 1.31 (m, 4H), 1.01 (t, J = 8.0 Hz, 9H), 0.92, (t, J = 6.9 Hz, 3H), 0.77 (q, J = 8.0 Hz, 6H).

^{13}C{^{1}H} NMR (75 MHz, $CDCl_3$): δ 161.2, 156.2, 121.6, 104.7, 31.6, 28.3, 27.9, 22.6, 14.1, 7.5, 3.5.

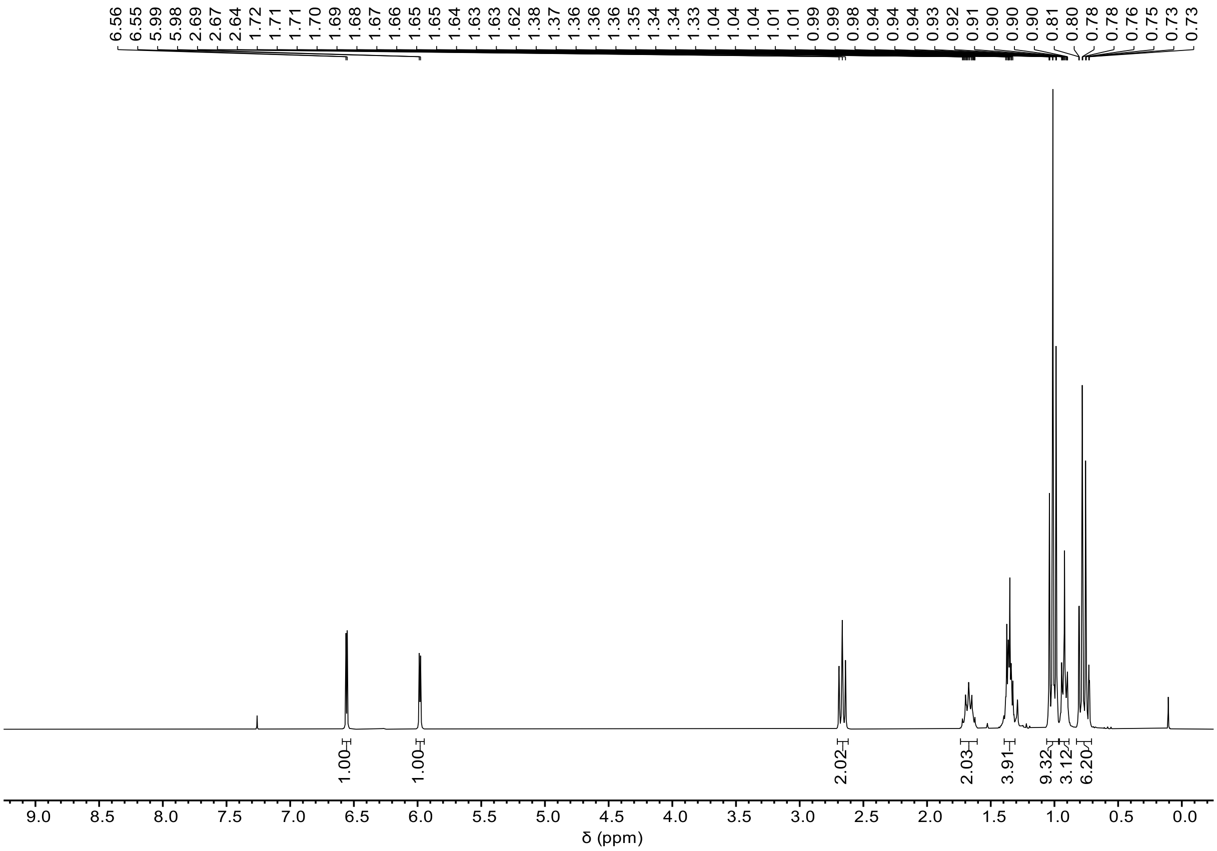

Figure S61. ^{1}H NMR of **3j** in $CDCl_3$.

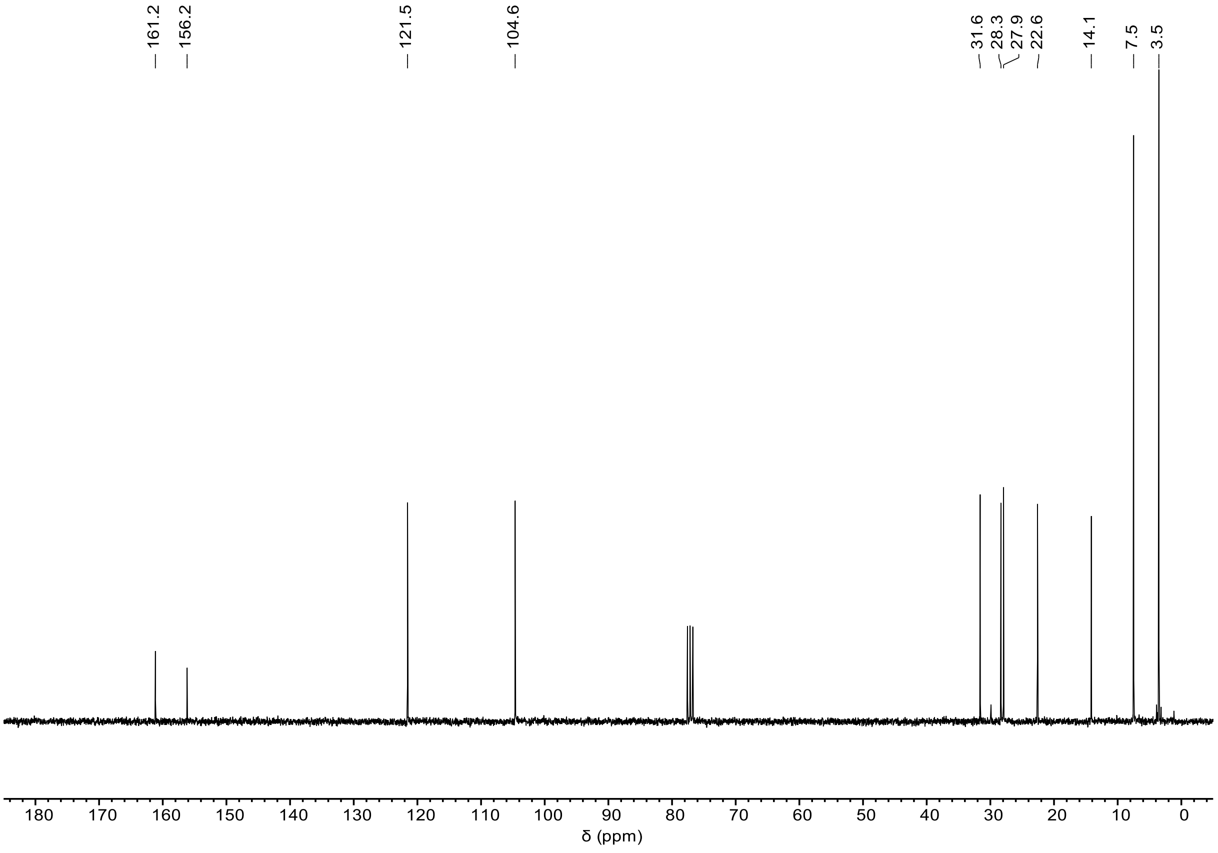

Figure S62. $^{13}C\{^{1}H\}$ NMR of **3j** in $CDCl_3$.

(Triethylsilyl)pyridine – Mixture of C2, C3 and C4-isomers (3k)

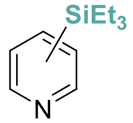

$C_{11}H_{19}NSi$
Molecular Weight: 193.36

Prepared according to General Procedure A, performing the addition of the reagents at -30 ºC and keeping the mixture at that temperature for 6 h. NMR yield for **3k** (overall 60%) as a mixture of C2 (14%),[9] C3 (21%)[20] and C4 (25%)[9] silylation was measured by adding 1,1,2,2-tetrachloroethane (26.4 μL, 0.25 mmol) and comparing the corresponding signal in the ^{1}H NMR spectrum.

^{1}H NMR (300 MHz, $CDCl_3$):

· *for 2-(triethylsilyl)pyridine:* *δ* 8.72 – 8.66 (m, 1H), 7.50 – 7.43 (m, 1H), 7.40 – 7.35 (m, 1H), 7.11 – 7.04 (m, 1H), 0.91 – 0.84 (m, 9H), 0.78 – 0.69 (m, 6H).

· *for 3-(triethylsilyl)pyridine:* *δ* 8.60 – 8.57 (m, 1H), 8.52 – 8.47 (m, 1H), 7.70 – 7.63 (m, 1H), 7.19 – 7.13 (m, 1H), 0.91 – 0.84 (m, 9H), 0.78 – 0.69 (m, 6H).

· *for 4-(triethylsilyl)pyridine:* *δ* 8.49 – 8.44 (m, 2H), 7.30 – 7.25 (m, 2H), 0.91 – 0.84 (m, 9H), 0.78 – 0.69 (m, 6H).

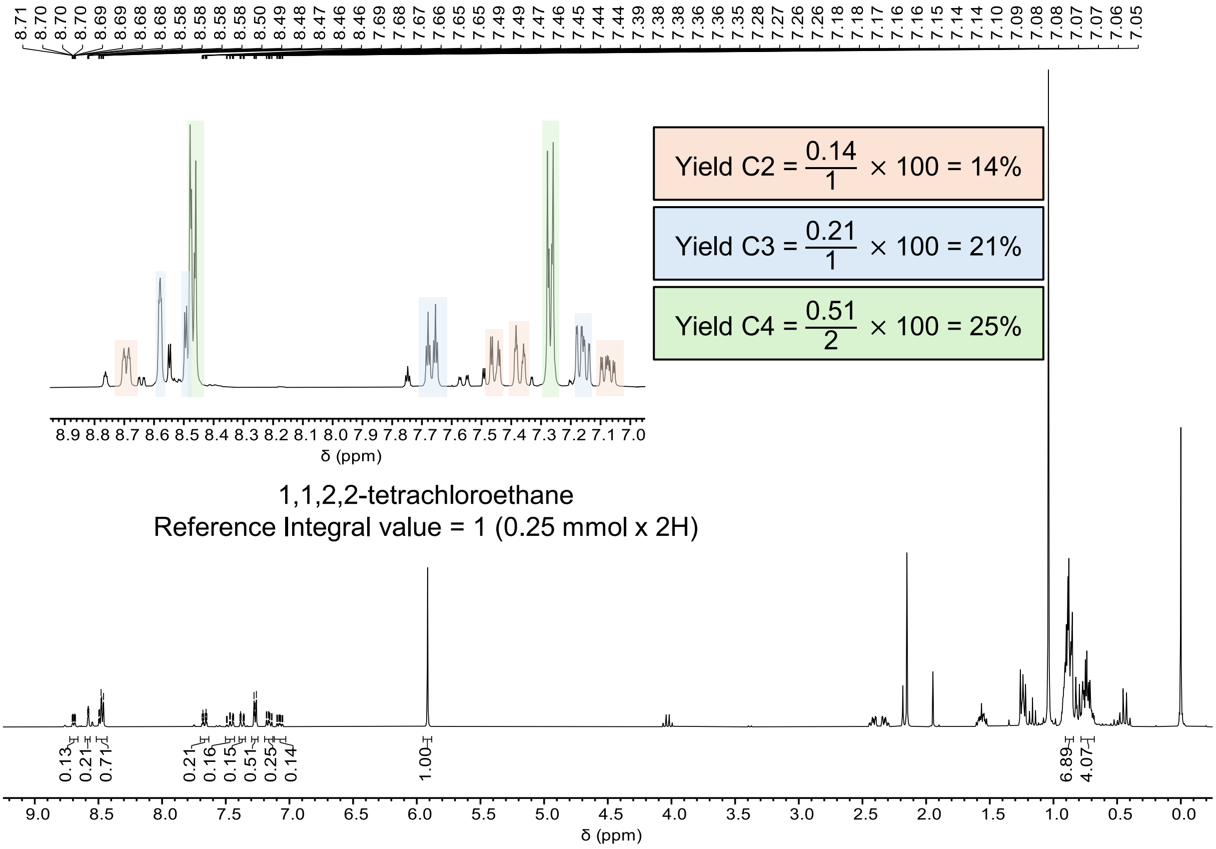

Figure S63. ^{1}H NMR of **3k** (mixture of 2, 3 and 4-(triethylsilyl)pyridine) in $CDCl_3$.

2-Methoxy-3-(triethylsilyl)pyridine (3l)

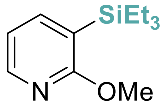

$C_{12}H_{21}NOSi$
Molecular Weight: 223.39

Prepared according to General Procedure A and purified by flash column chromatography in silica gel and hexane:EtOAc (97:3), compound **3l** was isolated as a colorless oil (76 mg, 68 % yield).

^{1}H NMR (300 MHz, $CDCl_3$): δ 8.15 (dd, J = 5.1, 2.1 Hz, 1H), 7.62 (dd, J = 7.0, 2.1 Hz, 1H), 8.15 (dd, J = 7.0, 5.1 Hz, 1H), 3.91 (s, 3H), 0.97 – 0.89 (m, 9H), 0.85 – 0.75 (m, 6H).

^{13}C{^{1}H} NMR (75 MHz, $CDCl_3$): δ 168.0, 147.7, 145.6, 119.0, 116.7, 53.1, 7.6, 3.1.

HRMS (ESI+) m/z: $[M+H]^+$ Calculated for $C_{12}H_{22}ONSi$ 224.1465. Found 224.1462.

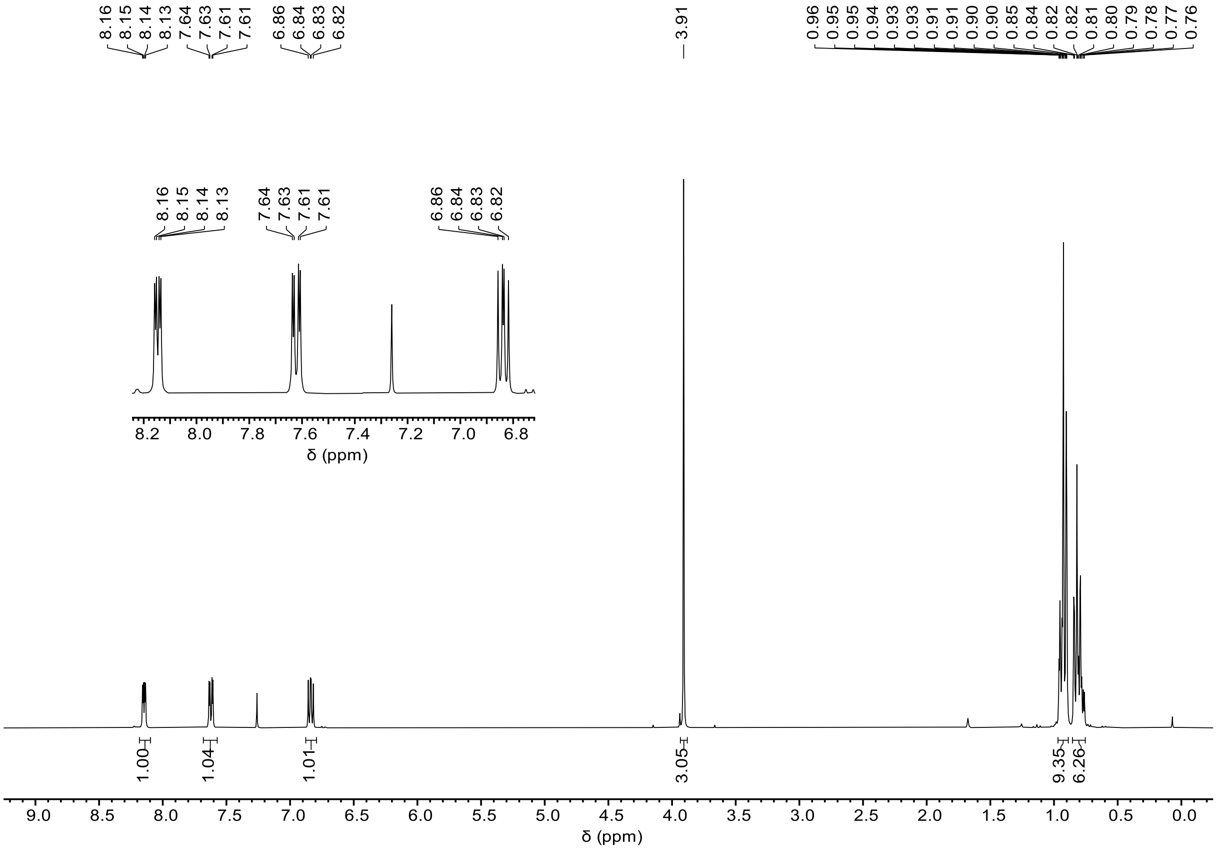

Figure S64. ^{1}H NMR of **3l** in $CDCl_3$.

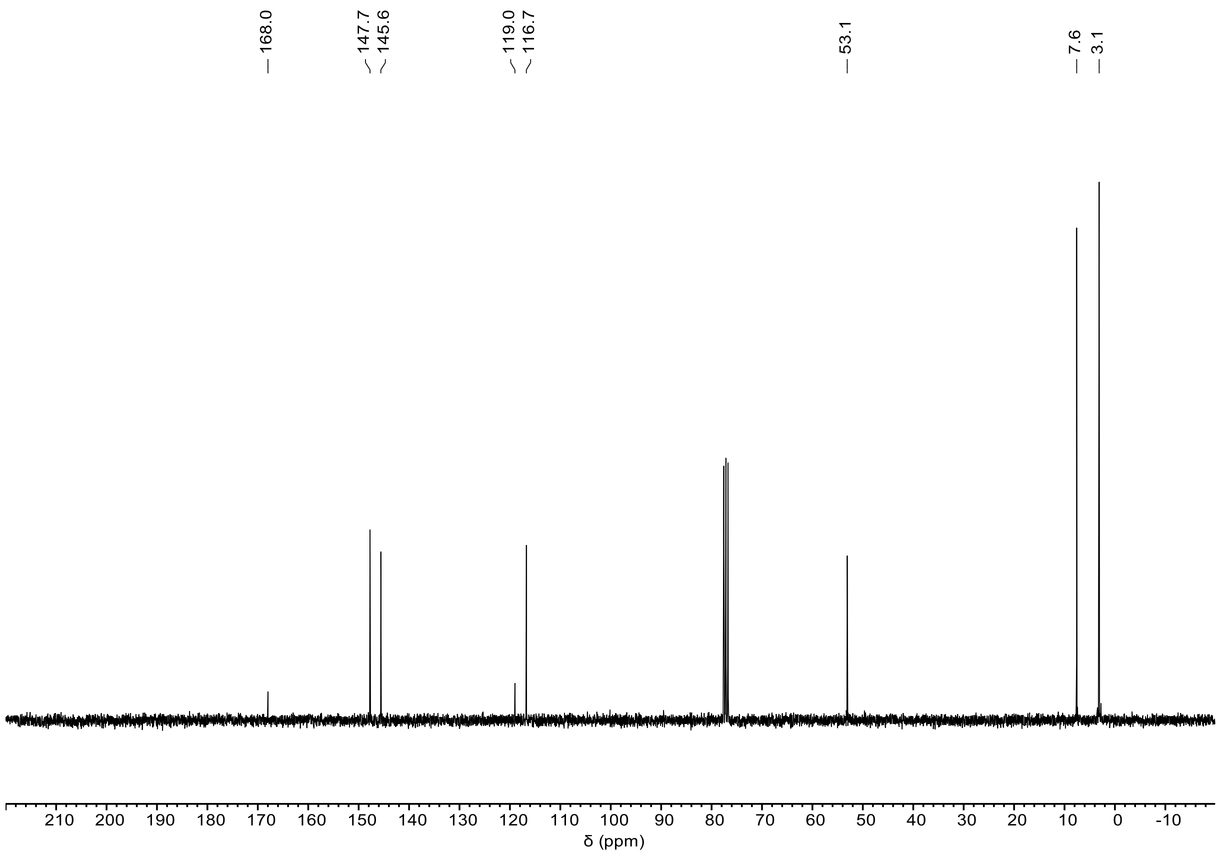

Figure S65. $^{13}C\{^{1}H\}$ NMR of **3l** in $CDCl_3$.

2,6-Di-*tert*-butyl-4-(triethylsilyl)pyridine (3m)

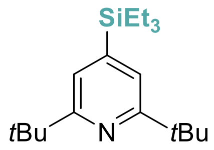

$C_{19}H_{35}NSi$
Molecular Weight: 305.58

Prepared according to General Procedure A and purified by flash column chromatography in silica gel and hexane, compound **3m** was isolated as a colorless oil (98 mg, 64 % yield).

^{1}H NMR (300 MHz, $CDCl_3$): δ 7.21 (s, 2H), 1.39 (s, 18H), 1.02 (t, J = 7.4 Hz, 9H), 0.83 (q, J = 7.4 Hz, 6H).

^{13}C{^{1}H} NMR (75 MHz, $CDCl_3$): δ 166.1, 146.7, 120.5, 37.7, 30.4, 7.6, 3.3.

HRMS (ESI+) m/z: $[M+H]^+$ Calculated for $C_{19}H_{36}NSi$ 306.2612. Found 306.2606.

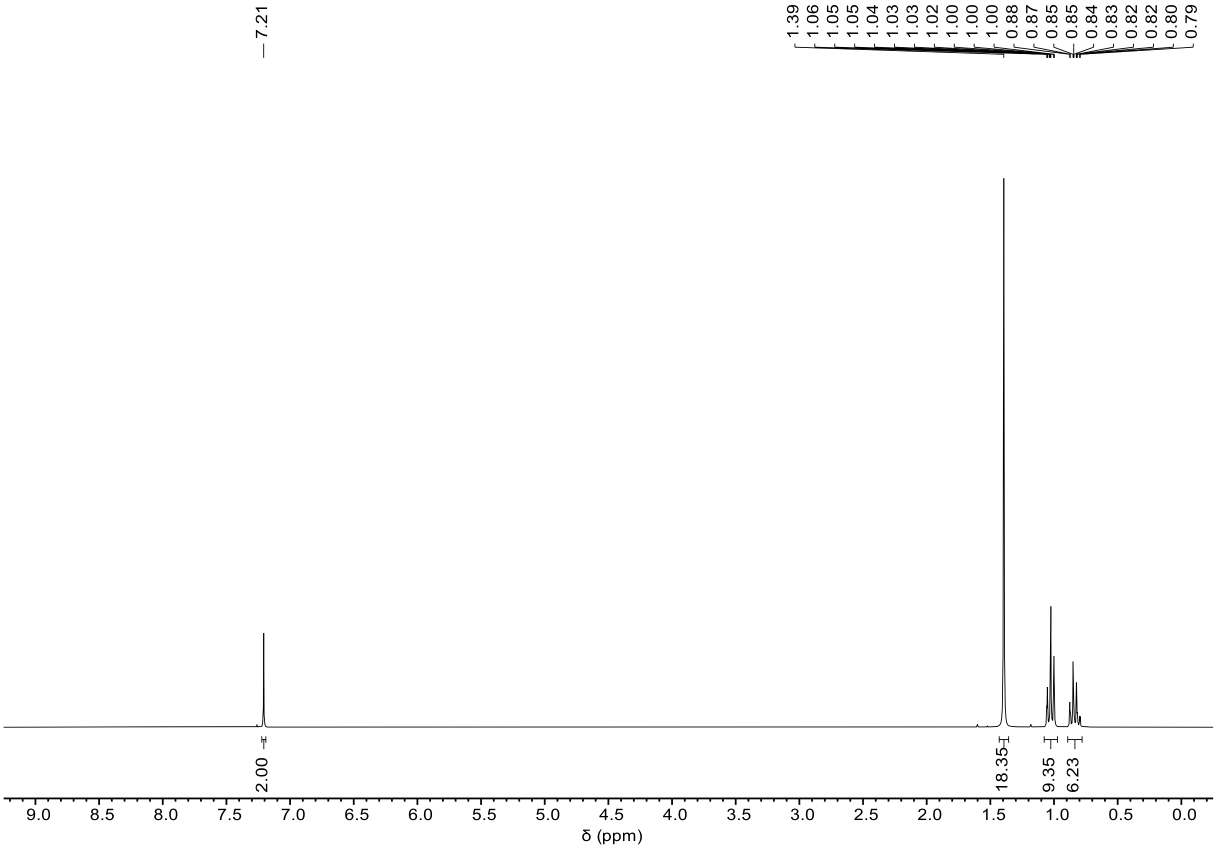

Figure S66. ^{1}H NMR of **3m** in $CDCl_3$.

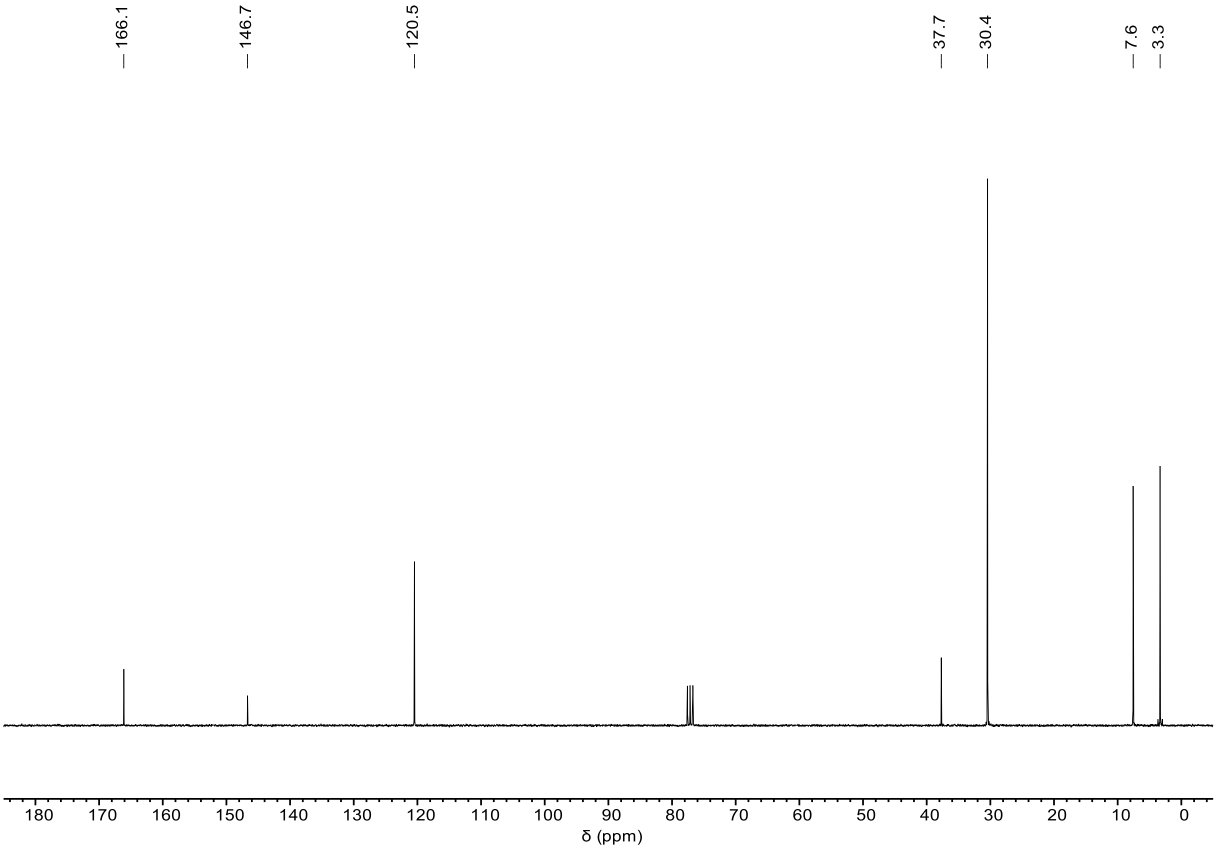

Figure S67. $^{13}C\{^{1}H\}$ NMR of **3m** in $CDCl_3$.

4-(Triethylsilyl)acridine (3n)

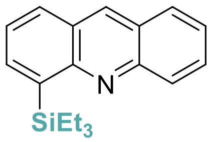

$C_{19}H_{23}NSi$
Molecular Weight: 293.48

Prepared according to General Procedure A, performing the addition of the reagents at -30 ºC keeping the mixture at that temperature for 6 h, and purified by flash column chromatography in silica gel and hexane, compound **3n** was isolated as a colorless oil (60 mg, 41 % yield). Spectroscopic data are in accordance with literature reports.[21]

^{1}H NMR (300 MHz, $CDCl_3$): *δ* 8.70 (s, 1H), 8.25 (dq, *J* = 8.8, 0.9 Hz, 1H), 8.02 – 7.96 (m, 2H), 7.94 (dd, *J* = 6.5, 1.5 Hz, 1H), 7.75 (ddd, *J* = 8.8, 6.6, 1.5 Hz, 1H), 7.56 – 7.47 (m, 2H), 1.22 – 1.12 (m, 6H), 1.07 – 0.99 (m, 9H).

^{13}C{^{1}H} NMR (75 MHz, $CDCl_3$): *δ* 153.4, 148.3, 139.8, 138.0, 135.7, 130.1, 129.5, 129.2, 128.1, 126.4, 126.2, 125.5, 125.5, 8.0, 4.4.

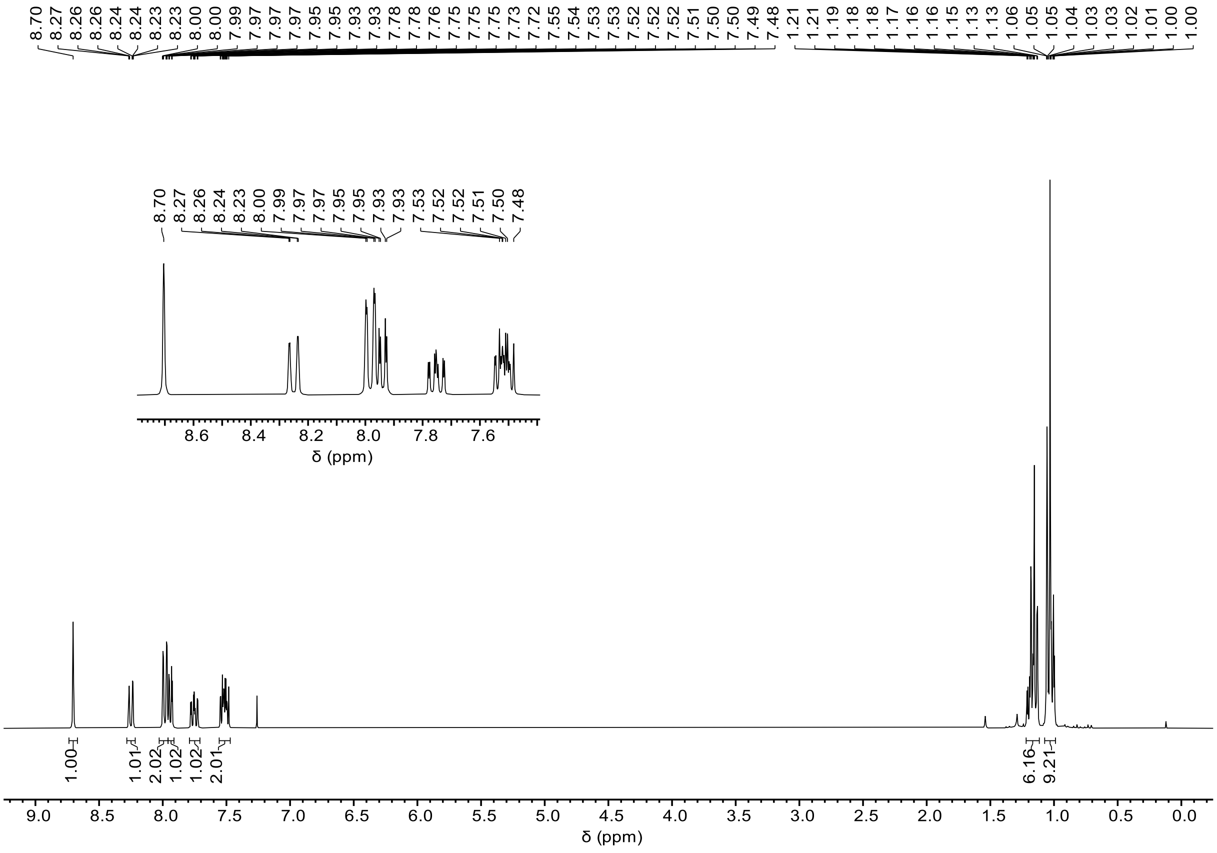

Figure S68. ^{1}H NMR of **3n** in $CDCl_3$.

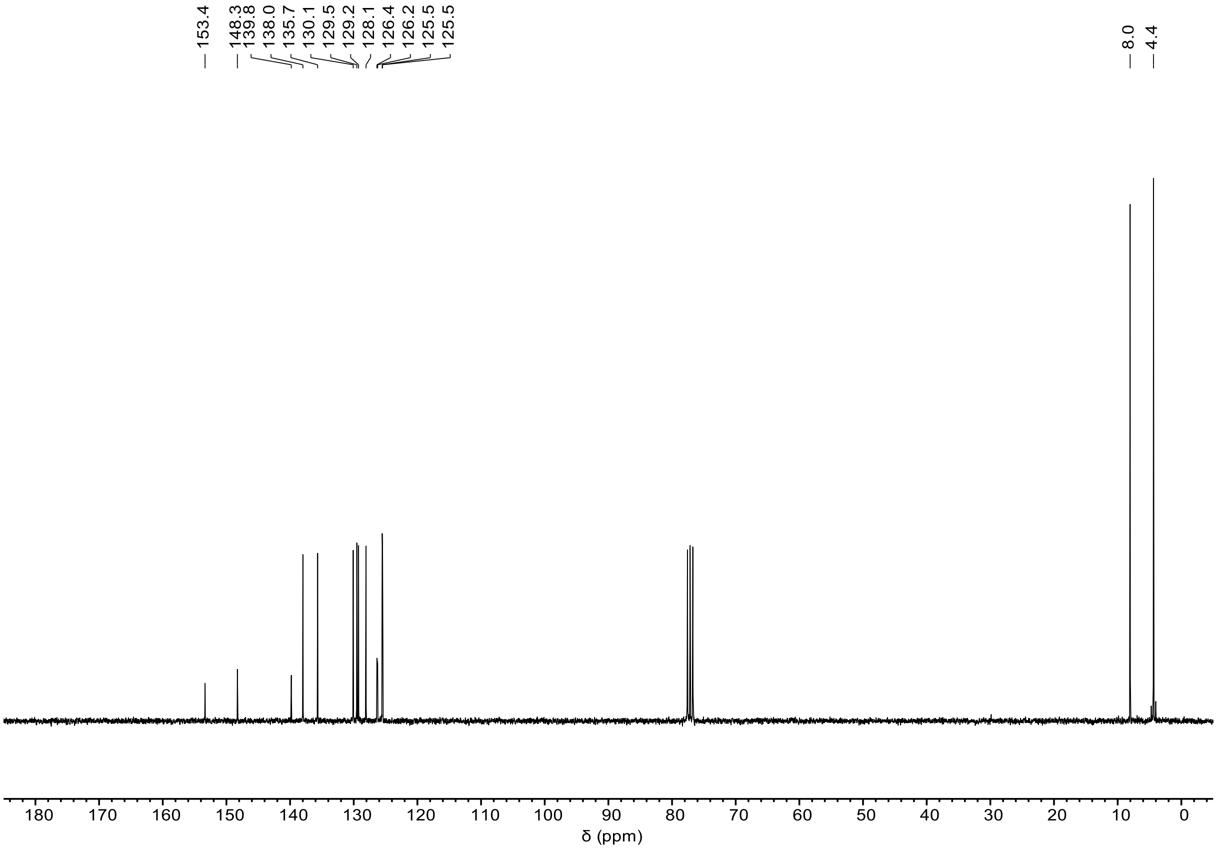

Figure S69. $^{13}C\{^{1}H\}$ NMR of **3n** in $CDCl_3$.

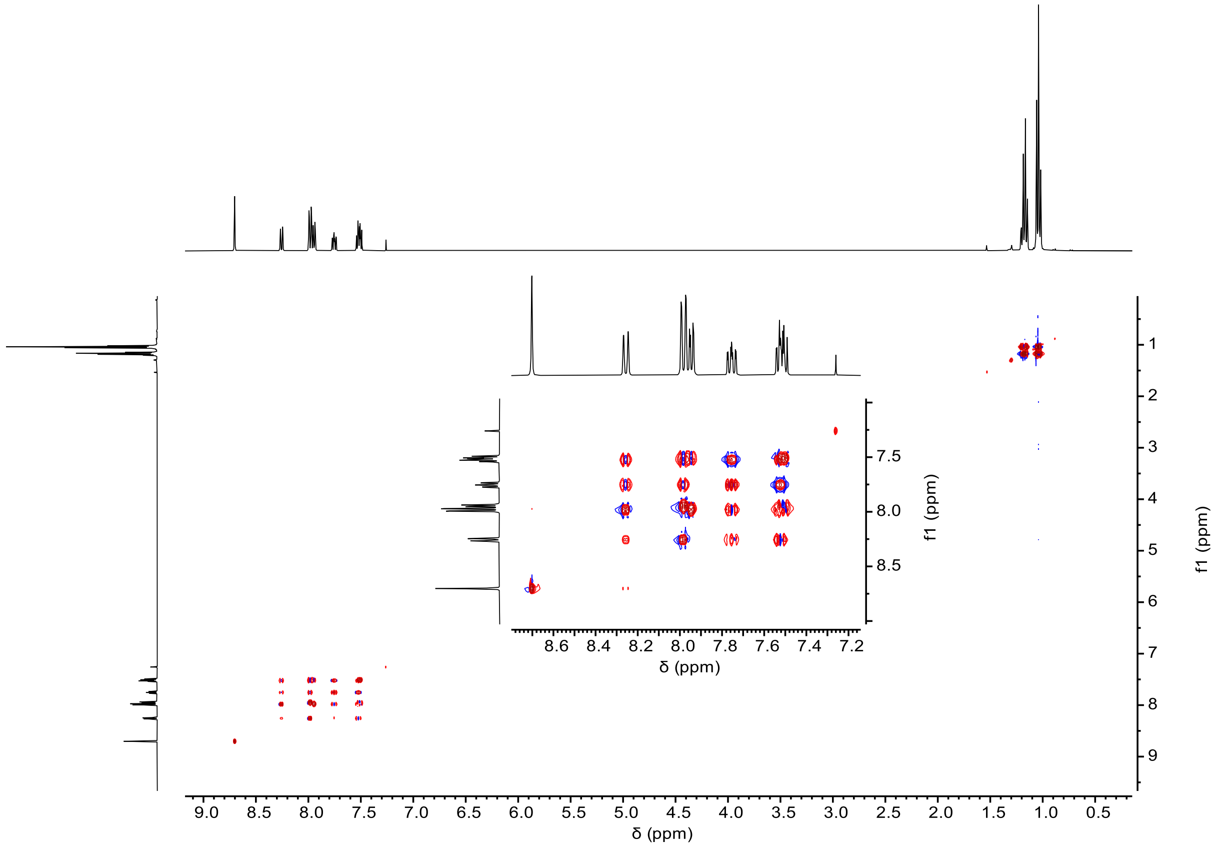

Figure S69b. ^{1}H–^{1}H TOCSY NMR of **3n** in $CDCl_3$.

3-(1-Methylpyrrolidin-2-yl)-5-(triethylsilyl)pyridine (3o)

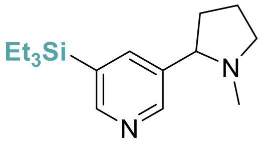

$C_{16}H_{28}N_2Si$
Molecular Weight: 276.50

Prepared according to General Procedure A, performing the addition of the reagents at -30 ºC and keeping the mixture at that temperature for 6 h. NMR yield for **3o** (31%) was measured by adding 1,1,2,2-tetrachloroethane (26.4 µL, 0.25 mmol) and comparing the corresponding signal in the ^{1}H NMR spectrum.

^{1}H NMR (300 MHz, $CDCl_3$): δ 8.47 – 8.44 (m, 1H), 8.43 – 8.41 (m, 1H), 7.68 – 7.65 (m, 1H), 3.20 – 3.11 (m, 1H), 3.04 – 2.92 (m, 1H), 2.36 – 2.25 (m, 1H), 2.25 – 2.14 (m, 1H), 2.16 (s, 3H), 1.95 – 1.81 (m, 1H), 1.80 – 1.53 (m, 2H), 0.94 – 0.84 (m, 9H), 0.81 – 0.70 (m, 6H).

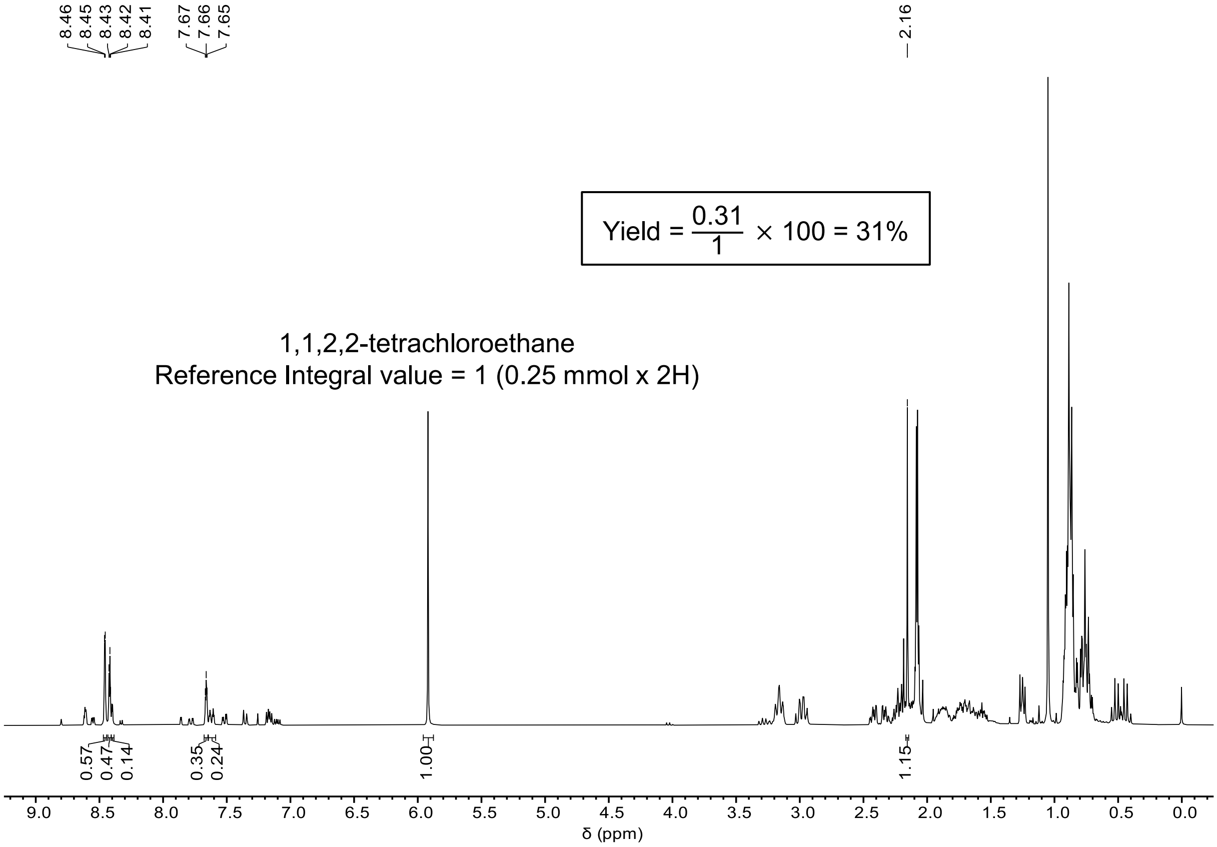

Figure S70. ^{1}H NMR of **3o** (not isolated) in $CDCl_3$.

Synthesis of *gem*-disilane

(Phenylmethylene)bis(triethylsilane) (4a)

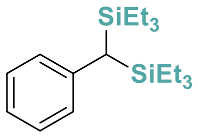

$C_{19}H_{36}Si_2$
Molecular Weight: 320.67

In an oven-dried and argon-flushed Schlenk flask containing a Teflon-coated magnetic stirrer, NaTMP (81.6 mg, 0.5 mmol, 1 eq.) was suspended in 3 mL of dry hexane. The resulting white suspension was stirred and Et_3SiCl (83.9 µL, 1.0 mmol, 1 eq.) was added, followed by toluene (53.4 µL, 0.5 mmol, 1 eq.) and PMDETA (20.8 µL, 0.1 mmol, 20 mol%). The mixture was stirred at room temperature for 4 h, and then a second equivalent of NaTMP and Et_3SiCl were added. The mixture was then stirred at room temperature for 16 h. Afterwards, the reaction was quenched with a saturated solution of NH_4Cl (10 mL) and extracted with Et_2O (3 x 10 mL). The organic phase was dried over Na_2SO_4, filtered and dried under reduced pressure. NMR yield was measured by adding 1,1,2,2-tetrachloroethane (26.4 µL, 0.25 mmol) and comparing the corresponding signal in the ^{1}H NMR spectrum. Purification was performed by flash column chromatography in silica gel and hexane. Compound **4a** was isolated as a colorless oil (120 mg, 75 % yield).

Alternatively, compound **4a** can be prepared from benzyltriethylsilane (**1a**), NaTMP, Et_3SiCl and PMDETA (see NMR monitoring experiments).

^{1}H NMR (300 MHz, $CDCl_3$): δ 7.19 – 7.12 (m, 2H), 7.02 (tt, *J* = 7.3, 1.3 Hz, 1H), 7.00 – 6.94 (m, 2H), 1.78 (s, 1H), 0.90 (t, *J* = 8.1 Hz, 18H), 0.58 (q, *J* = 8.1 Hz, 12H).

^{13}C{^{1}H} NMR (75 MHz, $CDCl_3$): δ 142.9, 129.5, 128.0, 123.3, 22.1, 7.9, 4.9.

HRMS (EI) m/z: $[M]^+$ Calculated for $C_{19}H_{36}Si_2$ 320.2350. Found 320.2356.

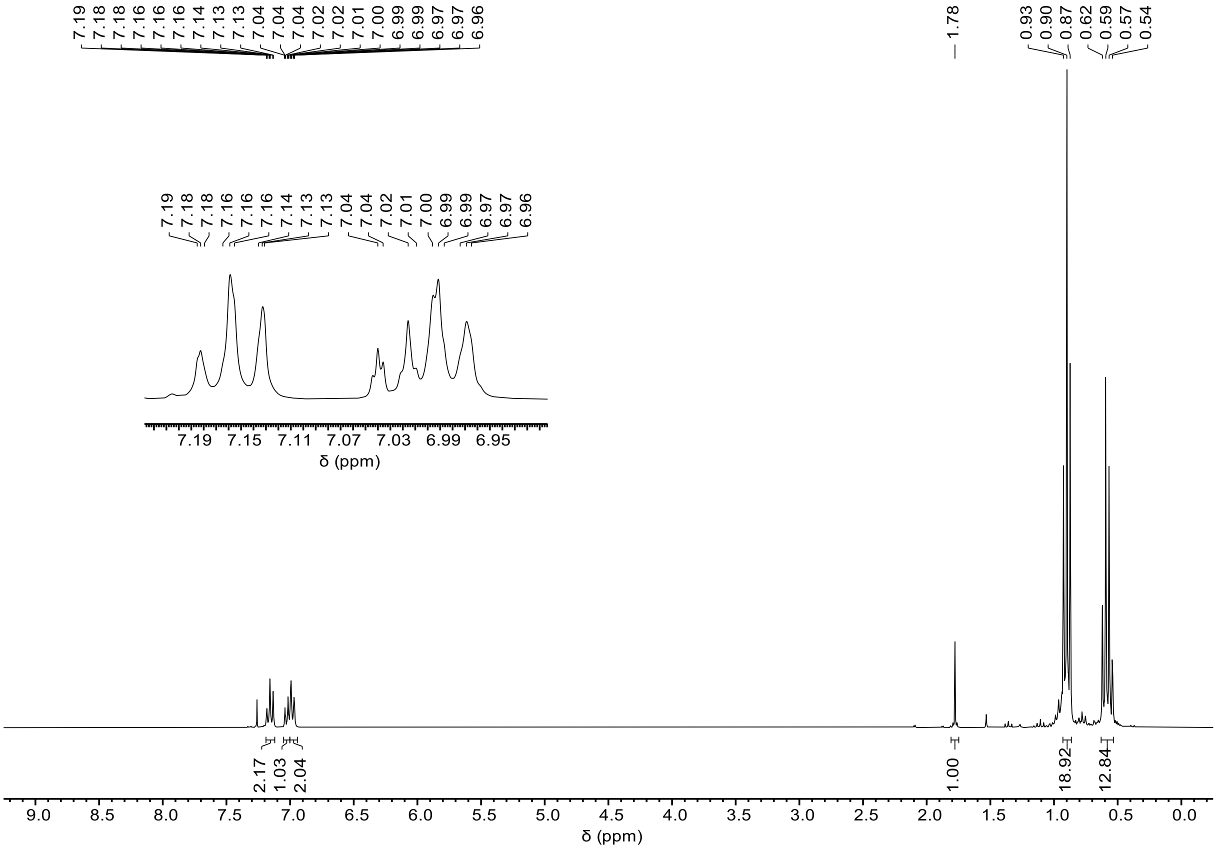

Figure S71. ^{1}H NMR of **4a** in $CDCl_3$.

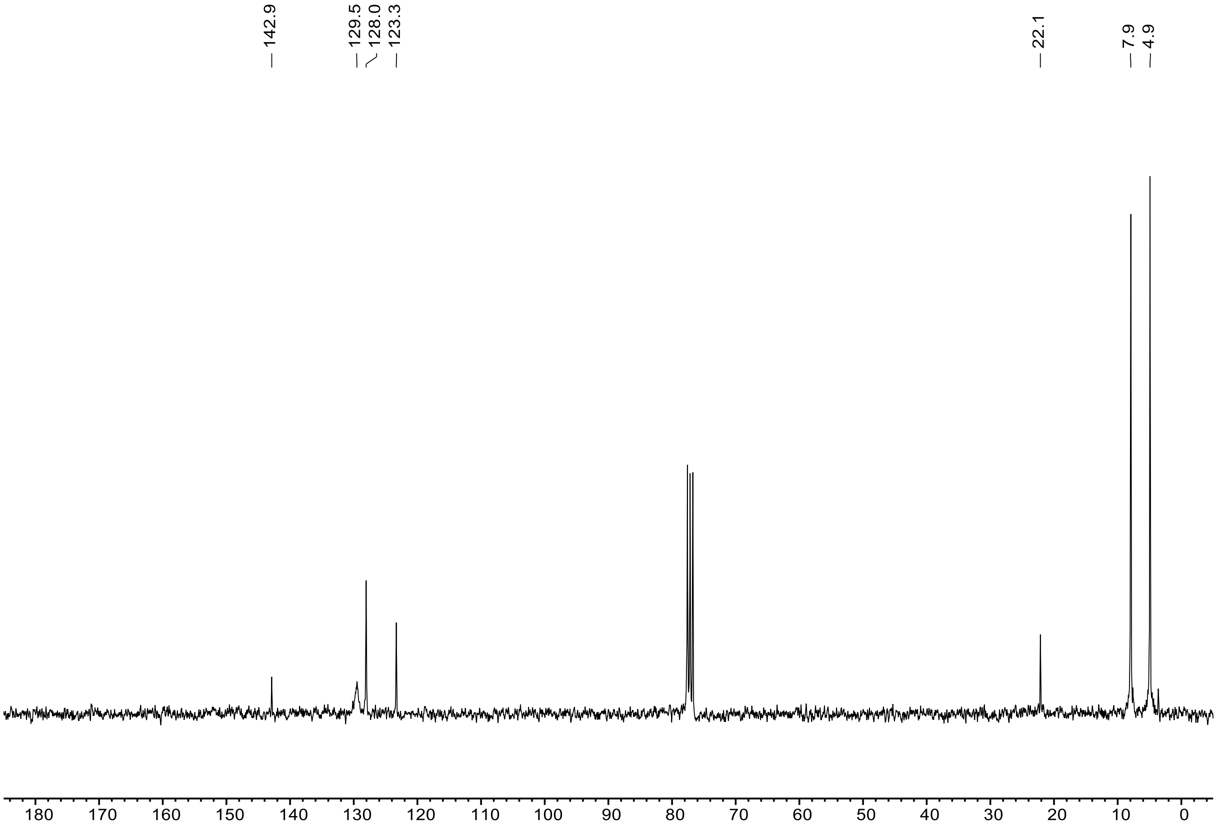

Figure S72. $^{13}C\{^{1}H\}$ NMR of **4a** in $CDCl_3$.

General Procedure B: Multisilylation

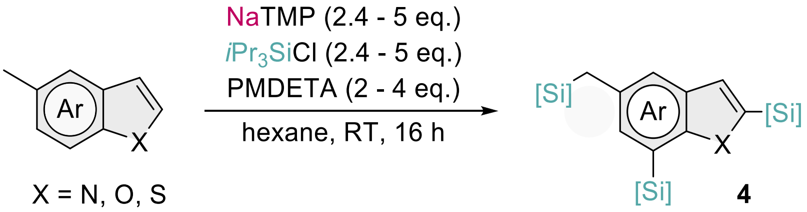

In an oven-dried and argon-flushed Schlenk flask containing a Teflon-coated magnetic stirrer, NaTMP was suspended in 3 mL of dry hexane. The resulting white suspension was stirred and iPr$_3$SiCl was added, followed by the desired substrate (0.5 mmol, 1 eq.) and PMDETA. The mixture was then stirred at room temperature for 16 h. Afterwards, the reaction was quenched with a saturated solution of NH_4Cl (10 mL) and extracted with Et_2O (3 x 10 mL). The organic phase was dried over Na_2SO_4, filtered and dried under reduced pressure. NMR yield was measured by adding 1,1,2,2-tetrachloroethane (26.4 µL, 0.25 mmol) and comparing the corresponding signal in the ^{1}H NMR spectrum. Purification of the silylated compounds was performed by flash column chromatography. The number of equivalents of each reagent, the details of the purification process and other possible deviations from the General Method are specified with each substrate.

Spectroscopic Data for Compounds 4b-q

1,4-Bis((triisopropylsilyl)methyl)benzene (4b)

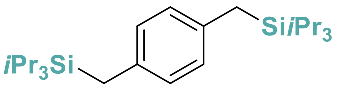

$C_{26}H_{50}Si_2$
Molecular Weight: 418.86

Prepared according to General Procedure B (2.4 eq. of NaTMP, 2.4 eq. of *i*Pr_3SiCl and 2 eq. of PMDETA) and purified by flash column chromatography in silica gel and hexane, compound **4b** was isolated as a white solid (203 mg, 97 % yield).

^{1}H NMR (300 MHz, $CDCl_3$): δ 6.99 (s, 4H), 2.18 (s, 4H), 1.19 – 1.10 (m, 6H), 1.10 – 1.03 (m, 36H).

^{13}C{^{1}H} NMR (75 MHz, $CDCl_3$): δ 136.2, 128.6, 18.8, 18.4, 11.2.

HRMS (ESI+) m/z: $[M+H]^+$ Calculated for $C_{26}H_{51}Si_2$ 419.3524. Found 419.3526.

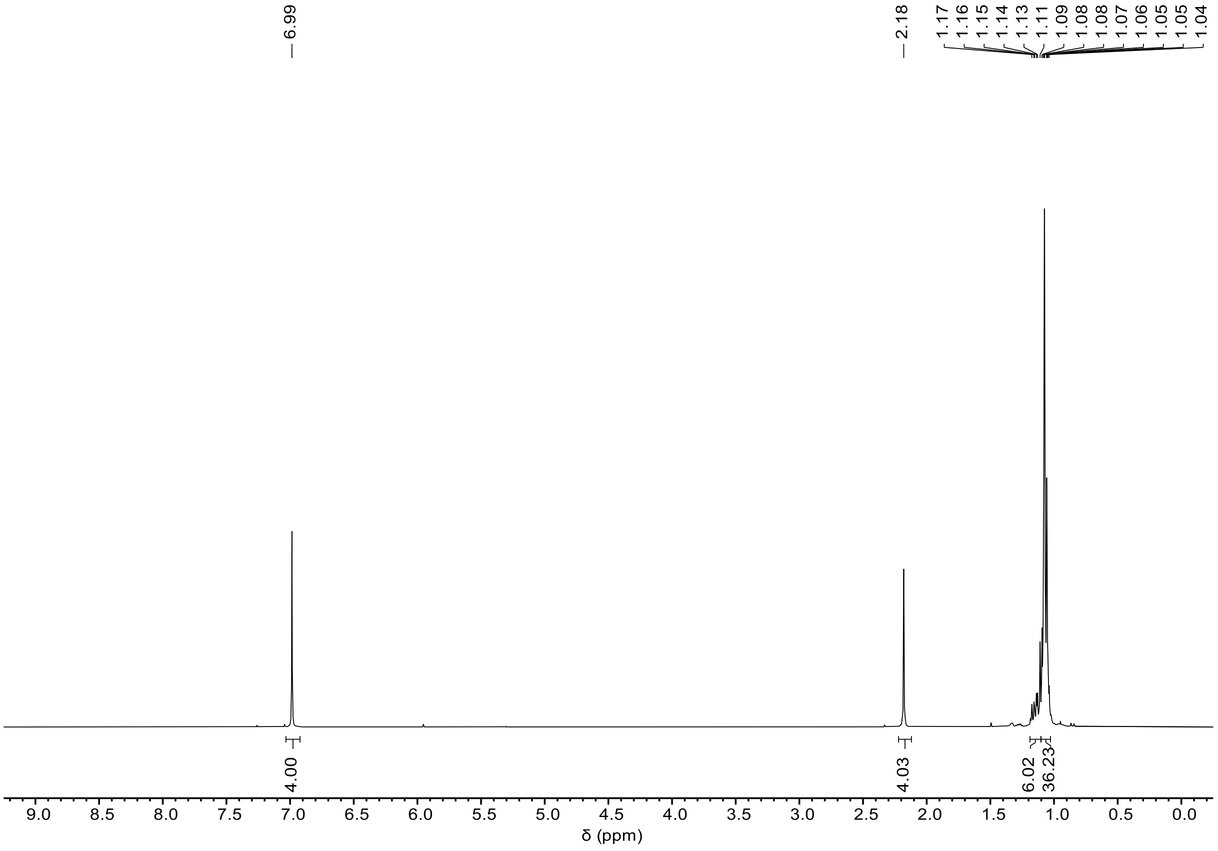

Figure S73. ^{1}H NMR of **4b** in $CDCl_3$.

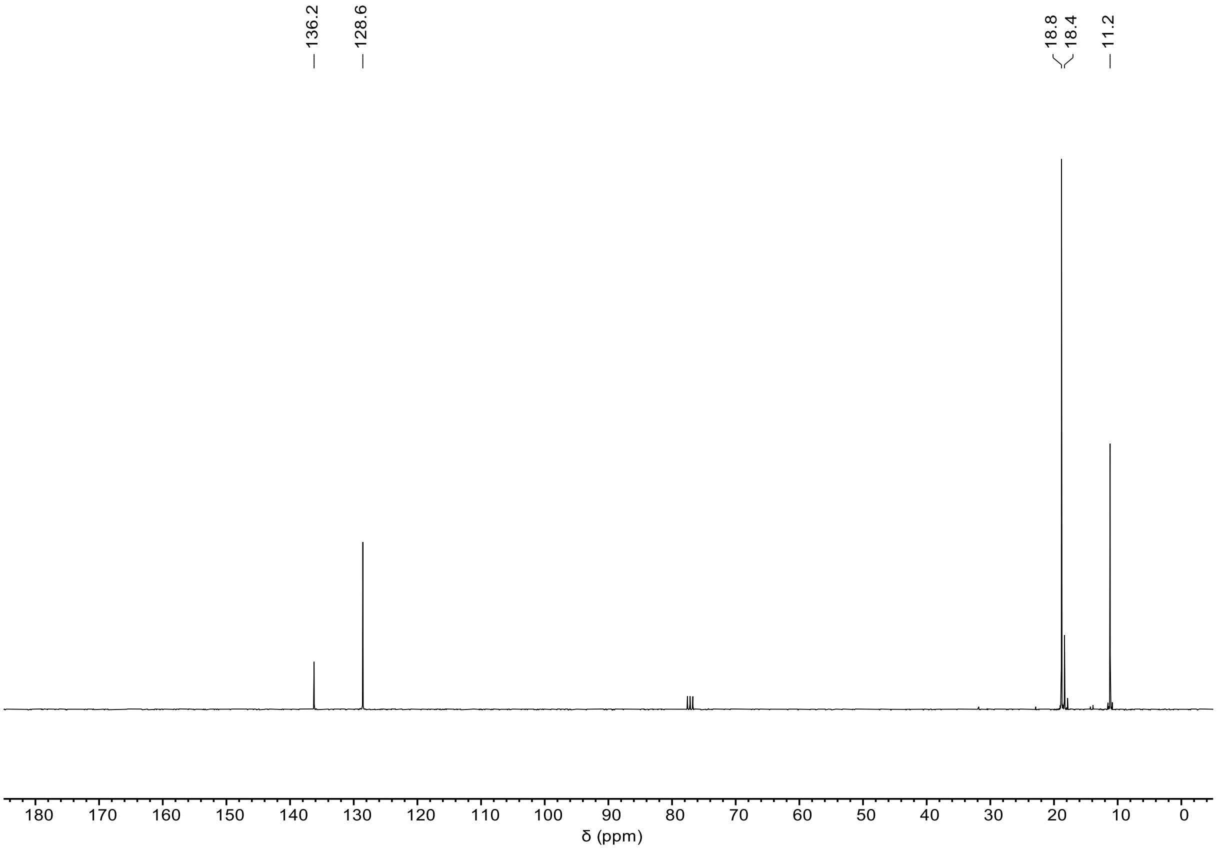

Figure S74. $^{13}C\{^{1}H\}$ NMR of **4b** in $CDCl_3$.

2,6-Bis((triisopropylsilyl)methyl)pyridine (4c)

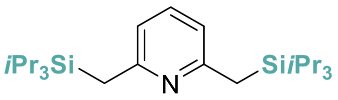

$C_{25}H_{49}NSi_2$
Molecular Weight: 419.84

Prepared according to General Procedure B (3 eq. of NaTMP, 3 eq. of *i*Pr_3SiCl and 3 eq. of PMDETA) and purified by flash column chromatography in silica gel and hexane/EtOAc (97:3), compound **4c** was isolated as a colorless oil (191 mg, 81 % yield).

^{1}H NMR (300 MHz, $CDCl_3$): δ 7.25 (t, J = 7.7 Hz, 1H), 6.73 (d, J = 7.7 Hz, 2H), 2.34 (s, 4H), 1.20 – 1.09 (m, 6H), 1.08 – 1.00 (m, 36H).

^{13}C{^{1}H} NMR (75 MHz, $CDCl_3$): δ 161.0, 135.4, 118.2, 22.6, 18.8, 11.4.

HRMS (ESI+) m/z: $[M+H]^+$ Calculated for $C_{25}H_{50}NSi_2$ 420.3476. Found 420.3471.

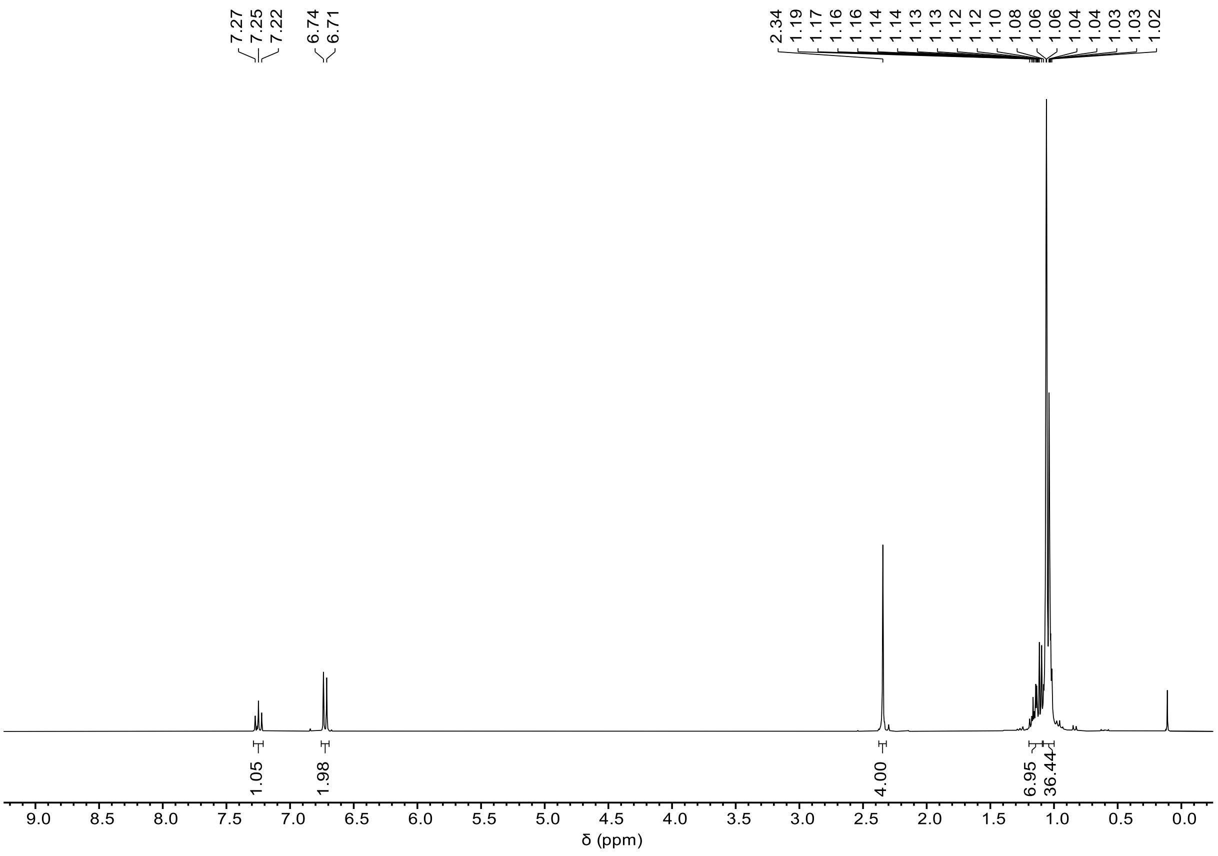

Figure S75. ^{1}H NMR of **4c** in $CDCl_3$.

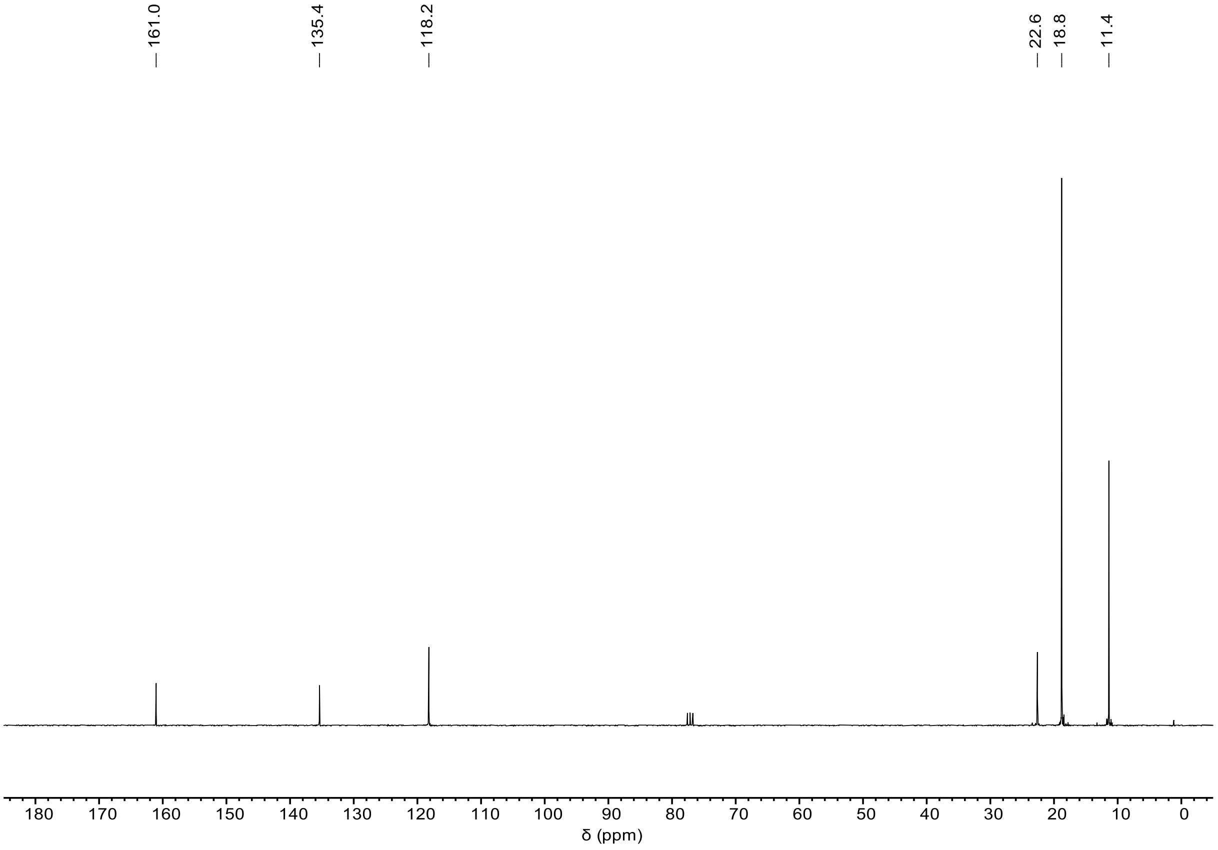

Figure S76. $^{13}\{^{1}H\}$ NMR of **4c** in $CDCl_3$.

1,3,5-Tris((triisopropylsilyl)methyl)benzene (4d)

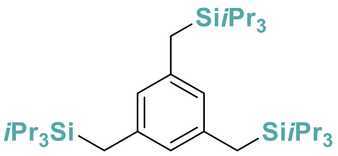

$C_{36}H_{72}Si_3$
Molecular Weight: 589.23

Prepared according to General Procedure B (4 eq. of NaTMP, 4 eq. of *i*Pr_3SiCl and 3 eq. of PMDETA) and purified by flash column chromatography in silica gel and hexane, compound **4d** was isolated as a colorless oil (282 mg, 96 % yield).

^{1}H NMR (300 MHz, $CDCl_3$): *δ* 6.55 (s, 3H), 2.05 (s, 6H), 1.12 – 1.06 (m, 9H), 1.05 – 1.00 (m, 54H).

^{13}C{^{1}H} NMR (75 MHz, $CDCl_3$): *δ* 140.6, 125.2, 18.9, 17.9, 11.3.

HRMS (EI) m/z: $[M]^+$ Calculated for $C_{36}H_{72}Si_3$ 588.4936. Found 588.4961.

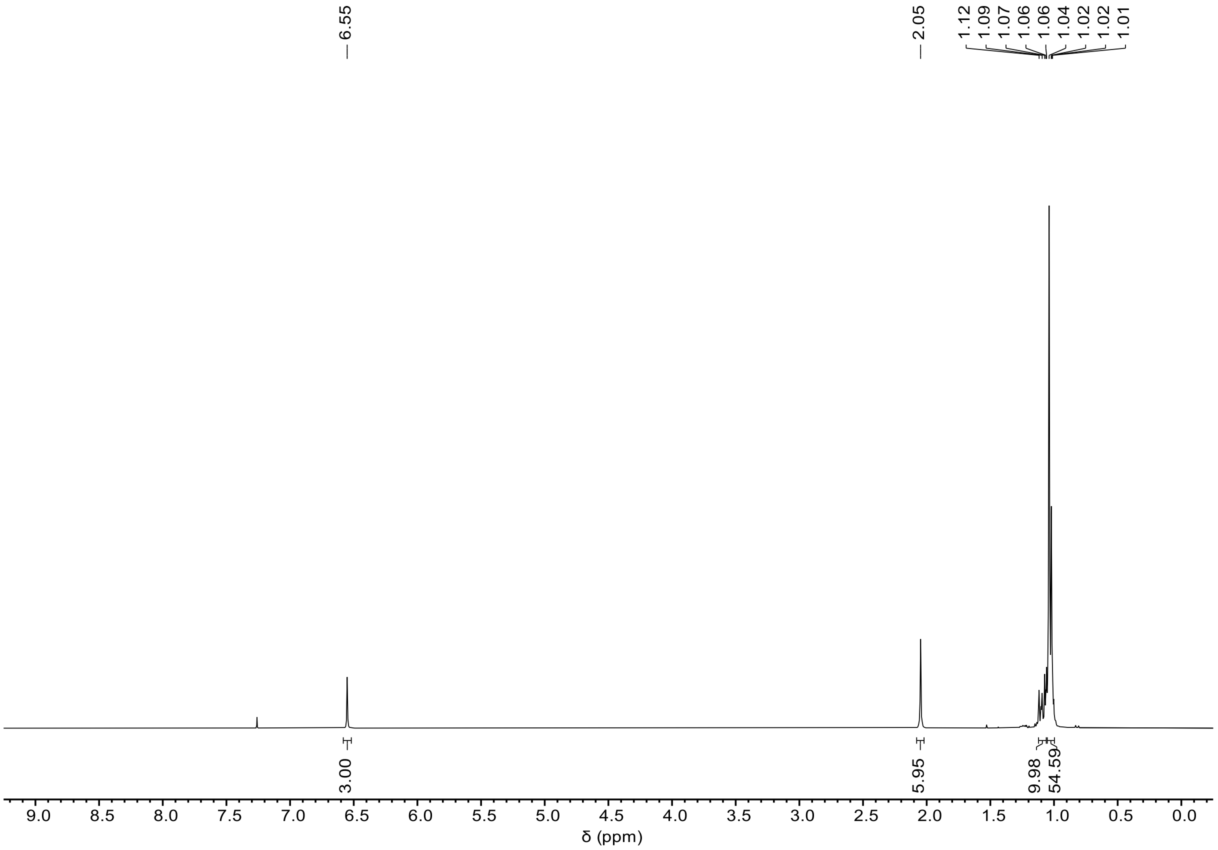

Figure S77. ^{1}H NMR of **4d** in $CDCl_3$.

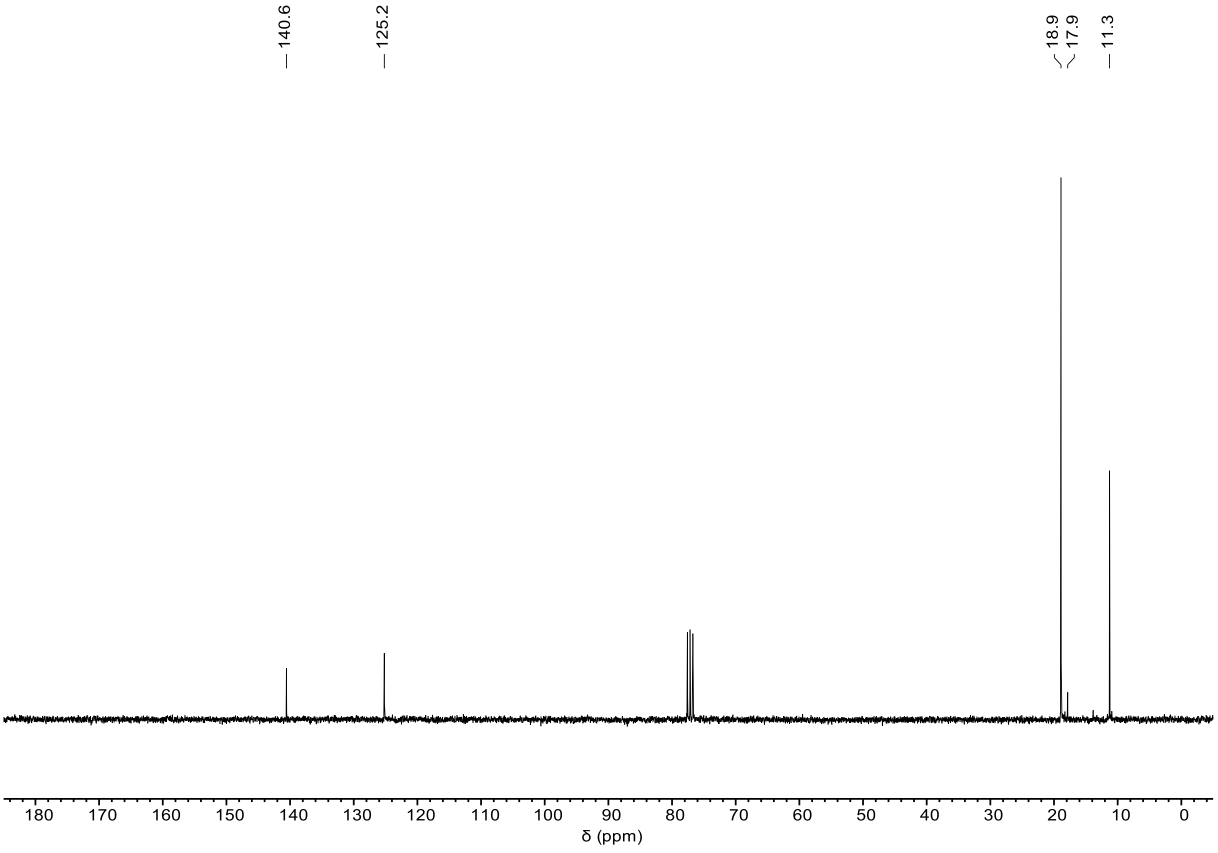

Figure S78. $^{13}C\{^{1}H\}$ NMR of **4d** in $CDCl_3$.

Tris(4-((triisopropylsilyl)methyl)phenyl)phosphane (4e)

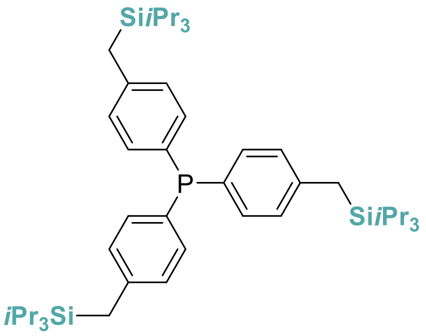

$C_{48}H_{81}PSi_3$
Molecular Weight: 773.40

Prepared according to General Procedure B (4 eq. of NaTMP, 4 eq. of iPr$_3$SiCl and 3 eq. of PMDETA) and purified by flash column chromatography in silica gel and hexane/EtOAc (98:2), compound **4e** was isolated as a white solid (302 mg, 81 % yield).

^{1}H NMR (300 MHz, $CDCl_3$): δ 7.12 – 7.00 (m, 12H), 2.19 (s, 6H), 1.13 – 1.04 (m, 9H), 1.13 – 0.96 (m, 54H).

^{13}C{^{1}H} NMR (75 MHz, $CDCl_3$): δ 141.9, 133.7 (d, J = 19.3 Hz), 132.7 (d, J = 8.6 Hz), 128.7 (d, J = 7.2 Hz), 19.1, 18.7, 11.1.

^{31}P{^{1}H} NMR (121 MHz, $CDCl_3$): δ -9.4.

HRMS (ESI+) m/z: $[M+H]^+$ Calculated for $C_{48}H_{82}PSi_3$ 773.5457. Found 773.5452.

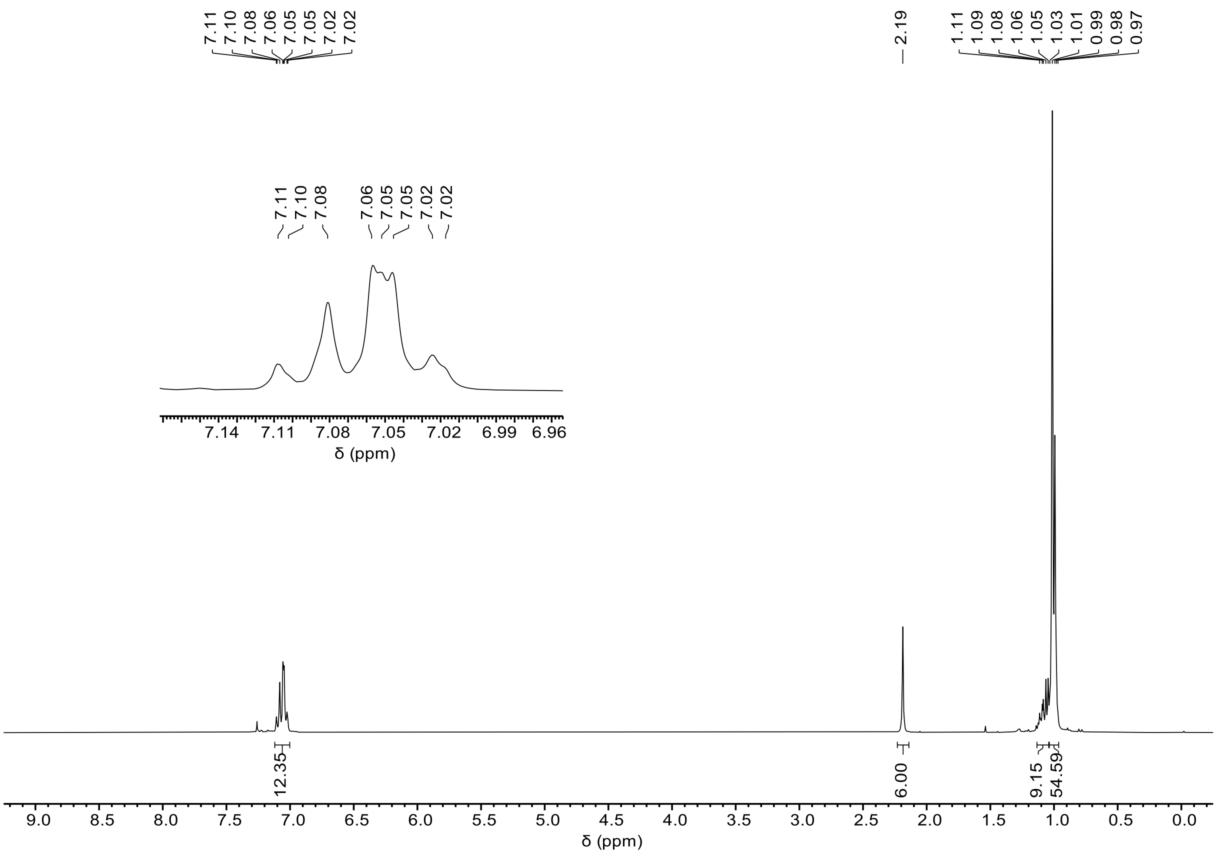

Figure S79. ^{1}H NMR of **4e** in $CDCl_3$.

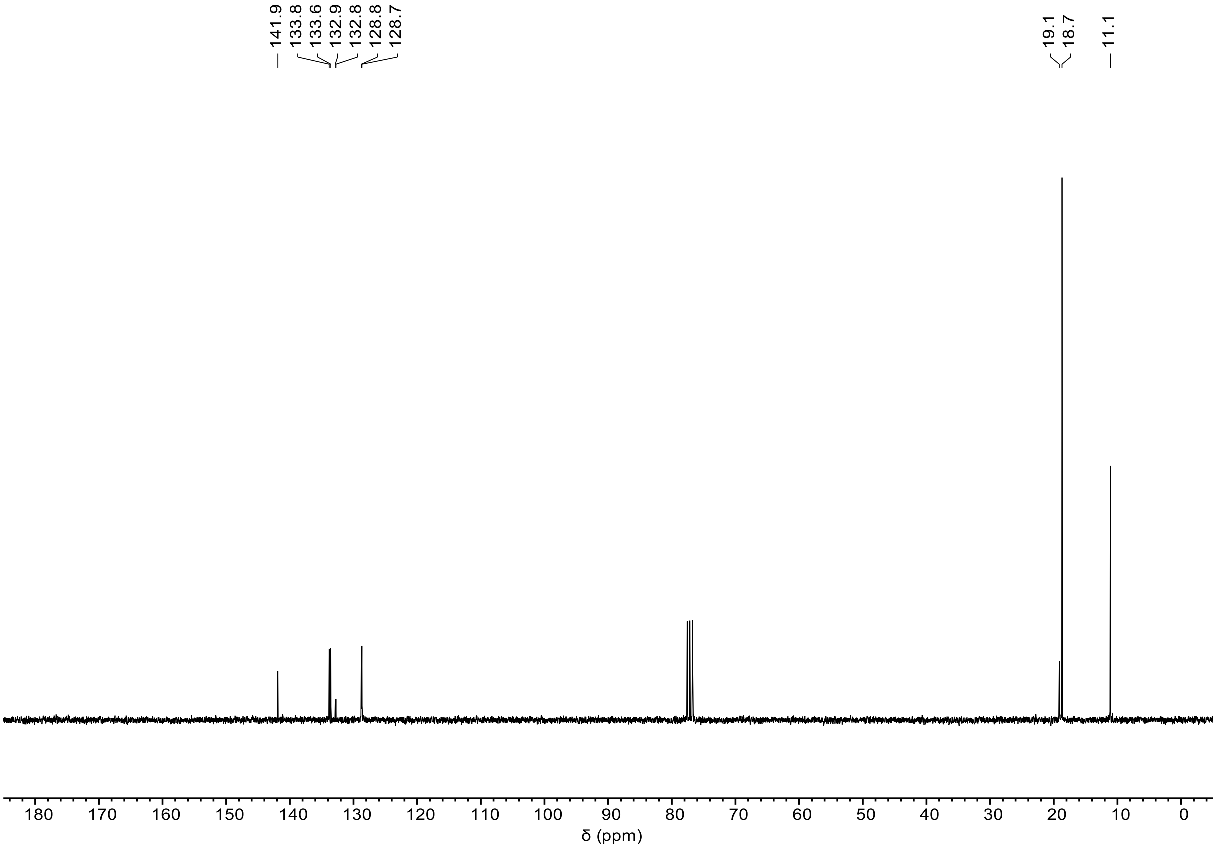

Figure S80. $^{13}C\{^{1}H\}$ NMR of **4e** in $CDCl_3$.

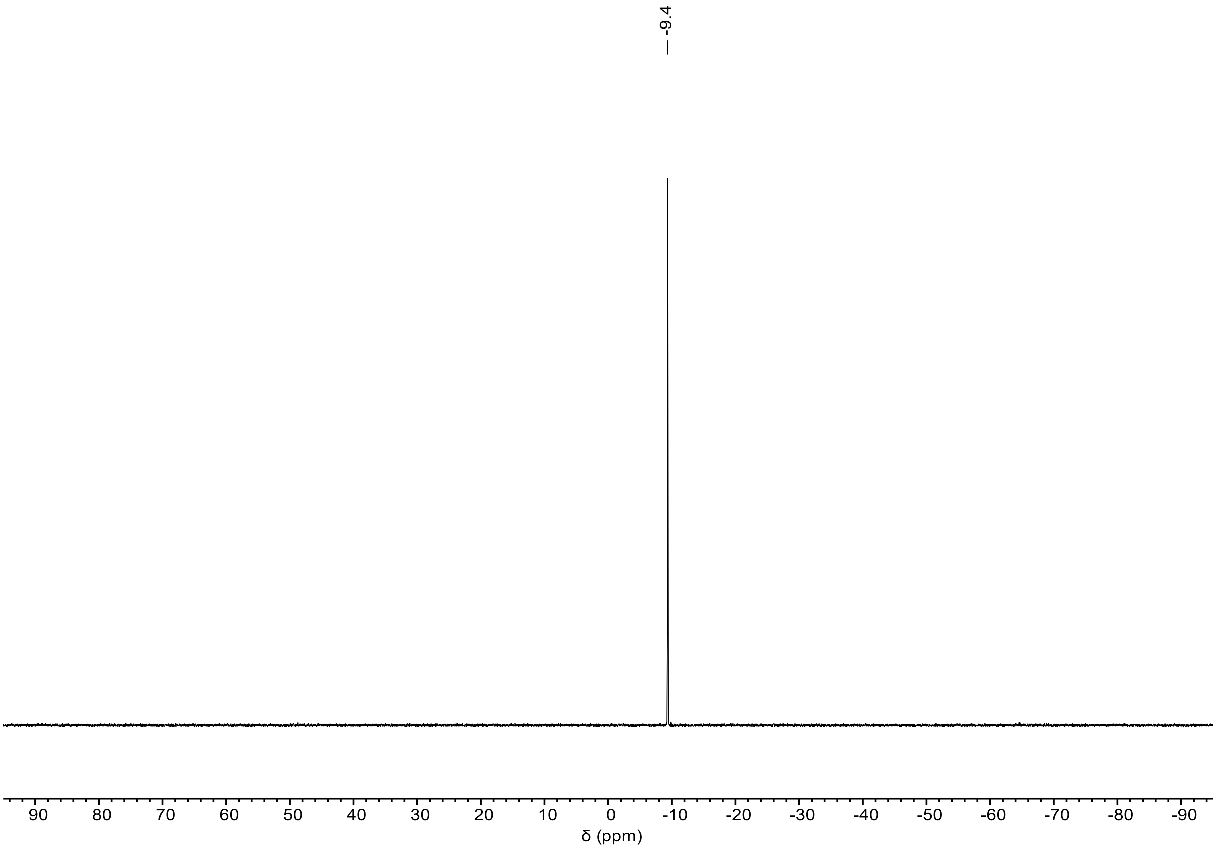

Figure S81. $^{31}P\{^{1}H\}$ NMR of **4e** in $CDCl_3$.

3,3',5,5'-Tetrakis((triisopropylsilyl)methyl)-1,1'-biphenyl (4f)

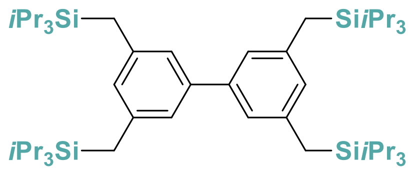

$C_{52}H_{98}Si_4$
Molecular Weight: 835.70

Prepared according to General Procedure B (5 eq. of NaTMP, 5 eq. of *i*Pr_3SiCl and 4 eq. of PMDETA) and purified by flash column chromatography in silica gel and hexane, compound **4f** was isolated as a white solid (386 mg, 93 % yield).

^{1}H NMR (300 MHz, $CDCl_3$): *δ* 7.03 (d, *J* = 1.3 Hz, 4H), 6.84 (t, *J* = 1.3 Hz, 2H), 2.24 (s, 8H), 1.23 – 1.13 (m, 12H), 1.13 – 1.04 (m, 72H).

^{13}C{^{1}H} NMR (75 MHz, $CDCl_3$): *δ* 141.7, 141.0, 127.4, 123.8, 19.2, 18.8, 11.3.

HRMS (ESI+) m/z: $[M+H]^+$ Calculated for $C_{52}H_{99}Si_4$ 835.6818. Found 835.6841.

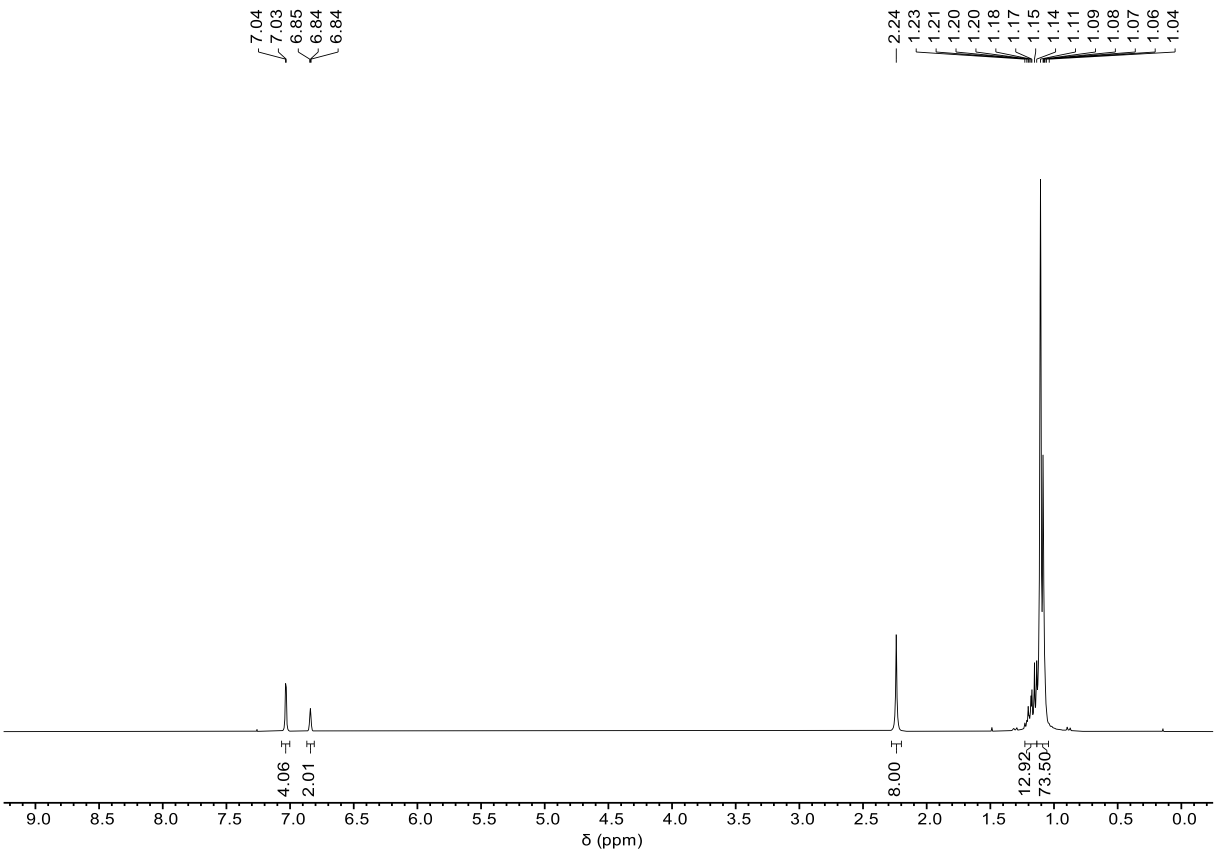

Figure S82. ^{1}H NMR of **4f** in $CDCl_3$.

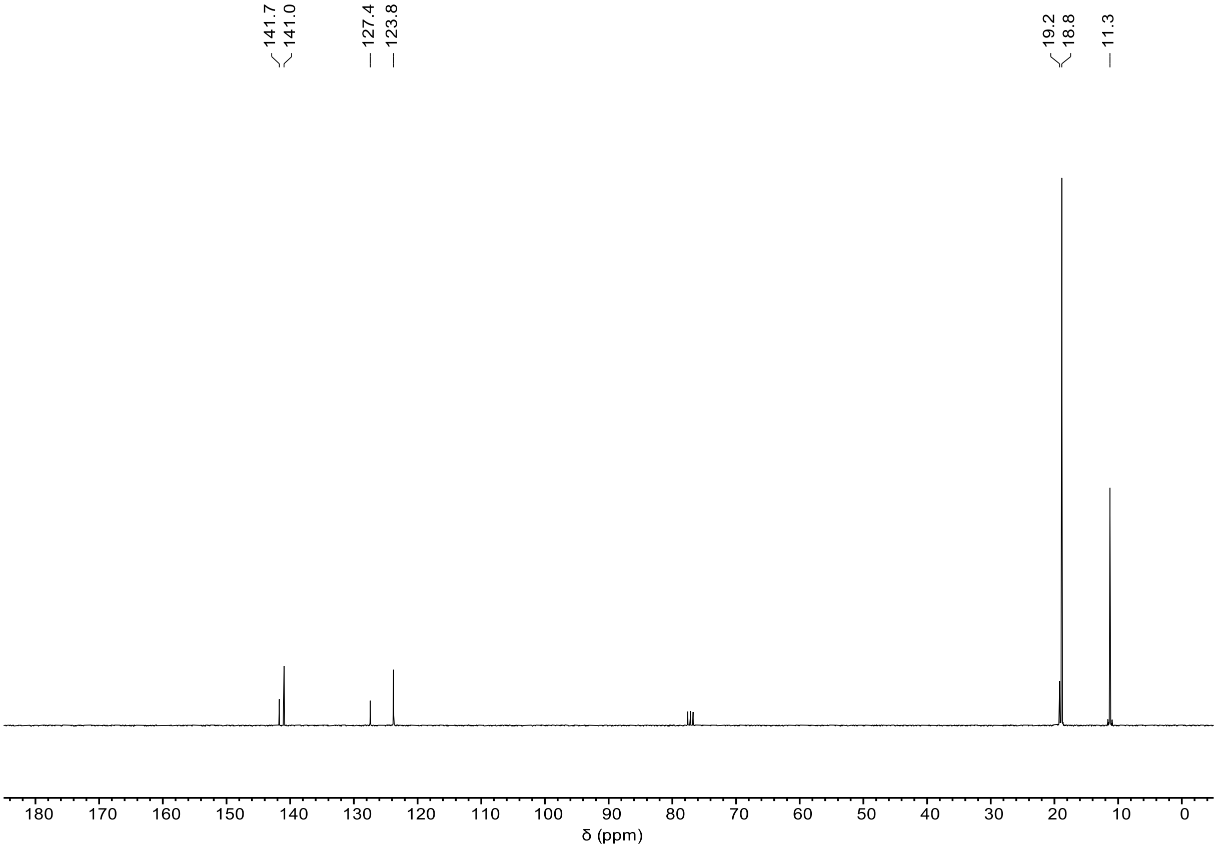

Figure S83. $^{13}C\{^{1}H\}$ NMR of **4f** in $CDCl_3$.

4,6-Bis(triisopropylsilyl)dibenzo[*b*,*d*]furan (4g)

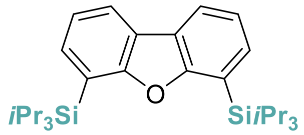

$C_{30}H_{48}OSi_2$
Molecular Weight: 480.88

Prepared according to General Procedure B (2.4 eq. of NaTMP, 2.4 eq. of *i*Pr_3SiCl and 2 eq. of PMDETA) and purified by flash column chromatography in silica gel and hexane, compound **4g** was isolated as a white solid (229 mg, 95 % yield).

^{1}H NMR (300 MHz, $CDCl_3$): δ 8.07 (dd, *J* = 7.6, 1.4 Hz, 2H), 7.66, (dd, *J* = 7.3, 1.4 Hz, 2H), 7.42 (t, *J* = 7.5 Hz, 2H), 1.88 (sept, *J* = 7.6 Hz, 6H), 1.26 (d, *J* = 7.6 Hz, 36H).

^{13}C{^{1}H} NMR (75 MHz, $CDCl_3$): δ 161.2, 134.7, 123.5, 122.2, 121.3, 118.7, 19.1, 12.3.

HRMS (EI) m/z: $[M]^+$ Calculated for $C_{30}H_{48}OSi_2$ 480.3238. Found 480.3247.

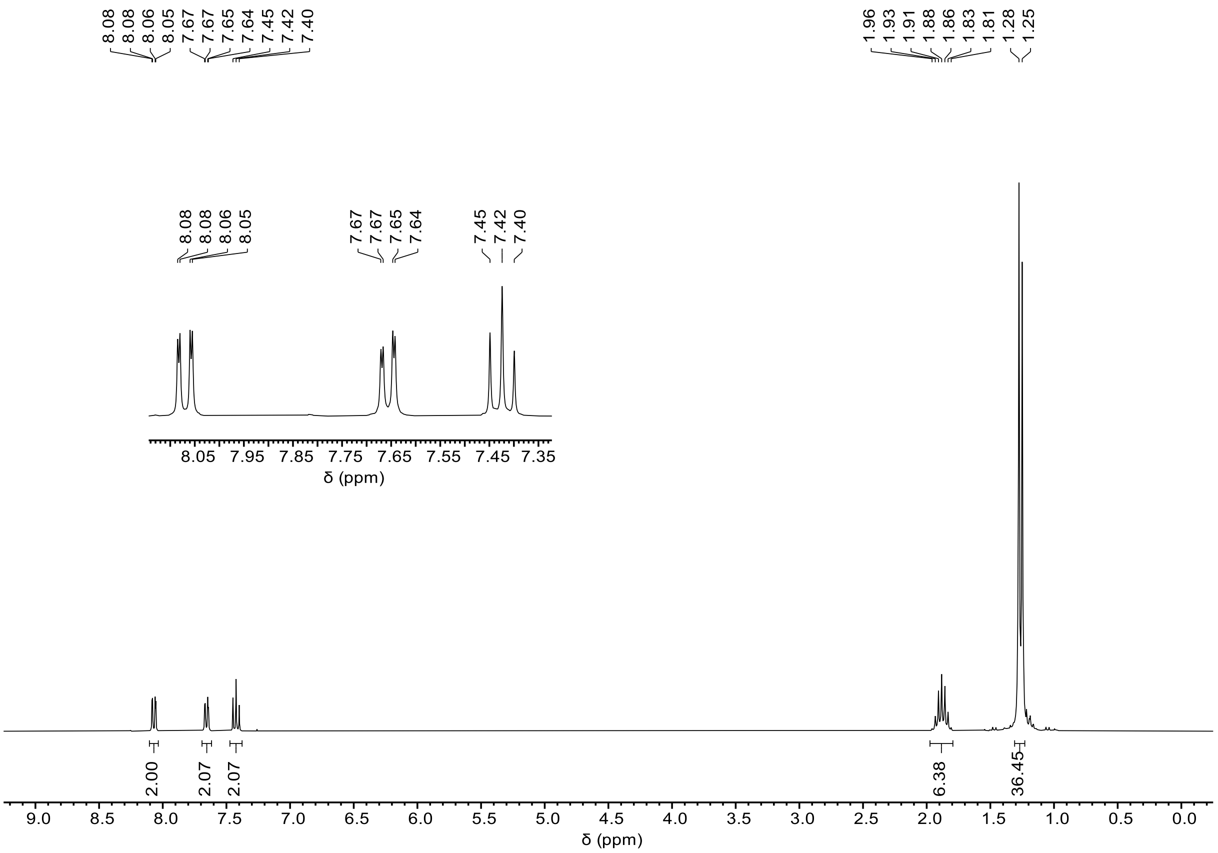

Figure S84. ^{1}H NMR of **4g** in $CDCl_3$.

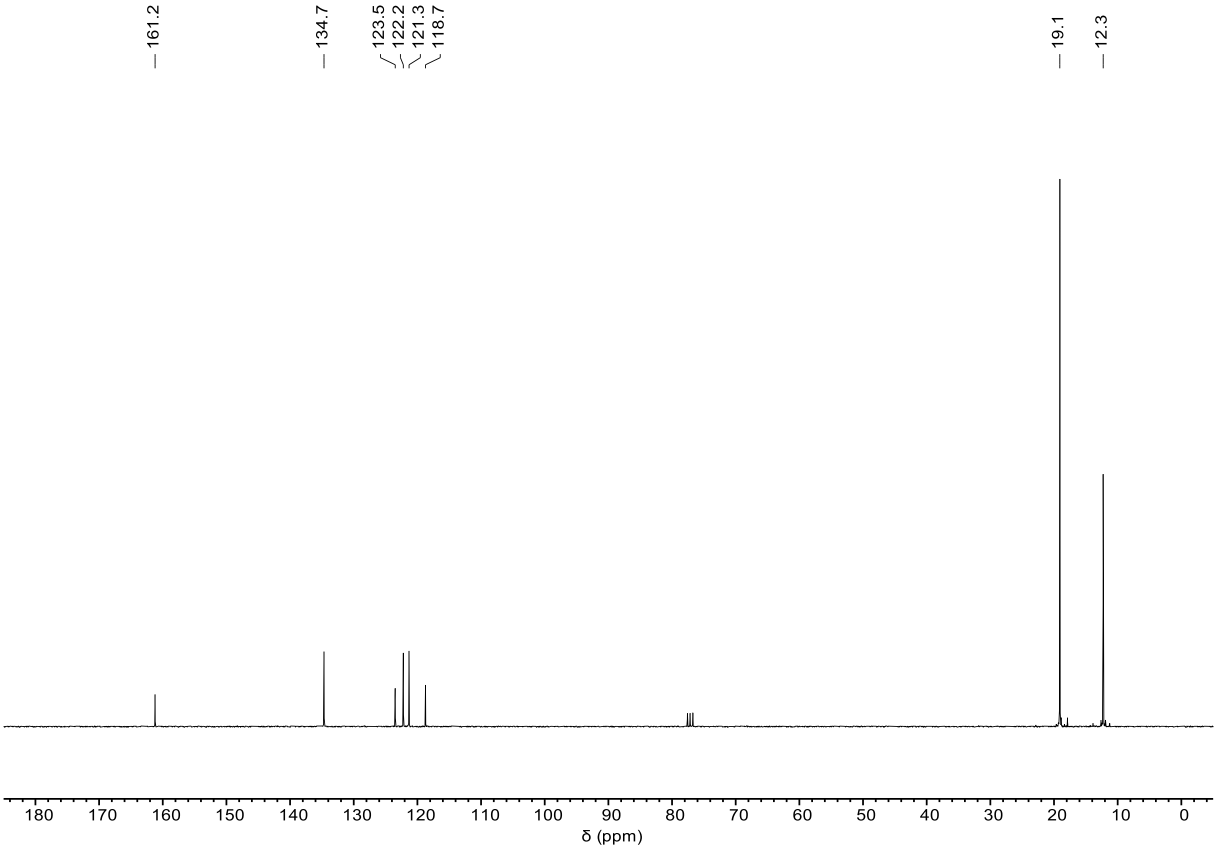

Figure S85. $^{13}C\{^1H\}$ NMR of **4g** in $CDCl_3$.

2,5-Bis(triisopropylsilyl)thiophene (4h)

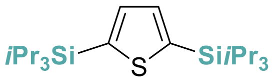

$C_{22}H_{44}SSi_2$
Molecular Weight: 396.82

Prepared according to General Procedure B (2.4 eq. of NaTMP, 2.4 eq. of *i*Pr_3SiCl and 2 eq. of PMDETA) and purified by flash column chromatography in silica gel and hexane, compound **4h** was isolated as a white solid (184 mg, 93 % yield).

^{1}H NMR (300 MHz, $CDCl_3$): *δ* 7.40 (s, 2H), 1.48 – 1.31 (m, 6H), 1.15 (d, *J* = 7.5 Hz, 36H).

^{13}C{^{1}H} NMR (75 MHz, $CDCl_3$): *δ* 139.6, 136.2, 18.9, 12.2.

HRMS (EI) m/z: $[M]^+$ Calculated for $C_{22}H_{44}SSi_2$ 396.2697. Found 396.2705.

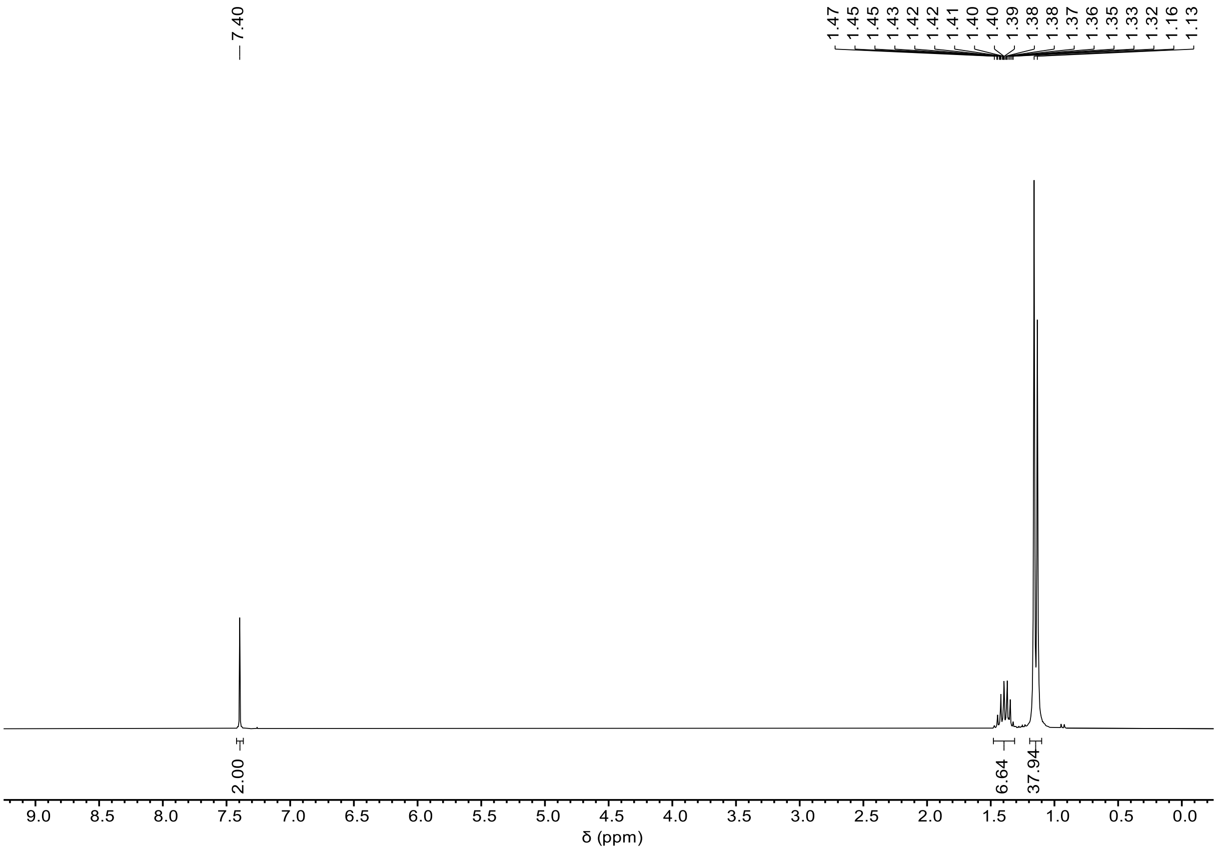

Figure S86. ^{1}H NMR of **4h** in $CDCl_3$.

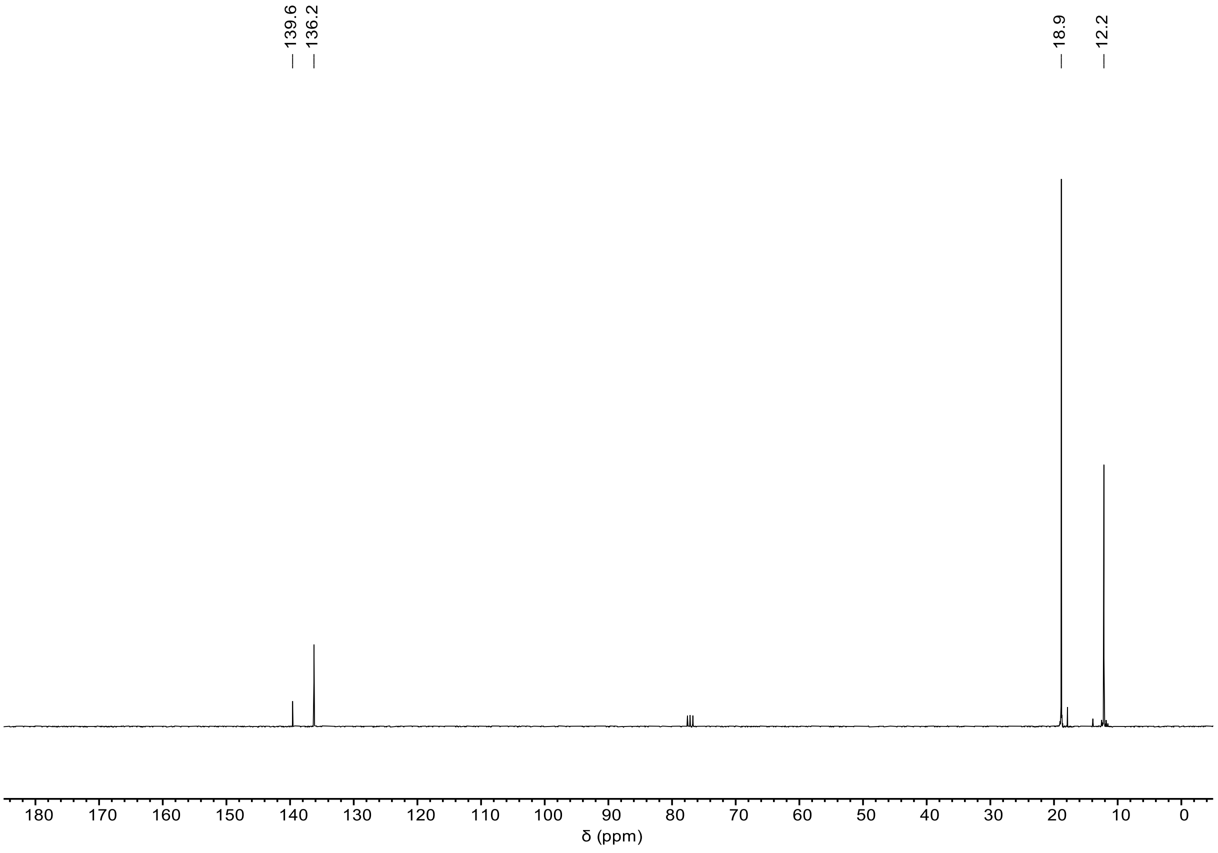

Figure S87. $^{13}C\{^{1}H\}$ NMR of **4h** in $CDCl_3$.

1,1'-Bis(Triisopropylsilyl)ferrocene (4i)

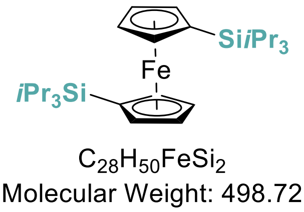

Prepared according to General Procedure B (2.4 eq. of NaTMP, 2.4 eq. of *i*Pr_3SiCl and 2 eq. of PMDETA) and purified by flash column chromatography in silica gel and hexane, compound **4i** was isolated as an orange solid (220 mg, 88 % yield).

^{1}H NMR (300 MHz, $CDCl_3$): *δ* 4.38 – 4.31 (m, 4H), 4.11 – 4.04 (m, 4H), 1.33 – 1.20 (m, 6H), 1.15 (d, *J* = 6.8 Hz, 36H).

^{13}C{^{1}H} NMR (75 MHz, $CDCl_3$): *δ* 74.8, 71.3, 67.8, 19.1, 11.9.

HRMS (ESI+) m/z: $[M]^+$ Calculated for $C_{28}H_{50}FeSi_2$ 498.2795. Found 498.2794.

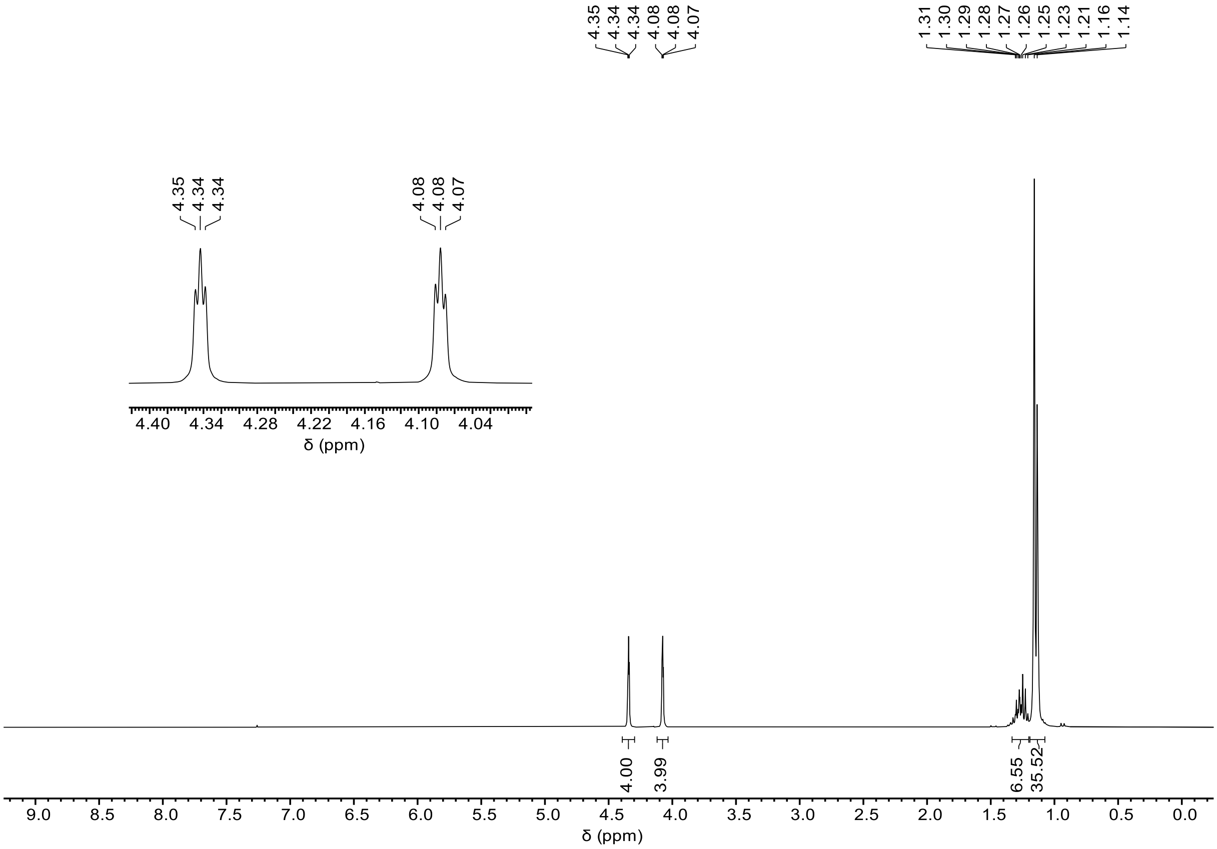

Figure S88. ^{1}H NMR of **4i** in $CDCl_3$.

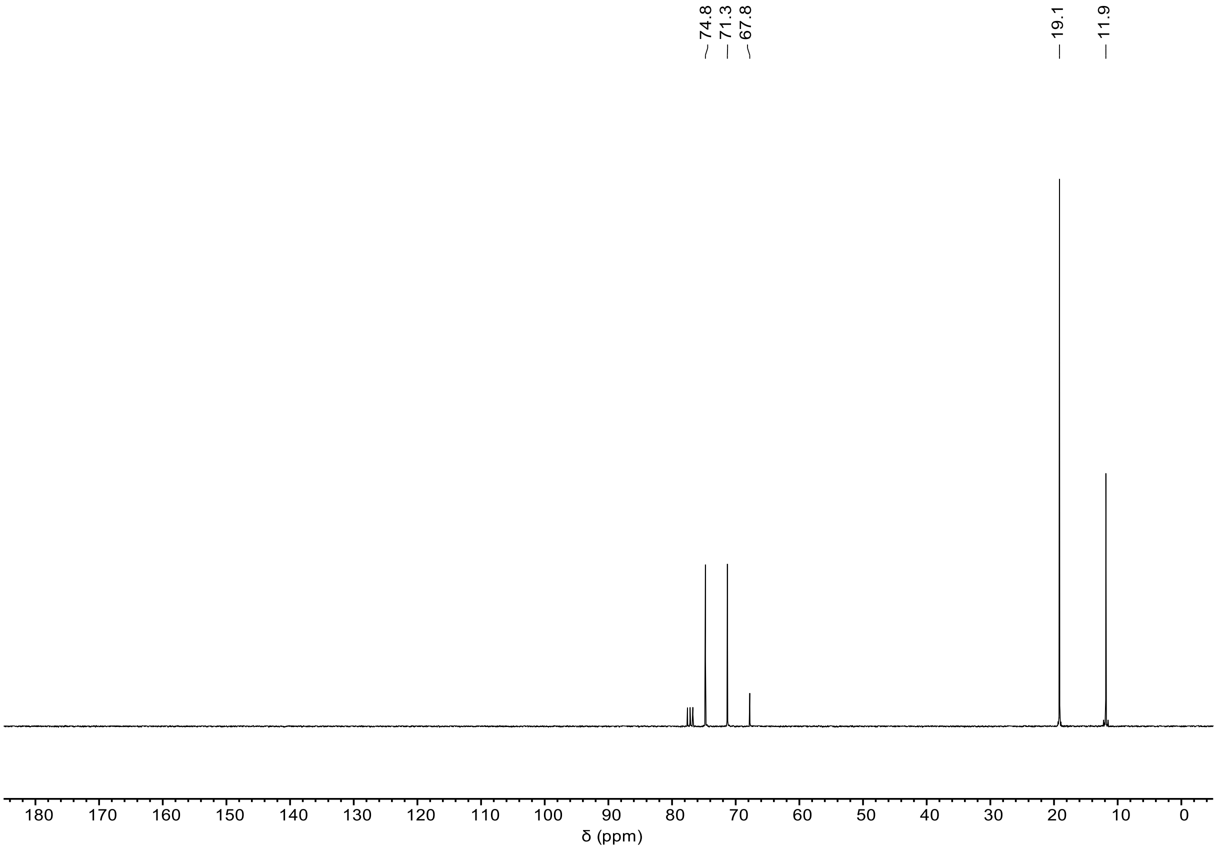

Figure S89. $^{13}C\{^1H\}$ NMR of **4i** in $CDCl_3$.

(2,5-Dimethoxy-1,4-phenylene)bis(triisopropylsilane) (4j)

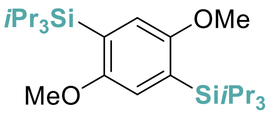

$C_{26}H_{50}O_2Si_2$
Molecular Weight: 450.85

Prepared according to General Procedure B (3 eq. of NaTMP, 3 eq. of *i*Pr_3SiCl and 3 eq. of PMDETA) extracted with dichloromethane and purified by filtration through silica, compound **4j** was isolated as a white solid (220 mg, 98 % yield).

^{1}H NMR (300 MHz, $CDCl_3$): δ 6.85 (s, 2H), 3.71 (s, 6H), 1.43 (sept, J = 7.6 Hz, 6H), 1.08 (d, J = 7.6 Hz, 36H).

^{13}C{^{1}H} NMR (75 MHz, $CDCl_3$): δ 158.4, 124.9, 117.6, 55.2, 19.2, 11.9.

HRMS (ESI+) m/z: $[M+H]^+$ Calculated for $C_{26}H_{51}O_2Si_2$ 451.3422. Found 451.3421.

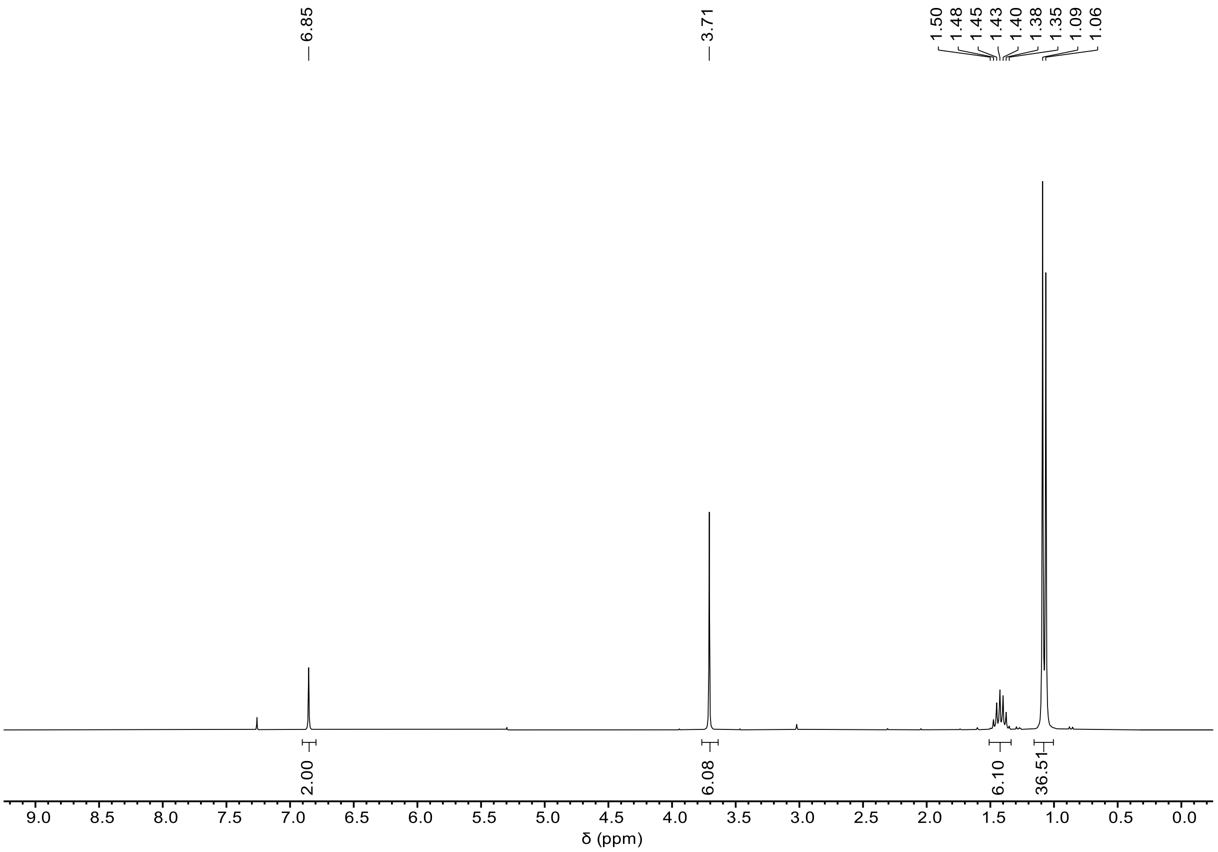

Figure S90. ^{1}H NMR of **4j** in $CDCl_3$.

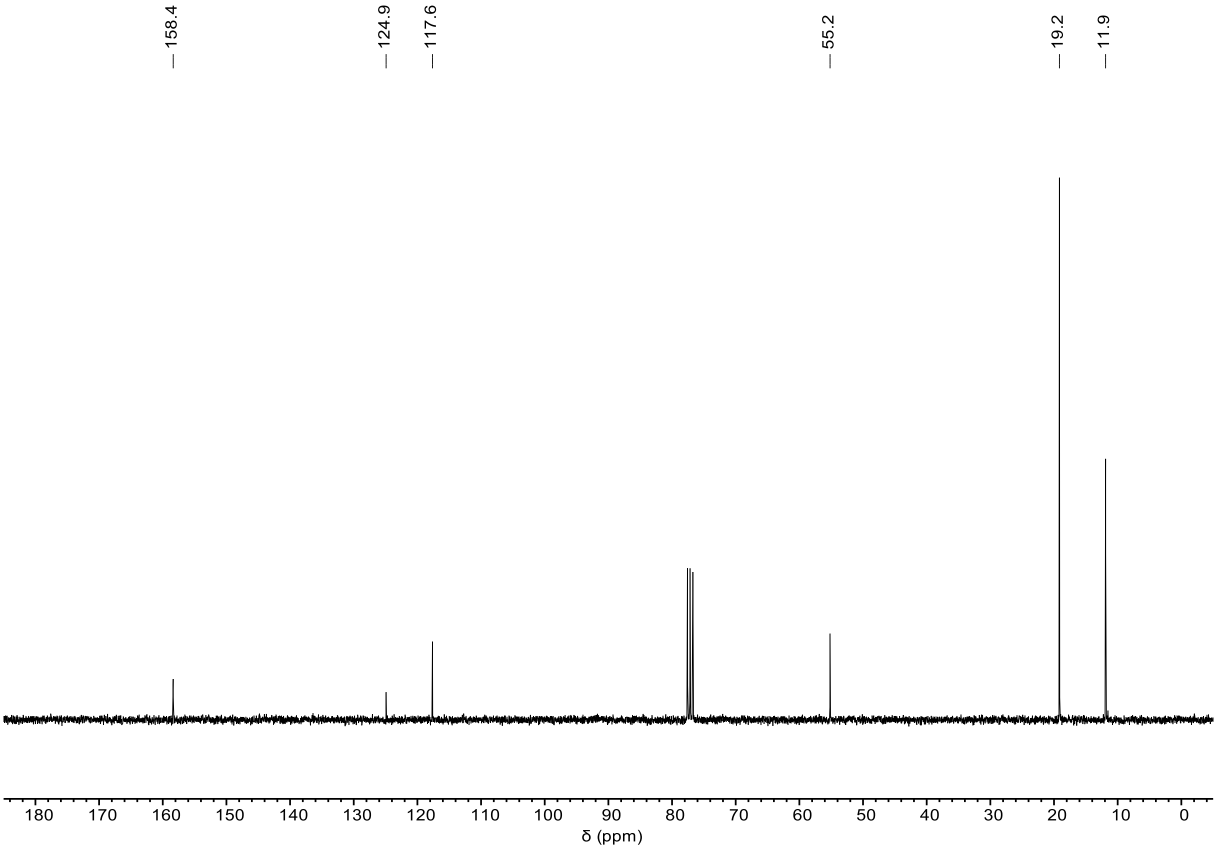

Figure S91. $^{13}C\{^{1}H\}$ NMR of **4j** in $CDCl_3$.

(2-Methoxy-1,4-phenylene)bis(triisopropylsilane) (4k)

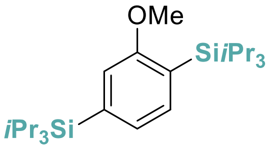

$C_{25}H_{48}OSi_2$
Molecular Weight: 420.83

Prepared according to General Procedure B (3 eq. of NaTMP, 3 eq. of iPr$_3$SiCl and 3 eq. of PMDETA) and purified by flash column chromatography in silica gel and hexane, compound **4k** was isolated as a white solid (174 mg, 83 % yield).

^{1}H NMR (300 MHz, $CDCl_3$): δ 7.27 (d, J = 7.1 Hz, 1H), 6.96 (d, J = 7.1 Hz, 1H), 6.82 (s, 1H), 3.66 (s, 3H), 1.41 – 1.25 (m, 6H), 1.08 – 0.95 (m, 36H).

^{13}C{^{1}H} NMR (75 MHz, $CDCl_3$): δ 163.8, 137.3, 135.9, 127.3, 123.7, 115.7, 54.5, 19.2, 18.9, 11.9, 11.1.

HRMS (EI) m/z: [M-iPr]$^+$ (from fragmentation) Calculated for $C_{22}H_{41}OSi_2$ 377.2690. Found 377.2693.

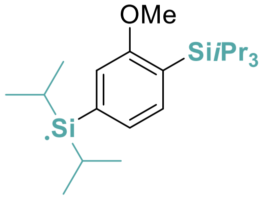

$C_{22}H_{41}OSi_2^{\bullet}$
Exact Mass: 377.27

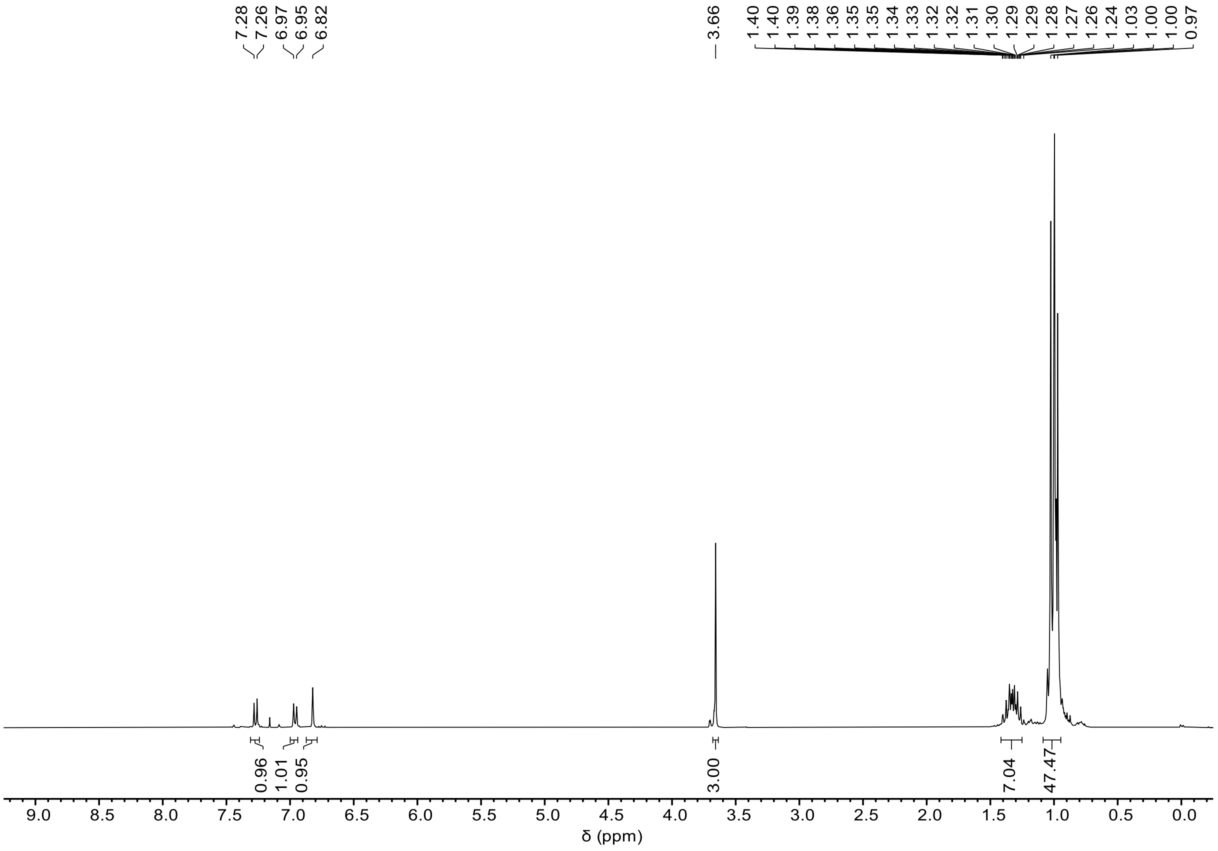

Figure S92. ^{1}H NMR of **4k** in $CDCl_3$.

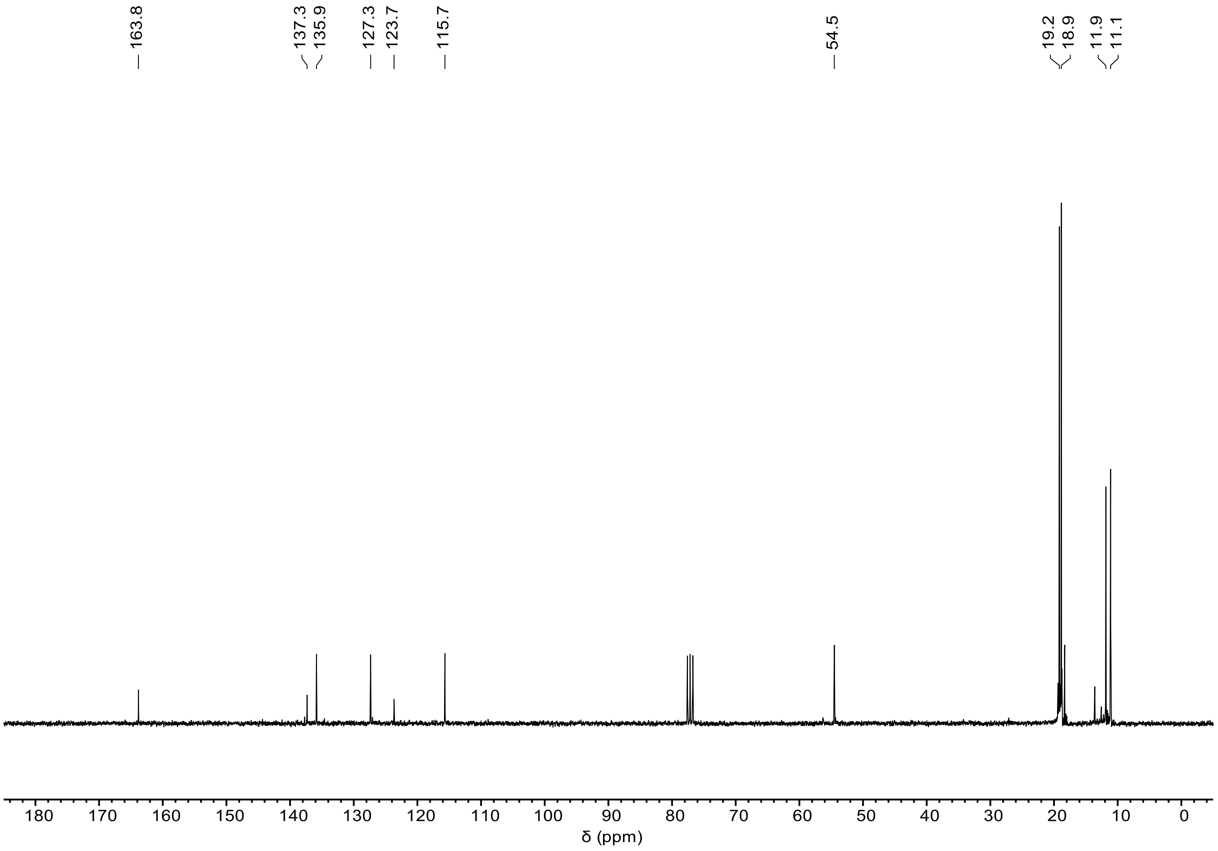

Figure S93. $^{13}C\{^{1}H\}$ NMR of **4k** in $CDCl_3$.

2,6-Bis(triisopropylsilyl)naphthalene (4l)

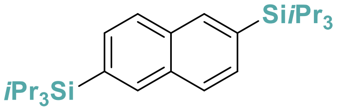

$C_{28}H_{48}Si_2$
Molecular Weight: 440.86

Prepared according to General Procedure B (3 eq. of NaTMP, 3 eq. of *i*Pr_3SiCl and 3 eq. of PMDETA), extracted with dichloromethane and purified by filtration through silica, compound **4l** was isolated as a white solid (210 mg, 95 % yield).

^{1}H NMR (300 MHz, $CDCl_3$): *δ* 7.98 (s, 2H), 7.81 (d, *J* = 8.1 Hz, 2H), 7.57 (d, *J* = 8.1 Hz, 2H), 1.51 (sept, *J* = 7.5 Hz, 6H), 1.12 (d, *J* = 7.5 Hz, 36H).

^{13}C{^{1}H} NMR (75 MHz, $CDCl_3$): *δ* 135.9, 133.2, 133.1, 131.7, 126.8, 18.8, 11.1.

HRMS (EI) m/z: $[M]^+$ Calculated for $C_{28}H_{48}Si_2$ 440.3289. Found 440.3299.

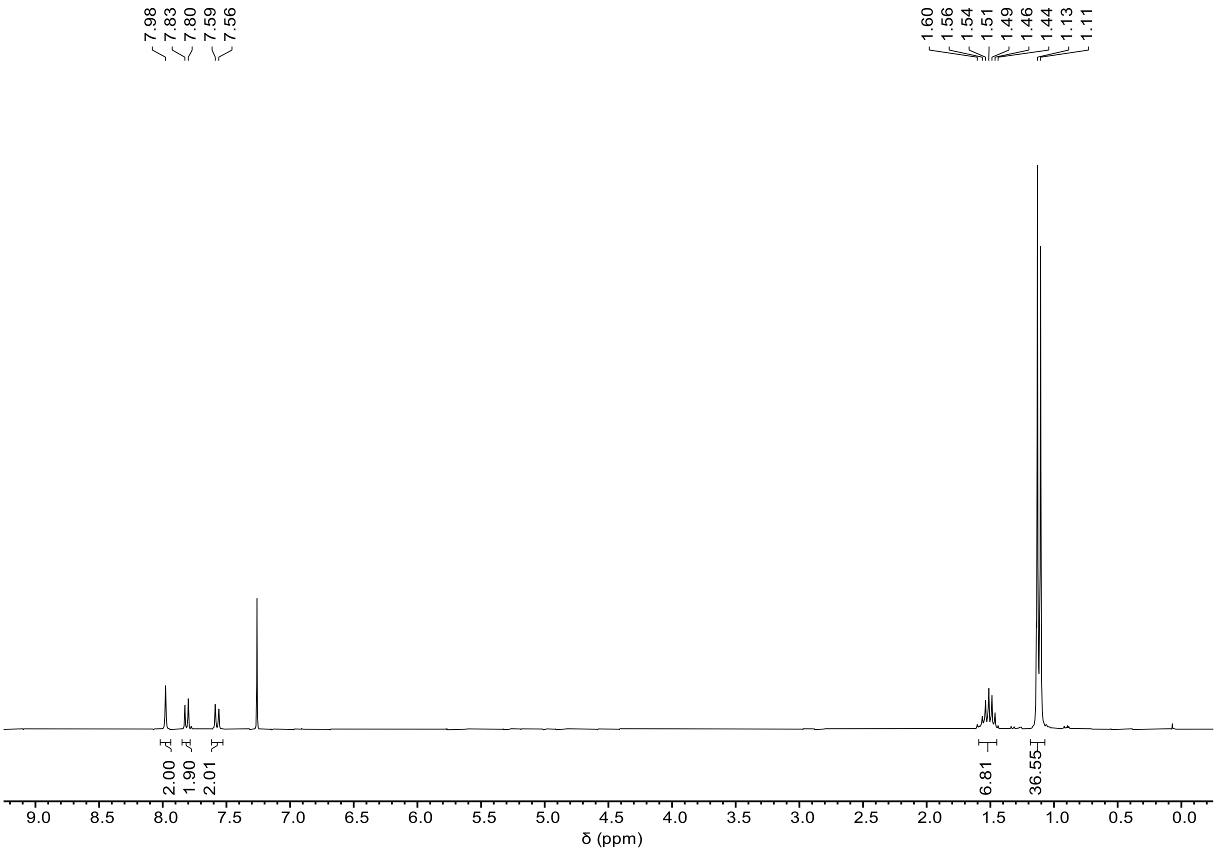

Figure S94. ^{1}H NMR of **4l** in $CDCl_3$.

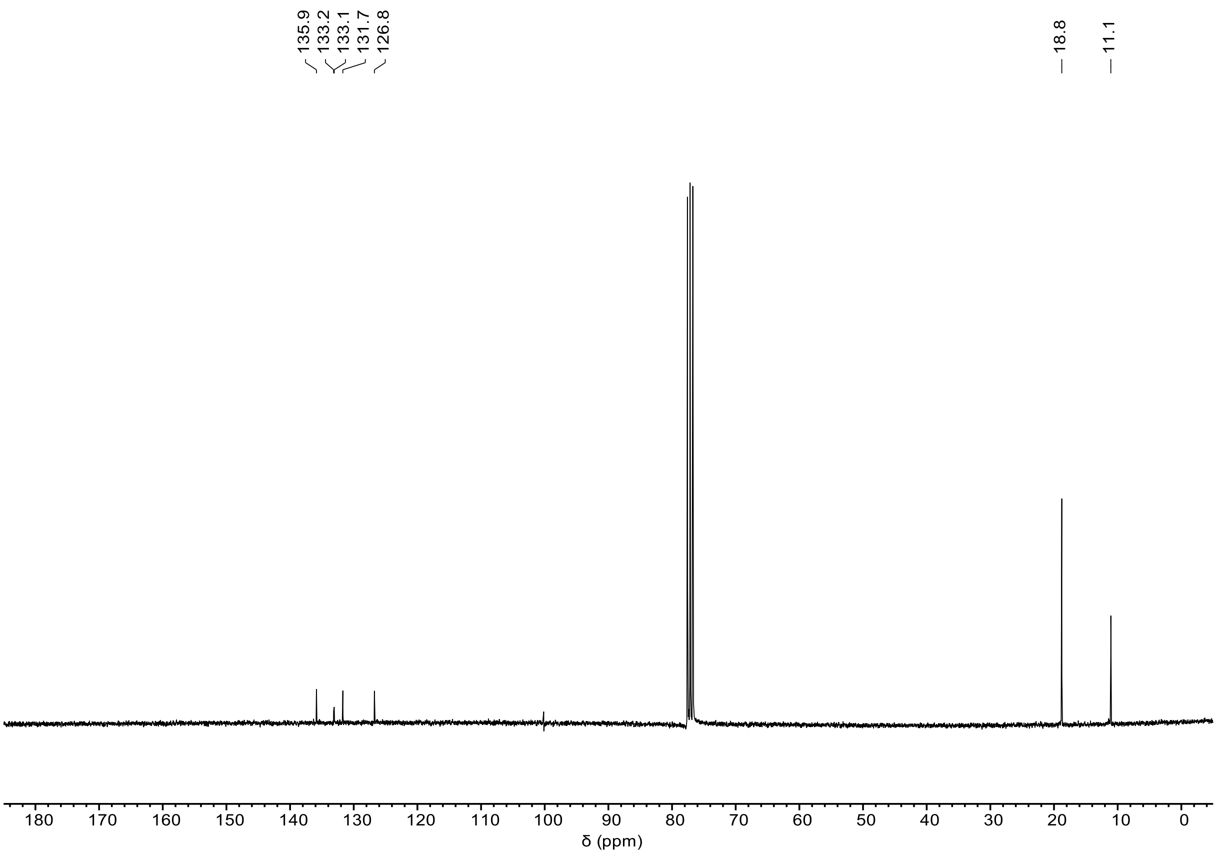

Figure S95. $^{13}C\{^1H\}$ NMR of **4l** in $CDCl_3$.

2,7-Bis(triisopropylsilyl)pyrene (4m)

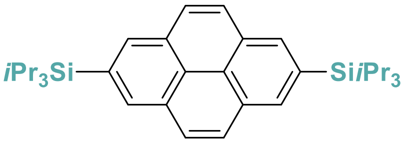

$C_{34}H_{50}Si_2$
Molecular Weight: 514.94

Prepared according to General Procedure B (4 eq. of NaTMP, 4 eq. of *i*Pr_3SiCl and 3 eq. of PMDETA), extracted with dichloromethane and purified by filtration through silica, compound **4m** was isolated as a white solid (152 mg, 59 % yield).

^{1}H NMR (300 MHz, $CDCl_3$): δ 8.28 (s, 4H), 8.07 (s, 4H), 1.66 (sept, J = 7.5 Hz, 6H), 1.17 (d, J = 7.5 Hz, 36H).

^{13}C{^{1}H} NMR (75 MHz, $CDCl_3$): δ 132.9, 131.9, 130.4, 127.5, 125.0, 18.9, 11.2.

HRMS (EI) m/z: $[M]^+$ Calculated for $C_{34}H_{50}Si_2$ 514.3451. Found 514.3454.

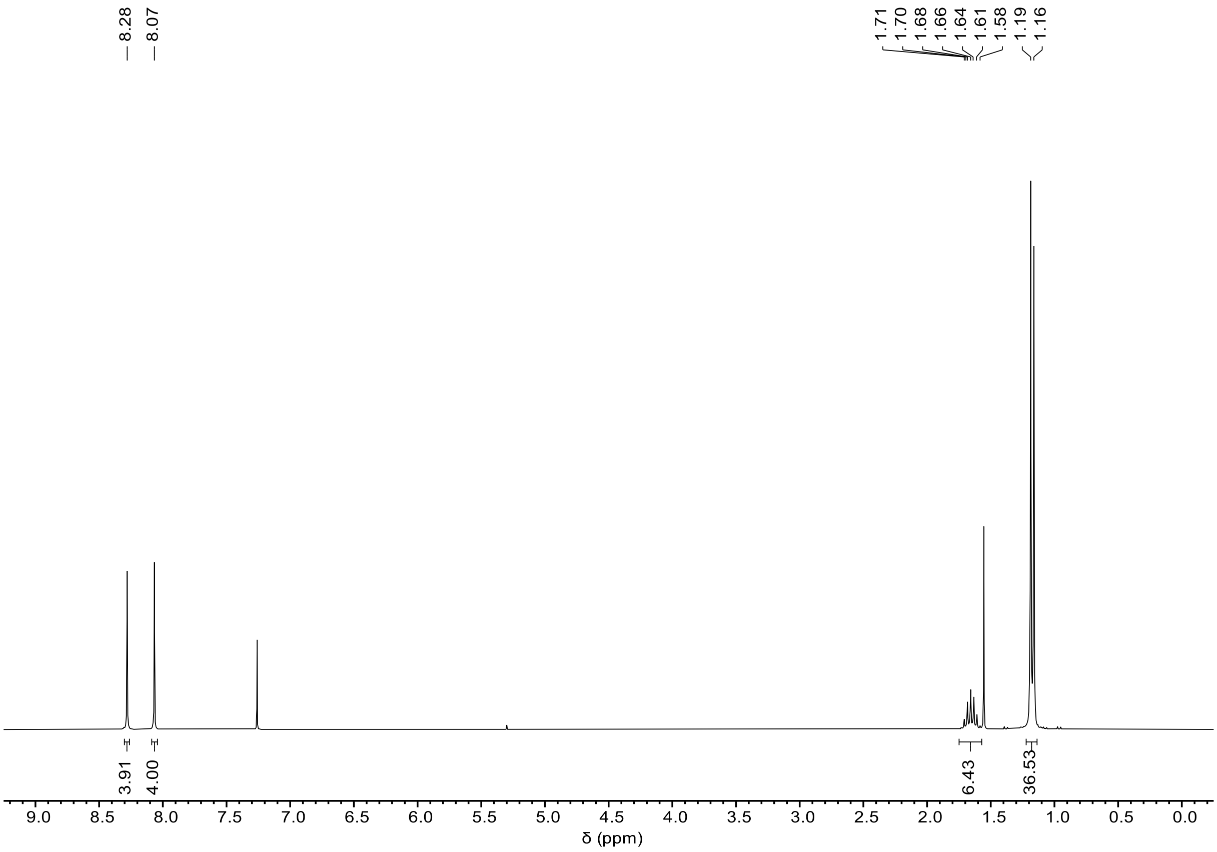

Figure S96. ^{1}H NMR of **4m** in $CDCl_3$.

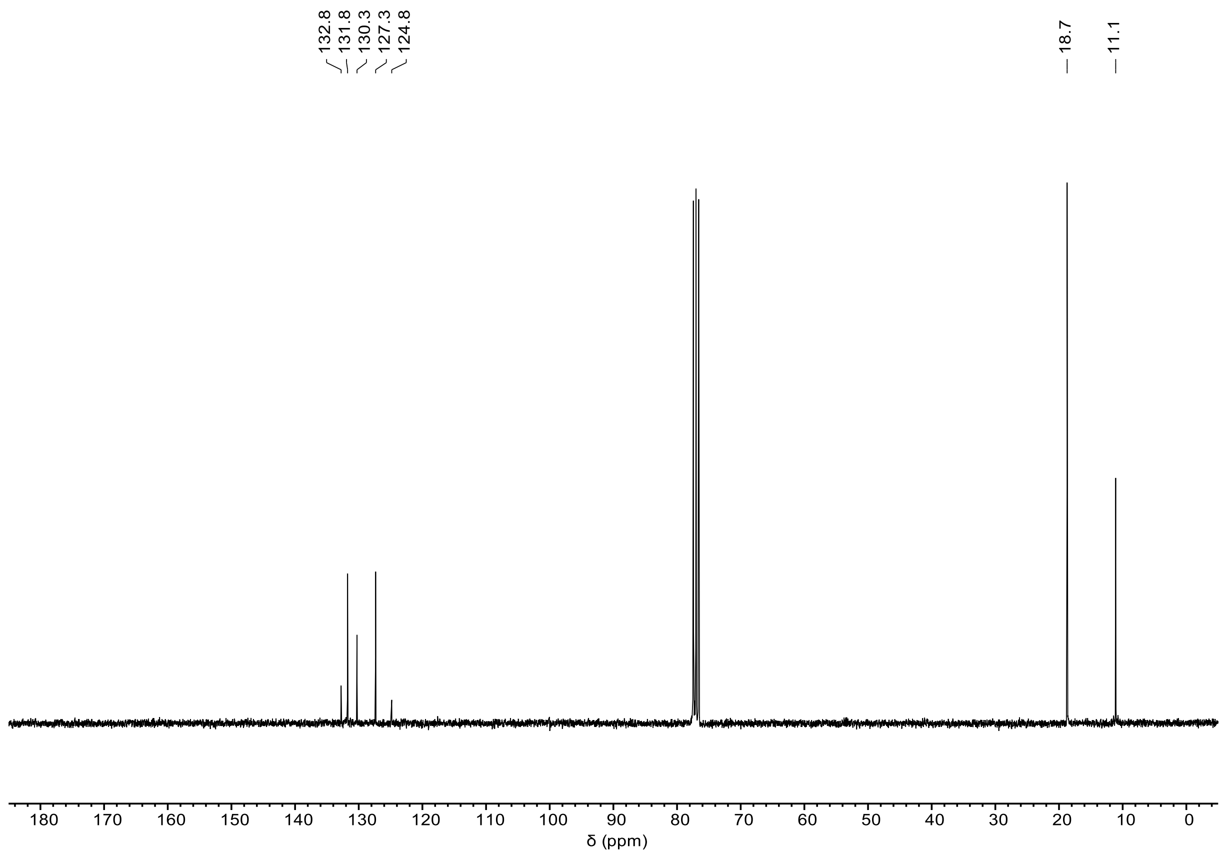

Figure S97. $^{13}C\{^1H\}$ NMR of **4m** in $CDCl_3$.

(4-Methoxy-3-(triisopropylsilyl)benzyl)triisopropylsilane (4n)

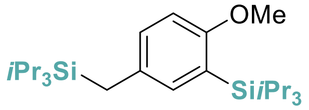

$C_{26}H_{50}OSi_2$
Molecular Weight: 434.86

Prepared according to General Procedure B (3 eq. of NaTMP, 3 eq. of *i*Pr_3SiCl and 3 eq. of PMDETA) and purified by flash column chromatography in silica gel and hexane, compound **4n** was isolated as a white solid (211 mg, 97 % yield).

^{1}H NMR (300 MHz, $CDCl_3$): *δ* 7.17 (d, *J* = 2.3 Hz, 1H), 7.08 (dd, *J* = 8.3 , 2.3 Hz, 1H), 6.72 (d, *J* = 8.3 Hz, 1H), 3.74 (s, 3H), 2.17 (s, 2H), 1.46 (sept, *J* = 7.5 Hz, 3H), 1.20 – 1.13 (m, 3H), 1.10 (d, *J* = 7.5 Hz, 18H), 1.05 (d, *J* = 5.9 Hz, 18H).

^{13}C{^{1}H} NMR (75 MHz, $CDCl_3$): *δ* 161.8, 136.9, 131.9, 130.1, 123.0, 109.4, 54.6, 19.1, 18.8, 17.8, 11.9, 11.2.

HRMS (ESI+) m/z: $[M+Na]^+$ Calculated for $C_{26}H_{50}ONaSi_2$ 457.3292. Found 457.3294.

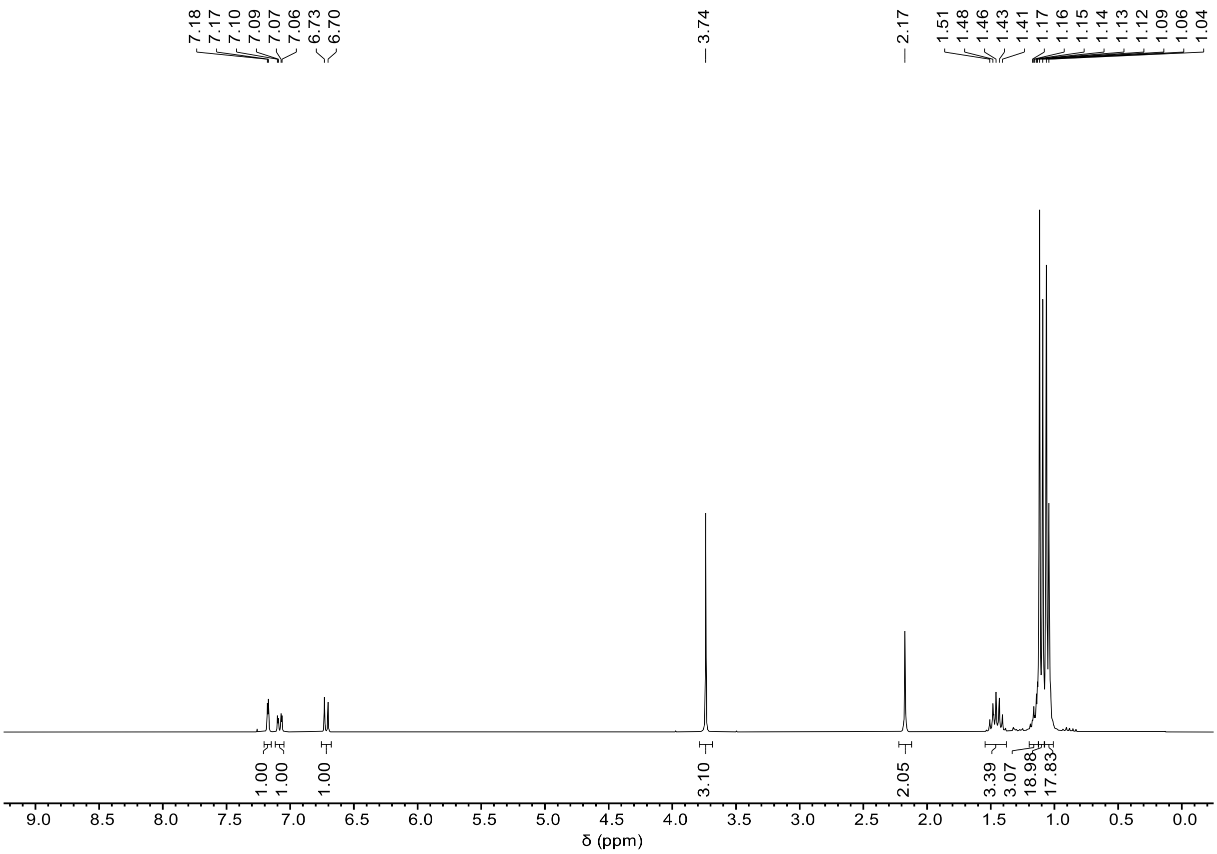

Figure S98. ^{1}H NMR of **4n** in $CDCl_3$.

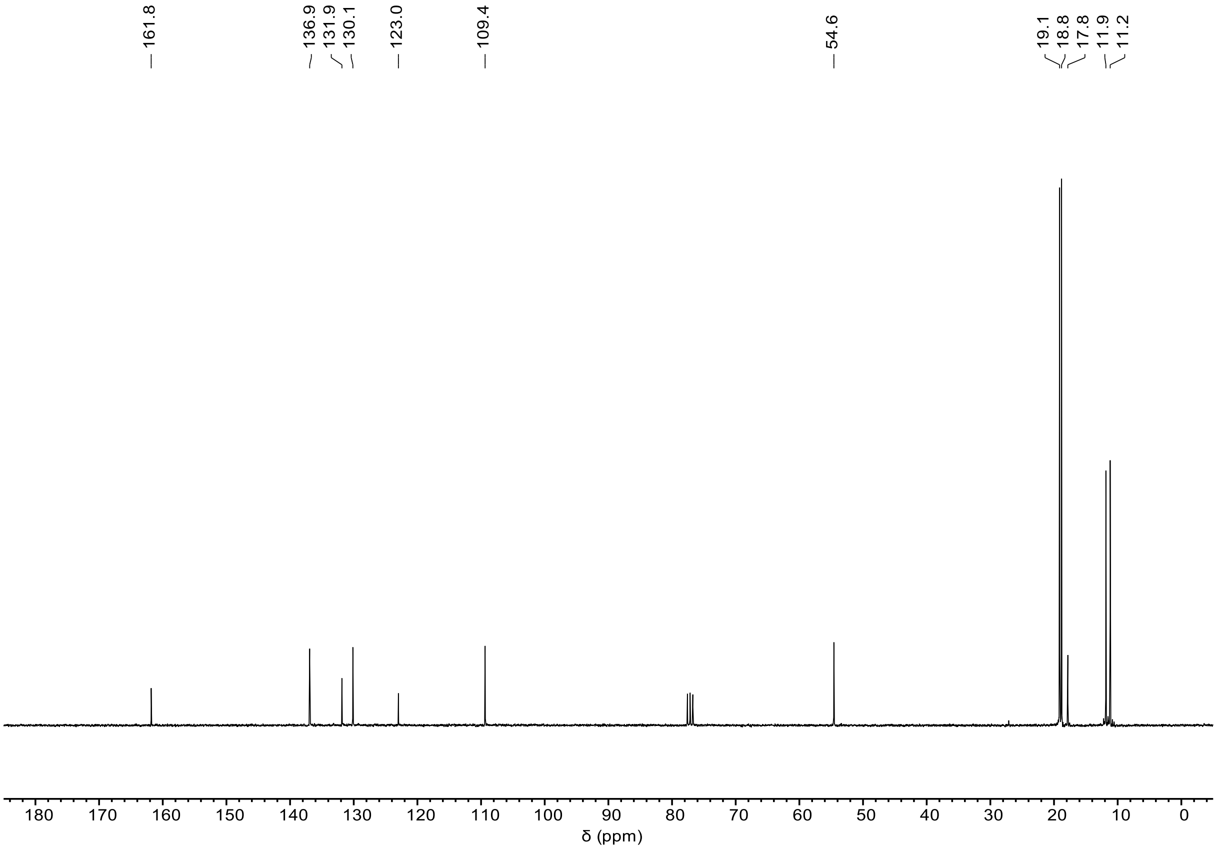

Figure S99. $^{13}C\{^{1}H\}$ NMR of **4n** in $CDCl_3$.

Triisopropyl((2-(triisopropylsilyl)benzo[*b*]thiophen-5-yl)methyl)silane (4o)

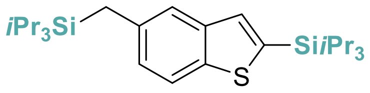

$C_{27}H_{48}SSi_2$
Molecular Weight: 460.91

Prepared according to General Procedure B (2.4 eq. of NaTMP, 2.4 eq. of *i*Pr_3SiCl and 2 eq. of PMDETA) and purified by flash column chromatography in silica gel and hexane, compound **4o** was isolated as a white solid (224 mg, 97 % yield).

^{1}H NMR (300 MHz, $CDCl_3$): *δ* 7.78 (d, *J* = 8.3 Hz, 1H), 7.64 (d, *J* = 1.3 Hz, 1H), 7.48 (s, 1H), 7.18 (dd, *J* = 8.3, 1.5 Hz, 1H), 2.41 (s, 2H), 1.60 – 1.42 (m, 3H), 1.28 – 1.23 (m, 18H), 1.23 – 1.17 (m, 3H), 1.17 – 1.12 (m, 18H).

^{13}C{^{1}H} NMR (75 MHz, $CDCl_3$): *δ* 141.7, 139.7, 136.7, 136.4, 132.3, 126.0, 122.6, 121.5, 19.1, 18.9, 18.8, 12.1, 11.3.

HRMS (ESI+) m/z: $[M+H]^+$ Calculated for $C_{47}H_{49}SSi_2$ 461.3088. Found 461.3083.

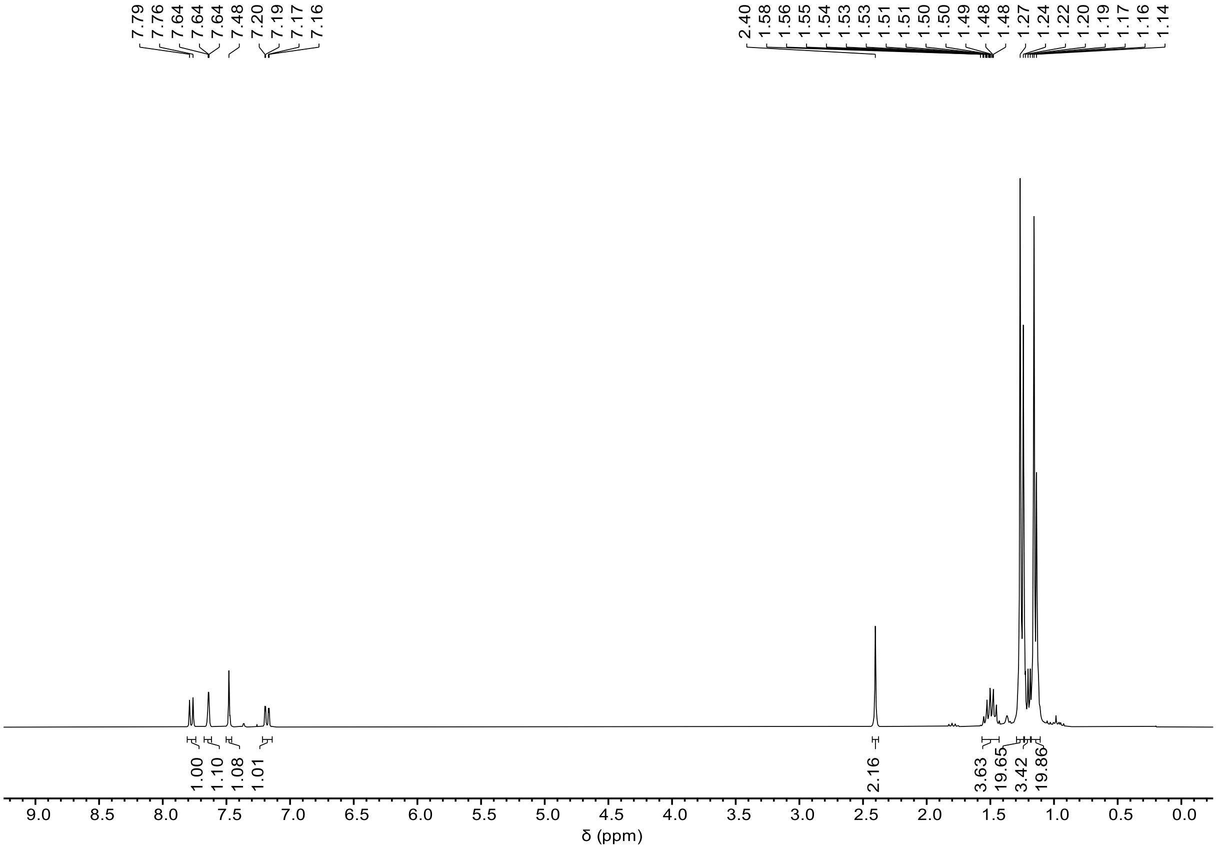

Figure S100. ^{1}H NMR of **4o** in $CDCl_3$.

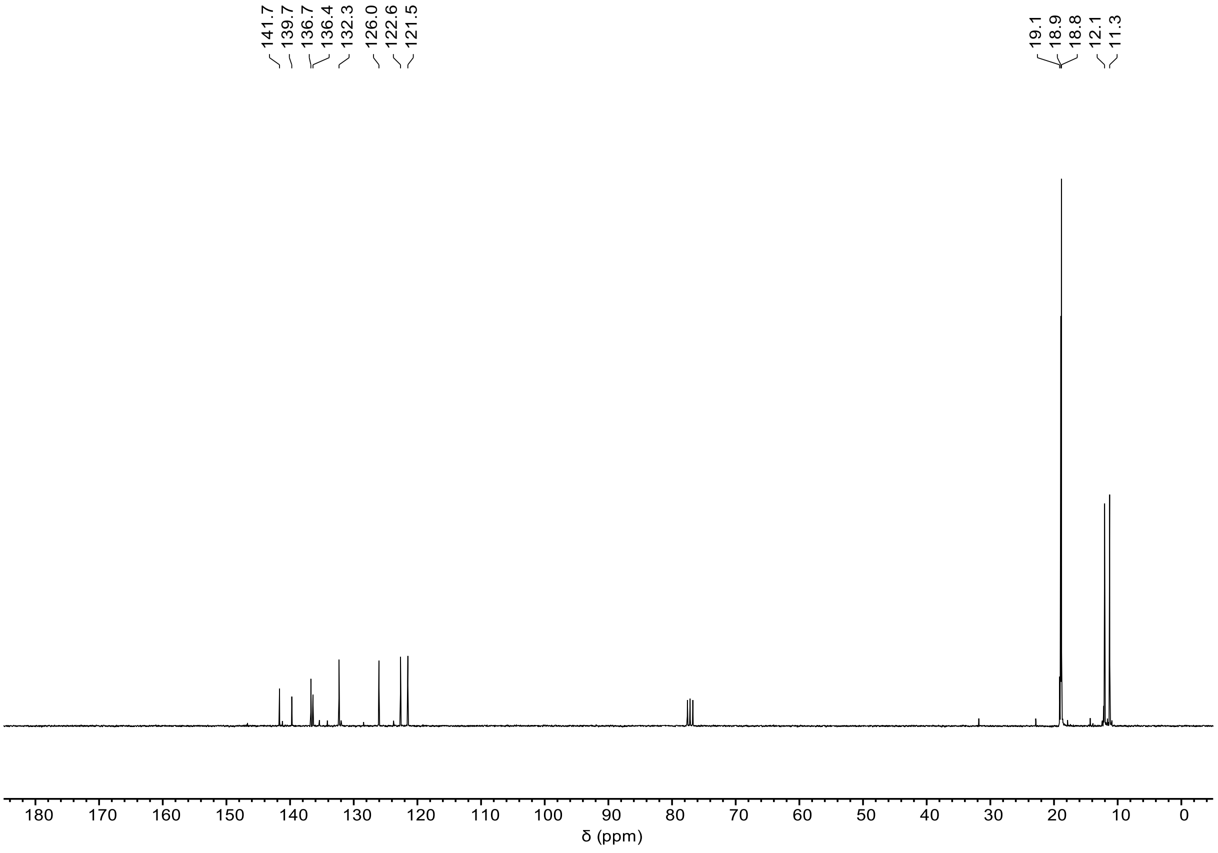

Figure S101. $^{13}C\{^1H\}$ NMR of **4o** in $CDCl_3$.

Benzo[*b*]furan-2,7-diylbis(triisopropylsilane) (4p)

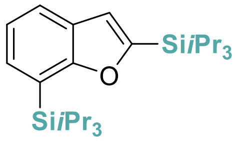

$C_{26}H_{46}OSi_2$
Molecular Weight: 430.82

Prepared according to General Procedure B (2.4 eq. of NaTMP, 2.4 eq. of *i*Pr_3SiCl and 2 eq. of PMDETA) and purified by flash column chromatography in silica gel and hexane, compound **4p** was isolated as a colorless oil (160 mg, 74 % yield).

^{1}**H NMR** (300 MHz, $CDCl_3$): *δ* 7.55 (dd, *J* = 7.6, 1.3 Hz, 1H), 7.32 (dd, *J* = 7.3, 1.3 Hz, 1H), 7.15 (t, *J* = 7.5 Hz, 1H), 6.98 (s, 1H), 1.64 (sept, *J* = 7.4 Hz, 3H), 1.35 (sept, *J* = 7.4 Hz, 3H), 1.08 (d, *J* = 7.4 Hz, 18H), 1.08 (d, *J* = 7.4 Hz, 18H).

^{13}**C{**^{1}**H} NMR** (75 MHz, $CDCl_3$): *δ* 163.4, 159.3, 132.0, 126.8, 122.0, 121.9, 118.5, 117.4, 19.0, 18.8, 11.7, 11.2.

HRMS (EI) m/z: $[M]^+$ Calculated for $C_{26}H_{46}OSi_2$ 430.3082. Found 430.3086.

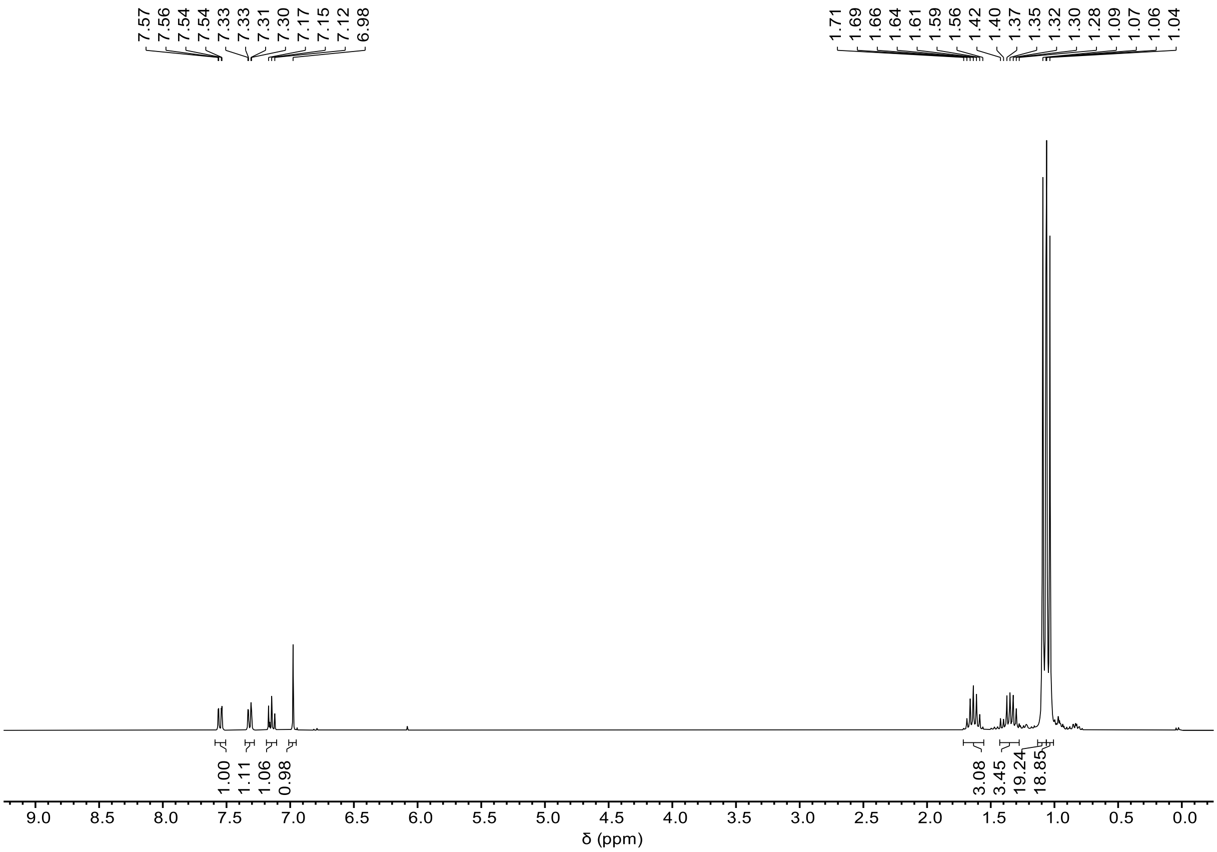

Figure S102. ^{1}H NMR of **4p** in $CDCl_3$.

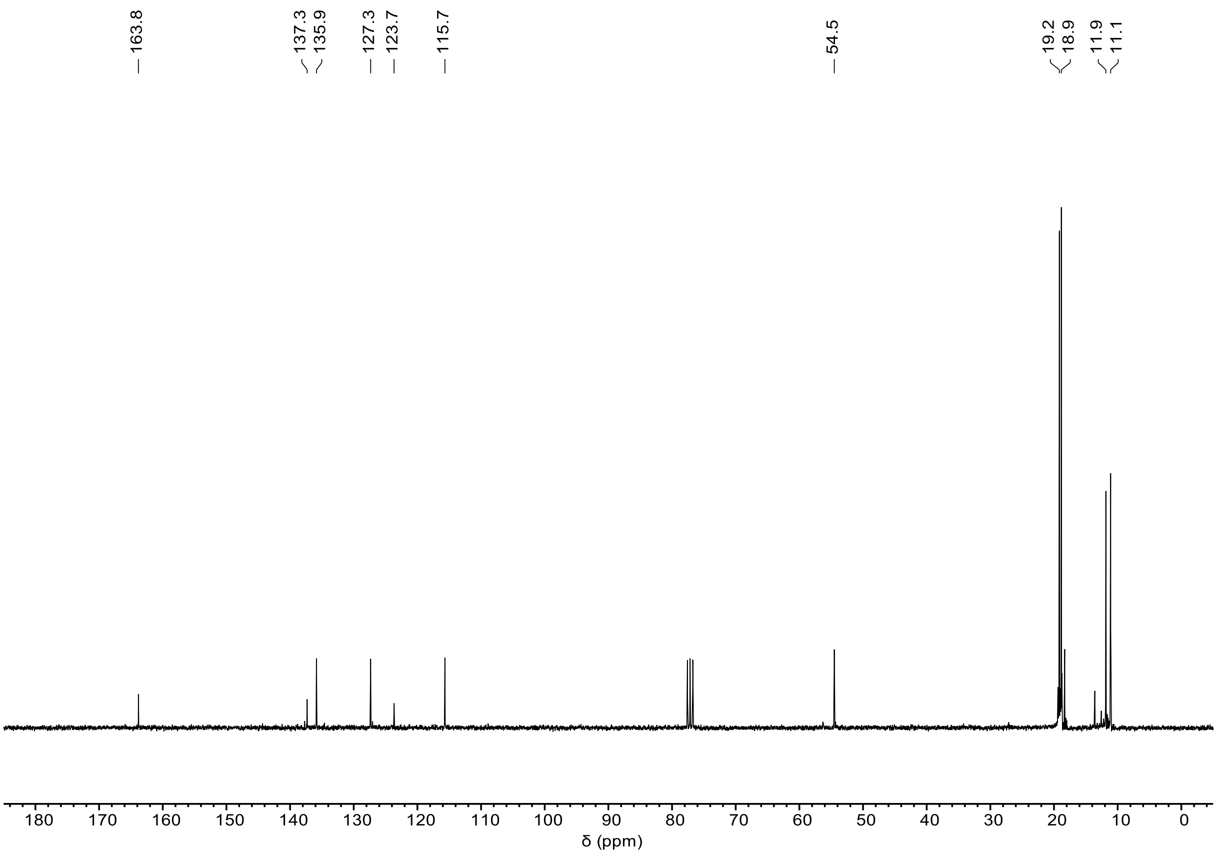

Figure S103. $^{13}C\{^{1}H\}$ NMR of **4p** in $CDCl_3$.

Triisopropyl(4-((triisopropylsilyl)ethynyl)benzyl)silane (4q)

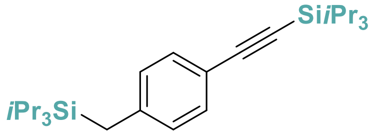

$C_{27}H_{48}Si_2$
Molecular Weight: 428.85

Prepared according to General Procedure B (2.4 eq. of NaTMP, 2.4 eq. of *i*Pr_3SiCl and 2 eq. of PMDETA) and purified by flash column chromatography in silica gel and hexane, compound **4q** was isolated as a colorless oil (178 mg, 83 % yield).

^{1}H NMR (300 MHz, $CDCl_3$): *δ* 7.39 – 7.31 (m, 2H), 7.10 – 7.02 (m, 2H), 2.23 (s, 2H), 1.21 – 1.13 (m, 22H), 1.10 – 1.00 (m, 20H).

$^{13}C\{^1H\}$ NMR (75 MHz, $CDCl_3$): *δ* 142.2, 132.1, 128.5, 119.1, 107.8, 89.1, 19.7, 18.9, 18.8, 11.6, 11.2.

HRMS (EI) m/z: $[M]^+$ Calculated for $C_{27}H_{48}Si_2$ 428.3289. Found 428.3292.

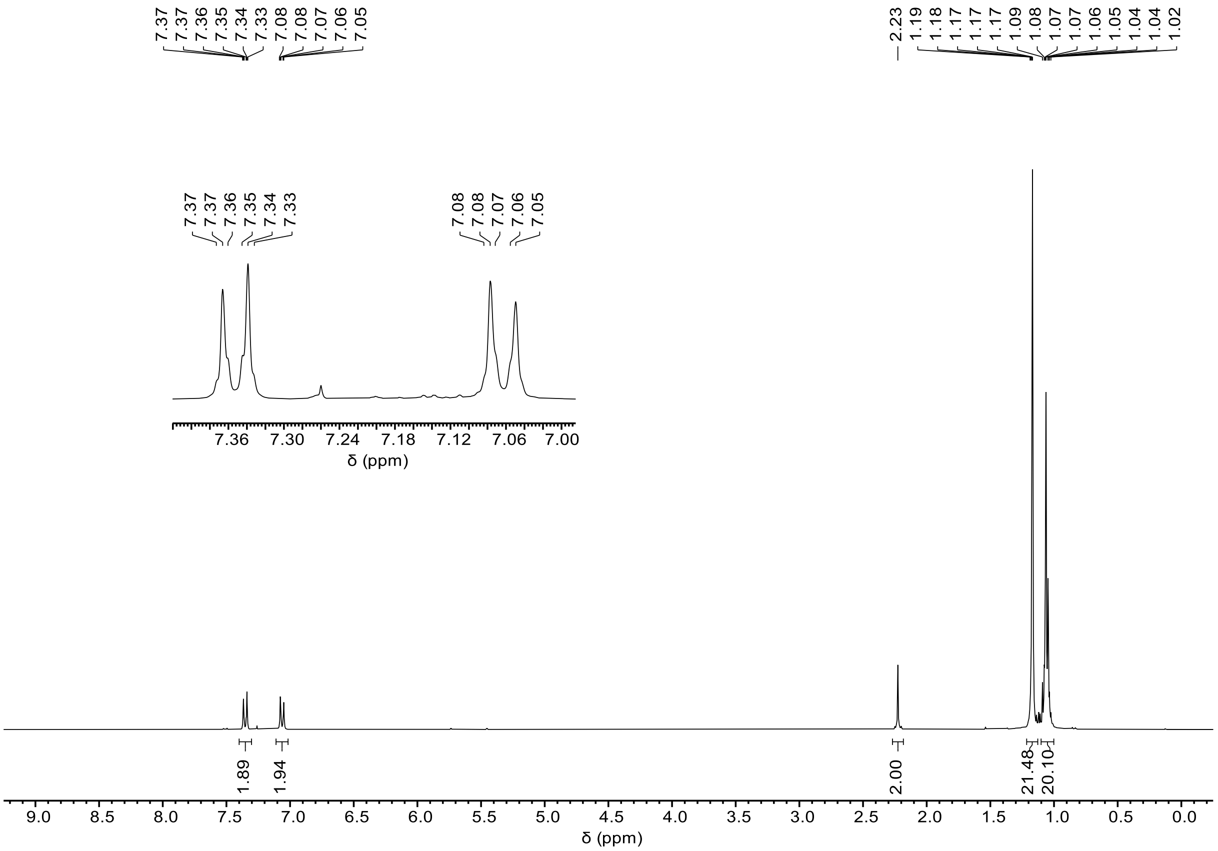

Figure S104. ^{1}H NMR of **4q** in $CDCl_3$.

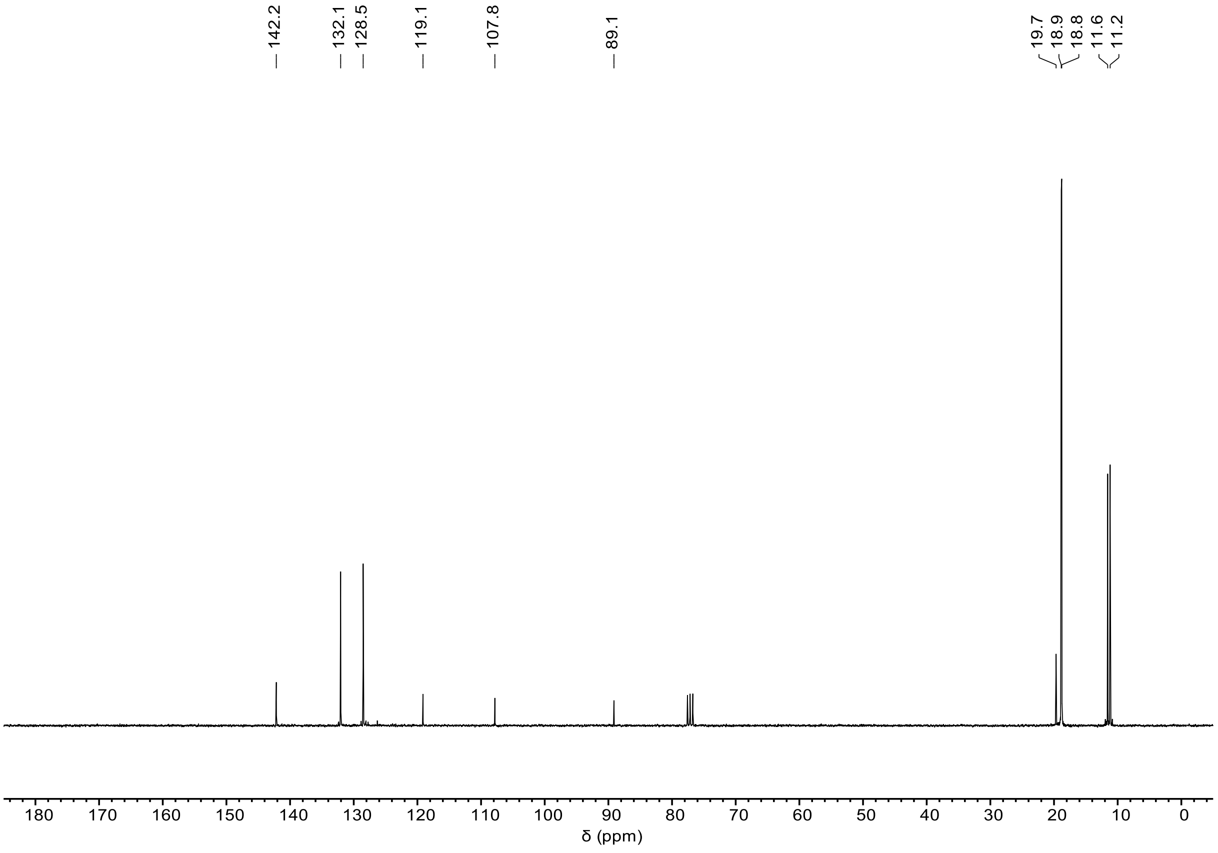

Figure S105. $^{13}C\{^1H\}$ NMR of **4q** in $CDCl_3$.

Regiodivergent Monosilylation of 4-Methylanisole

(4-Methoxybenzyl)triisopropylsilane (4n')

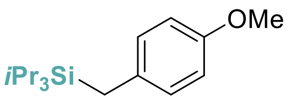

$C_{17}H_{30}OSi$
Molecular Weight: 278.51

Prepared according to a modification of General Procedure A using *i*Pr_3SiCl as silicon electrophile and 0.75 mmol (1.5 eq.) of substrate, and purified by flash column chromatography in silica gel and hexane/EtOAc (98:2), compound **4n'** was isolated as a colorless oil (121 mg, 87 % yield).

^{1}H NMR (300 MHz, $CDCl_3$): δ 6.97 – 6.89 (m, 2H), 6.72 – 6.64 (m, 2H), 3.68 (s, 3H), 2.05 (s, 2H), 1.07 – 0.96 (m, 3H), 0.96 – 0.90 (m, 18H).

^{13}C{^{1}H} NMR (75 MHz, $CDCl_3$): δ 156.6, 132.9, 129.4, 113.8, 55.3, 18.8, 17.8, 11.1.

HRMS (EI) m/z: $[M]^+$ Calculated for $C_{17}H_{30}OSi$ 278.2060. Found 278.2066.

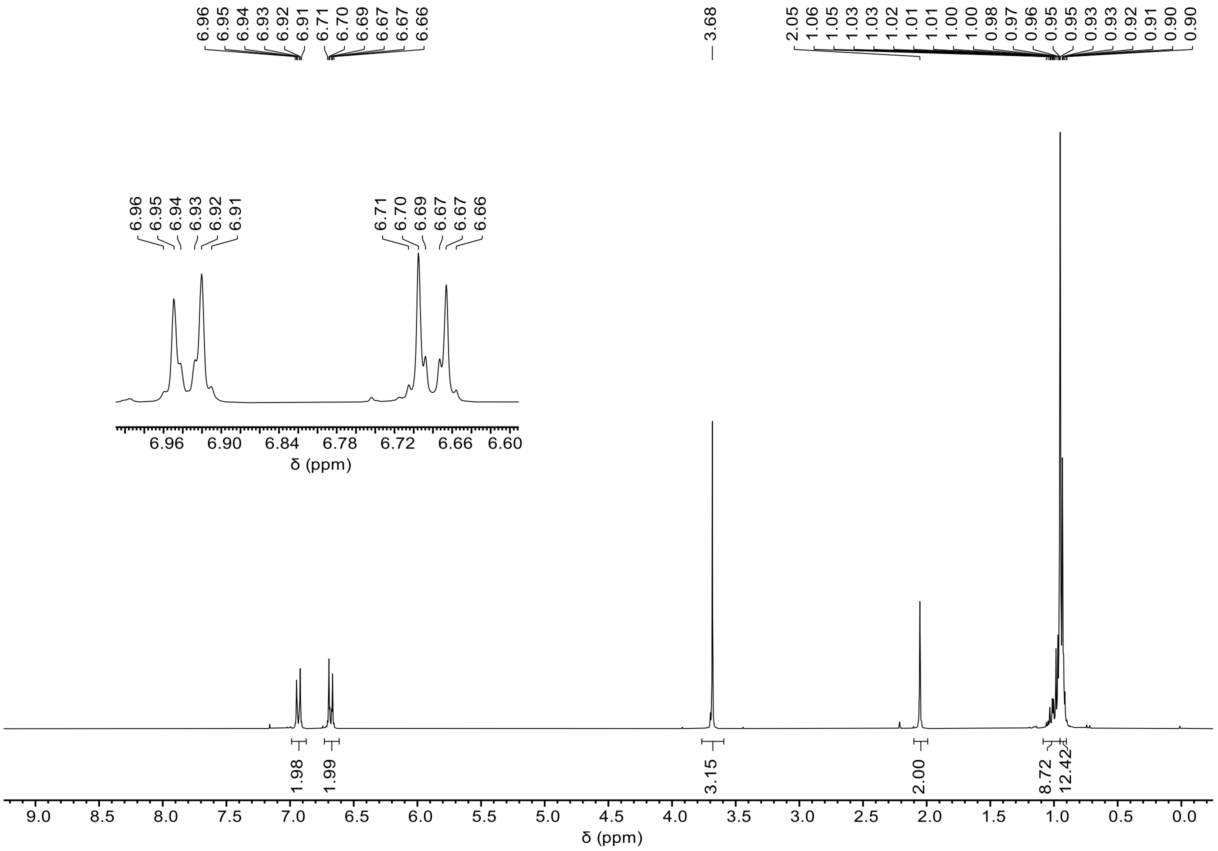

Figure S106. ^{1}H NMR of **4n'** in $CDCl_3$.

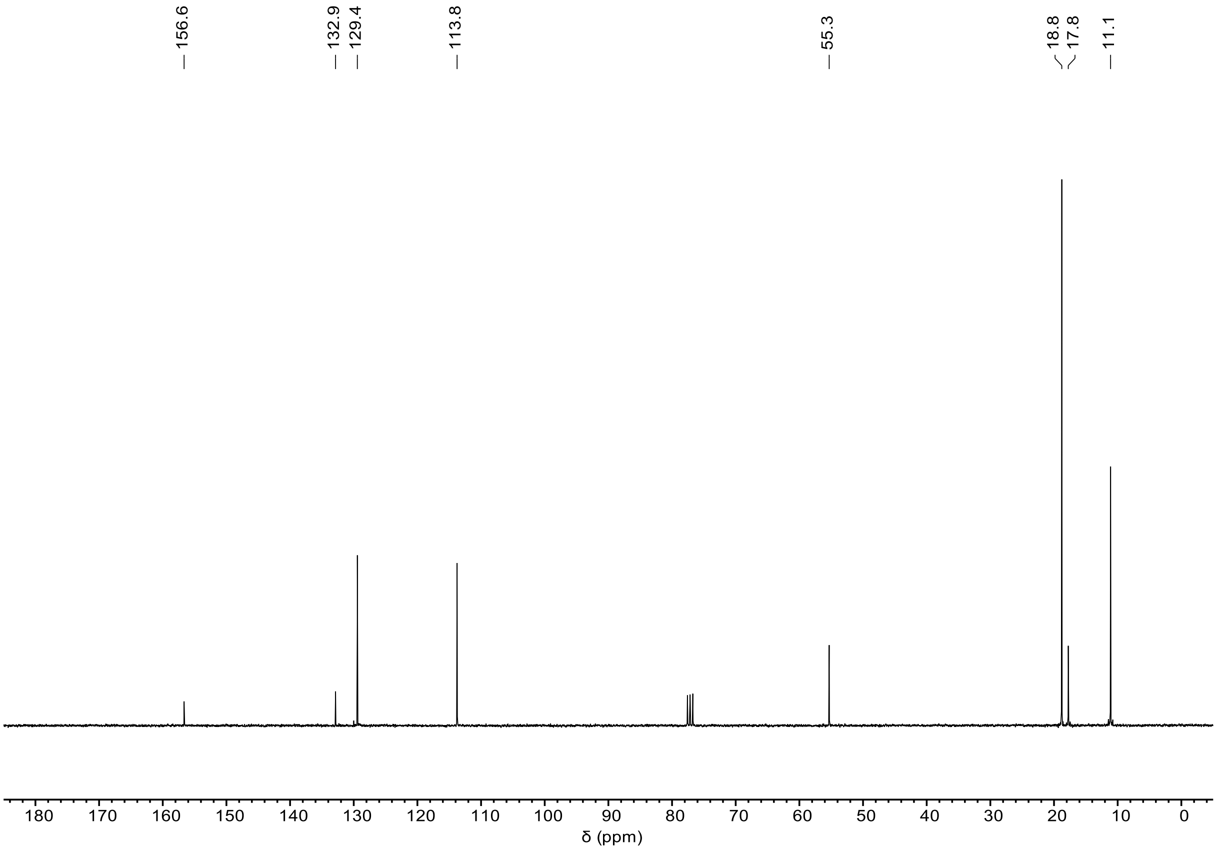

Figure S107. $^{13}C\{^1H\}$ NMR of **4n’** in $CDCl_3$.

Combined Hydrogen Isotope Exchange and Silylation Procedure

(Dibenzo[*b*,*d*]furan-4,6-diyl-*d*$_6$)bis(triisopropylsilane) (4g-*d*$_6$)

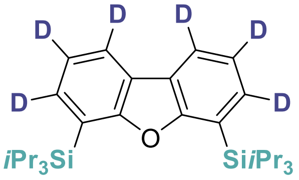

$C_{30}H_{48}OSi_2$
Molecular Weight: 480.88

In an oven-dried and argon-flushed Schlenk flask containing a Teflon-coated magnetic stirrer, NaTMP (196 mg, 1.2 mmol, 2.4 eq.) was suspended in 2.5 mL of dry C_6D_6. The resulting white suspension was stirred and dibenzo[*b*,*d*]furan (84.1 mg, 0.5 mmol, 1 eq.) was added, followed by PMDETA (210 µL, 0.1 mmol, 2 eq.). The mixture was then stirred at room temperature for 12 h. Afterwards, *i*Pr_3SiCl (257 µL, 1.2 mmol, 2.4 eq.) was added and the mixture was stirred for 12 h more. The reaction was quenched with a saturated solution of NH_4Cl (10 mL) and extracted with Et_2O (3 x 10 mL). The organic phase was dried over Na_2SO_4, filtered and dried under reduced pressure. NMR yield was measured by adding 1,1,2,2-tetrachloroethane (26.4 µL, 0.25 mmol) and comparing the corresponding signal in the ^{1}H NMR spectrum. Purification was performed by flash column chromatography in silica gel and hexane. Compound **4g**-*d*$_6$ was isolated as a white solid (222 mg, 91 % yield).

^{1}H NMR (300 MHz, $CDCl_3$): δ 8.07 – 8.02 (m, 1.38H, 31%D), 7.65 – 7.61, (m, 0.16H, 92%D), 7.43 – 7.38 (m, 0.16H, 92%D), 1.85 (sept, J = 7.6 Hz, 6H), 1.24 (d, J = 7.6 Hz, 36H).

^{13}C{^{1}H} NMR (75 MHz, $CDCl_3$): δ 161.2, 134.6, 123.5, 122.1, 121.2, 118.6, 19.1, 12.3.

HRMS (EI) m/z: $[M+6]^+$ Calculated for $C_{30}H_{42}{}^2H_6OSi_2$ 486.3615. Found 486.3598. Isotopic distribution (relative): M+3 (22.30), M+4 (100), M+5 (71.68), M+6 (21.38), M+7 (13.41). Overall deuteration: 70.5 %.

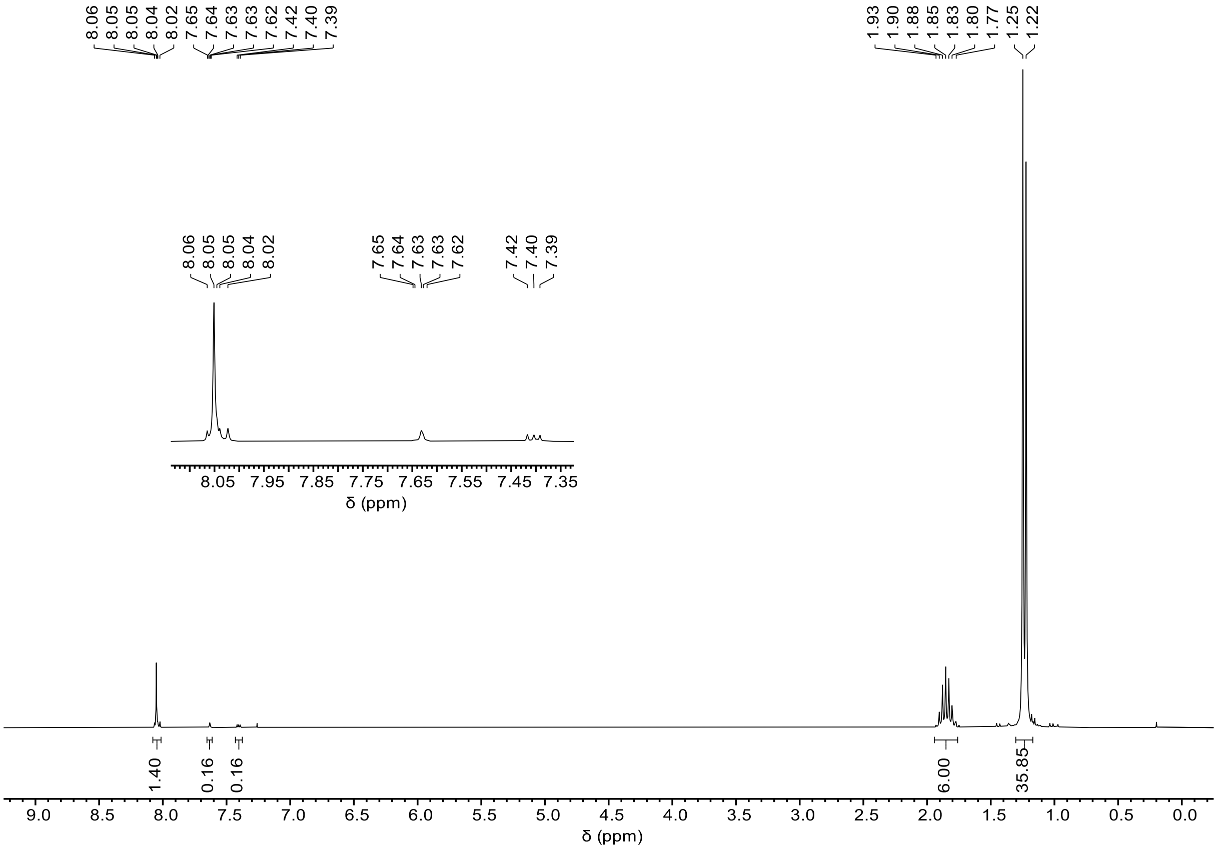

Figure S108. ^{1}H NMR of **4g**-*d*$_6$ in $CDCl_3$.

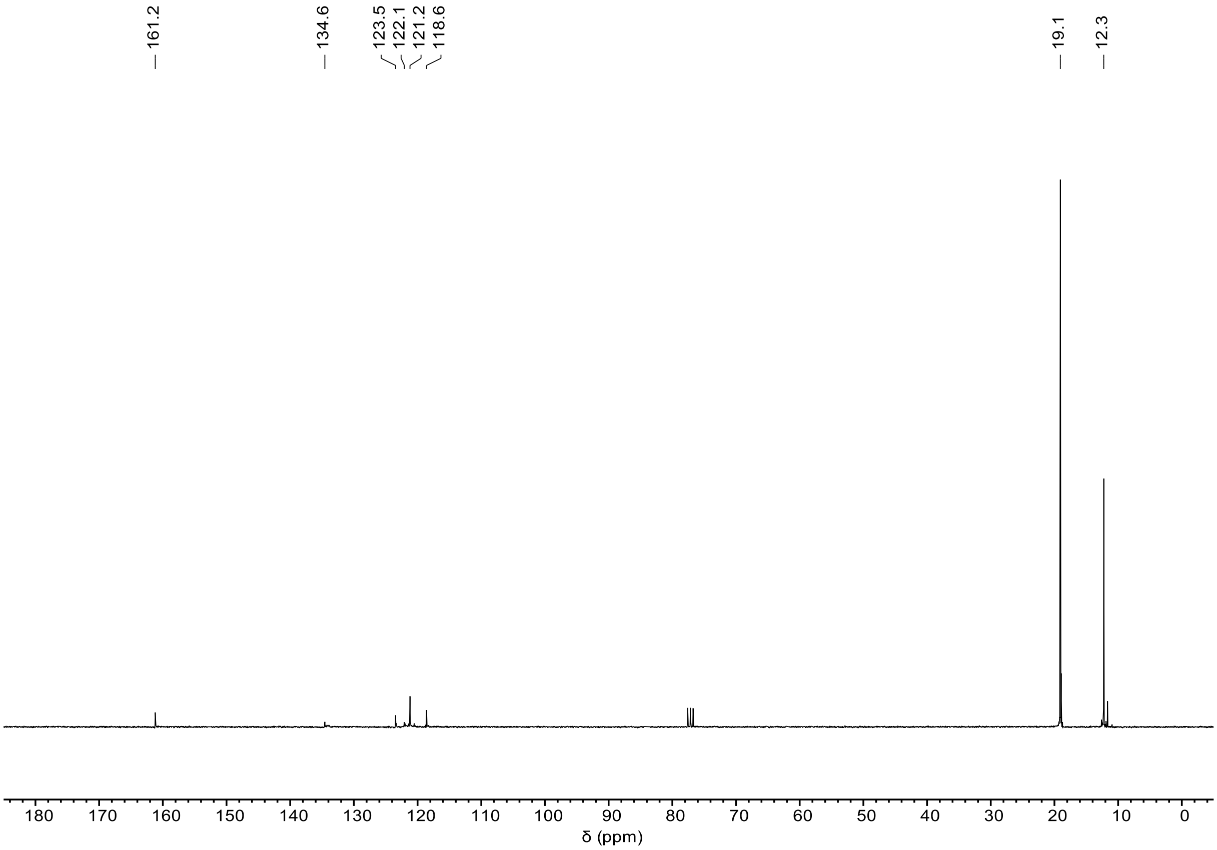

Figure S109. $^{13}C\{^{1}H\}$ NMR of **4g**-*d*$_6$ in $CDCl_3$.

Combined Alkene Isomerization and Silylation Procedure

Cinnamyltriisopropylsilane (5a)

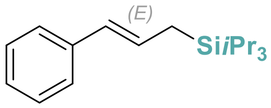

$C_{18}H_{30}Si$
Molecular Weight: 274.52

In an oven-dried and argon-flushed Schlenk flask containing a Teflon-coated magnetic stirrer, NaTMP (97.9 mg, 0.6 mmol, 1.2 eq.) was suspended in 3 mL of dry hexane. The resulting white suspension was stirred and allylbenzene (66.2 µL, 0.5 mmol, 1 eq.) was added, followed by PMDETA (10.4 µL, 0.05 mmol, 0.1 eq.). The mixture was then stirred at room temperature for 4 h. Afterwards, *i*Pr_3SiCl (97 µL, 0.5 mmol, 1 eq.) was added and the mixture was stirred for 6 h more. The reaction was quenched with a saturated solution of NH_4Cl (10 mL) and extracted with Et_2O (3 x 10 mL). The organic phase was dried over Na_2SO_4, filtered and dried under reduced pressure. NMR yield was measured by adding 1,1,2,2-tetrachloroethane (26.4 µL, 0.25 mmol) and comparing the corresponding signal in the ^1H NMR spectrum. Purification was performed by flash column chromatography in silica gel and hexane. Compound **5a** was isolated as a colorless oil (112 mg, 82 % yield). Spectroscopic data are in accordance with literature reports.[22]

^{1}H NMR (300 MHz, $CDCl_3$): *δ* 7.31 – 7.24 (m, 4H), 7.18 – 7.10 (m, 1H), 6.33 – 6.25 (m, 2H), 1.82 – 1.77 (m, 2H), 1.12 – 1.06 (m, 21H).

^{13}C{^{1}H} NMR (75 MHz, $CDCl_3$): *δ* 138.7, 128.8, 128.6, 128.4, 126.3, 125.6, 18.9, 16.8, 11.2.

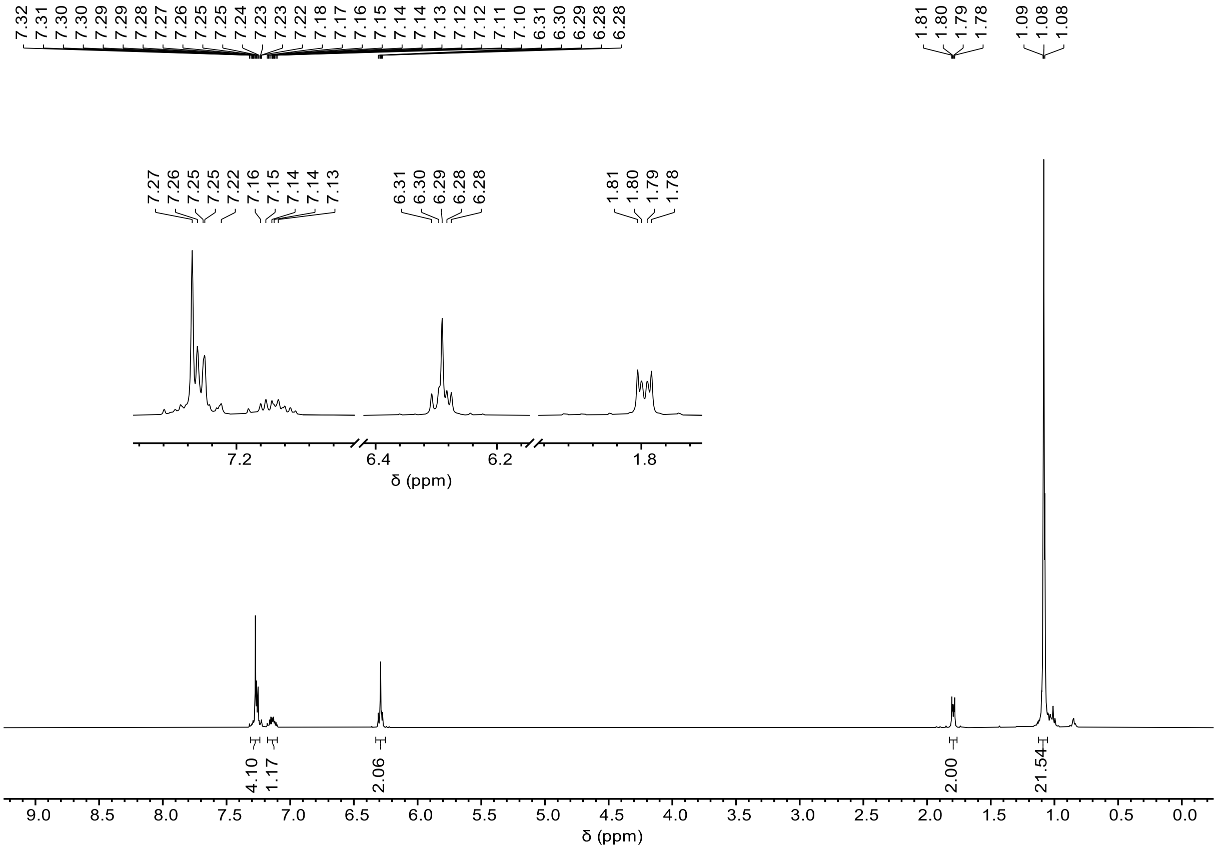

Figure S110. ^{1}H NMR of **5a** in $CDCl_3$.

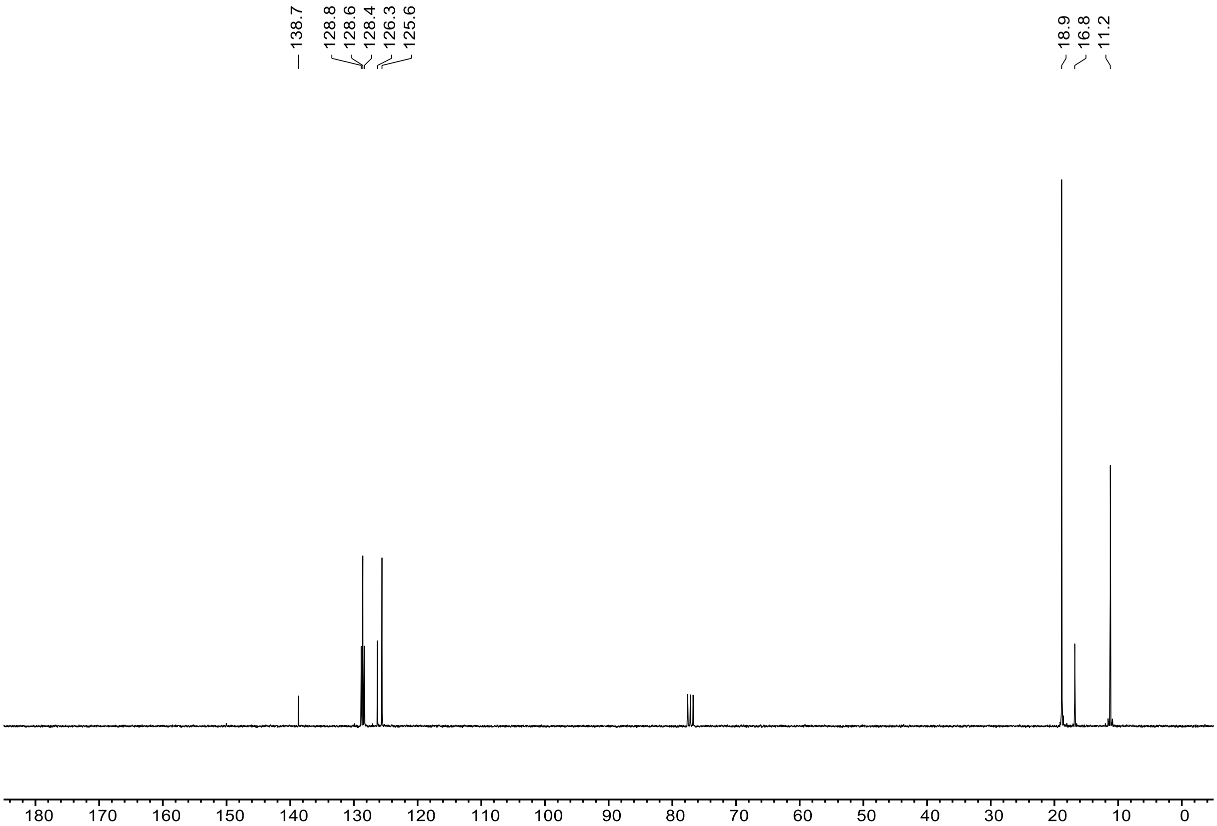

Figure S111. $^{13}C\{^{1}H\}$ NMR of **5a** in $CDCl_3$.

Synthesis of Silyl-Bridged Derivative

Bis(benzo[*b*]thiophen-2-yl)diisopropylsilane (5b)

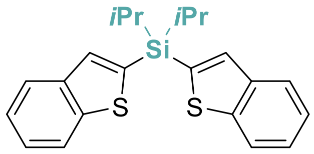

$C_{22}H_{24}S_2Si$
Molecular Weight: 380.64

In an oven-dried and argon-flushed Schlenk flask containing a Teflon-coated magnetic stirrer, NaTMP (81.6 mg, 0.5 mmol, 2 eq.) was suspended in 3 mL of dry hexane. The resulting white suspension was stirred and benzothiophene (67.1 mg, 0.5 mmol, 2 eq.) was added, followed by $i$$Pr_2SiCl_2$ (45.1 µL, 0.25 mmol, 1 eq.) and PMDETA (104 µL, 0.5 mmol, 2 eq.). The mixture was then stirred at room temperature for 16 h. Afterwards, the reaction was quenched with a saturated solution of NH_4Cl (10 mL) and extracted with Et_2O (3 x 10 mL). The organic phase was dried over Na_2SO_4, filtered and dried under reduced pressure. NMR yield was measured by adding 1,1,2,2-tetrachloroethane (26.4 µL, 0.25 mmol) and comparing the corresponding signal in the ^{1}H NMR spectrum. Purification was performed by filtration through silica and dichloromethane. Compound **5b** was isolated as a white solid (88 mg, 92 % yield).

^{1}H NMR (300 MHz, $CDCl_3$): δ 8.01 – 7.88 (m, 4H), 7.75 (d, J = 0.7 Hz, 2H), 7.47 – 7.36 (m, 4H), 1.67 (sept, J = 7.4 Hz, 2H), 1.22 (d, J = 7.4 Hz, 12H).

^{13}C{^{1}H} NMR (75 MHz, $CDCl_3$): δ 144.1, 140.9, 134.6, 134.1, 124.7, 124.2, 123.8, 122.2, 17.9, 12.3.

HRMS (ESI) m/z: $[M+Na]^+$ Calculated for $C_{22}H_{24}NaS_2Si$ 403.0981. Found 403.0983.

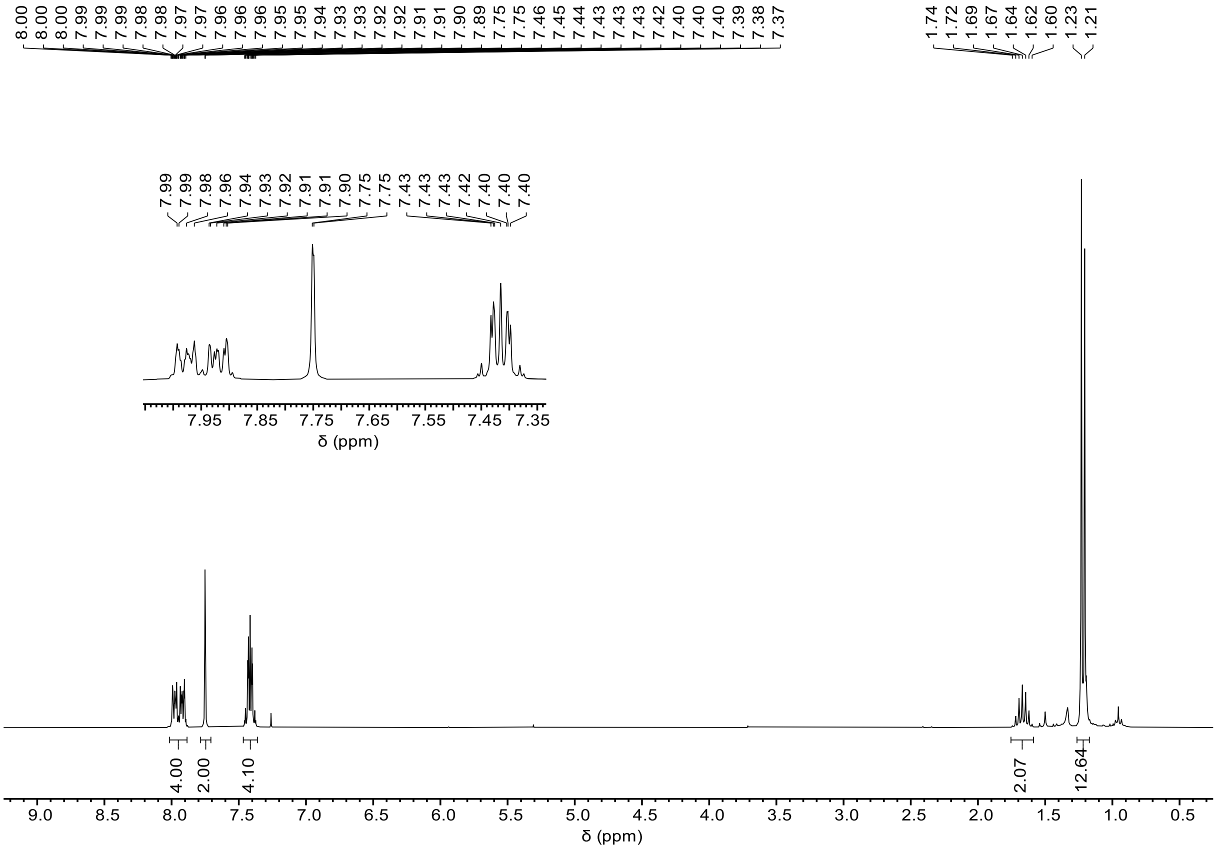

Figure S112. ^{1}H NMR of **5b** in $CDCl_3$.

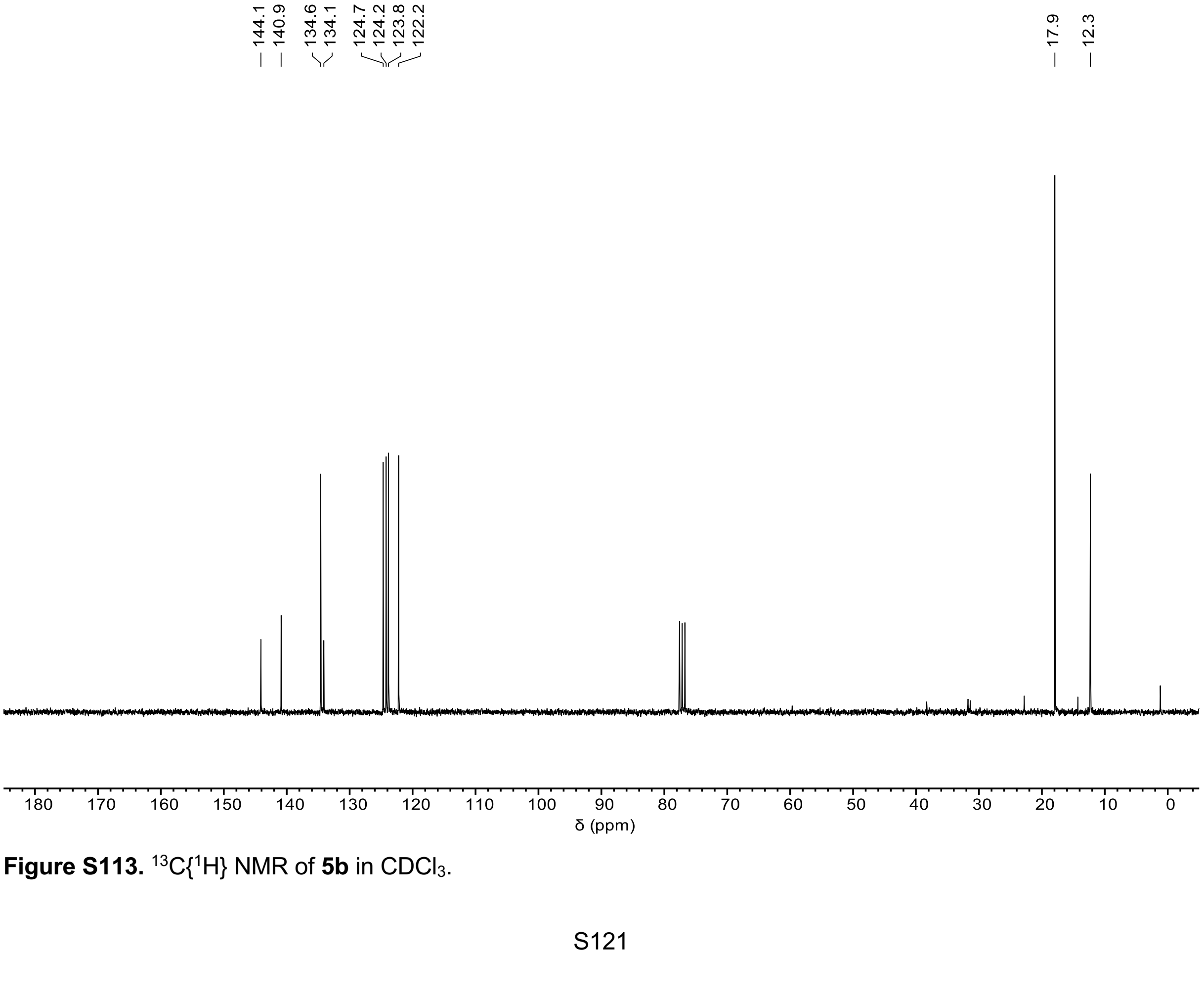

Figure S113. $^{13}C\{^{1}H\}$ NMR of **5b** in $CDCl_3$.

C–C Coupling of Organosilicon Reagent

4-(4-Benzylpyridin-2-yl)morpholine (5c)

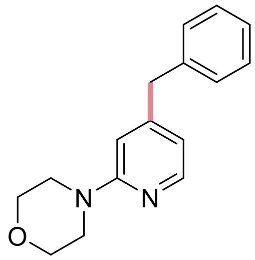

$C_{16}H_{18}N_2O$
Molecular Weight: 254.33

In an oven-dried and argon-flushed Schlenk flask containing a Teflon-coated magnetic stirrer, 2-morpholinoisonicotinonitrile (94.6 mg, 0.5 mmol, 1 eq.), benzyltriethylsilane **1a** (123.8 mg, 0.6 mmol, 1.2 eq.) and 18-crown-6 (1 M in dry THF, 145.4 mg, 0.55 mmol, 1.1 eq.) were charged and dissolved in 1.5 mL of dry DMSO. The resulting colorless suspension was stirred and CsF (76 mg, 0.5 mmol, 1 eq.) was added. The mixture was then stirred at room temperature for 16 h. Afterwards, the reaction was quenched with distilled water (10 mL) and extracted with Et_2O (3 x 10 mL). The organic phase was dried over Na_2SO_4, filtered and dried under reduced pressure. NMR yield was measured by adding 1,1,2,2-tetrachloroethane (26.4 µL, 0.25 mmol) and comparing the corresponding signal in the ^1H NMR spectrum. Purification was performed by flash column chromatography in silica gel and hexane/EtOAc (65:35), compound **5c** was isolated as an off-white solid (90 mg, 71 % yield).

^{1}H NMR (300 MHz, $CDCl_3$): δ 8.01 (dd, J = 5.1, 0.6 Hz, 1H), 7.25 – 7.17 (m, 2H), 7.17 – 7.12 (m, 1H), 7.12 – 7.06 (m, 2H), 6.42 (dd, J = 5.1, 1.4 Hz, 1H), 6.37 (dd, J = 1.4, 0.6 Hz, 1H), 3.74 – 3.68 (m, 4H), 3.40 – 3.34 (m, 4H).

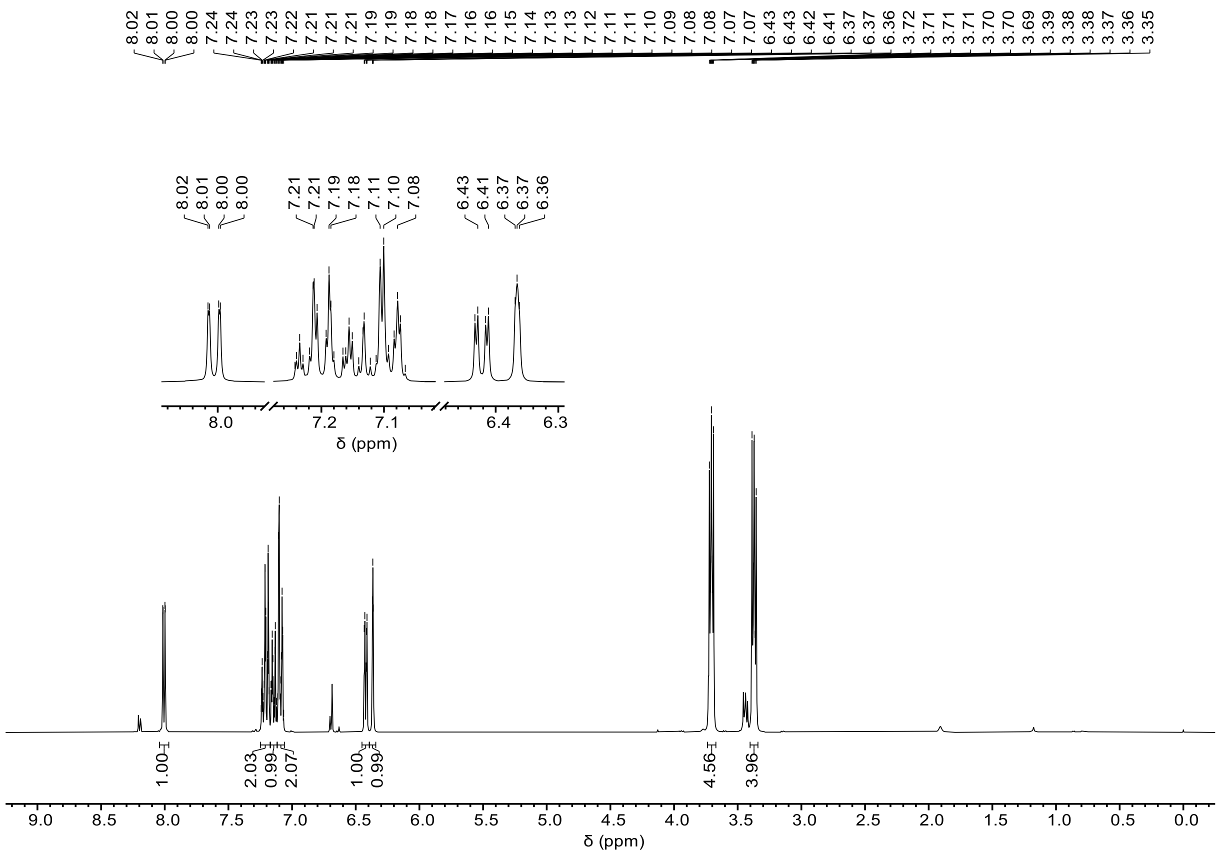

Figure S114. ^1H NMR of **5c** in $CDCl_3$.

Decomposition of Fluoroarenes

Decomposition of 4-fluoroanisole and trapping of the benzyne intermediate

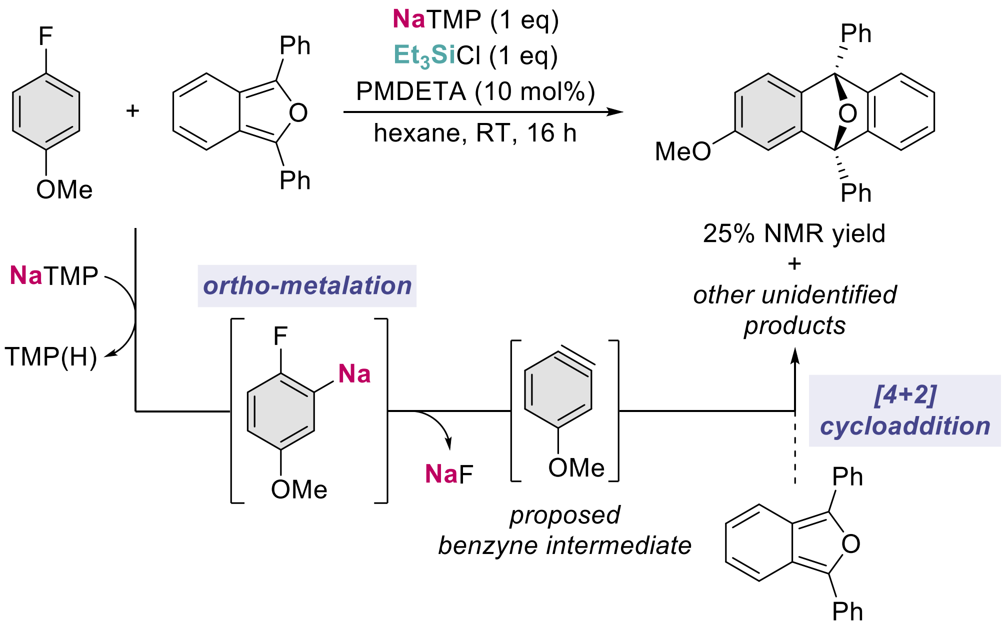

In an oven-dried and argon-flushed Schlenk flask containing a Teflon-coated magnetic stirrer, NaTMP (81.6 mg, 0.5 mmol, 1 eq.) was suspended in 3 mL of dry hexane. The resulting white suspension was stirred and Et_3SiCl (83.9 µL, 0.5 mmol, 1 eq.) was added, followed by 1,3-diphenylisobenzofuran (135.2 mg, 0.5 mmol, 1 eq.), 4-fluoroanisole (56.6 µL, 0.5 mmol, 1 eq.) and PMDETA (10.4 µL, 0.05 mmol, 10 mol%). The mixture was then stirred at room temperature for 16 h. Afterwards, the reaction was quenched with a saturated solution of NH_4Cl (10 mL) and extracted with Et_2O (3 x 10 mL). The organic phase was dried over Na_2SO_4, filtered and dried under reduced pressure. NMR yield was measured by adding 1,1,2,2-tetrachloroethane (26.4 µL, 0.25 mmol) and comparing the corresponding signal in the ^1H NMR spectrum. The ^1H NMR shows a 25% yield for the [4+2]-cycloaddition product[23] (based on the formation of a benzyne intermediate, as shown in the above scheme) along with other unidentified products.

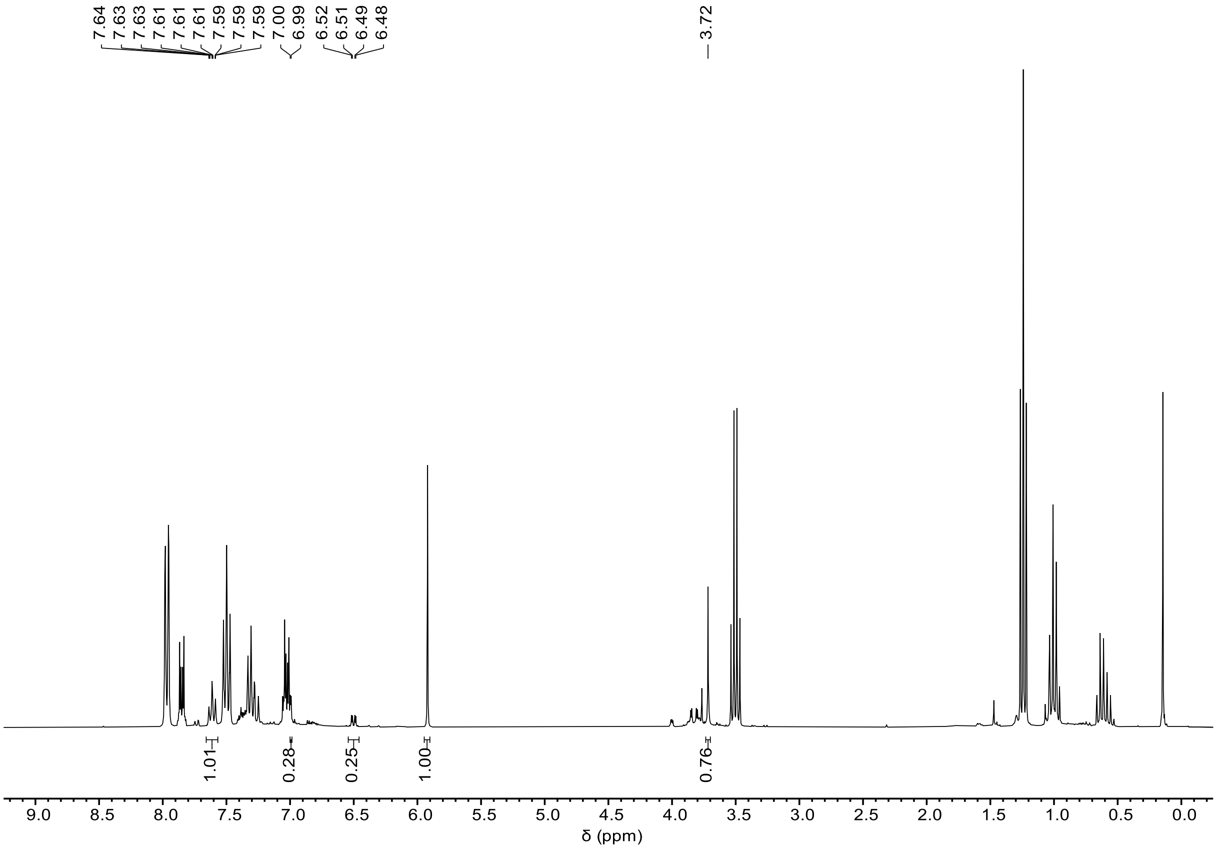

Figure S115. ^{1}H NMR of the reaction crude after work-up and addition of 1,1,2,2-tetrachloroethane. Integrals are given for internal standard (5.92 ppm) and non-overlapping main signals for [4+2]-cycloaddition product.

NMR Monitoring Experiments

C(sp^3)–H silylation of 2-methylnaphthalene

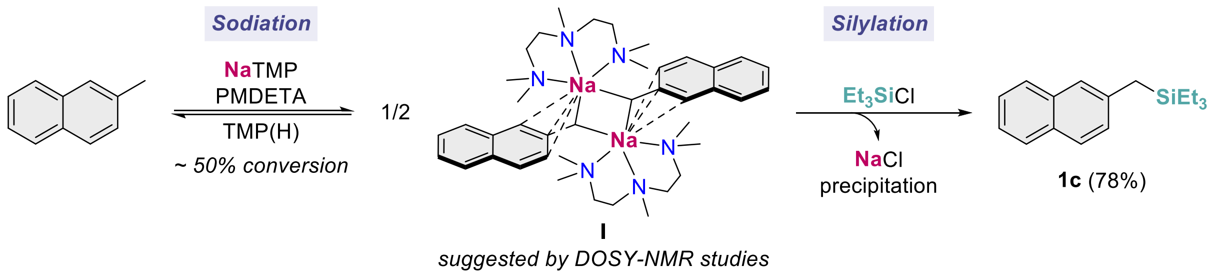

In an oven-dried J-Young NMR tube inside the glovebox, 2-methylnaphthalene (14.2 mg, 0.1 mmol) was dissolved in 0.5 mL of dry C_6D_{12}. To the resulting colorless solution NaTMP (16.3 mg, 0.1 mmol) and PMDETA (20.9 µL, 0.1 mmol) were added, leading to the immediate occurrence of a deep purple solution and the partial precipitation of a solid of the same color, the sodiated species {(PMDETA)Na(2-CH_2Naph)} (**I**). Afterwards, Et_3SiCl (16.8 µL, 0.1 mmol) was added, resulting in the formation of a colorless solution, containing the silylated product triethyl(naphthalen-2-ylmethyl)silane (**1c**) in a 78% yield, and the concomitant precipitation of NaCl as a white solid.

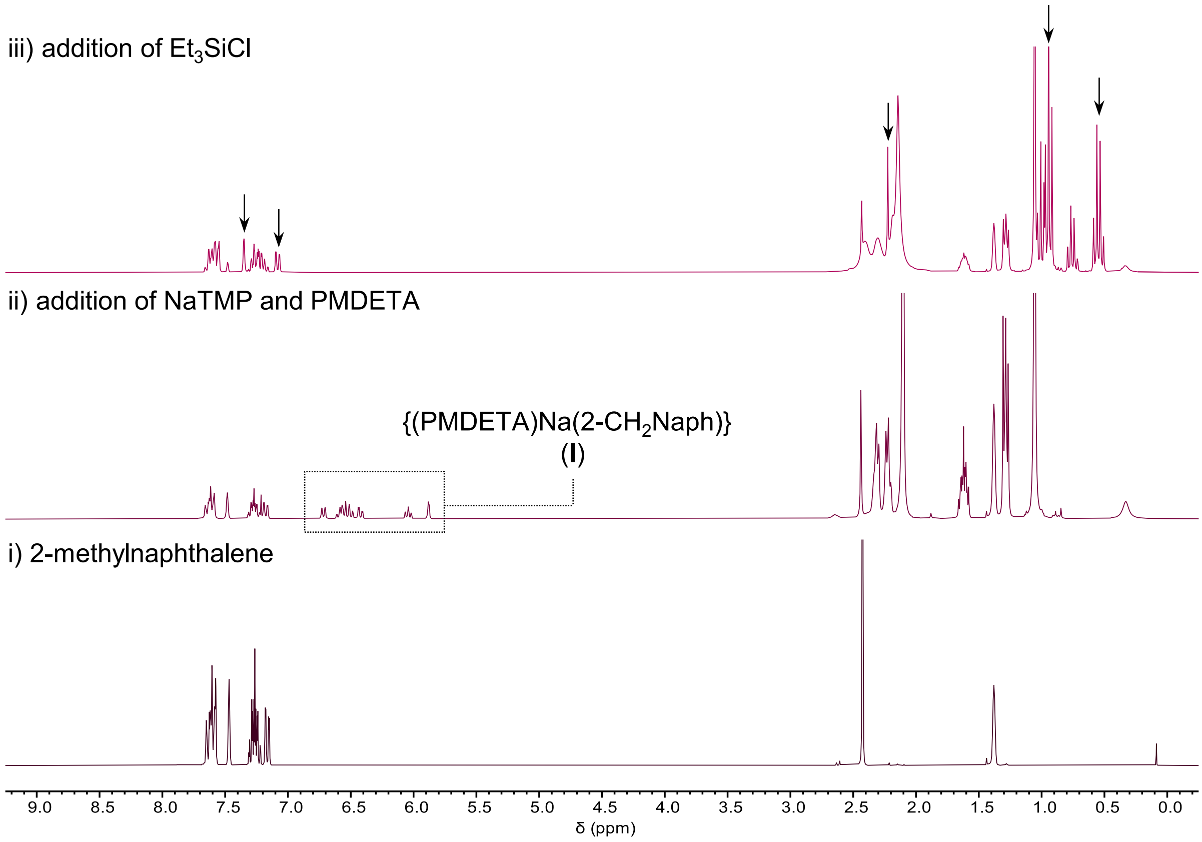

Figure S116. ^{1}H NMR monitoring experiments for the C(sp^3)–H silylation of 2-methylnaphthalene in C_6D_{12}. Arrows indicate the main signals for silylated product triethyl(naphthalen-2-ylmethyl)silane (**1c**).

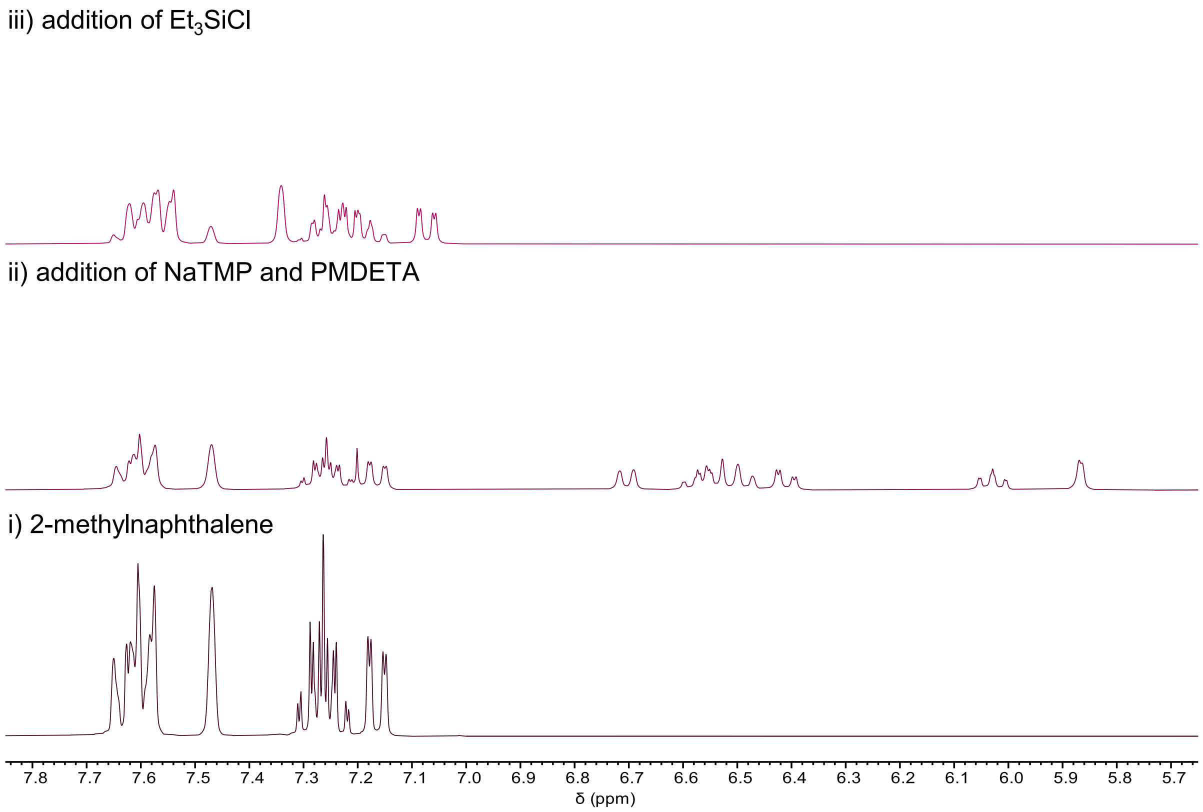

Figure S117. ^{1}H NMR monitoring experiments for the C(sp^3)–H silylation of 2-methylnaphthalene in C_6D_{12}. Amplification of the aromatic region.

C(sp^3)–H silylation of benzyltriethylsilane (1a)

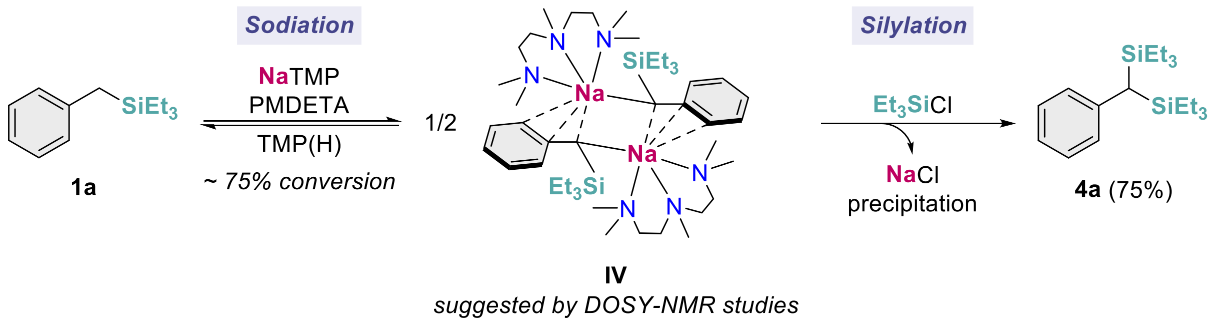

In an oven-dried J-Young NMR tube inside the glovebox, benzyltriethylsilane (**1a**) (20.6 mg, 0.1 mmol) was dissolved in 0.5 mL of dry C_6D_{12}. To the resulting colorless solution NaTMP (16.3 mg, 0.1 mmol) and PMDETA (20.9 µL, 0.1 mmol) were added, leading to the immediate occurrence of a yellow solution. Afterwards, Et_3SiCl (16.8 µL, 0.1 mmol) was added, resulting in the formation of a colorless solution, containing the silylated product (phenylmethylene)bis(triethylsilane) (**4a**) in a 75% yield, and the concomitant precipitation of NaCl as a white solid.

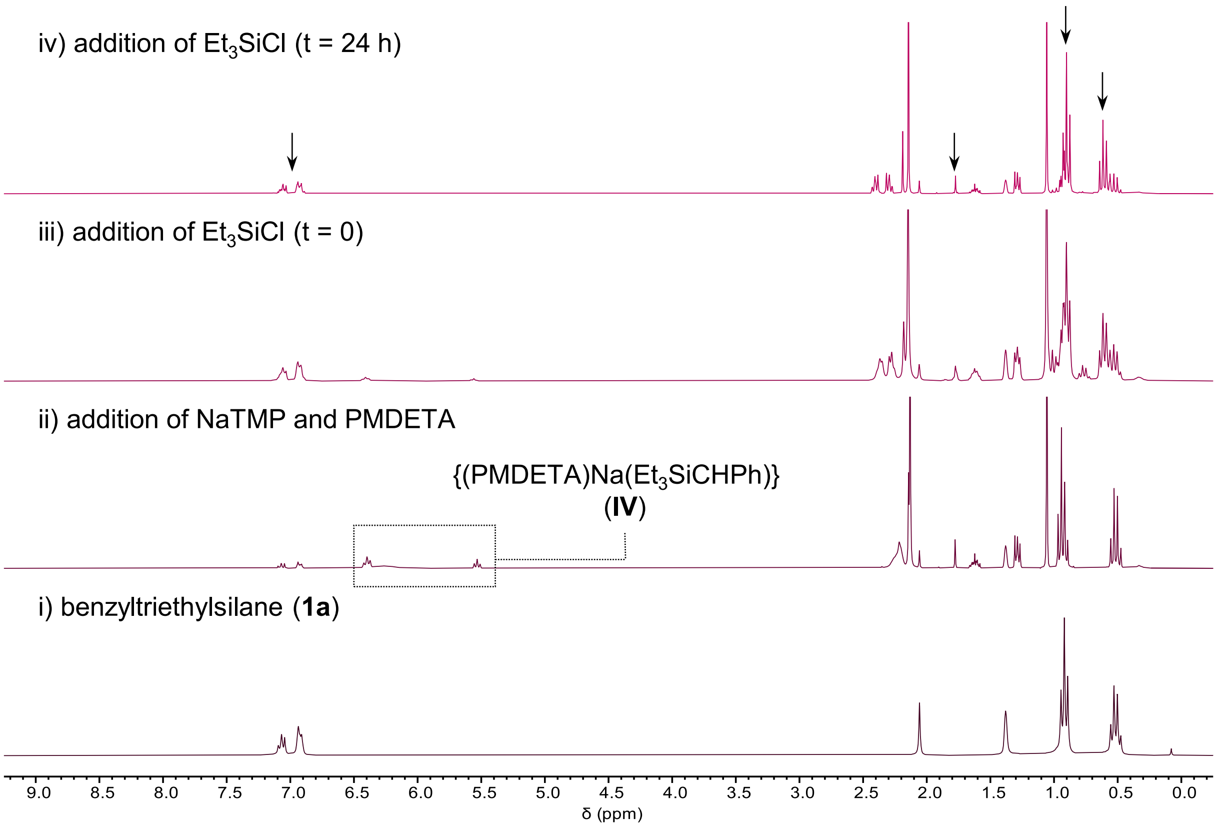

Figure S118. ^{1}H NMR monitoring experiments for the C(sp^3)–H silylation of benzyltriethylsilane (**1a**) in C_6D_{12}. Arrows indicate the main signals for silylated product (phenylmethylene)bis(triethylsilane) (**4a**).

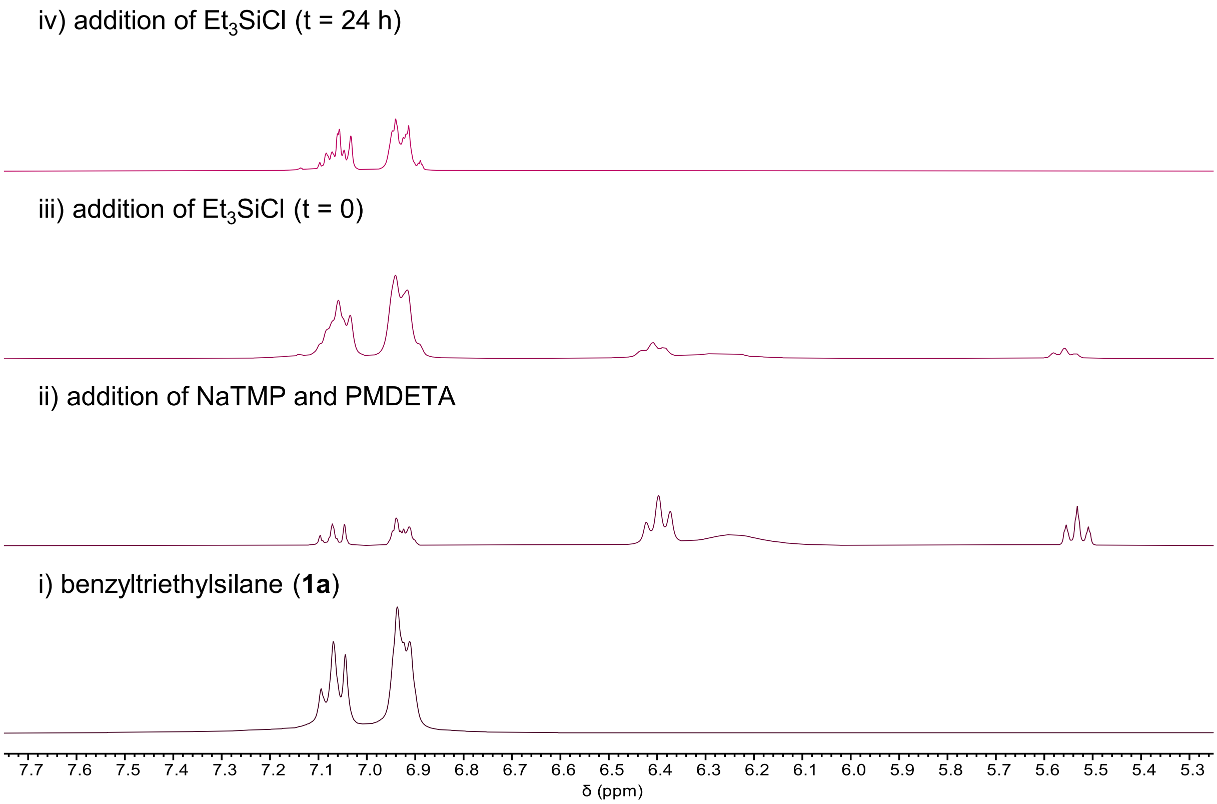

Figure S119. ^1H NMR monitoring experiments for the C(sp^3)–H silylation of benzyltriethylsilane (**1a**). Amplification of the aromatic region.

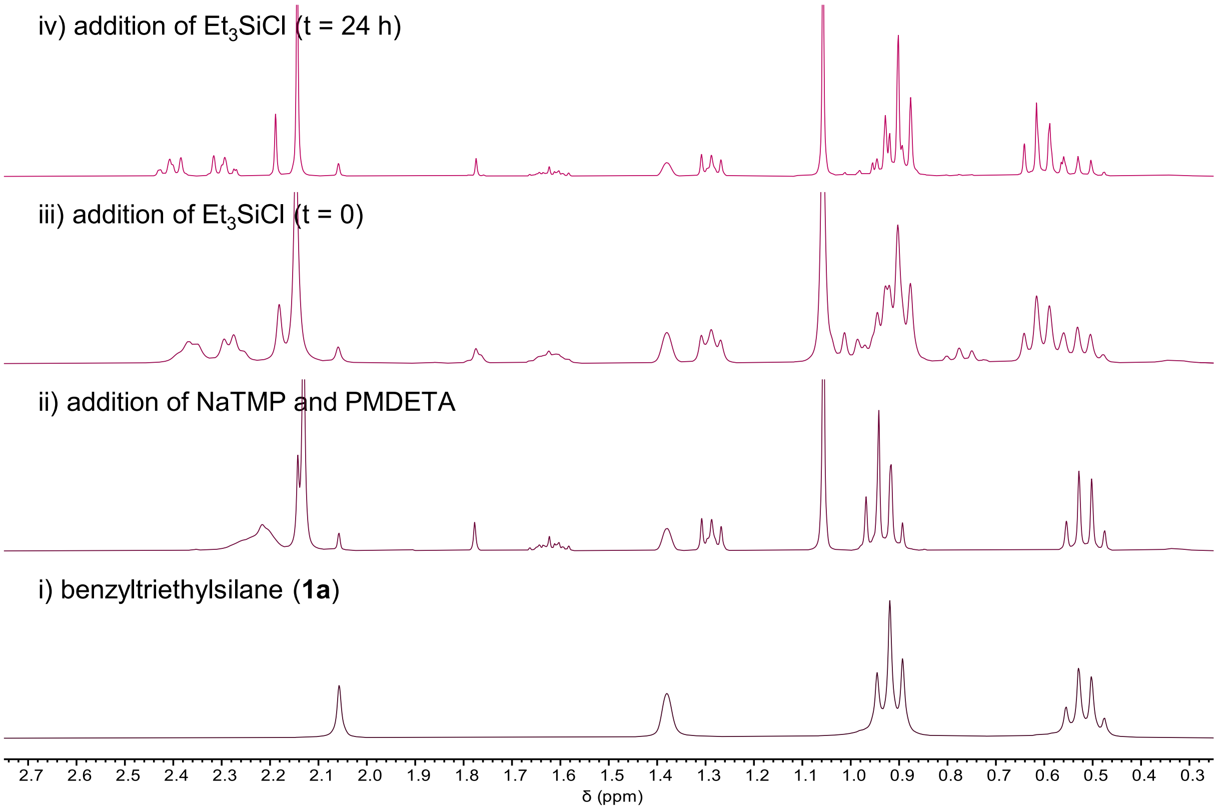

Figure S120. ^1H NMR monitoring experiments for the C(sp^3)–H silylation of benzyltriethylsilane (**1a**). Amplification of the aliphatic region.

Decomposition studies of trialkylchlorosilanes R_3SiCl with NaTMP

In an oven-dried J-Young NMR tube inside the glovebox, trialkylchlorosilane R_3SiCl (0.05 mmol, 1 eq.) and PMDETA (10.4 µL, 0.05 mmol, 1 eq.) were dissolved in C_6D_{12}. The colorless solution was monitored for 16 h confirming before the addition of NaTMP, confirming that both substances remained unreacted in solution. Afterwards, NaTMP (8.2 mg, 0.05 mmol, 1 eq.) was added. The reaction was monitored from t = 0 to 24 h.

· *for Me_3SiCl:* addition of NaTMP leads to immediate changes in the NMR spectrum along with the precipitation of a white solid, presumably NaCl. At t = 0 the only resonance for Me_3SiCl (singlet at 0.36 ppm) splits into several broad unidentified signals ranging from 0.41 to 0.02 ppm. Near-quantitative formation of TMP(H) can be observed (resonances at 1.62, 1.29, 1.06 ppm). No further changes were observed at longer reaction times. The mixture was analyzed by ^{29}Si–^{1}H HMBC and mass spectrometry.

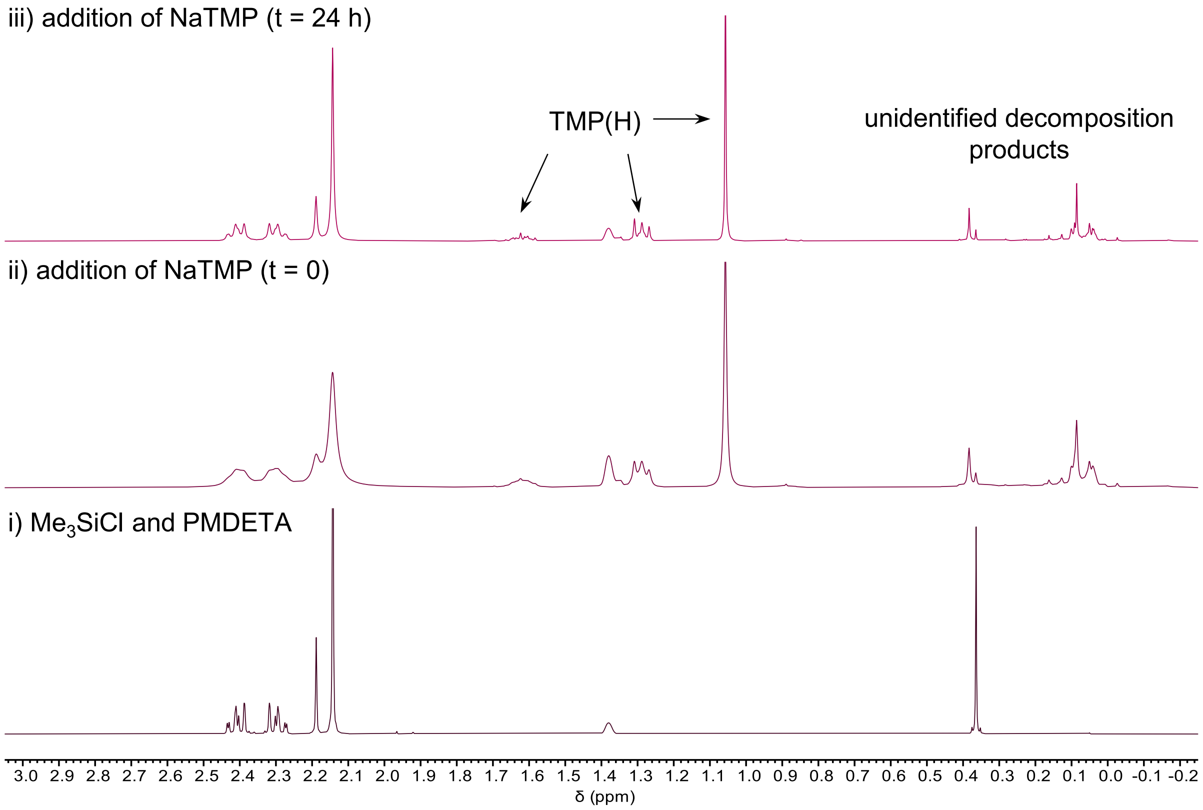

Figure S121. ^{1}H NMR monitoring experiments for the decomposition studies of Me_3SiCl with NaTMP and PMDETA in C_6D_{12}.

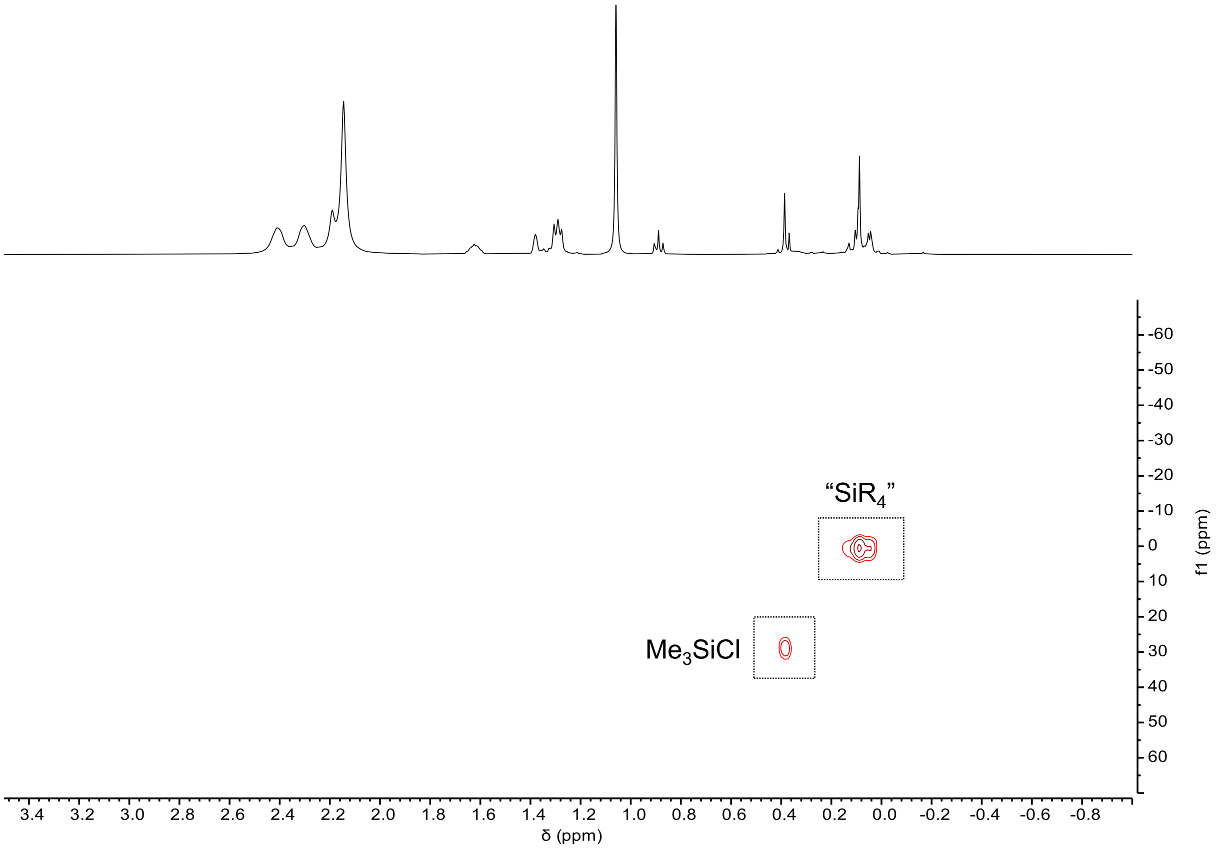

Figure S122. ^{29}Si-^{1}H HMBC experiment for the decomposition study of Me_3SiCl with NaTMP and PMDETA in C_6D_{12}. "SiR_4" refers to a Si species bonded to four alkyl groups (see MS analysis for a plausible species).

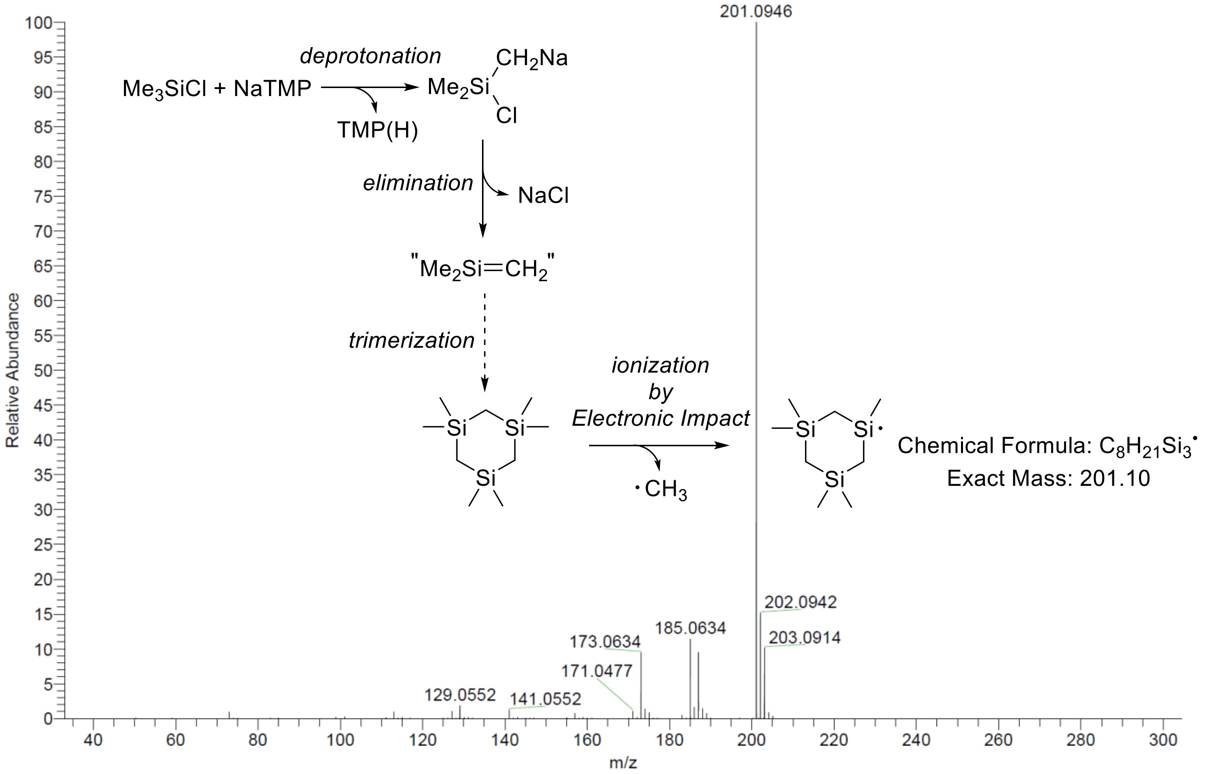

Figure S123. Mass spectrum of the crude reaction mixture and plausible mechanism for the decomposition of Me_3SiCl with NaTMP.

· *for Et_3SiCl:* addition of NaTMP leads to immediate changes in the NMR spectrum although to a lesser extent compared to the reaction with Me_3SiCl. Partial precipitation of a white solid, presumably NaCl, was also observed. At t = 0 the resonances for Et_3SiCl (triplet at 1.01 ppm and quartet at 0.76 ppm) broaden and minor unidentified signals emerge ranging from 1.19 to 0.45 ppm. Concomitant formation of TMP(H) can be observed (resonances at 1.62, 1.29, 1.06 ppm) in an approximate 40%, which slightly increases up to around 50% within 2 h. No further changes were observed at longer reaction times.

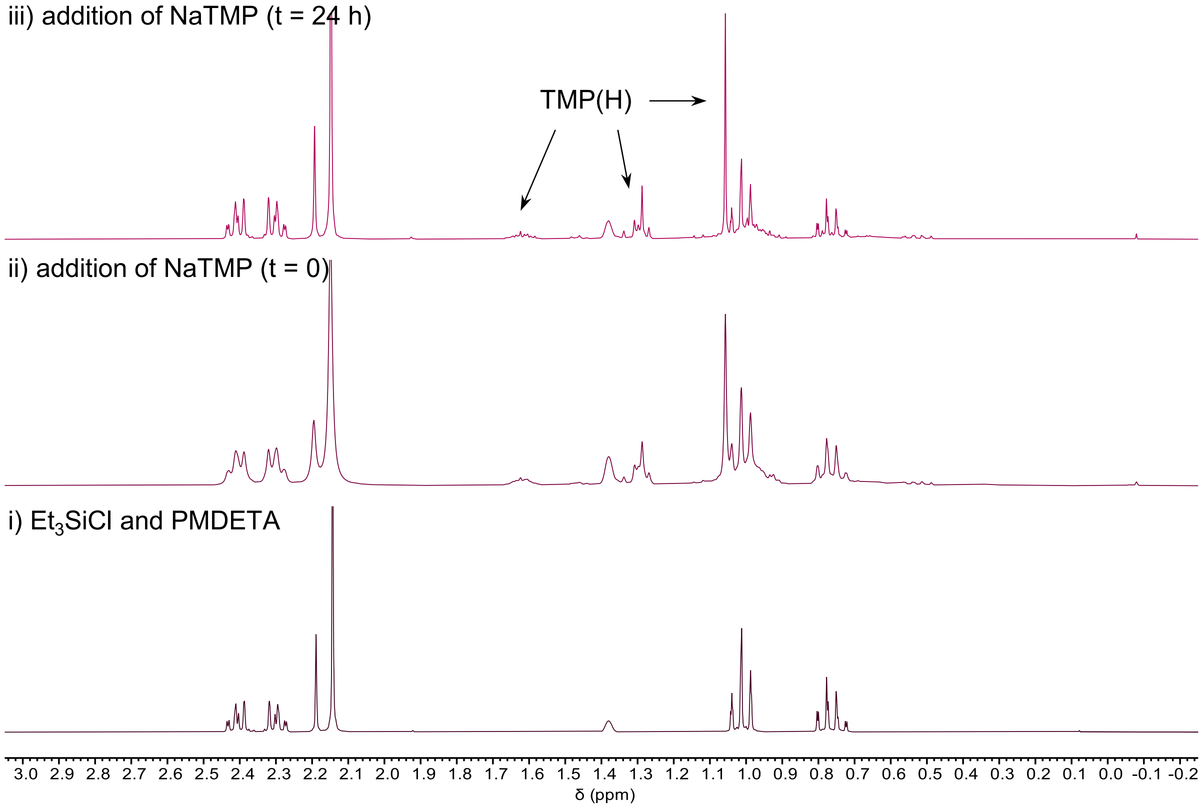

Figure S124. ^{1}H NMR monitoring experiments for the decomposition studies of Et_3SiCl with NaTMP and PMDETA in C_6D_{12}.

· *for iPr₃SiCl:* addition of NaTMP does not cause immediate changes in the NMR spectrum. No precipitation of any solids was observed. At t = 0 the resonances for *i*Pr_3SiCl (multiplet at 1.18 ppm and pseudo-doublet at 1.10 ppm) remain unmodified. Broad signals for NaTMP can be observed (resonances at 1.69, 1.23, 1.03 ppm). TMP(H) resonances can be observed at a very minor extent (<10%) increasing within 24 h (approximately 20%), although signal overlapping complicates accurate integration. Note that weak resonances appear around 2.56 ppm during the monitoring, which could be ascribed to minor PMDETA metalation by NaTMP with concomitant formation of TMP(H).

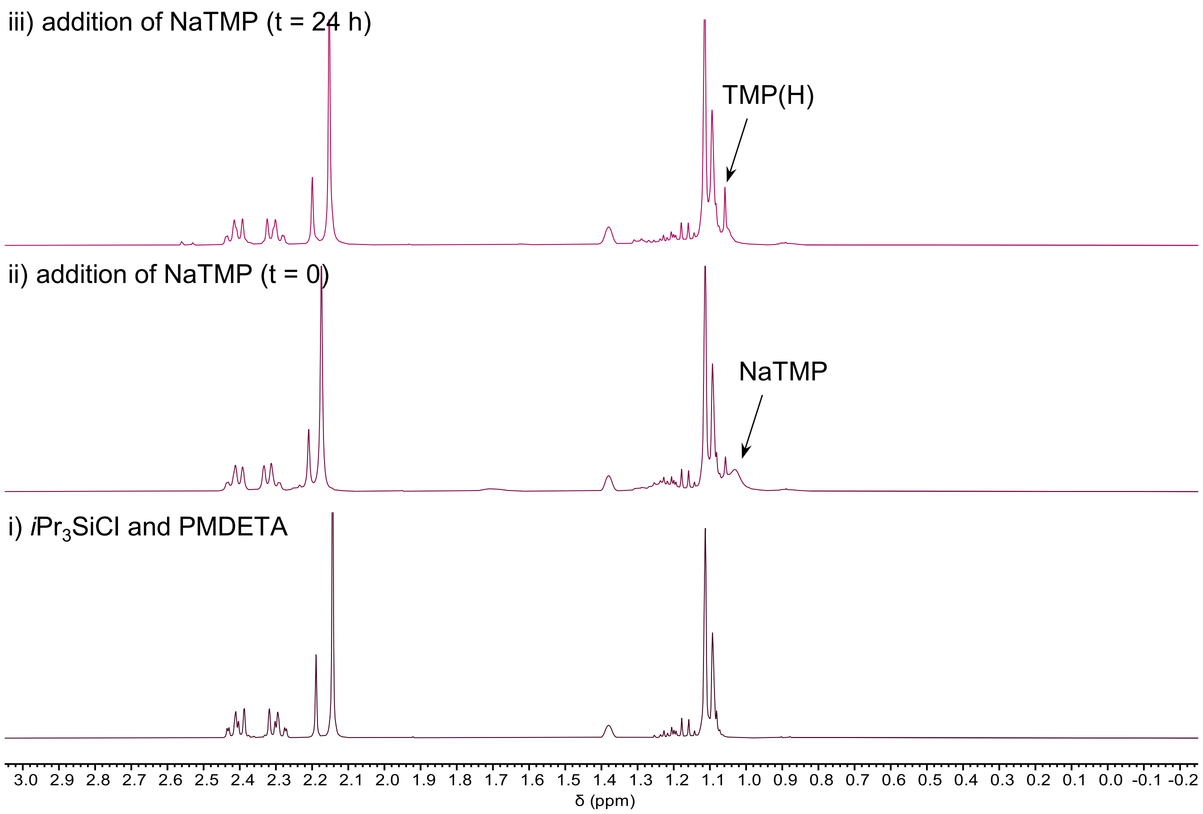

Figure S125. ^{1}H NMR monitoring experiments for the decomposition studies of *i*Pr_3SiCl with NaTMP and PMDETA in C_6D_{12}.

^{1}H DOSY NMR Experiments

{(PMDETA)Na(2-CH$_2$Naph)} (I)

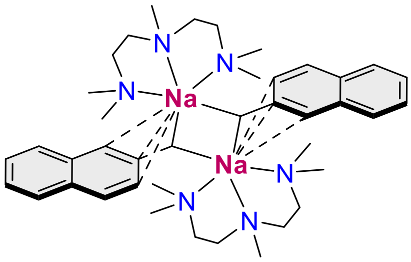

$C_{40}H_{64}N_6Na_2$
Molecular Weight: 674.97

In an oven-dried J-Young NMR tube inside the glovebox, 2-methylnaphthalene (3.6 mg, 0.025 mmol) was dissolved in 0.5 mL of dry C_6D_{12}. To the resulting colorless solution NaTMP (4.1 mg, 0.025 mmol) and PMDETA (5.2 µL, 0.025 mmol) were added, leading to the immediate occurrence of a deep purple solution. Analysis of the reaction crude by ^{1}H DOSY using the residual protic signal of the solvent as reference suggested an estimated molecular weight (DSE) for **I** of 727 g·mol^{-1}, which is in agreement with the theoretical molecular weight of 675 g·mol^{-1} (−7%) for the corresponding dimeric structure {(PMDETA)Na(2-CH$_2$Naph)}$_2$ in C_6D_{12}.

D(average) = 3.034e^{-10} m^2·s^{-1}

D(C_6D_{12}) = 10.77e^{-10} m^2·s^{-1}

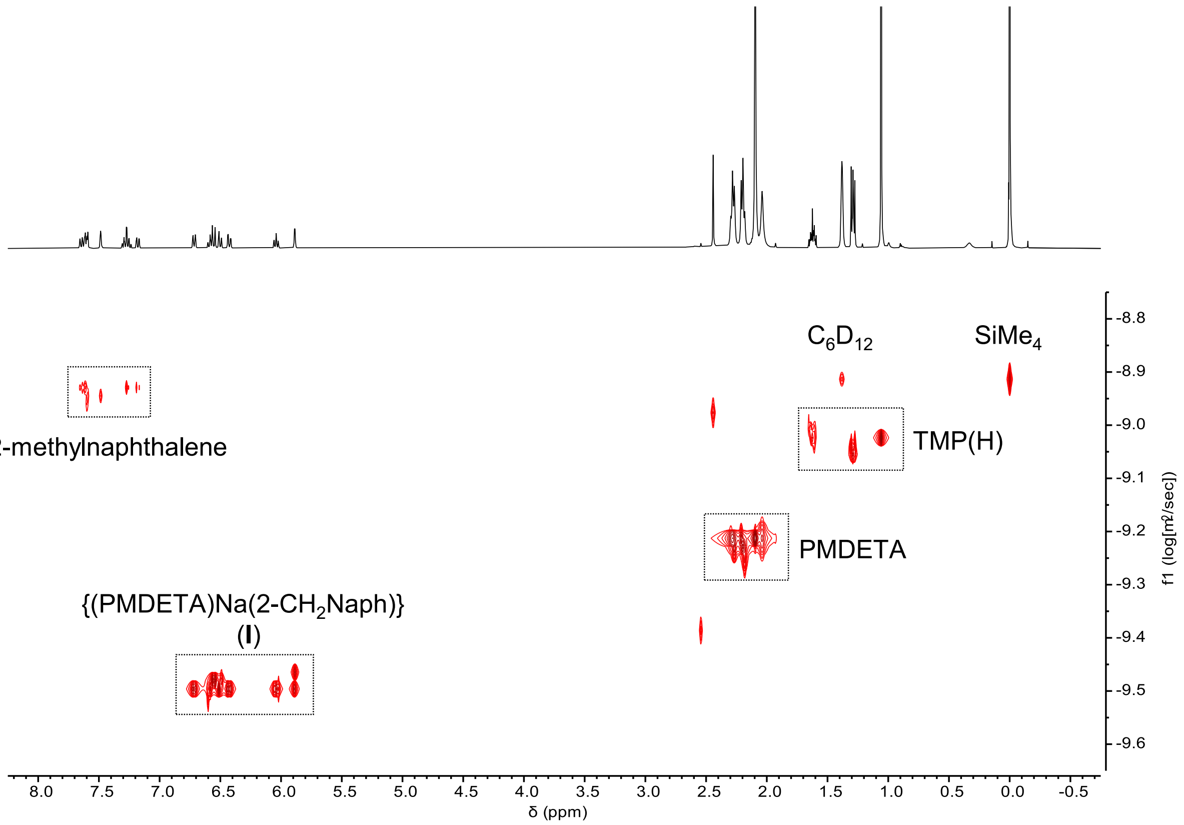

Figure S126. ^{1}H DOSY NMR spectrum of complex **I** in C_6D_{12}.

{(PMDETA)Na(Et$_3$SiCHPh)} (IV)

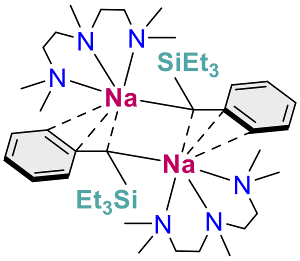

$C_{44}H_{88}N_6Na_2Si_2$
Molecular Weight: 803.38

In an oven-dried J-Young NMR tube inside the glovebox, benzyltriethylsilane (**1a**) (5.2 mg, 0.025 mmol) was dissolved in 0.5 mL of dry C_6D_{12}. To the resulting colorless solution NaTMP (4.1 mg, 0.025 mmol) and PMDETA (5.2 µL, 0.025 mmol) were added, leading to the immediate occurrence of a yellow solution. Analysis of the reaction crude by ^{1}H DOSY using the signal of $SiMe_4$ as reference suggested an estimated molecular weight (DSE) for **IV** of 720 g·mol^{-1}, which is in agreement with the theoretical molecular weight of 803 g·mol^{-1} (+12%) for the corresponding dimeric structure {(PMDETA)Na(Et$_3$SiCHPh)}$_2$ in C_6D_{12}.

D(average) = 3.234e^{-10} m^2·s^{-1}

D($SiMe_4$) = 11.53e^{-10} m^2·s^{-1}

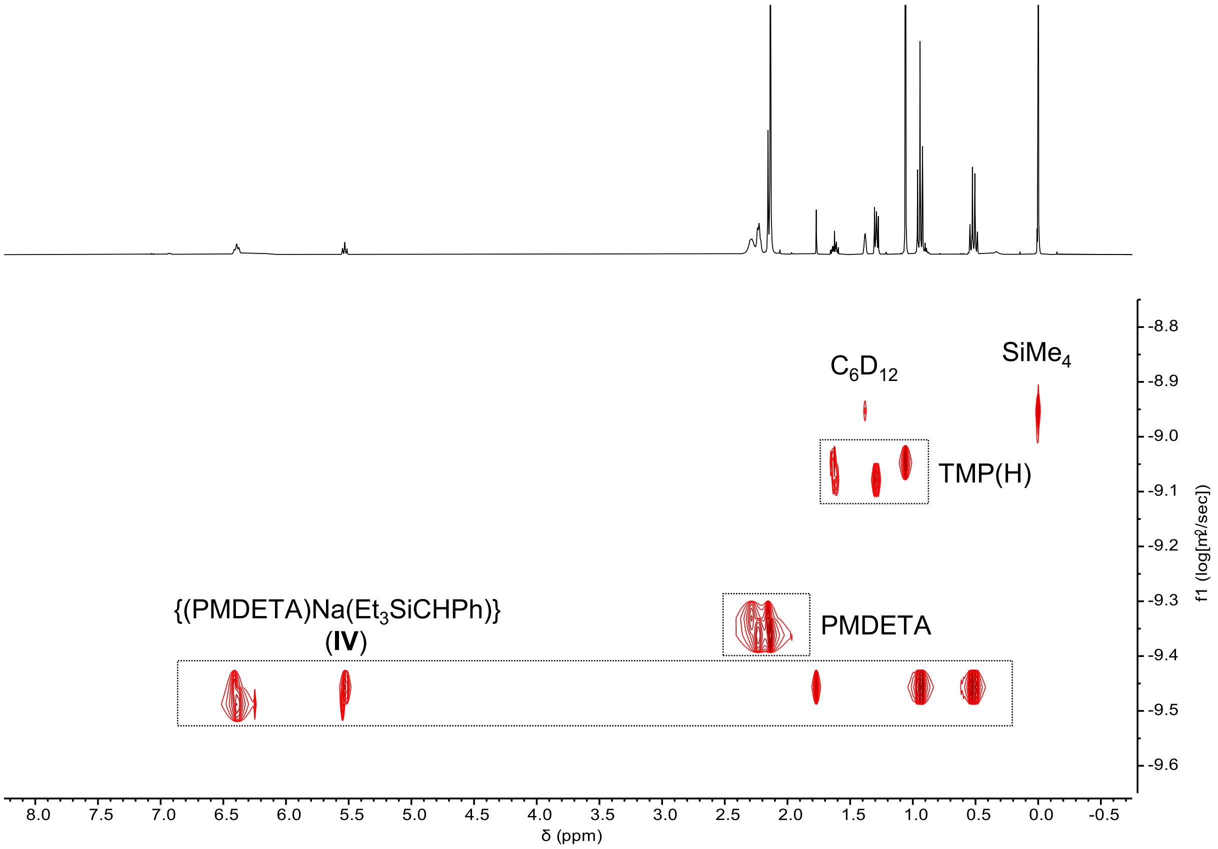

Figure S127. ^{1}H DOSY NMR spectrum of complex **IV** in C_6D_{12}.

Metalation of toluene with NaTMP

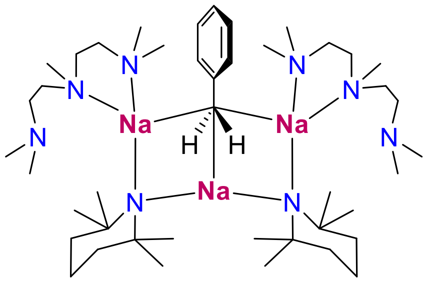

$C_{43}H_{89}N_8Na_3$
Molecular Weight: 787.21

In an oven-dried J-Young NMR tube inside the glovebox, toluene (2.7 µL, 0.025 mmol) was dissolved in 0.5 mL of dry C_6D_{12}. To the resulting colorless solution NaTMP (4.1 mg, 0.025 mmol) and PMDETA (5.2 µL, 0.025 mmol) were added, leading to the immediate occurrence of a yellow solution. Analysis of the reaction crude by ^{1}H DOSY using the residual protic signal of the solvent as reference suggested an estimated molecular weight (DSE) for $NaCH_2Ph$ of 768 g·mol^{-1}, which is in agreement with the theoretical molecular weight of 787 g·mol^{-1} (+2%) for the corresponding heteroleptic trimeric structure $\{(PMDETA)_2Na_3(TMP)_2(CH_2Ph)\}$ in C_6D_{12}. On the other hand, the estimated molecular weight (DSE) found for NaTMP (593 g·mol^{-1}) suggests that it could be participating in different speciation equilibria, as in the monomeric {(PMDETA)NaTMP} (336.54 g·mol^{-1}) and homoleptic dimeric $\{(PMDETA)NaTMP\}_2$ (673.08 g·mol^{-1}) species and the heteroleptic trimeric structure $\{(PMDETA)_2Na_3(TMP)_2(CH_2Ph)\}$ shown above, which is in agreement with our theoretical calculations.

D(average – $NaCH_2Ph$) = 2.872e^{-10} m^2·s^{-1} D(average – NaTMP) = 3.449e^{-10} m^2·s^{-1}

D(C_6D_{12}) = 10.60e^{-10} m^2·s^{-1}

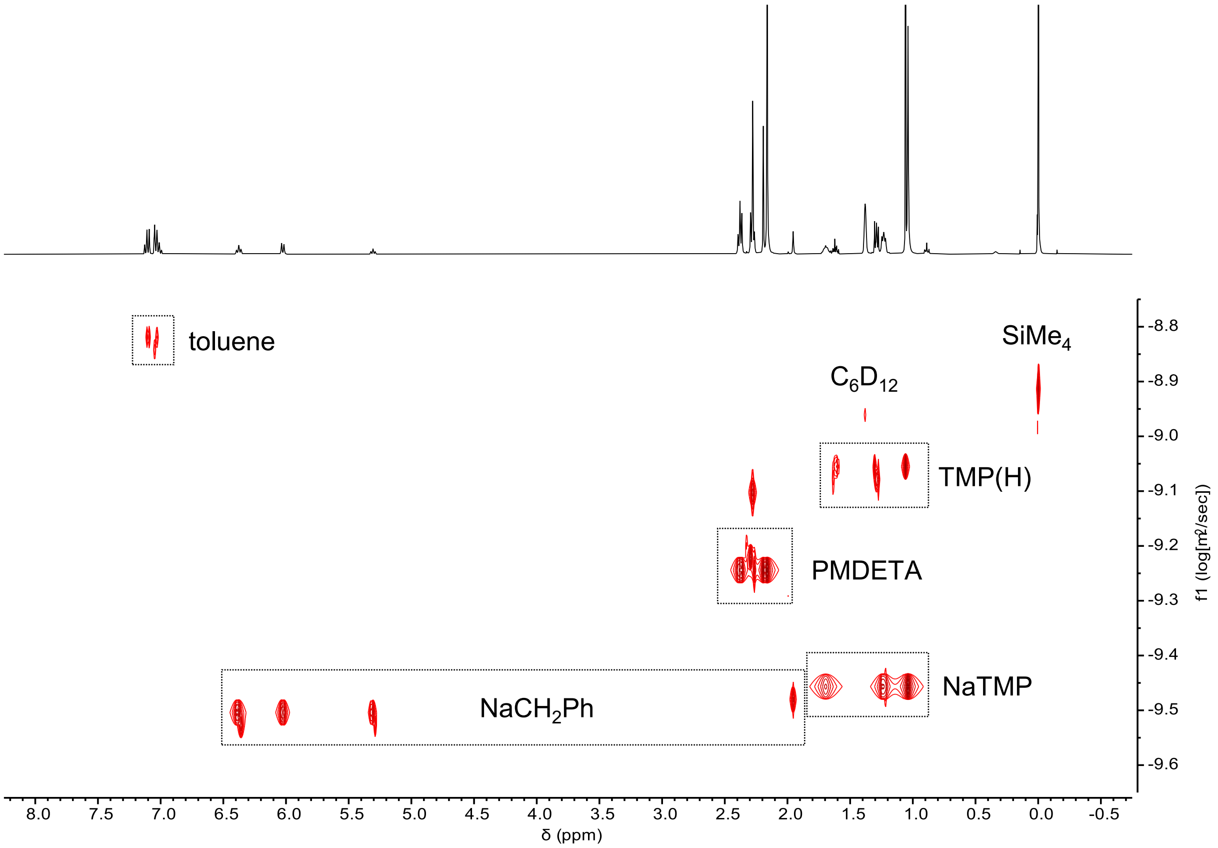

Figure S128. ^{1}H DOSY NMR spectrum of in situ generated BnNa from toluene and NaTMP in C_6D_{12}.

X-Ray Crystallographic Details

{(PMDETA)Na(2-CH_2Naph)} (I)

A crystal of $C_{20}H_{32}N_3Na$ immersed in parabar oil was mounted at 173K using the X-TEMP2[24-25] cold temperature device under the microscope, carried to the diffractometer inside a dewar containing liquid nitrogen and then transferred into a stream of gaseous nitrogen (100 K). All measurements were made on a *RIGAKU XtaLAB Synergy R*, HyPix-Arc 100 area-detector diffractometer using mirror optics monochromated Mo *K*α radiation (λ = 0.71073 Å). The unit cell constants and an orientation matrix for data collection were obtained from a least-squares refinement of the setting angles of reflections in the range 2.381° < θ < 29.020°. A total of 3484 frames were collected using ω scans, with 2.8 seconds exposure time, a rotation angle of 0.25° per frame, a crystal-detector distance of 43.0 mm, at T = 100.01(10) K. Data reduction was performed using the *CrysAlisPro* program. The intensities were corrected for Lorentz and polarization effects, and a numerical absorption correction based on gaussian integration over a multifaceted crystal model with additional empirical absorption correction using spherical harmonics using SCALE3 ABSPACK in *CrysAlisPro* was applied. The structure was solved by intrinsic phasing using *SHELXT*,[26] which revealed the positions of all non-hydrogen atoms of the title compound. All non-hydrogen atoms were refined anisotropically. H-atoms were assigned in geometrically calculated positions and refined using a riding model where each H-atom was assigned a fixed isotropic displacement parameter with a value equal to 1.2Ueq of its parent atom (1.5Ueq for methyl groups). Refinement of the structure was carried out on F^2 using full-matrix least-squares procedures, which minimized the function $\Sigma w(F_o^2 - F_c^2)^2$. The weighting scheme was based on counting statistics and included a factor to downweight the intense reflections. All calculations were performed using the *SHELXL-2014/7*[27] program in OLEX2.[28]

{(PMDETA)Na_2[(C_5H_4)Fe(C_5H_5)](TMP)}$_2$ (II)

A crystal of $C_{56}H_{100}Fe_2N_8Na_4$ immersed in parabar oil was mounted at 173K using the X-TEMP2[24-25] cold temperature device under the microscope, carried to the diffractometer inside a dewar containing liquid nitrogen and then transferred into a stream of gaseous nitrogen (173 K). All measurements were made on a *RIGAKU XtaLAB Synergy R*, HyPix-Arc 100 area-detector diffractometer using mirror optics monochromated Mo *K*α radiation (λ = 0.71073 Å). The unit cell constants and an orientation matrix for data collection were obtained from a least-squares refinement of the setting angles of reflections in the range 2.361° < θ < 33.614°. A total of 1734 frames were collected using ω scans, with 1.3 seconds exposure time, a rotation angle of 0.5° per frame, a crystal-detector distance of 43.0 mm, at T = 173.00(10) K. Data reduction was performed using the *CrysAlisPro* program. The intensities were corrected for Lorentz and polarization effects, and a numerical absorption correction based on gaussian integration over a multifaceted crystal model with additional empirical absorption correction using spherical harmonics using SCALE3 ABSPACK in *CrysAlisPro* was applied. The structure was solved by intrinsic phasing using *SHELXT*,[26] which revealed the positions of all non-hydrogen atoms of the title compound. All non-hydrogen atoms were refined anisotropically. H-atoms were assigned in geometrically calculated positions and refined using a riding model where each H-atom was assigned a fixed isotropic displacement parameter with a value equal

to 1.2Ueq of its parent atom (1.5Ueq for methyl groups). Refinement of the structure was carried out on F^2 using full-matrix least-squares procedures, which minimized the function $\Sigma w(F_o^2 - F_c^2)^2$. The weighting scheme was based on counting statistics and included a factor to downweight the intense reflections. All calculations were performed using the *SHELXL-2014/7*[27] program in OLEX2.[28] Disorder model was used for the structure where the occupancies of each disorder component was refined through the use of a free variable. The sum of equivalent components was constrained to 1, i.e. 100%.

{(PMDETA)Na(1-Me-2-indolyl)}$_2$ (III)

A crystal of $C_{36}H_{62}N_8Na_2$ immersed in parabar oil was mounted at ambient conditions and transferred into the stream of nitrogen (173 K). All measurements were made on a *RIGAKU Synergy S* area-detector diffractometer using mirror optics monochromated Cu *K*α radiation (λ = 1.54184 Å). The unit cell constants and an orientation matrix for data collection were obtained from a least-squares refinement of the setting angles of reflections in the range 4.504° < θ < 80.064°. A total of 11298 frames were collected using ω scans, with 0.4 seconds exposure time (0.6 s for high-angle reflections), a rotation angle of 0.5° per frame, a crystal-detector distance of 31.0 mm, at T = 173.00(10) K. Data reduction was performed using the *CrysAlisPro* program. The intensities were corrected for Lorentz and polarization effects, and a numerical absorption correction based on gaussian integration over a multifaceted crystal model with additional empirical absorption correction using spherical harmonics using SCALE3 ABSPACK in *CrysAlisPro* was applied. The structure was solved by intrinsic phasing using *SHELXT*,[26] which revealed the positions of all non-hydrogen atoms of the title compound. All non-hydrogen atoms were refined anisotropically. H-atoms were assigned in geometrically calculated positions and refined using a riding model where each H-atom was assigned a fixed isotropic displacement parameter with a value equal to 1.2Ueq of its parent atom (1.5Ueq for methyl groups). Refinement of the structure was carried out on F^2 using full-matrix least-squares procedures, which minimized the function $\Sigma w(F_o^2 - F_c^2)^2$. The weighting scheme was based on counting statistics and included a factor to downweight the intense reflections. All calculations were performed using the *SHELXL-2014/7*[27] program in OLEX2.[28] Disorder model was used for parts of the structure where the occupancies of each disorder component was refined through the use of a free variable. The sum of equivalent components was constrained to 1, i.e. 100%.

{(PMDETA)Na(Et$_3$SiCHPh)} (IV)

A crystal of $C_{22}H_{44}N_3NaSi$ immersed in parabar oil was mounted at 173K using the X-TEMP2[24-25] cold temperature device under the microscope, carried to the diffractometer inside a dewar containing liquid nitrogen and then transferred into a stream of gaseous nitrogen (173 K). All measurements were made on a *RIGAKU Synergy S* area-detector diffractometer using mirror optics monochromated Cu *K*α radiation (λ = 1.54184 Å). The unit cell constants and an orientation matrix for data collection were obtained from a least-squares refinement of the setting angles of reflections in the range 4.076° < θ < 79.882°. A total of 5982 frames were collected using ω scans, with 0.5 seconds exposure time (0.8 s for high-angle reflections), a rotation angle of 0.5° per frame, a crystal-detector distance of 31.0 mm, at T = 173.00(10) K. Data reduction was performed using the *CrysAlisPro* program. The intensities were corrected

for Lorentz and polarization effects, and a numerical absorption correction based on gaussian integration over a multifaceted crystal model with additional empirical absorption correction using spherical harmonics using SCALE3 ABSPACK in *CrysAlisPro* was applied. The structure was solved by intrinsic phasing using *SHELXT*,[26] which revealed the positions of all non-hydrogen atoms of the title compound. All non-hydrogen atoms were refined anisotropically. H-atoms were assigned in geometrically calculated positions and refined using a riding model where each H-atom was assigned a fixed isotropic displacement parameter with a value equal to 1.2Ueq of its parent atom (1.5Ueq for methyl groups), except for that attached to C1, where the hydrogen atom was located from the difference density map and had its position and isotropic displacement parameter refined freely. Refinement of the structure was carried out on F^2 using full-matrix least-squares procedures, which minimized the function $\Sigma w(F_o^2 - F_c^2)^2$. The weighting scheme was based on counting statistics and included a factor to downweight the intense reflections. All calculations were performed using the *SHELXL-2014/7*[27] program in OLEX2.[28]

2,7-Bis(triisopropylsilyl)pyrene (4m)

A crystal of $C_{34}H_{50}Si_2$ immersed in parabar oil was mounted at ambient conditions and transferred into a stream of nitrogen (173 K). All measurements were done on a *RIGAKU Synergy S* area-detector diffractometer using mirror optics monochromated Cu *K*α radiation (λ = 1.54184 Å). The unit cell constants and an orientation matrix for data collection were obtained from a least-squares refinement of the setting angles of reflections in the range 4.569° < θ < 80.159°. A total of 12098 frames were collected using ω scans, with 0.3 seconds exposure time (0.6 s for high-angle reflections), a rotation angle of 0.5° per frame, a crystal-detector distance of 31.0 mm, at T = 173.0(1) K. Data reduction was performed using the *CrysAlisPro* program. The intensities were corrected for Lorentz and polarization effects, and a numerical absorption correction based on gaussian integration over a multifaceted crystal model with additional empirical absorption correction using spherical harmonics using SCALE3 ABSPACK in *CrysAlisPro* was applied. The structure was solved by intrinsic phasing using *SHELXT*,[26] which revealed the positions of all non-hydrogen atoms of the title compound. All non-hydrogen atoms were refined anisotropically. All hydrogen atoms were found in the Fourier difference map and were refined freely, but isotropically. Refinement of the structure was carried out on F^2 using full-matrix least-squares procedures, which minimized the function $\Sigma w(F_o^2 - F_c^2)^2$. The weighting scheme was based on counting statistics and included a factor to downweight the intense reflections. All calculations were performed using the *SHELXL-2014/7*[27] program in OLEX2.[28]

Table S2. Selected crystallographic parameters for compounds **I**, **II**, **III**, **IV** and **4m**.

	I	II	III	IV	4m
**CCDC Number**	2512326	2512327	2512328	2512329	2512330
**Empirical Formula**	$C_{20}H_{32}N_3Na$	$C_{56}H_{100}Fe_2N_8Na_4$	$C_{36}H_{62}N_8Na_2$	$C_{22}H_{44}N_3NaSi$	$C_{34}H_{50}Si_2$
**Formula Weight**	337.47	1089.09	652.91	401.68	514.92
**Temperature / K**	100.01(10)	173.00(10)	173.00(10)	173.00(10)	100.01(10)
**Crystal System**	monoclinic	orthorhombic	monoclinic	orthorhombic	monoclinic
**Space Group**	$P2_1/c$	$C222_1$	$P2_1/n$	$Pna2_1$	$P2_1/n$
**a / Å**	13.7187(4)	15.1114(2)	10.15290(8)	16.76107(10)	8.05030(10)
**b / Å**	8.8375(2)	18.6687(3)	12.73204(10)	14.12974(7)	15.00830(10)
**c / Å**	17.2905(4)	21.5488(4)	15.30651(11)	10.87529(5)	12.57050(10)
**α / ˚**	90	90	90	90	90
**β / ˚**	100.529(2)	90	93.5041(7)	90	95.1060(10)
**γ / ˚**	90	90	90	90	90
**Volume / Å^3**	2061.00(9)	6079.15(17)	1974.93(3)	2575.59(2)	1512.76(2)
**Z**	4	4	2	4	2
**ρ_{calc}g / cm^3**	1.088	1.190	1.098	1.036	1.130
**μ / mm^{-1}**	0.082	0.547	0.703	1.031	1.194
**F(000)**	736.0	2352.0	712.0	888.0	564.0
**Crystal size / mm^3**	0.79 × 0.33 × 0.12	0.391 × 0.23 × 0.207	0.185 × 0.171 × 0.119	0.433 × 0.252 × 0.209	0.26 × 0.24 × 0.19
**Radiation**	Mo Kα (λ = 0.71073)	Mo Kα (λ = 0.71073)	Cu Kα (λ = 1.54184)	Cu Kα (λ = 1.54184)	Cu Kα (λ = 1.54184)
**2Θ range for data collection / °**	4.792 to 61.012	4.364 to 52.744	9.042 to 148.986	8.184 to 148.95	9.198 to 148.91
**Index ranges**	$-19 \le h \le 19$, $-12 \le k \le 12$, $-23 \le l \le 24$	$-18 \le h \le 18$, $-23 \le k \le 23$, $-26 \le l \le 26$	$-12 \le h \le 12$, $-15 \le k \le 15$, $-19 \le l \le 19$	$-20 \le h \le 20$, $-16 \le k \le 17$, $-13 \le l \le 13$	$-10 \le h \le 10$, $-18 \le k \le 18$, $-15 \le l \le 15$
**Reflections collected**	51164	74560	78571	53736	58731
**Independent reflections**	6257 [R_{int} = 0.0208, R_{sigma} = 0.0144]	6198 [R_{int} = 0.0583, R_{sigma} = 0.0226]	4041 [R_{int} = 0.0382, R_{sigma} = 0.0132]	4933 [R_{int} = 0.0261, R_{sigma} = 0.0163]	3090 [R_{int} = 0.0493, R_{sigma} = 0.0127]
**Data/restraints/parameters**	6257/414/420	6198/306/511	4041/126/291	4933/1/256	3090/0/263
**Goodness-of-fit on F^2**	1.073	1.120	1.087	1.049	1.069
**Final R indexes [I>=2σ (I)]**	R_1 = 0.0678, wR_2 = 0.1964	R_1 = 0.1012 wR_2 = 0.2370	R_1 = 0.0415 wR_2 = 0.1205	R_1 = 0.0276 wR_2 = 0.0765	R_1 = 0.0353 wR_2 = 0.0961
**Final R indexes [all data]**	R_1 = 0.0928, wR_2 = 0.2152	R_1 = 0.1017, wR_2 = 0.2372	R_1 = 0.0452, wR_2 = 0.1235	R_1 = 0.0285, wR_2 = 0.0774	R_1 = 0.0359, wR_2 = 0.0965
**Largest diff. peak/hole / e Å^{-3}**	0.41/-0.29	0.55/-1.46	0.31/-0.19	0.23/-0.11	0.32/-0.34
**Flack parameter**	-	0.35(7)	-	0.004(10)	-

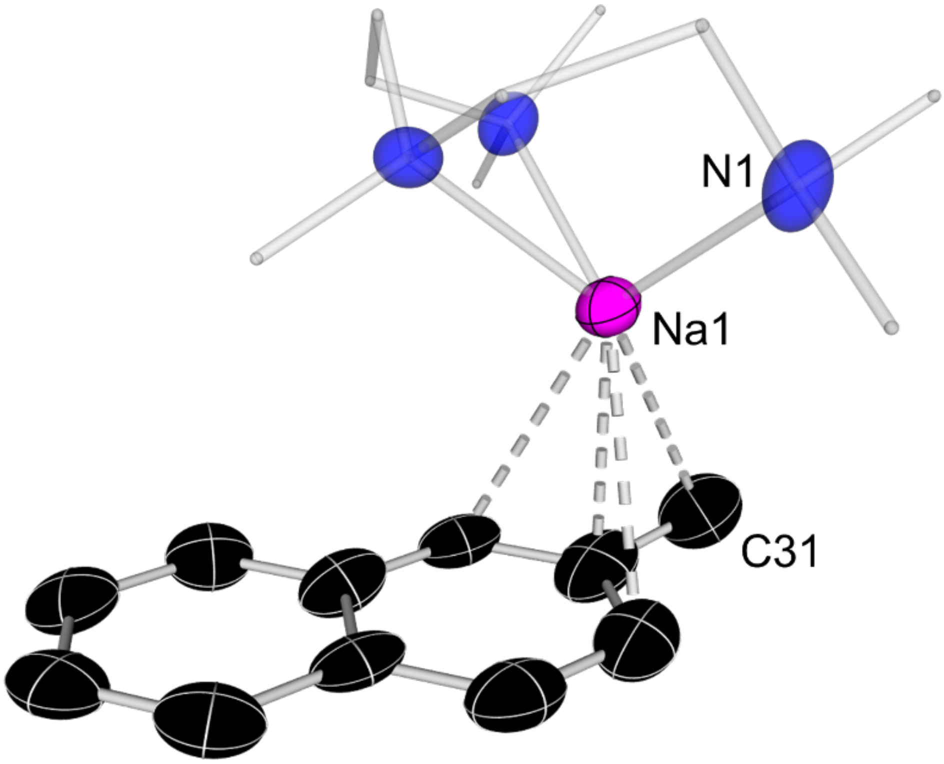

Figure S129. Molecular structure of {(PMDETA)Na(2-CH_2Naph)} (**I**) with 30% probability displacement ellipsoids. Hydrogen atoms have been omitted for clarity.

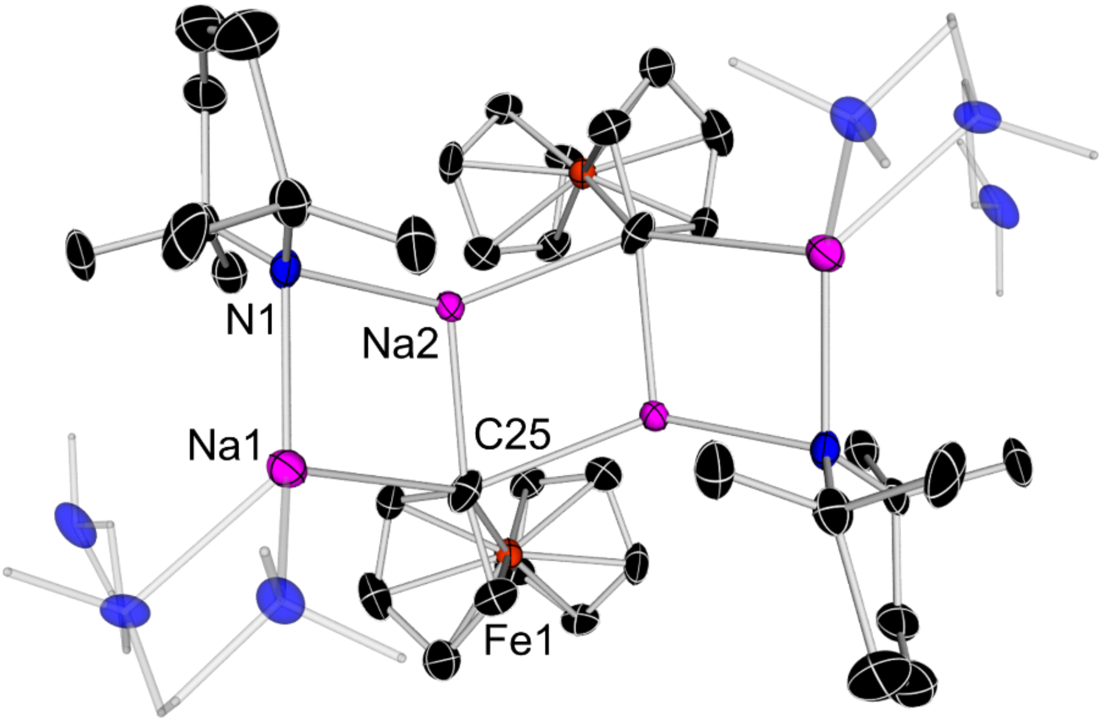

Figure S130. Molecular structure of {(PMDETA)Na_2[(C_5H_4)Fe(C_5H_5)](TMP)}$_2$ (**II**) with 30% probability displacement ellipsoids. Hydrogen atoms have been omitted for clarity.

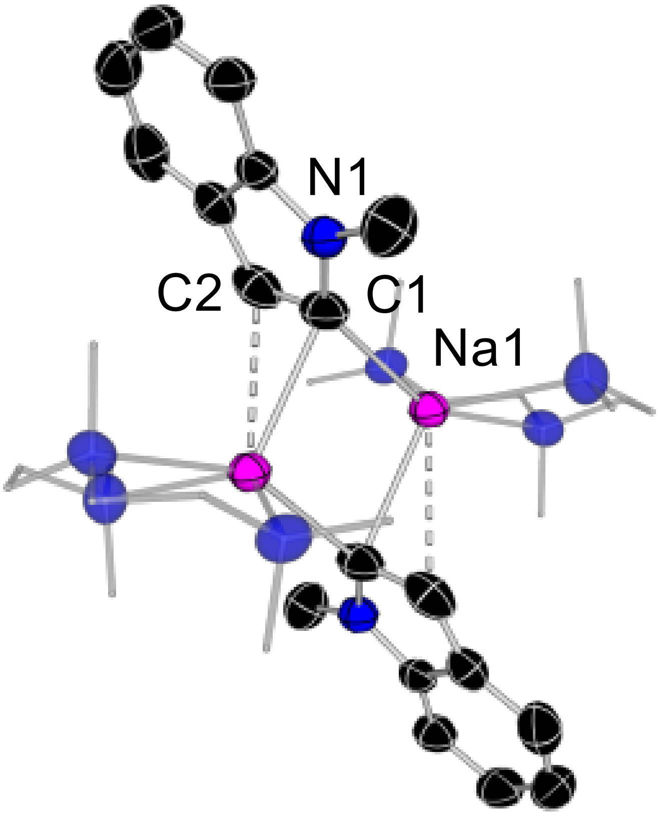

Figure S131. Molecular structure of {(PMDETA)Na(1-Me-2-indolyl)}$_2$ (**III**) with 30% probability displacement ellipsoids. Hydrogen atoms have been omitted for clarity.

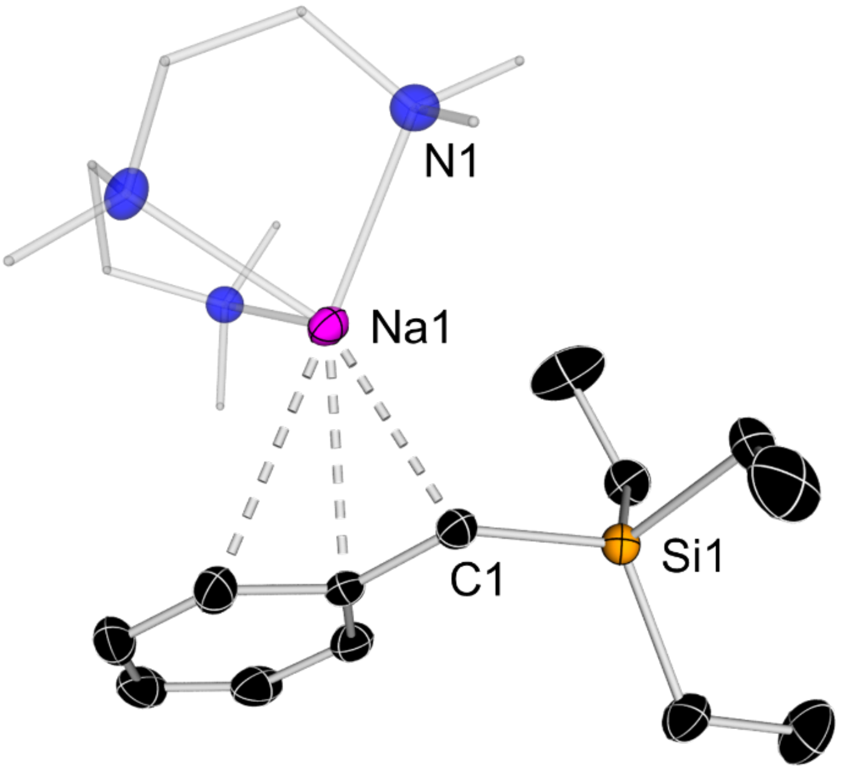

Figure S132. Molecular structure of {(PMDETA)Na(Et$_3$SiCHPh)} (**IV**) with 30% probability displacement ellipsoids. Hydrogen atoms have been omitted for clarity.

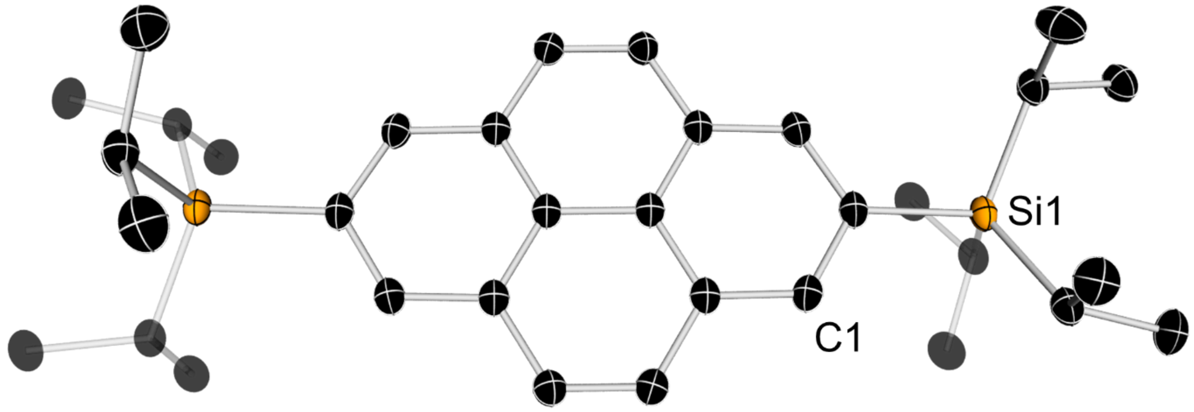

Figure S133. Molecular structure of 2,7-bis(triisopropylsilyl)pyrene (**4m**) with 30% probability displacement ellipsoids. Hydrogen atoms have been omitted for clarity.

Computational Methods

The reaction mechanism for the silylation of toluene was elucidated through density functional theory (DFT) calculations using the dispersion-corrected hybrid exchange-correlation functional ωB97X-D,[29] as implemented in the Gaussian16 software.[30]

Geometry optimizations were performed in vacuum without imposing any symmetry constraints. The Na, Si, C, and H atoms were described using the 6-31g(d,p) double- ζ basis set, while the same basis set with an additional diffuse function, 6-31+g(d,p) was employed to describe the more electronegative N and Cl atoms. Frequency calculations were performed at the same level of theory to obtain the Gibbs energy corrections at the experimental conditions (298.15 K, 1 atm), as well as to confirm the nature of the stationary points, with energy minima displaying only real frequencies while transition states exhibit one imaginary frequency. Additionally, the optimized transition states were relaxed in both directions along the reaction coordinate to confirm that they connect the desired energy minima.

Solvent effects were accounted for through the implicit solvent model based on density (SMD) using *n*-hexane (ε = 1.8819).[31] Single-point calculations with the extended basis set 6-311g(d,p) for Na, Si, C and H, and 6-311+g(d,p) for N and Cl, were carried out to obtain the Gibbs solvation energies reported in this work.

Standard state corrections were applied by adding (or subtracting) 1.90 kcal·mol^{-1} to the computed Gibbs energies for every additional molecule with respect to the products in a given reaction step.[32]

All the DFT data underlying this work, including the cartesian coordinates and energies of all the modeled structures, is openly accessible via the following ioChem-BD online dataset:

https://iochem-bd.bsc.es/browse/handle/100/479506

Alternative Transition State Structures and Energies

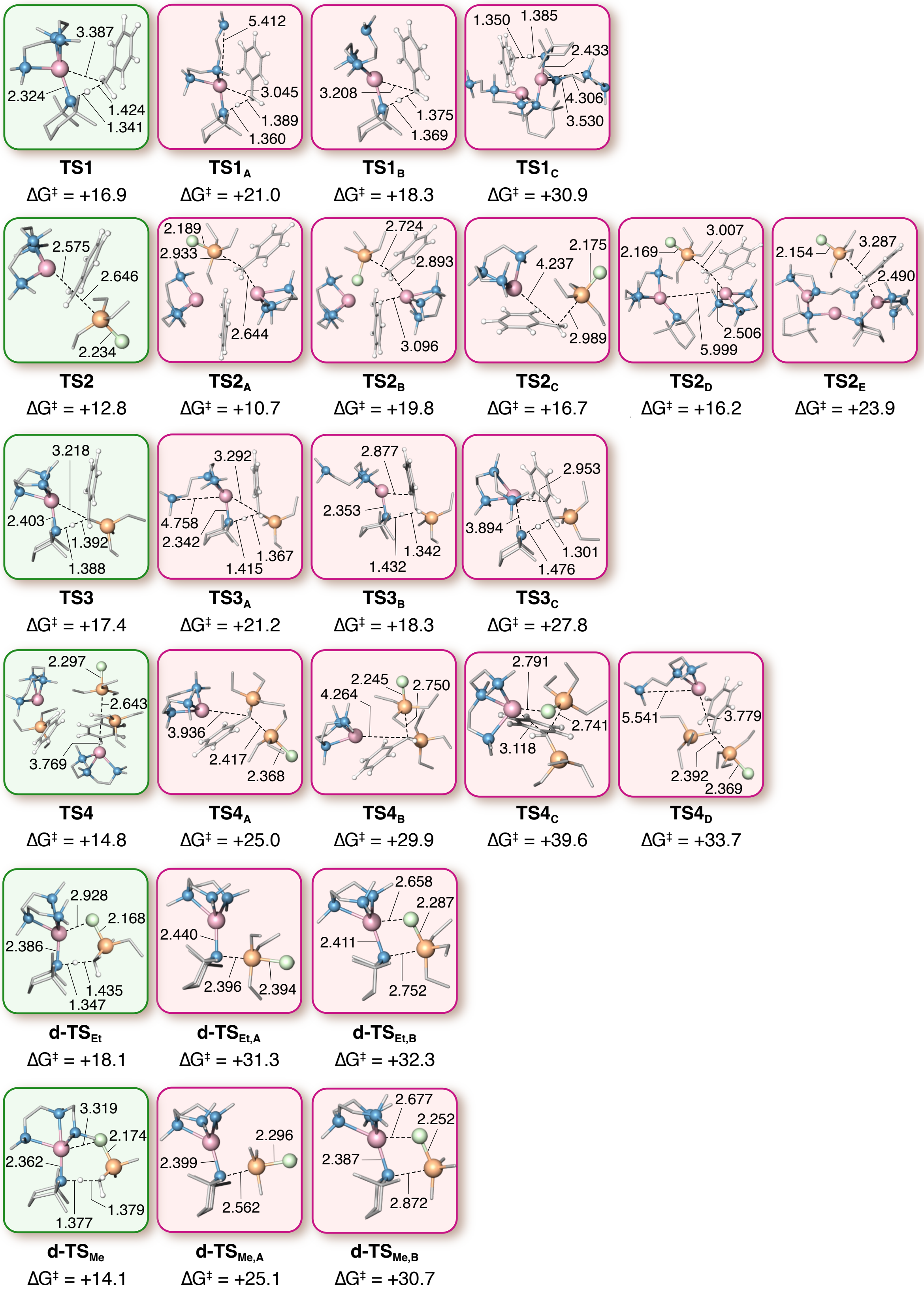

Figure S134. DFT-optimized structures and corresponding Gibbs energies of alternative transition states modelled. Gibbs energies are reported in kcal·mol^{-1}, relative to **R1** for **TS1**, **TS2**, **TS3**, and **d-TS$_{Me/Et}$**, and relative to **I8$_{dimer}$** for **TS4**, at the experimental conditions of 298.15 K and 1 atm in *n*-hexane. Relevant bond lengths are shown in Å and some H atoms have been omitted for clarity.

Gibbs Energy Profile for the Decompositions of R_3SiCl (R = Me, Et, *i*Pr)

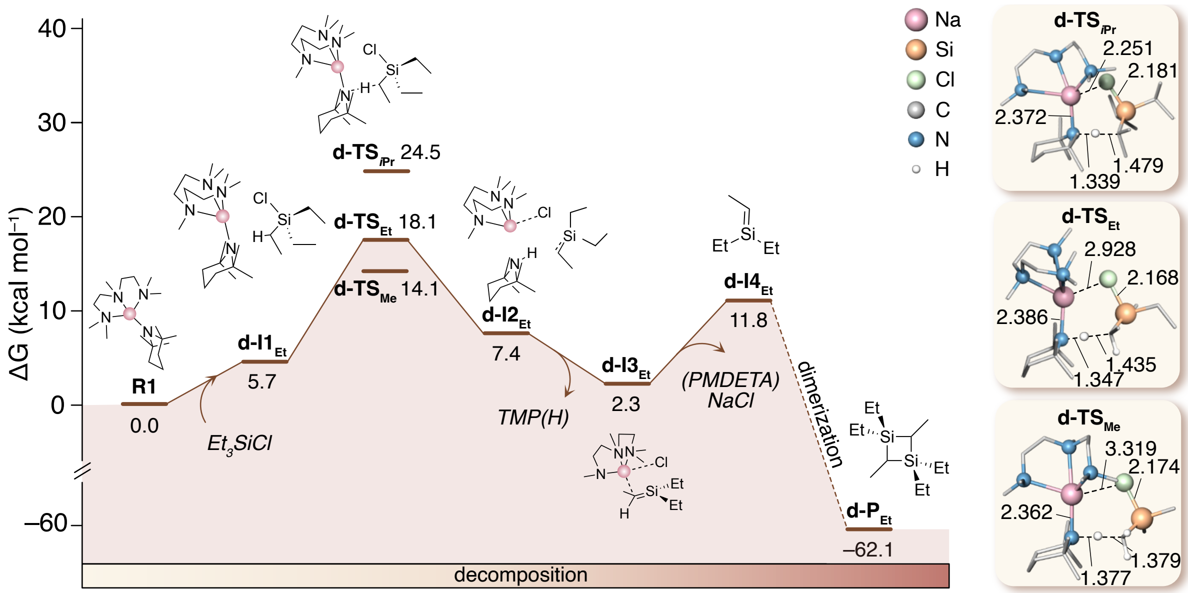

Figure S135. Gibbs energy profile for the decomposition of Et_3SiCl with (PMDETA)NaTMP to form product **d-P$_{Et}$** via the α-deprotonation pathway. Gibbs energies are reported in kcal·mol^{-1} relative to (PMDETA)NaTMP (**R1**) under experimental conditions (298.15 K, 1 atm, *n*-hexane). Insets show the transition state structures for R = Me, Et, and *i*Pr with selected bond lengths in Å. H atoms are omitted for clarity where appropriate.

Conformational Analysis

The conformational space of complexes containing flexible ethyl groups was explored using the systematic rotor search as implemented in Avogadro[33] with the UFF forcefield. The rotatable C–C ethyl bonds (highlighted in orange in Figure S132) were sampled by 120° torsional increments, while constraining all other atoms excluding the methyl substituents directly attached to the rotated bonds. In the case of **TS2/TS2$_{dimer}$**, the ethyl groups of the silane undergoing S_N2 substitution adopt a planar arrangement perpendicular to the C–Si–Cl axis, thereby restricting conformational freedom. Consequently, no rotor search was required. All generated conformers were optimized in the gas phase (ωB97X-D: 6-31g(d,p)/6-31+g(d,p)); only the lowest energy structures are reported in this work.

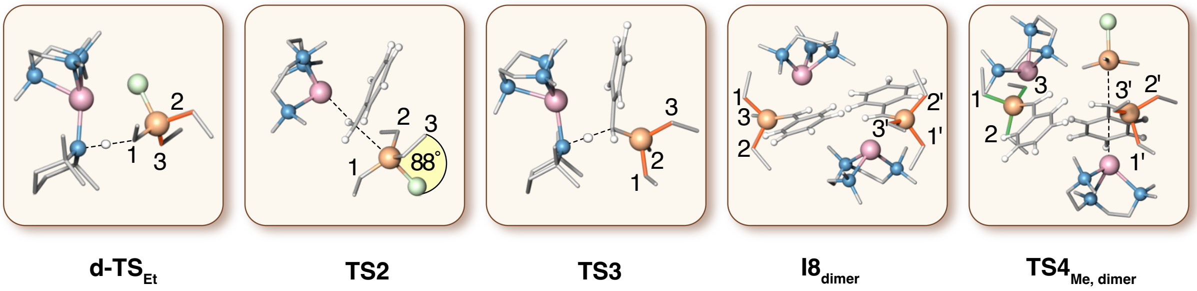

Figure S136. DFT-optimized structures of intermediates and transition states showing the rotatable bonds labelled and highlighted in orange. H atoms are omitted for clarity where appropriate.

Table S3. Electronic (E_{gas}) and Gibbs (G_{gas}) gas-phase energies of the 9 optimized conformers of **d-TS$_{Et}$**. The ethyl group (Et_1, Figure S132) undergoing deprotonation was kept fixed, while the remaining two ethyl groups (Et_2, Et_3) were allowed to rotate to generate nine conformers. E_{gas} and G_{gas} are given in Ha, while $\Delta\Delta E_{gas}$ and $\Delta\Delta G_{gas}$ are given in kcal·mol^{-1}, relative to **d-TS$_{Et}$**.

	E_{gas}	$\Delta\Delta E_{gas}$	G_{gas}	$\Delta\Delta G_{gas}$
**d-TS$_{Et}$**	–2079.059871	0.0	–2078.340456	0.0
**d-TS$_{Et}$ conf 2**	–2079.057429	+1.5	–2078.337796	+1.7
**d-TS$_{Et}$ conf 3**	–2079.056188	+2.3	–2078.337495	+1.9
**d-TS$_{Et}$ conf 4**	–2079.056524	+2.1	–2078.336867	+2.3
**d-TS$_{Et}$ conf 5**	–2079.056312	+2.2	–2078.336524	+2.5
**d-TS$_{Et}$ conf 6**	–2079.056179	+2.3	–2078.336160	+2.7
**d-TS$_{Et}$ conf 7**	–2079.054917	+3.1	–2078.334740	+3.6
**d-TS$_{Et}$ conf 8**	–2079.055443	+2.8	–2078.334193	+3.9
**d-TS$_{Et}$ conf 9**	–2079.055128	+3.0	–2078.333748	+4.2

Table S4. Electronic (E_{gas}) and Gibbs (G_{gas}) gas-phase energies of the 27 optimized conformers of **TS3**, obtained by rotating all three ethyl groups (Et_1, Et_2, Et_3) of the benzylic silane. E_{gas} and G_{gas} are given in Ha, while $\Delta\Delta E_{gas}$ and $\Delta\Delta G_{gas}$ are given in kcal·mol^{-1}, relative to **TS3**.

	E_{gas}	$\Delta\Delta E_{gas}$	G_{gas}	$\Delta\Delta G_{gas}$
**TS3**	–1889.723196	0.0	–1888.888815	0.0
**TS3 conf 2**	–1889.721868	+0.8	–1888.888500	+0.2
**TS3 conf 3**	–1889.723087	+0.1	–1888.888261	+0.3
**TS3 conf 4**	–1889.723249	0.0	–1888.888204	+0.4
**TS3 conf 5**	–1889.723513	–0.2	–1888.888156	+0.4
**TS3 conf 6**	–1889.724076	–0.6	–1888.888120	+0.4
**TS3 conf 7**	–1889.724076	–0.6	–1888.888102	+0.4
**TS3 conf 8**	–1889.721775	+0.9	–1888.887907	+0.6
**TS3 conf 9**	–1889.721676	+1.0	–1888.887695	+0.7
**TS3 conf 10**	–1889.722242	+0.6	–1888.886679	+1.3
**TS3 conf 11**	–1889.722439	+0.5	–1888.886558	+1.4
**TS3 conf 12**	–1889.720450	+1.7	–1888.886010	+1.8
**TS3 conf 13**	–1889.721734	+0.9	–1888.885812	+1.9
**TS3 conf 14**	–1889.720876	+1.5	–1888.885672	+2.0
**TS3 conf 15**	–1889.721261	+1.2	–1888.885521	+2.1
**TS3 conf 16**	–1889.719978	+2.0	–1888.885273	+2.2
**TS3 conf 17**	–1889.720476	+1.7	–1888.885200	+2.3
**TS3 conf 18**	–1889.720889	+1.4	–1888.885144	+2.3
**TS3 conf 19**	–1889.720800	+1.5	–1888.885059	+2.4
**TS3 conf 20**	–1889.720389	+1.8	–1888.885016	+2.4
**TS3 conf 21**	–1889.719146	+2.5	–1888.884420	+2.8
**TS3 conf 22**	–1889.719397	+2.4	–1888.884297	+2.8
**TS3 conf 23**	–1889.719397	+2.4	–1888.884297	+2.8
**TS3 conf 24**	–1889.720191	+1.9	–1888.884037	+3.0
**TS3 conf 25**	–1889.717127	+3.8	–1888.882302	+4.1
**TS3 conf 26**	–1889.716240	+4.4	–1888.880501	+5.2
**TS3 conf 27**	–1889.716461	+4.2	–1888.880221	+5.4

Table S5. Electronic (E_{gas}) and Gibbs (G_{gas}) gas-phase energies of the 27 optimized conformers of **I8$_{dimer}$**. E_{gas} and G_{gas} are given in Ha, while $\Delta\Delta E_{gas}$ and $\Delta\Delta G_{gas}$ are given in kcal·mol^{-1}, relative to **I8$_{dimer}$**. To exploit the molecular symmetry of **I8$_{dimer}$** and reduce computational costs, the lowest-energy conformation of one moiety (Et_1, Et_2, Et_3) was mirrored onto the second ($Et_{1'}$, $Et_{2'}$, $Et_{3'}$).

	E_{gas}	$\Delta\Delta E_{gas}$	G_{gas}	$\Delta\Delta G_{gas}$
**I8$_{dimer}$**	–2961.348116	0.0	–2960.171469	0.0
**I8$_{dimer}$ conf 2**	–2961.348141	0.0	–2960.171510	0.0
**I8$_{dimer}$ conf 3**	–2961.345844	+1.4	–2960.169893	+1.0
**I8$_{dimer}$ conf 4**	–2961.345322	+1.8	–2960.169531	+1.2
**I8$_{dimer}$ conf 5**	–2961.344213	+2.4	–2960.169156	+1.5
**I8$_{dimer}$ conf 6**	–2961.345828	+1.4	–2960.169080	+1.5
**I8$_{dimer}$ conf 7**	–2961.346889	+0.8	–2960.168947	+1.6
**I8$_{dimer}$ conf 8**	–2961.344110	+2.5	–2960.168875	+1.6
**I8$_{dimer}$ conf 9**	–2961.345260	+1.8	–2960.168841	+1.6
**I8$_{dimer}$ conf 10**	–2961.342489	+3.5	–2960.168790	+1.7
**I8$_{dimer}$ conf 11**	–2961.344057	+2.5	–2960.168741	+1.7
**I8$_{dimer}$ conf 12**	–2961.344057	+2.5	–2960.168528	+1.8
**I8$_{dimer}$ conf 13**	–2961.344227	+2.4	–2960.168118	+2.1
**I8$_{dimer}$ conf 14**	–2961.341742	+4.0	–2960.167961	+2.2
**I8$_{dimer}$ conf 15**	–2961.343045	+3.2	–2960.167821	+2.3
**I8$_{dimer}$ conf 16**	–2961.344430	+2.3	–2960.167735	+2.3
**I8$_{dimer}$ conf 17**	–2961.344371	+2.3	–2960.167613	+2.4
**I8$_{dimer}$ conf 18**	–2961.344604	+2.2	–2960.167558	+2.5
**I8$_{dimer}$ conf 19**	–2961.345523	+1.6	–2960.167515	+2.5
**I8$_{dimer}$ conf 20**	–2961.341234	+4.3	–2960.167154	+2.7
**I8$_{dimer}$ conf 21**	–2961.344601	+2.2	–2960.167037	+2.8
**I8$_{dimer}$ conf 22**	–2961.342714	+3.4	–2960.166964	+2.8
**I8$_{dimer}$ conf 23**	–2961.343336	+3.0	–2960.166873	+2.9
**I8$_{dimer}$ conf 24**	–2961.342714	+3.4	–2960.166395	+3.2
**I8$_{dimer}$ conf 25**	–2961.344181	+2.5	–2960.166295	+3.2
**I8$_{dimer}$ conf 26**	–2961.344472	+2.3	–2960.166181	+3.3
**I8$_{dimer}$ conf 27**	–2961.343375	+3.0	–2960.166003	+3.4

Table S6. Electronic (E_{gas}) and Gibbs (G_{gas}) gas-phase energies of the optimized conformers of **TS4$_{Me, dimer}$**. E_{gas} and G_{gas} are given in Ha, while $\Delta\Delta E_{gas}$ and $\Delta\Delta G_{gas}$ are given in kcal·mol^{-1}, relative to **TS4$_{Me, dimer}$**. For **TS4$_{Me, dimer}$**, the ethyl groups (Et_1, Et_2, Et_3) of the non-reactive moiety were kept fixed in the lowest-energy geometry previously determined for **I8$_{dimer}$**. The three ethyl groups ($Et_{1'}$, $Et_{2'}$, $Et_{3'}$) of the reactive moiety were rotated to generate 27 conformers. The same conformation was used for **TS4$_{Et, dimer}$**, as the ethyl groups of the silane undergoing S_N2 remain perpendicular to the C–Si–Cl axis.

	E_{gas}	$\Delta\Delta E_{gas}$	G_{gas}	$\Delta\Delta G_{gas}$
**TS4$_{Me, dimer}$**	–3830.806039	0.0	–3829.520097	0.0
**TS4$_{Me, dimer}$ conf 2**	–3830.806039	0.0	–3829.520098	0.0
**TS4$_{Me, dimer}$ conf 3**	–3830.804097	+1.2	–3829.517773	+1.5
**TS4$_{Me, dimer}$ conf 4**	–3830.803240	+1.8	–3829.517365	+1.7
**TS4$_{Me, dimer}$ conf 5**	–3830.804611	+0.9	–3829.517279	+1.8
**TS4$_{Me, dimer}$ conf 6**	–3830.805439	+0.4	–3829.517061	+1.9
**TS4$_{Me, dimer}$ conf 7**	–3830.803984	+1.3	–3829.515824	+2.7
**TS4$_{Me, dimer}$ conf 8**	–3830.802232	+2.4	–3829.515628	+2.8
**TS4$_{Me, dimer}$ conf 9**	–3830.804353	+1.1	–3829.515374	+3.0
**TS4$_{Me, dimer}$ conf 10**	–3830.800089	+3.7	–3829.515030	+3.2
**TS4$_{Me, dimer}$ conf 11**	–3830.804169	+1.2	–3829.514644	+3.4
**TS4$_{Me, dimer}$ conf 12**	–3830.800327	+3.6	–3829.514560	+3.5
**TS4$_{Me, dimer}$ conf 13**	–3830.799845	+3.9	–3829.513711	+4.0
**TS4$_{Me, dimer}$ conf 14**	–3830.797755	+5.2	–3829.513621	+4.1
**TS4$_{Me, dimer}$ conf 15**	–3830.799622	+4.0	–3829.513316	+4.3
**TS4$_{Me, dimer}$ conf 16**	–3830.798420	+4.8	–3829.513179	+4.3
**TS4$_{Me, dimer}$ conf 17**	–3830.799829	+3.9	–3829.512045	+5.1
**TS4$_{Me, dimer}$ conf 18**	–3830.797702	+5.2	–3829.511871	+5.2
**TS4$_{Me, dimer}$ conf 19**	–3830.799698	+4.0	–3829.511159	+5.6
**TS4$_{Me, dimer}$ conf 10**	–3830.795422	+6.7	–3829.511029	+5.7
**TS4$_{Me, dimer}$ conf 21**	–3830.794992	+6.9	–3829.509065	+6.9
**TS4$_{Me, dimer}$ conf 22**	–3830.796227	+6.2	–3829.508853	+7.1
**TS4$_{Me, dimer}$ conf 23**	–3830.795885	+6.4	–3829.508846	+7.1
**TS4$_{Me, dimer}$ conf 24**	–3830.797193	+5.6	–3829.507779	+7.7
**TS4$_{Me, dimer}$ conf 25**	–3830.794079	+7.5	–3829.506879	+8.3
**TS4$_{Me, dimer}$ conf 26**	–3830.792395	+8.6	–3829.505679	+9.0
**TS4$_{Me, dimer}$ conf 27**	–3830.791471	+9.1	–3829.503093	+10.7

The conformational space of **I3**$_{\textbf{het-trimer}}$ was explored using PCMODEL (v.10.0)[34] with the MMX forcefield. The rotatable bonds shown in Figure S133a were allowed to rotate with a termination criterion of 10,000 duplicate conformers and an energy window of 20 kcal·mol^{-1}. For more detailed setting of PCMODEL see below. The resulting 873 conformers were divided into 17 distinct clusters using the k-means clustering method, the number distinct clusters was determined by the elbow method.[35] Cluster validity was assessed through UMAP dimensionality reduction,[36] which provided a visual confirmation of the clustering performance, as shown in Figure S133b. A single representative structure from each k-mean cluster's most central data point was selected and reoptimized with DFT, and the lowest energy was selected. The python script to perform this analysis is openly accessible on GitHub via the link:

https://github.com/Manting-Mu/post-processing-small-scale-conformer-search.git

PCMODEL settings

Energy Window: 20

Calculation Method: Gmmx

Boltzmann Temp: 298

Min Cutoff Distance: 0.25

Min Cutoff Energy: 0.5

Emin found x Times: 5

Duplicate Conf found x Times: 20

Min # Conf Minimized: 100

Max # Conf Maximized: 10,000

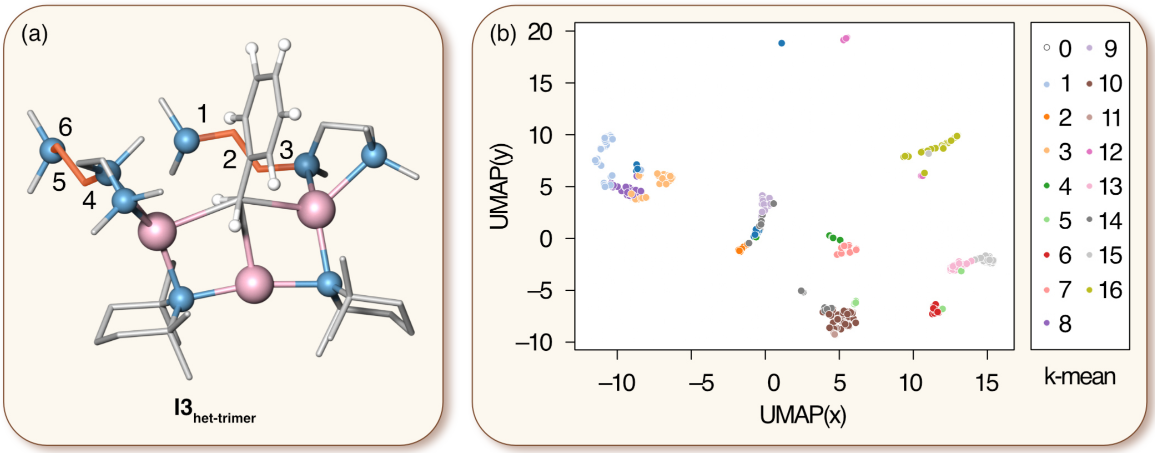

Figure S137. (a) DFT-optimized structure of **I3**$_{\textbf{het-trimer}}$ showing the rotatable bonds labelled and highlighted in orange. H atoms are omitted for clarity where appropriate. (b) The conformers are plotted in the reduced 2-dimensional UMAP space with each K-cluster color by a distinct color from 0 to 16.

Gibbs Energy Profile for the Silylation of Toluene with Et_3SiCl via the Dimeric Pathway

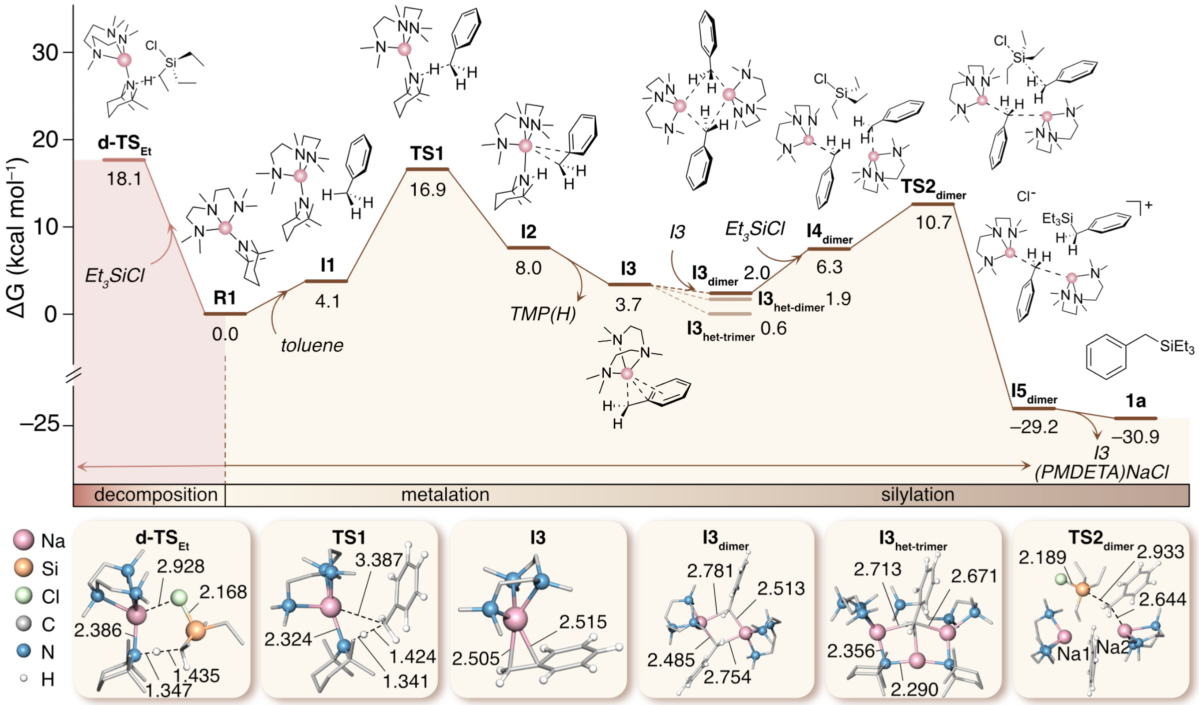

Figure S138. Gibbs energy profile for the silylation of toluene with Et_3SiCl to form product **1a** via the dimeric pathway (right), and for the decomposition of Et_3SiCl with (PMDETA)NaTMP (left). Gibbs energies are reported in kcal·mol^{-1} relative to (PMDETA)NaTMP (**R1**) under experimental conditions (298.15 K, 1 atm, *n*-hexane). Insets show key intermediate and transition state structures with selected bond lengths in Å. H atoms are omitted for clarity where appropriate.

Microkinetic Modelling

Due to the presence of various silylation transition states of similar energies (**TS2**$_{\mathbf{A-E}}$, Figure S130), a microkinetic model was constructed using the software COPASI[37] to investigate their contributions towards the formation of the silylated product **1a**. With the DFT-calculated energy barriers of each elementary step, we determined the forward (k_1) rate constant using the Eyring equation (Eq. 1), taking the transmission coefficient (κ) as unity.

$$k_1 = \kappa \frac{k_B T}{h} e^{-\frac{\Delta G^{\ddagger}}{RT}} \tag{1}$$

Based on a combination of diffusion and collision theories,[37] a simple expression for the diffusion-controlled rate constant (k_D) can be obtained using the solvent viscosity η and temperature T (Eq. 2).

$$k_D = \frac{8 k_B T}{3\eta} \tag{2}$$

Using the viscosity of *n*-hexane (η = 0.3 mPa at 298 K),[38-39] this relation gave the diffusion-controlled rate constant ($k_D = 2.20\times10^{10}$ M·s^{-1}), which corresponds to a barrier of +3.3 kcal·mol^{-1}.

The Gibbs energy (Eq. 3) and equilibrium (Eq. 4) equations with the DFT-calculated energy of intermediates and k_1 were used to obtain the reverse (k_{-1}) rate constants for each elementary step, constructing the microkinetic model summarized in Figure S135.

$$\Delta G^{\circ} = -RT ln(K) \tag{3}$$

$$K = \frac{k_1}{k_{-1}} \tag{4}$$

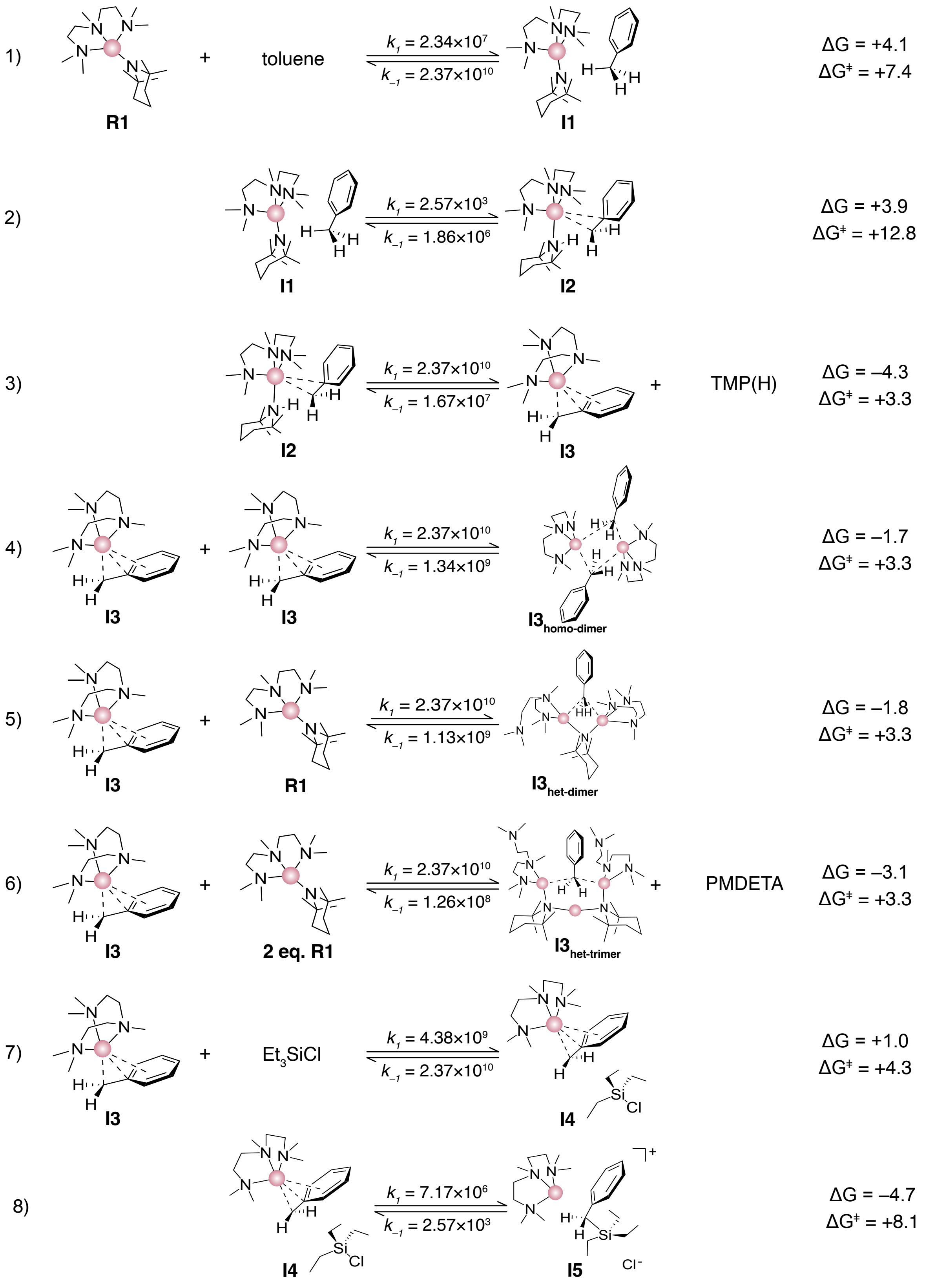

Figure S139. Microkinetic model used to simulate the time-concentration profiles for the silylation of toluene with Et_3SiCl, using the DFT-calculated energies at the experimental conditions (298.15 K, 1 atm, *n*-hexane). Gibbs energies are reported in kcal·mol^{-1} and rate constants in s^{-1} and L·mol^{-1}·s^{-1} for first- and second-order reactions, respectively.

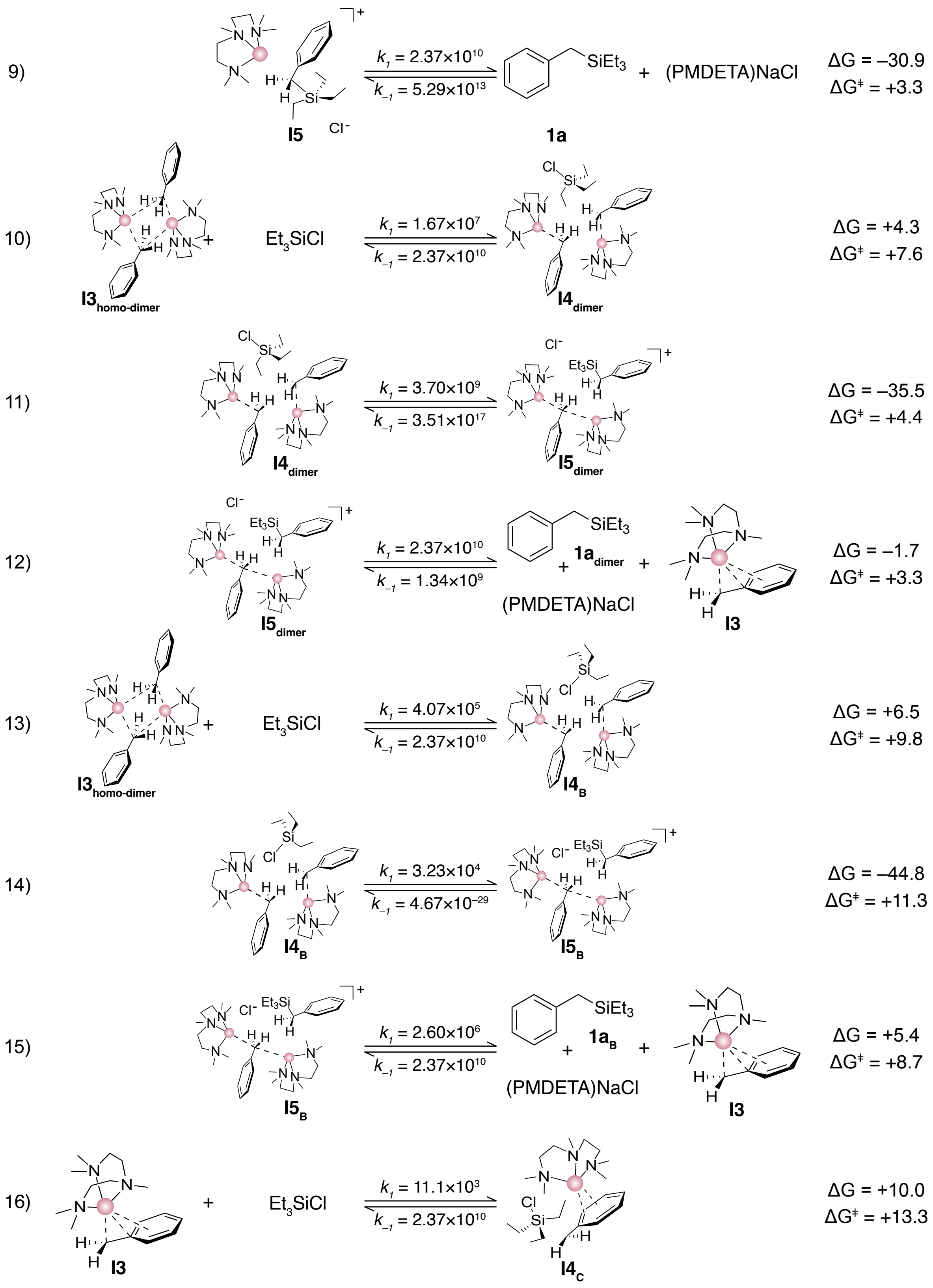

Figure S139 (continued). Microkinetic model used to simulate the time-concentration profiles for the silylation of toluene with Et_3SiCl, using the DFT-calculated energies at the experimental conditions (298.15 K, 1 atm, *n*-hexane). Gibbs energies are reported in kcal·mol^{-1} and rate constants in s^{-1} and L·mol^{-1}·s^{-1} for first- and second-order reactions, respectively.

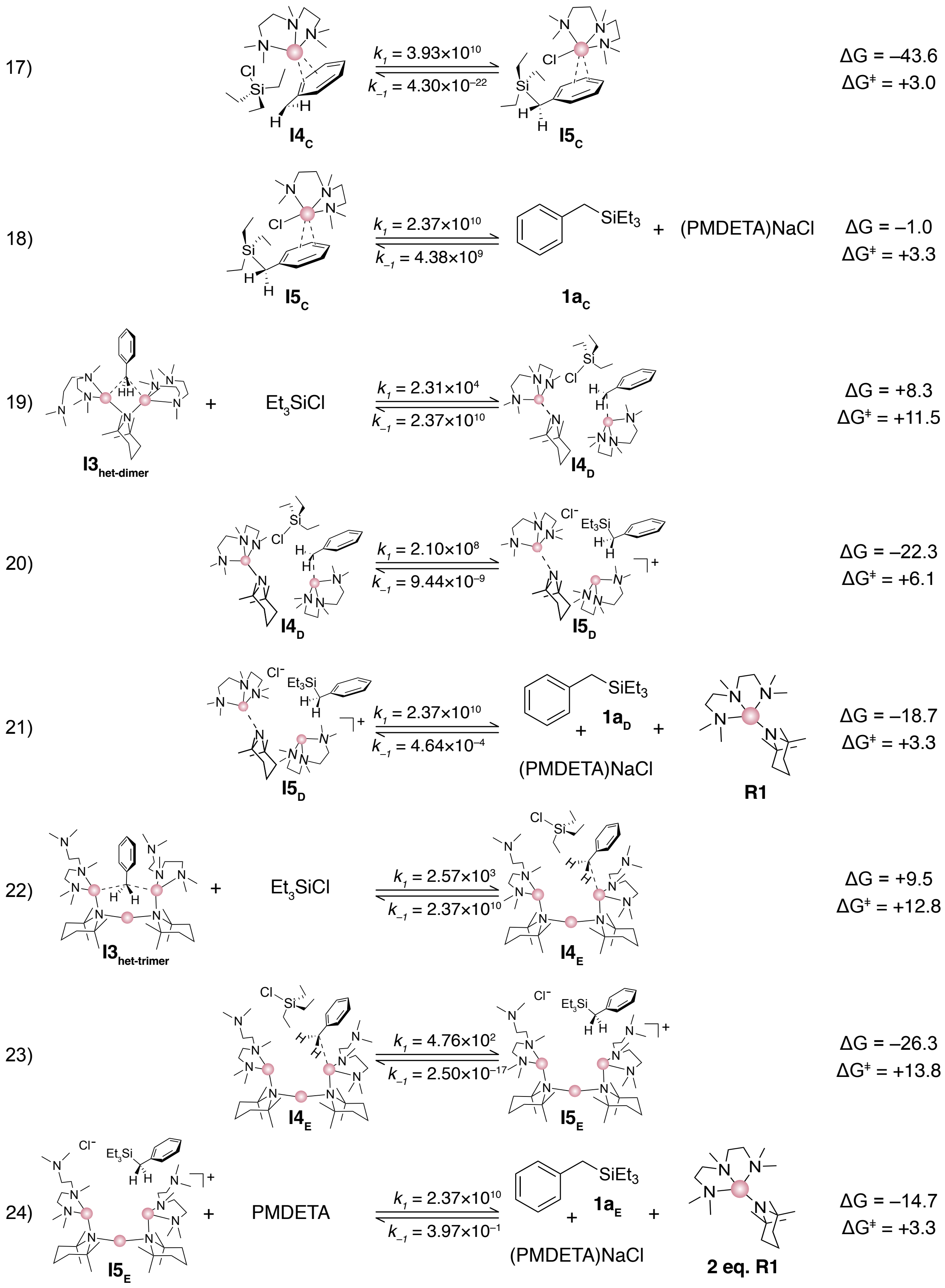

Figure S139 (continued). Microkinetic model used to simulate the time-concentration profiles for the silylation of toluene with Et_3SiCl, using the DFT-calculated energies at the experimental conditions (298.15 K, 1 atm, *n*-hexane). Gibbs energies are reported in kcal·mol^{-1} and rate constants in s^{-1} and L·mol^{-1}·s^{-1} for first- and second-order reactions, respectively.

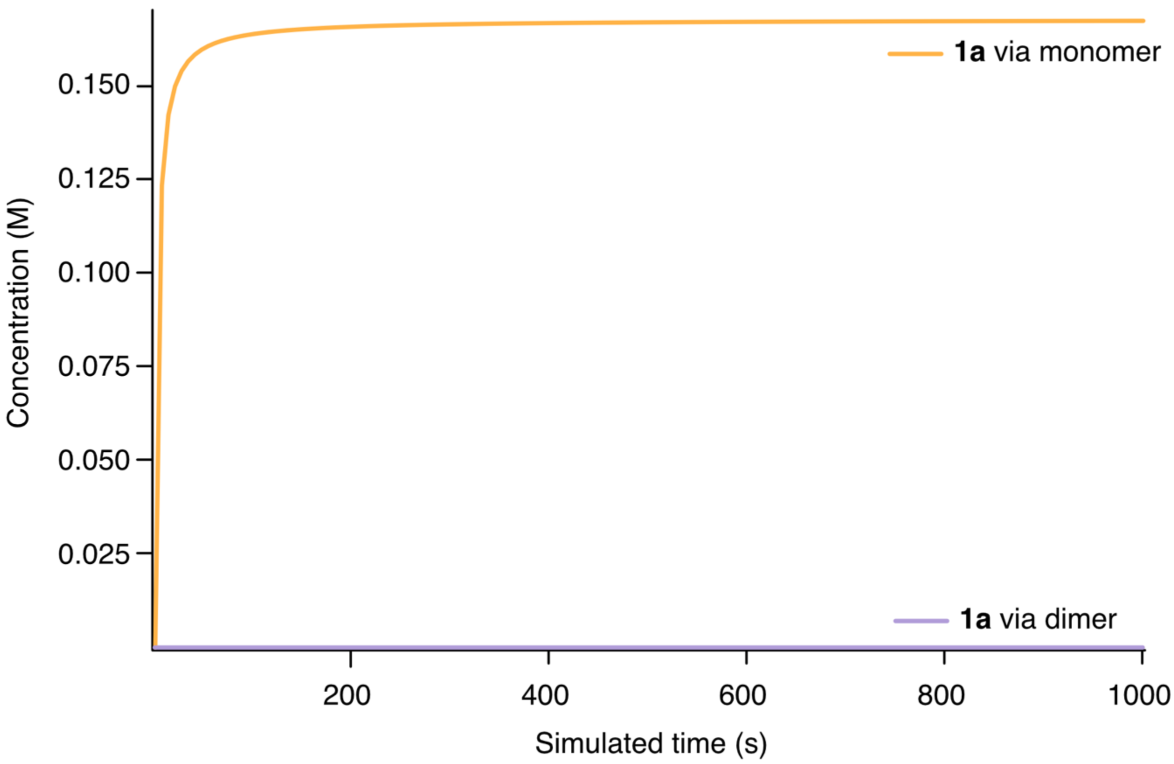

Figure S140. Simulated concentration profile depicting the evolution of **1a** formed via the monomeric (orange) and dimeric (purple) pathways, using the rate constants in Figure S135 and the experimental concentrations of [(PMDETA)NaTMP]$_0$ = [Et_3SiCl]$_0$ = [toluene]$_0$ = 0.167 M.

Gibbs Energy Profile for the Association of (PMDETA)Na^+ and Cl^-

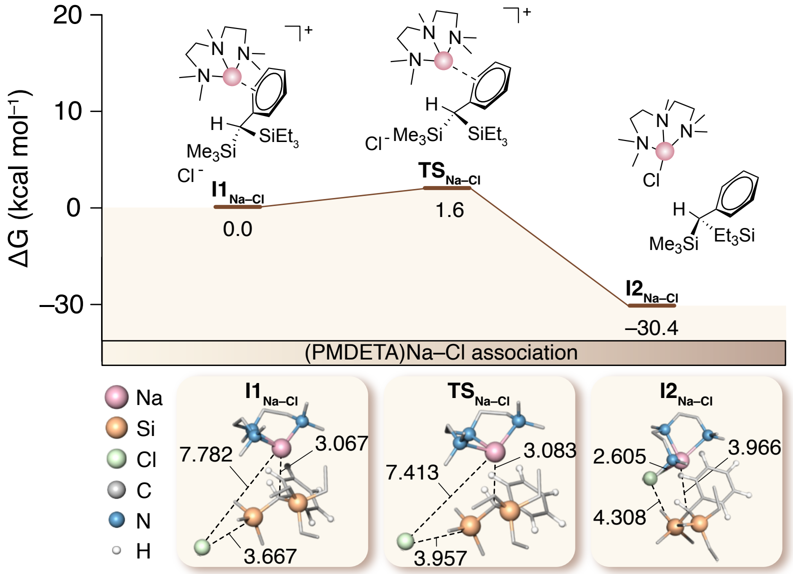

Figure S141. Gibbs energy profile for the migration of Cl^- through *n*-hexane to form (PMDETA)NaCl and the disilylated product (where R_1 = Me, R_2 = Et). Gibbs energies are reported in kcal·mol^{-1} relative to $\mathbf{I1_{Na–Cl}}$, where the (PMDETA)Na^+ and Cl^- moieties are separated by the disilylated product, under experimental conditions (298.15 K, 1 atm, *n*-hexane). The insets show key intermediate and transition state structures with selected bond lengths in Å. H atoms are omitted for clarity where appropriate.

Gibbs Energy Profile for the Silylation of 1a with Et_3SiCl

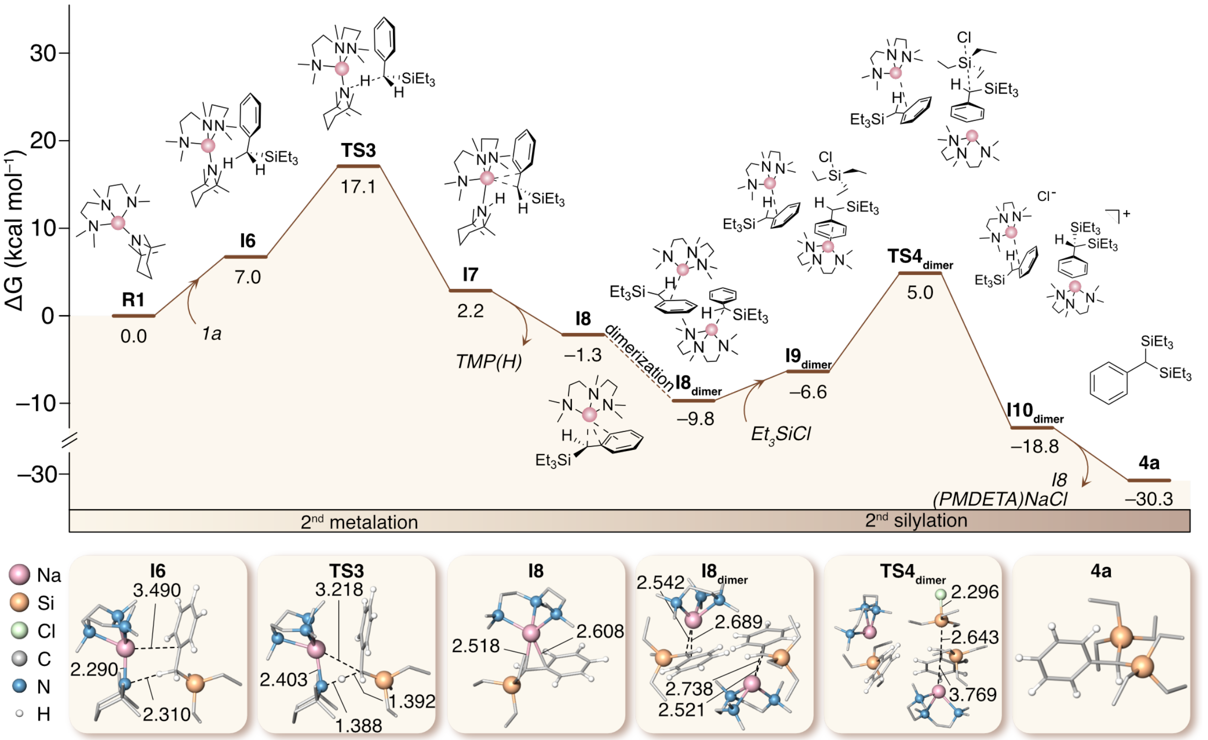

Figure S142. Gibbs energy profile for the silylation of **1a** with Et_3SiCl to form product **4a**. Gibbs energies are reported in kcal·mol^{-1} relative to (PMDETA)NaTMP (**R1**) under experimental conditions (298.15 K, 1 atm, *n*-hexane). Insets show key intermediate and transition state structures with selected bond lengths in Å. H atoms are omitted for clarity where appropriate.

Gibbs Energy Profile for the Silylation of Toluene with Me_3SiCl

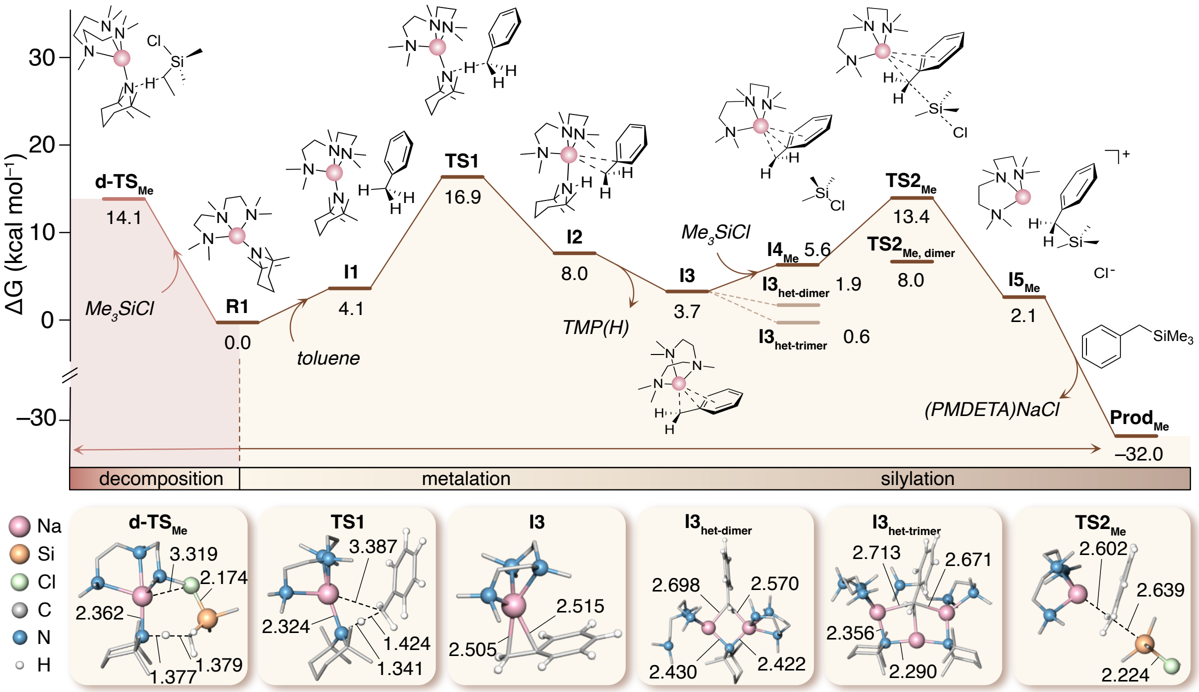

Figure S143. Gibbs energy profile for the silylation of toluene with Me_3SiCl to form **Prod$_{Me}$** via the monomeric pathway (right), and for the decomposition of Me_3SiCl with (PMDETA)NaTMP (left). Gibbs energies are reported in kcal·mol^{-1} relative to (PMDETA)NaTMP (**R1**) under experimental conditions (298.15 K, 1 atm, *n*-hexane). Insets show key intermediate and transition state structures with selected bond lengths in Å. H atoms are omitted for clarity where appropriate.

Gibbs Energy Profile for the Silylation of 1a with Me_3SiCl

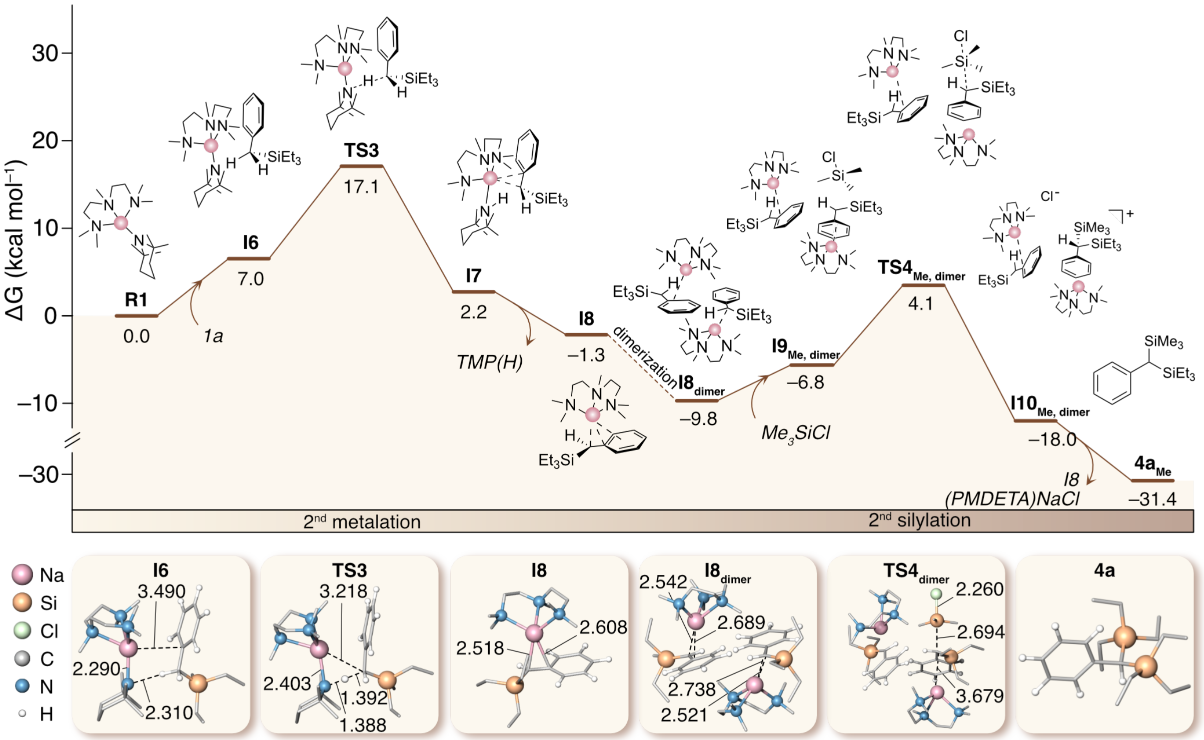

Figure S144. Gibbs energy profile for the silylation of **1a** with Me_3SiCl to form product $\mathbf{4a_{Me}}$. Gibbs energies are reported in kcal·mol^{-1} relative to (PMDETA)NaTMP (**R1**) under experimental conditions (298.15 K, 1 atm, *n*-hexane). Insets show key intermediates and transition states with selected bond lengths in Å. H atoms are omitted for clarity where appropriate.

References

[1] R. Neufeld, D. Stalke, *Chem. Sci.* **2015**, *6*, 3354-3364.

[2] C. C. Gruber, G. Oberdorfer, C. V. Voss, J. M. Kremsner, C. O. Kappe, W. Kroutil, *J. Org. Chem.* **2007**, *72*, 5778-5783.

[3] A. Tortajada, D. E. Anderson, E. Hevia, *Helv. Chim. Acta* **2022**, *105*, e202200060.

[4] E. Hevia, A. R. Kennedy, R. E. Mulvey, D. L. Ramsay, S. D. Robertson, *Chem. Eur. J.* **2013**, *19*, 14069-14075.

[5] D. E. Anderson, A. Tortajada, E. Hevia, *Angew. Chem. Int. Ed.* **2023**, *62*, e202218498.

[6] B. Neil, L. Saadi, L. Fensterbank, C. Chauvier, *Angew. Chem. Int. Ed.* **2023**, *62*, e202306115.

[7] Z. Liu, J. Huo, T. Fu, H. Tan, F. Ye, M. L. Hossain, J. Wang, *Chem. Commun.* **2018**, *54*, 11419-11422.

[8] E.-H. Wang, Y.-J. Ping, Z.-R. Li, H. Qin, Z.-J. Xu, C.-M. Che, *Org. Lett.* **2018**, *20*, 4641-4644.

[9] C. Zarate, M. Nakajima, R. Martin, *J. Am. Chem. Soc.* **2017**, *139*, 1191-1197.

[10] Y. Yuan, Y. Gu, Y.-E. Wang, J. Zheng, J. Ji, D. Xiong, F. Xue, J. Mao, *J. Org. Chem.* **2022**, *87*, 13907-13918.

[11] C. C. Mak, M. K. Tse, K. S. Chan, *J. Org. Chem.* **1994**, *59*, 3585-3589.

[12] Y. Fukumoto, M. Hirano, N. Chatani, *ACS Catal.* **2017**, *7*, 3152-3156.

[13] X.-W. Liu, C. Zarate, R. Martin, *Angew. Chem. Int. Ed.* **2019**, *58*, 2064-2068.

[14] Z. Xu, L. Chai, Z.-Q. Liu, *Org. Lett.* **2017**, *19*, 5573-5576.

[15] A. A. Toutov, W.-B. Liu, K. N. Betz, A. Fedorov, B. M. Stoltz, R. H. Grubbs, *Nature* **2015**, *518*, 80-84.

[16] S. Chen, X. Guo, H. Hou, S. Geng, Z. Liu, Y. He, X.-S. Xue, Z. Feng, *Angew. Chem. Int. Ed.* **2023**, *62*, e202303470.

[17] B. Neil, F. Lucien, L. Fensterbank, C. Chauvier, *ACS Catal.* **2021**, *11*, 13085-13090.

[18] C. Gao, S. A. Blum, *J. Org. Chem.* **2022**, *87*, 13124-13137.

[19] B. Lu, J. R. Falck, *Angew. Chem. Int. Ed.* **2008**, *47*, 7508-7510.

[20] H.-J. Lee, C. Kwak, D.-P. Kim, H. Kim, *Green Chem.* **2021**, *23*, 1193-1199.

[21] M. Murai, N. Nishinaka, K. Takai, *Angew. Chem. Int. Ed.* **2018**, *57*, 5843-5847.

[22] M. Mato, A. M. Echavarren, *Angew. Chem. Int. Ed.* **2019**, *58*, 2088-2092.

[23] M. Uchiyama, Y. Kobayashi, T. Furuyama, S. Nakamura, Y. Kajihara, T. Miyoshi, T. Sakamoto, Y. Kondo, K. Morokuma, *J. Am. Chem. Soc.* **2008**, *130*, 472-480.

[24] T. Kottke, D. Stalke, *Journal of Appl. Crystallogr.* **1993**, *26*, 615-619.

[25] D. Stalke, *Chem. Soc. Rev.* **1998**, *27*, 171-178.

[26] G. Sheldrick, *Acta Crystallogr. A* **2015**, *71*, 3-8.

[27] G. Sheldrick, *Acta Crystallogr. C* **2015**, *71*, 3-8.

[28] O. V. Dolomanov, L. J. Bourhis, R. J. Gildea, J. A. K. Howard, H. Puschmann, *Journal of Appl. Crystallogr.* **2009**, *42*, 339-341.

[29] J.-D. Chai, M. Head-Gordon, *Phys. Chem. Chem. Phys.* **2008**, *10*, 6615-6620.

[30] Frisch, M.J., Trucks, G.W., Schlegel, H.B., Scuseria, G.E., Robb, M.A., Cheeseman, J.R.; Scalmani, G.; Barone, V.; Petersson, G.A.; Nakatsuji, H.; Li, X.; Caricato, M.; Marenich, A.V.; Bloino, J., Janesko, B.G., Gomperts, R., Mennucci, B., Hratchian, H.P., Ortiz, J.V., Izmaylov, A.F., Sonnenberg, J.L., Williams-Young, D., Ding, F., Lipparini, F., Egidi, F., Goings, J., Peng, B., Petrone, A., Henderson, T., Ranasinghe, D., Zakrzewski, V.G., Gao, J., Rega, N., Zheng, G., Liang, W., Hada, M., Ehara, M., Toyota, K., Fukuda, R., Hasegawa, J., Ishida, M., Nakajima, T., Honda, Y., Kitao, O., Nakai, H., Vreven, T., Throssell, K., Montgomery Jr., J.A., Peralta, J.E., Ogliaro, F., Bearpark, M.J., Heyd, J.J., Brothers, E.N., Kudin, K.N., Staroverov, V.N., Keith, T.A., Kobayashi, R., Normand, J., Raghavachari, K., Rendell, A.P., Burant, J.C., Iyengar, S.S., Tomasi, J., Cossi, M., Millam, J.M., Klene, M., Adamo, C., Cammi, R., Ochterski, J.W., Martin, R.L., Morokuma, K., Farkas, O., Foresman, J.B., Fox, D.J. Gaussian, Inc., Wallingford CT. Gaussian 16, Revision B.01. *2016*.

[31] A. V. Marenich, C. J. Cramer, D. G. Truhlar, *J. Phys. Chem. B* **2009**, *113*, 6378-6396.

[32] J. H. Jensen, *Phys. Chem. Chem. Phys.* **2015**, *17*, 12441-12451.

[33] M. D. Hanwell, D. E. Curtis, D. C. Lonie, T. Vandermeersch, E. Zurek, G. R. Hutchison, *J. Cheminform.* **2012**, *4*, 17.

[34] A. L. Gille, B. C. Dutmer, T. M. Gilbert, *J. Am. Chem. Soc.* **2009**, *131*, 5714-5714.

[35] S. Lloyd, *IEEE Trans. Inf. Theory* **1982**, *28*, 129-137.

[36] L. McInnes, J. Healy, J. Open Source Softw. **2018**, *3*, 861.

[37] S. Hoops, S. Sahle, R. Gauges, C. Lee, J. Pahle, N. Simus, M. Singhal, L. Xu, P. Mendes, U. Kummer, *Bioinformatics* **2006**, *22*, 3067-3074.

[38] J. N. Harvey, F. Himo, F. Maseras, L. Perrin, *ACS Catal.* **2019**, *9*, 6803-6813.

[39] J. H. Dymond, H. A. O/ye, *J. Phys. Chem. Ref. Data* **1994**, *23*, 41-53.
